# ETS-Domain Transcription Factor Elk-1 Regulates Stemness Genes in Brain Tumors and CD133+ BrainTumor-Initiating Cells

**DOI:** 10.3390/jpm11020125

**Published:** 2021-02-14

**Authors:** Melis Savasan Sogut, Chitra Venugopal, Basak Kandemir, Ugur Dag, Sujeivan Mahendram, Sheila Singh, Gizem Gulfidan, Kazim Yalcin Arga, Bayram Yilmaz, Isil Aksan Kurnaz

**Affiliations:** 1 Institute of Biotechnology, Gebze Technical University, 41400 Kocaeli, Turkey; melis.savasan.sogut@gmail.com (M.S.S.); bkandemir@baskent.edu.tr (B.K.); 2 Molecular Neurobiology Laboratory (AxanLab), Department of Molecular Biology and Genetics, Gebze Technical University, 41400 Kocaeli, Turkey; 3 Biotechnology Graduate Program, Graduate School of Sciences, Yeditepe University, 26 Agustos Yerlesimi, Kayisdagi, 34755 Istanbul, Turkey; dagu@janelia.hhmi.org; 4 Stem Cell and Cancer Research Institute, McMaster University, Hamilton, ON L8S 4K1, Canada; venugop@mcmaster.ca (C.V.); smahendram@gmail.com (S.M.); ssingh@mcmaster.ca (S.S.); 5 Department of Bioengineering, Marmara University, 34722 Istanbul, Turkey; gizemgulfidn@gmail.com (G.G.); kazim.arga@marmara.edu.tr (K.Y.A.); 6 Department of Physiology, Faculty of Medicine, Yeditepe University, 26 Agustos Yerlesimi, Kayisdagi, 34755 Istanbul, Turkey

**Keywords:** ETS, Elk-1, stem cell, microarray, brain-tumor-initiating cell (BTIC)

## Abstract

Elk-1, a member of the ternary complex factors (TCFs) within the ETS (E26 transformation-specific) domain superfamily, is a transcription factor implicated in neuroprotection, neurodegeneration, and brain tumor proliferation. Except for known targets, *c-fos* and *egr-1*, few targets of Elk-1 have been identified. Interestingly, *SMN*, *SOD1*, and *PSEN1* promoters were shown to be regulated by Elk-1. On the other hand, Elk-1 was shown to regulate the CD133 gene, which is highly expressed in brain-tumor-initiating cells (BTICs) and used as a marker for separating this cancer stem cell population. In this study, we have carried out microarray analysis in SH-SY5Y cells overexpressing Elk-1-VP16, which has revealed a large number of genes significantly regulated by Elk-1 that function in nervous system development, embryonic development, pluripotency, apoptosis, survival, and proliferation. Among these, we have shown that genes related to pluripotency, such as *Sox2*, *Nanog*, and *Oct4*, were indeed regulated by Elk-1, and in the context of brain tumors, we further showed that Elk-1 overexpression in CD133+ BTIC population results in the upregulation of these genes. When Elk-1 expression is silenced, the expression of these stemness genes is decreased. We propose that Elk-1 is a transcription factor upstream of these genes, regulating the self-renewal of CD133+ BTICs.

## 1. Introduction

The ternary complex factor (TCF) Elk-1 of the ETS domain superfamily is a ubiquitous transcription factor, yet it interacts with neuronal microtubules and motor proteins, is found mainly in neuronal axons and dendrites, and is phosphorylated at Serine 383 residue in fear conditioning or synaptic plasticity paradigms [1,2,3,4,5,6]. Phosphorylation of Elk-1 by MAPKs, in particular Serine 383 and Serine 389 within the activation domain, was shown to induce its binding to DNA [7,8].

Elk-1 transcription factor has been widely studied with respect to its mitogen-induced activation through phosphorylation by mitogen-activated protein kinases (MAPKs) and regulation of the *c-fos* promoter in complex with serum response factor (SRF) [9]. However, Elk-1 and other ternary complex factor (TCF) members have a rather large number of targets, some of which have a high degree of redundancy [10]. A thousand new promoters were identified for Elk-1 binding using a ChIP-chip assay, with two distinct binding modes: SRF-dependent and SRF-independent; furthermore, it was shown that there was a redundancy of promoter occupancy by other ETS proteins in a subset of promoters [10]. Elk-1 was also shown to regulate survival in neuronal cell models by regulating the Survival of Motor Neuron (SMN) promoter as a novel target [11]. CD133, a widely-accepted Cancer Stem Cell (CSC) marker [12,13,14], was also shown to be regulated through *ets* motifs as well as hypoxia-inducible elements, through the interaction of HIF-1α and Elk-1 on the promoter [15].

Elk-1 was recently found to have both activating and repressive role in human embryonic stem cells (hESCs), particularly through SRF interaction, and found to be upregulated in mesoderm differentiation [16].

In this study, we have first aimed to identify novel targets of Elk-1 using SH-SY5Y neuroblastoma cell line in a transcriptomics approach. We have identified novel pathways and genes that were up- or downregulated upon Elk-1-VP16 overexpression, and when promoters of a subset of these genes were analyzed, several *ets* motifs were identified. Among these, genes related to pluripotency or early neuronal development were particularly interesting, hence we have further analyzed and verified the regulation of a selected set of genes by Elk-1 using qPCR and investigated the regulation of *SOX2*, *NANOG*, and *POU5F1* promoters by Elk-1 and its binding to predicted *ets* motifs in neuroblastoma and glioblastoma (GBM) cell lines. Considering Elk-1 was previously shown to regulate CD133 expression [15], we have also studied Elk-1 expression levels in CD133− and CD133+ cell lines as well as primary brain tumors, indicating Elk-1 was indeed overexpressed in CD133+ cells, and when Elk-1 expression was silenced by RNAi, *SOX2*, and *NANOG* expression were reduced in both CD133+ primary GBMs, as well as CD133+ cell lines in a cell context-dependent manner.

## 2. Materials and Methods

### 2.1. Cell Culture and BTIC Isolation from Cell Lines and Primary Tumors

SK-N-BE (2) (ATCC CRL-2271) and SH-SY5Y (ATCC CRL-2266) human neuroblastoma cell lines as well as U-87 MG (ATCC^®^ HTB-14), A172 (ATCC CRL-1620), and T98G (ATCC CRL-1690) human GBM cell lines were used. U87-MG, A172, and T98G cell lines were provided by Assist. Prof. Tugba Bagci Onder from Koc University. For all the stated cell lines for monolayer culture, DMEM high-glucose (4.5 g/L) medium (Gibco, #41966029, Waltham, MA, USA) was used as a basal medium and supplemented with one percent penicillin-streptomycin solution (Gibco, #15140122, Rockville, MA, USA) and 10 percent fetal bovine serum (FBS) (Life Technologies, #10500064, Carlsbad, CA, USA). Cells were grown in 37 °C and 5 percent CO2 incubator.

To form tumorsphere cultures from monolayer cells and support brain-tumor-initiating cells after conducting CD133+ isolation, initial proliferation media (IPM), N2 media, and coated culture plates were used. Plates were prepared by coating with poly-HEMA (poly (2-hydroxyethyl methacrylate) solution. To prepare poly-HEMA solution, 38 mL absolute ethanol was mixed with two mL double distilled water. Following the addition of 1.2 g of poly-HEMA (Sigma Aldrich, #P3932, Taufkirchen, Germany) powder into the mixture, it was placed in a shaker at 37 °C with a vigorous shake for four-five hours until no powder could be seen with the naked eye. This poly-HEMA solution was filtered through a 0.22-micron filter and kept at 4 °C up to six months. Initial proliferation medium (IPM) is necessary for culturing tumorspheres and isolated brain-tumor-initiating cells up to three passages. IPM is made up of neurobasal medium (Gibco, #21103049, Waltham, MA, USA), 1X B27 (Gibco, #17504044, Waltham, MA, USA), 1X GlutaMAX (Gibco, #35050061, Waltham, MA, USA), one percent penicillin-streptomycin solution (Gibco, #15140122, Waltham, MA, USA), 20 ng/mL FGF-2 (Gibco, #13256029, Waltham, MA, USA), and 20 ng/mL EGF (Gibco, #SRP3027, Waltham, MA, USA). N2 medium is necessary for culturing spheroids and isolated brain-tumor-initiating cells over three passages. N2 medium is made up of neurobasal medium (Gibco, #21103049), 1X N2 (Gibco, #17502048, Waltham, MA, USA), 1X GlutaMAX (Gibco, 35050061, Waltham, MA, USA), one percent penicillin-streptomycin solution (Gibco, #15140122, Waltham, MA, USA), 20 ng/mL FGF-2 (Gibco, #13256029, Waltham, MA, USA), and 20 ng/mL EGF (Waltham, MA, USA, #SRP3027, Waltham, MA, USA).

For brain-tumor-initiating cells’ (BTICs) isolation from cell lines, SK-N-BE (2) neuroblastoma cells were grown as monolayer cells up to 80 percent confluency, and on the day of isolation, the media was removed, cells were washed with five mL PBS/flask, and three mL of StemPro Accutase/flask was added onto the cells. The suspension was centrifuged at 300× *g* for five minutes. The cells were resuspended with MACS buffer [two percent bovine serum albumin (BSA), two mM EDTA, and phosphate-buffered saline (PBS) pH 7.2]. To prevent the clogging of the columns at the ongoing isolation procedure, cells were passed through the first 70-micron cell strainer several times until they could pass freely through it. Then, they were passed through a 30-micron filter several times, cell aggregates were removed, and the single-cell suspension was prepared. Cells could be counted at this stage of the procedure. The viable cell number was determined by staining the cells with 0.4 percent Trypan Blue Solution (Gibco, #15250061, Waltham, MA, USA). To continue, cells were centrifuged at 300× *g* for 10 min, and the supernatant was removed. Cells were resuspended in 60 µL MACS buffer/10^7^ cells and 20 µL FcR Blocking Agent/10^7^ cells, and 20 µL CD133 Microbeads/10^7^ cells were added (CD133 MicroBead Kit—Tumor Tissue, human, Miltenyl Biotec, #130-100-857, Gladbach, Germany). The cells were incubated at 4 °C for 30 min at a constant, slow rotation (12 rpm). Following incubation, two mL buffer/10^7^ cells were added to wash the cells, and then, they were centrifuged at 300× *g* for 10 min again. The supernatant was aspirated, and the pellet was resuspended in 500 µL MACS buffer/10^7^ cells and continued with magnetic separation part.

MACS MS column (Miltenyl Biotec, #130-042-201, Germany) was placed on the MACS Mini Separation stand and was equilibrated with 500 µL MACS buffer. The cells prepared in the previous step were loaded onto that column, and with gravity effect, the suspended cells flow through the column for positive selection. That is, the cells labeled for CD133 (CD133+) were kept in the column, while marker-free cells (CD133−) would not bind to the column and were collected in a tube. The column was washed three times with 500 µL of the buffer to wash column-retaining CD133+ cells, the flowing liquid was collected again, and the resulting cells were combined to assemble CD133− cells. For elution of CD133+ cells, the column was separated from the magnetic stand and allowed to stand in the non-magnetic field for about two minutes and flushed out with one mL MACS buffer with the supplied plunger. Cells were counted with Trypan Blue, centrifuged for five minutes at 150× *g* and resuspended in complete IPM and cultured for 7–10 days in a humidified incubator at 37 °C and five percent CO_2_, replacing the medium with freshly prepared IPM every three–four days until their size reached 200 microns, or they started dying from the center. When they reached the limitations, they were passaged. For passaging the cells, the suspension cells were collected from the dishes to a falcon and centrifuged at 300× *g* for 10 min. Following the centrifugation, the medium was aspirated, and one mL StemPro Accutase Cell Dissociation Reagent (Gibco, #A1110501, Waltham, MA, USA) was added onto the cells, and cells were incubated at 37 °C for five minutes. Cells were triturated about 40 times until the spheroids become single-cell suspension. Onto this single-cell suspension, five mL of PBS with antibiotics was added. Cells were counted at this stage if necessary or to continue cells were centrifuged at 300× *g* for five minutes. The cells were resuspended in complete IPM at the proper volume.

### 2.2. Dissociation and Culture of Primary GBM Tissue

Human GBM samples were obtained from consenting patients, as approved by the Hamilton Health Sciences/McMaster Health Sciences Research Ethics Board. Brain tumor samples were dissociated as previously described [17] and cultured as neurospheres in Neurocult complete (NCC) media, a chemically defined serum-free neural stem cell medium (STEMCELL Technologies, Vancouver, BC, Canada), supplemented with human recombinant epidermal growth factor (20 ng/mL: STEMCELL Technologies, Vancouver, Canada), basic fibroblast growth factor (20 ng/mL; STEMCELL Technologies, Vancouver, Canada), heparin (2 μg/mL 0.2% Heparin Sodium Salt in PBS; STEMCELL Technologies, Vancouver, Canada), antibiotic-antimycotic (10 mg/mL; Wisent Bioproducts, Saint Bruno, QC, Canada) in ultra-low attachment plates (Corning, New York, NY, USA). Primary GBM cells (BT 428, BT 458 and BT 624) were cultured in NSC complete media and flow-sorted for CD133+ and CD133− populations as described previously [18,19]. Transfections were carried out by Lipofectamine 2000 as per the manufacturer’s instructions.

### 2.3. Transient Transfection of Cells

For transfection of adherent cells, single-cell suspensions of adherent cell cultures were prepared and seeded at 0.3–0.6 × 10^6^ cells/cm^2^ density in complete DMEM medium, and they were incubated in 37 °C, five percent CO_2_ incubator, so that they would be 85–90 percent confluent at the time of transfection. On day one, for the formation of the carrier liposome complex, the desired plasmid and PEI were mixed at the determined ratio for each cell line in serum-free DMEM and incubated at room temperature for 20 min. At the end of the period, a complete DMEM medium with 10 percent FBS was added to the mixture at half the volume of the mix. Two hours later, complete DMEM medium containing 10 percent FBS was added to the wells/dishes and the cells were incubated for 48 h in 37 °C, five percent CO_2_ incubator for the transgene expression. Cells were transfected with empty pCDNA3 or pCMV plasmids, pCMV-Elk-1 and pRSV-Elk-1-VP16 (courtesy of Prof. A.D. Sharrocks) using the PEI reagent (CellnTech), in 3 replicas per sample. psiSTRIKE hMGFP-scrRNA (from here on referred to as scrRNA) and psiSTRIKE hMGFP-siElk-1 (from here on referred to as siElk-1) has been described elsewhere [11].

For transfection of BTICs, the suspension cells were collected from the dishes to a falcon and centrifuged at 300× *g* for 10 min. Following the centrifugation, the medium was aspirated, and one mL StemPro Accutase Cell Dissociation Reagent (Gibco, #A1110501) was added onto the cells, and cells were incubated 37 °C for five minutes. Cells were triturated for about 40 times until the spheroids become single-cell suspension. Onto this single-cell suspension, five mL of PBS with antibiotics was added and centrifuged at 300× *g* for five minutes. The cells were resuspended in complete IPM at the proper volume. Cell density and Lipofectamine 2000 (Thermo Fischer Scientific, Waltham, MA, USA) ratio were determined. Cells were seeded at 0.3–0.6 × 10^6^ cells/cm^2^ density in complete IPM without antibiotics on the day of transfection. Following the cell seeding, Lipofectamine 2000 and the nucleic acids were diluted in neurobasal medium without antibiotics, incubated at room temperature for five minutes, then the diluted Lipofectamine 2000 was gently combined with the dilute nucleic acids, and the mixture was incubated at room temperature for 20 min to form liposome. Then, the mixture was added directly onto wells containing cells, and the cells were incubated for 24–72 h in 37 °C, 5% CO_2_ incubator for the transgene expression

### 2.4. Soft Agar Assay

For softy agar assay, 100 cells in 100 µL IPM and an equal volume of 2.8% low-melting-point (LMP) agarose solution were mixed to generate 1.4% agarose-cell solution per well in a 96-well plate, and the mixture was incubated at 37 °C, 5% CO_2_ incubator for 14 days. At the end of 14 days, colonies were counted under a 10× magnification or stereo microscope. For staining, crystal violet was dissolved in PBS with two percent ethanol at a final concentration of 0.04 percent, filtered with 0.45 µm filter, and dishes were stained with 50 µL of this solution for one hour at room temperature. The plates were checked every ten minutes to prevent the staining of the background. Then, the staining solution was removed carefully, and the wells were washed with water three times for 30 min. At the last wash, water was kept in the wells overnight to remove the background. The assay was performed in quadruplicate; colonies ≥ 20 µm were counted and analyzed using MS Excel software; results were reported as mean ± standard deviation.

### 2.5. Limiting Dilution Analysis (LDA)

Limiting dilution analysis (LDA) has been extensively used to find out differences within multiple groups for a particular trait. In our case, LDA was used for determining the cancer cell initiating frequency of CD133+ and CD133− SKNBE (2) cells; in other words, to evaluate the self-renewing capacity of BTICs. For LDA, following the BTIC isolation procedure, cells were counted so that 10,000 cells/50 µL complete IPM would be present in the first tube. Through serial dilution by factor two up to 1 cell/50 µL, cells were seeded on poly-HEMA coated 96-well plates. For each condition/cell number, samples were seeded in quintuplet. Twenty-five microliters of culture media were added to each well every three–four days, and cells were examined for the presence/absence of spheres and quantified on day 10.

### 2.6. RNA Isolation, cDNA Synthesis, Reverse Transcription Polymerase Chain Reaction (RT-PCR), and Real-Time PCR

PureLink RNA Mini Kit (Life Technologies Ambion, #12183-018) and PicoPure RNA Isolation Kit (Arcturus, #KIT0202) were used for RNA isolation throughout the experiments. In summary, adherent cells grown in cell culture plates (usually 1.5x10^6^ cells/10 cm culture dish) were washed with cold PBS; then, the resuspended cells were centrifuged at 300× *g* for 5 min at 4 °C. An amount of 0.3–0.6 mL of lysis solution with beta-mercaptoethanol was added onto the cells depending on the number of cells, and they were mechanically burst and homogenized by triturating through an insulin syringe 15 times. The cells were centrifuged at 2000× *g* for five minutes at 4 °C, followed by the addition of 70 percent ethanol equal to the volume of the present cell lysate. The lysates were transferred to the filter cartridges and were centrifuged at 12,000× *g* for 30 s. This step was repeated until the whole sample was finished, and the washing process was started. For washing, 700 µL of wash buffer I was added and centrifuged at 12,000× *g* for 15 s. Following the first washing step, 500 µL of wash buffer II was added and repeated twice after centrifugation at 12,000× *g* for 30 s. Tubes were centrifuged for two minutes to dry the membrane. In the elution stage, the cartridges were transferred to new Eppendorf tubes, and depending on the starting number of cells, 20–35 µL of nuclease-free water was put onto the membrane surface and incubated for three minutes at room temperature. Total RNA isolation was completed with centrifugation at 12,000× *g* for one minute. The concentrations of RNA samples obtained were determined with NanoDrop Spectrophotometer (Thermo Fisher Scientific, Paisley, UK), and the samples were stored at −80 °C in the presence of RNase inhibitors or used for further experiments.

Following total RNA isolation, cDNA synthesis was performed using modified MMLV-derived reversible transcriptase using the iScript cDNA Synthesis Kit (BioRad, #1708891, Hercules, CA, USA). For this purpose, a maximum of one µg total RNA sample was diluted to a maximum volume of 15 µL. The RNA sample was denatured at 70 °C for five minutes and centrifuged briefly. After the addition of the 5X reaction buffer and iScript reversible transcriptase, the mix was ready for the cycling. Prepared cDNA samples were diluted with nuclease-free water to the desired concentration immediately before use in qPCR and/or stored at −20 °C for a maximum of one month.

PrimerQuest (Integrated DNA Technologies, IDT, Coralville, IA, USA), a free online software, was used for the qPCR primer design. The mRNA sequences of the target genes were obtained from the NCBI Gene (https://www.ncbi.nlm.nih.gov/gene/, accessed on 20 January 2021) database, the exon regions of the respective genes were determined, and the primers were designed to be at the exon–exon boundary (if possible). Potential primer pairs were evaluated for GC content, melting temperatures (Tm), and the hairpin formation and appropriate primers were determined. The NCBI BLAST database (https://blast.ncbi.nlm.nih.gov/, accessed on 20 January 2021) was used to check the specificity of the designed primers. The designed primers are listed in Table 1.

All the qPCR experiments were performed using SSOAdvanced Universal SYBR Green Supermix (Biorad, #1725274, Hercules, CA, USA) and Applied Bioscience StepOne Plus Real-Time PCR System (Thermo Fisher Scientific, Waltham, MA, USA); essentially, 1–10 ng cDNA was used as template; primers were used at 300 nM each, and the reaction was carried out at 60 °C for 40 cycles. The differences between the expression of target genes were normalized by the expressions of *β-actin* and *gapdh* genes. Each setup was prepared in triplicate and analyzed by the ΔΔCT method as described previously [20]. The fold changes in target gene expressions were calculated based on the mean of the reference gene expression and logarithm-transformed. All qPCR experiments were repeated at least 3 times, unless otherwise noted. The mean and standard deviation values were calculated for each group, and the differences in the gene expression levels were determined considering the control group. For statistical analysis, depending on the context, one-way ANOVA with Tukey post hoc test or Student’s t-test depending on the context with Prism 5 GraphPad software was used. *p*-value under 0.05 was considered statistically significant.

Total RNA was extracted using a Norgen Total RNA isolation kit and quantified using the NanoDrop Spectrophotometer ND-1000. Complementary DNA was synthesized from 0.5–1 µg RNA by using qScript cDNA Super Mix (Quanta Biosciences, Beverly, MA, USA) and a C1000 Thermo Cycler (Bio-Rad, Hercules, CA, USA) with the following cycle parameters: 4 min at 25 °C, 30 min at 42 °C, 5 min at 85 °C, hold at 4 °C. qRT-PCR was performed by using Perfecta SybrGreen (Quanta Biosciences, Waltham, MA, USA) and an Opticon Chroma4 instrument (Bio-Rad, Hercules, CA, USA). Gene expression was quantified by using Opticon software, and expression levels were normalized to 28srRNA expression. For statistical analysis, multiple Student’s t-tests with Prism 5 GraphPad software were used. *p*-value under 0.05 was considered statistically significant.

### 2.7. Microarray and Data Analysis

For microarray analysis, SH-SY5Y cells were transfected with Elk-1-VP16 expression plasmid or empty pCDNA3 plasmid as described above, and 48 h after transfection, RNA samples were isolated using Ambion Tri-pure RNA isolation kit, checked for quality, converted to cDNA, and confirmed for Elk-1 expression as described above. Thereafter, RNA was converted to cDNA using the Superscript Double-Stranded cDNA Synthesis (Invitrogen, Carlsbad, CA, USA) Kit and labeled with NimbleGen One Color DNA Labeling (NimbleGen, Roche, Madison, WI, USA). The labeled cDNA was hybridized to NimbleGen Human Gene Expression Array 12x135K (NimbleGen, Roche, Wisconsin, USA), which covers 45.033 genes with 3 probes per gene, containing 12 arrays per slide. After hybridization, slides were scanned using Genepix 4000B scanner and analyzed with NimbleScan 2.5 software using three arrays from the pCDNA3-transfected cell as reference samples. The expression datasets were normalized using the Robust Multi-Array Average expression measure [21], and differentially expressed genes (DEGs) and their fold-changes were identified from the normalized expression values using two-tailed Student’s t-test assuming equal variances and Benjamini-Hochberg’s method as the multiple testing option to control the false discovery rate. An adjusted *p*-value threshold of 0.15 was used to determine the statistical significance of differential expression. The dataset is accessible from EBI ArrayExpress, with the accession number of E-MTAB-9938.

Gene IDs were converted to official gene symbol, and gene set enrichment analyses of DEGs were performed through ConsensusPathDb (r.32) [22] using KEGG [23], Reactome [24], and Biocarta [25] as the data source for molecular pathways, and Gene Ontology Biological Process annotations [26] as the data source for biological processes. Whole-genome annotation for the human genome was used as the background reference set. *p*-values were determined through a modified Fisher exact test and adjusted via Benjamini-Hochberg’s method. A threshold of adjusted *p*-value < 0.05 was used to determine the statistical significance of the enrichment results. Besides, to characterize the molecular functions of each gene product, and their association with diseases, we manually searched GeneCards Human Gene Database [27].

### 2.8. Promoter Clonings and Site-Directed Mutagenesis

To identify the putative Elk-1 transcription factor binding sites in selected stemness gene promoters (*SOX2*, *NANOG*, *POU5F1*), the Cold Spring Harbor Laboratory—Transcriptional Regulatory Element Database (TRED), Swiss Institute of Bioinformatics—The Eukaryotic Promoter Database (EPD), and Alggen-Promo algorithmic analysis program were used. The promoter sequences that correspond to the genes of interest were retrieved from either the Transcriptional Regulatory Element Database (TRED) (http://rulai.cshl.edu/cgi-bin/TRED/tred.cgi?process=home, accessed on 20 January 2021), or the Eukaryotic Promoter Database (EPD) (http://epd.vital-it.ch/, accessed on 20 January 2021). The obtained promoter sequences were analyzed with Promo 3.0 (http://alggen.lsi.upc.es/cgi-bin/promo_v3/promo/promoinit.cgi?dirDB=TF_8.3, accessed on 20 January 2021). The promoter binding regions for transcription factors can be analyzed by the Promo 3.0 tool, and the results are displayed as “dissimilarity rate”. The dissimilarity matrix expresses the similarity pair to pair between Elk-1 DNA binding sequence and the putative sequences at analyzed genes. From this point of view, the smaller dissimilarity rates are the indicators of a higher possibility for the interaction between Elk-1 and the promoter of interest. The binding ability of Elk-1 to the predicted sites on the promoters could be confirmed by luciferase and chromatin immunoprecipitation assays, thereby verifying the microarray results (Table 2).

Cloning primers for human *SOX2*, *NANOG*, and *POU5F1* promoters were designed and analyzed with NetPrimer (http://www.premierbiosoft.com/netprimer/, accessed on 20 January 2021) and PrimerBlast (http://www.ncbi.nlm.nih.gov/tools/primer-blast/, accessed on 20 January 2021) softwares. The designed cloning primers are listed in Table 3. Gradient PCR with five different annealing temperatures was performed to detect the optimum annealing temperature of the primers. The PCR reactions were prepared with i-Taq DNA Polymerase (Intron, #25024, Seoul, Korea) kit using the genomic DNA isolated from the SH-SY5Y cell line as a template. After optimization, the preparation of the insert was carried out using Pfu DNA Polymerase (Thermo Scientific, #EP0571, Waltham, MA, USA) suitable annealing temperatures as indicated in text for 30 cycles. Following amplification, PCR products were purified by PureLink PCR Purification Kit (Invitrogen, # K3100-01) and cloned into pGL3luciferase reporter plasmid.

Intentional deletion mutations were made on cloned promoter sequences with site-directed mutagenesis (SDM). The promoter sequences were analyzed with Promo 3.0, as stated previously. Potential Elk-1 binding sites on stemness promoters were chosen according to the dissimilarity rate Promo 3.0. Accordingly, *ets1* motif on *NANOG* promoter, *ets1* and *ets2* motifs on *SOX2* promoter, and *ets1*, *ets2*, and *ets3* motifs on *POU5F1* promoter were deleted in corresponding pGL3 luciferase reporter constructs. SDM primers were designed using the NEB Base Changer (http://nebasechanger.neb.com/, accessed on 20 January 2021) website, and Q5^®^ Site-Directed Mutagenesis Kit Protocol (NEB, #E0554) was followed for the mutations. The primer pairs designed flanking the region to be deleted and eventually forming deletion mutants from the cloned promoter sequences are given in Table 4. The mutagenesis was carried out according to the manufacturer’s instructions.

### 2.9. Luciferase Reporter Assay

For each cell line, the necessary optimization experiments were performed, and cell numbers and DNA: PEI ratios were determined for co-transfections. For 24-well cell culture plates for luciferase analysis, for SK-N-BE (2), T98G, and A172 cells 80 × 10^4^ cells/well, and for SH-SY5Y and U87-MG cells, 60 × 10^4^ cells/well were seeded with triplicates for each transfection group. The following day, *SOX2*-luc, *NANOG*-luc, *POU5F1*-luc, or one of the deletion mutant of these plasmids, one of the Elk-1 series plasmids (pCDNA3.1, Elk1-VP16, Elk1-EN, siElk1, or scrRNA), Renilla-luc plasmid (pRL-TK (Promega, #E2241, Madison, WI, USA)) and the proper ratio of PEI mixture was prepared. After transfection, the cells were incubated for 42 h in a normoxic medium and subjected to one percent hypoxia for the last six hours for the normoxia–hypoxia experiments. At the end of hypoxia treatment, luciferase analysis was performed with Thermo Luminoskan Ascent device by using Dual-Glo Luciferase kit (Promega, Wisconsin, USA) with some modifications. For luciferase analysis of monolayer cell lines, 48 h of incubation was necessary before performing luciferase analysis.

On the day of the luciferase assay, the medium on the cells was aspirated, and the wells were washed with PBS. Cells were lysed with 100 µL of 5X Passive Lysis Buffer (PLB) (Promega, #E1941, Wisconsin, USA) diluted to 1X. Seventy-five microliters of the cells were transferred to luminometer compatible white-bottomed 96-well plates. To measure the Firefly luciferase activity, 75 µL of Dual-Glo^®^ Luciferase Reagent was added onto the lysed cells. For at least 15 min, the plates were incubated at room temperature, and the luminescence for Firefly luciferase activity was measured. To measure the Renilla luciferase activity, 75 µL of Dual-Glo^®^ Stop&Glo Luciferase Reagent was added to the wells. They were incubated at room temperature for the equal time that was done for Firefly luciferase, and the luminescence for Renilla luciferase activity was measured. Firefly/Renilla ratios were calculated, normalizations were done, and the results were graphed as relative luciferase activity. For statistical analysis, one-way ANOVA with Tukey post hoc test or Student’s t-test depending on the context with Prism 5 GraphPad software was used. *p*-value under 0.05 was considered significant.

### 2.10. Chromatin Immunoprecipitation (ChIP) Assay

In this assay, proteins and interacting DNA are crosslinked with formaldehyde; the chromatin is sheared with either sonication mechanically or micrococcal nuclease enzymatically. The nucleoprotein complex is enriched by immunoprecipitation, and through the reversal of the crosslinking, DNA and the interacting protein are separated. In the end, the interacting DNA fragment is purified and quantified with ChIP-qPCR. To determine the promoter fragment to be amplified in ChIP PCR, Promo3.0 analysis used for predicting *ets* motifs and their dissimilarity scores was used (Supplementary Table S3). The amplicon size was arranged between 75–150 bp; CpG islands were checked for the potential binding sites of Elk-1, and the position of Elk-1 to those sequences was considered for primer design. The UCSC in silico PCR tool was used to verify the amplicon (https://genome.ucsc.edu/cgi-bin/hgPcr, accessed on 20 January 2021); primers used for ChIP PCR are listed in Table 5.

Essentially, cells were seeded in three separate 150 mm cell culture dishes of 2 × 10^6^ cells/dish per experimental group on day zero. On day 1, cells were transfected with either an empty pCDNA3.1 plasmid or an expression plasmid for Elk1-VP16 plasmids and incubated 48 h at 37 °C, 5% CO_2_. Cells were then treated with 1% formaldehyde at room temperature for 20 min; glycine was then added to the dishes to a final concentration of 0.125 M and incubated for 5 min at room temperature. The dishes were washed three times with cold PBS on ice and then centrifuged at 400× *g* for five minutes at 4 °C with 1× protease inhibitor cocktail (PIC) (Roche, 4693159001). The supernatant was aspirated, and lysis buffer was added onto the cells with a volume of at least 10 times the pellet obtained. The suspension was incubated on ice for 10 min and passed through an insulin needle 20 times. One volume of the sample was mixed with an equal volume of 0.4 percent Trypan Blue Dye, and the cell nuclei were checked under the microscope. The volume of the sonication buffer to be used to dissolve the pellet was adjusted to 2–3 × 10^6^ nuclei/mL and sonicated in the Biorupter UCD-200 Sonicator (Diagenode, Denville, NJ, USA). Following the sonication, cell lysates were centrifuged at 22,000× *g* for 20 min at 4 °C to remove insoluble materials. The supernatant was then diluted five-fold with dilution buffer and pre-cleared for 4 h with slow rotation with protein A/G mixture beads. After incubation, the samples were precipitated at 150× *g* for 5 min at 4 °C, and 10% of the total supernatant was removed as total input control and kept in −20 °C. The rest of the supernatant was divided into two fractions of the negative control (IgG-mock) and immunoprecipitation (IP) per group.

Sixty microliters of ANTI-FLAG^®^ M2 Affinity Gel (Sigma Aldrich, #A2220, Taufkirchen, Germany) resin per group were washed and equilibrated with five volumes of dilution buffer and centrifuged three times at 400× *g* for one minute each at 4 °C. The negative control and IP fractions separated from the dilution in the previous step were mixed with Protein G-Plus agarose beads and anti-Flag M2 resin, respectively. The tubes were incubated at 4 °C overnight with slow rotation. The following day, the mix was centrifuged at 4 °C and 600× *g* for five minutes, and the pellet was collected. The beads were washed with one mL of low salt, high salt, LiCl, and TE buffers at 4 °C with rotation, respectively. Following each of the washing steps, the beads were centrifuged at 4 °C and 600× *g* for five minutes.

At the elution step, the inputs that were collected and frozen a day before were thawed and added as the third fraction of each group. After the last wash, 250 μL fresh elution buffer, pre-heated at 65 °C, was added onto the beads, and they were incubated on a shaker for 15 min. The tubes were vortexed with five-minute intervals and then centrifuged at 4 °C and 18,000× *g* for five minutes. The supernatant was collected for each fraction of each group, and the elution step was repeated with another 250 μL elution buffer. After elution of the crosslinked DNA–protein complex, 10 μL of RnaseA (10 mg/mL) (Intron, #BR003) and 25 μL of 5 M NaCl was added onto the elutes and incubated for at least five hours or overnight at 65 °C. The following day, 10 μL of 0.5 M EDTA, 20 μL 1 M Tris–HCl (pH 6.5), and two μL Proteinase K (20 mg/mL) (Invitrogen, #25530049, Carlsbad, CA, USA) mix were added and incubated again at 65 °C for two more hours. Using MEGAquick-spin™ Plus Total Fragment DNA Purification Kit (Intron Bio, #17290, Sungnam, Korea), the DNA was cleaned up. The resulting fractions were used for qPCR analysis.

SSOAdvanced Universal SYBR Green Supermix (Bio-Rad, #1725274, Hercules, CA, USA) and Applied Biosciences StepOne Plus Real-Time System were used for qPCR analysis with DNA isolated from ChIP. Ten microliters of PCR reaction were prepared by mixing 2X SSO Advanced Universal SYBR Green Supermix, 300 nM forward and reverse primers each, and 1 µL template. In the analysis phase, qPCR signals obtained from the ChIP samples were normalized by the signals obtained from the input, and the mock samples and the results are presented as fold change. For statistical analysis, one-way ANOVA with Tukey post hoc test or Student’s t-test depending on the context with Prism 5 GraphPad software was used. *p* value under 0.05 was considered significant.

## 3. Results

### 3.1. Microarray Analyses Reveal Novel Targets in Elk-1-VP16 Overexpressing SH-SY5Y Cells

**Figure 1:**
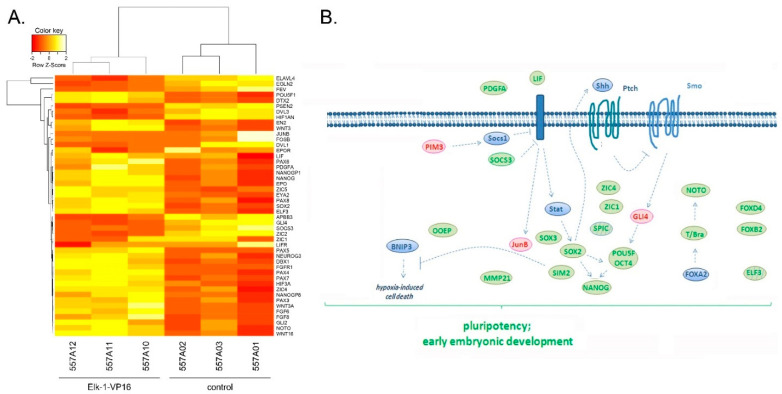
(**A**) Heatmap of a subset of genes regulated by Elk-1-VP16 showing increased (green) or decreased (red) expression in Elk1-VP16 overexpressing SH-SY5Y neuroblastoma cells; 557A10, 557A11, 557A12 correspond to SH-SY5Y cells transfected with Elk-1-VP16 expression plasmid, 557A01,557A02,557A03 control SH-SY5Y cells transfected with empty plasmid; color key shows up- and downregulation levels. (**B**) Schematic representation of the relation between selected genes in pluripotency and early embryonic development pathways that were found to be regulated by Elk-1-VP16 in microarray analysis.

Elk-1 is a ubiquitous transcription factor, yet it has been implicated in different biological processes in the nervous system. In order to identify novel target genes of Elk-1 with respect to survival in neurons, we have overexpressed Elk-1-VP16 constitutively active fusion protein in SH-SY5Y neuroblastoma cells. The comparative analysis of the transcriptome profiles indicated 11,018 differentially expressed genes (DEGs), of which 4212 were downregulated and 6806 were upregulated, when SH-SY5Y neuroblastoma cells were transfected with Elk1-VP16. The gene set enrichment analysis (GSEA) of these genes up- or downregulated by exogenous Elk-1-VP16 presented overrepresentation of quite a high number of biological processes such as anatomical structure development, cell proliferation, single-organism developmental process, developmental growth, and organ and tissue development, including forebrain and midbrain development (Supplement Tables S1 and S2). When a subset of these genes was analyzed further, stemness genes such as *POU5F1*, *SOX2*, and *NANOG*, as well as growth factors and receptors or transcription factors including FGFR1, WNT16, WNT 3, PDGFA, PAX6, PAX7, HIF3A, NOTO, among many others were found to be upregulated, whereas genes such as EGLN2, FEV, JUNB, and GLI4 were found to be downregulated upon overexpression of Elk-1-VP16 (Figure 1A,B).

Prediction of putative Elk-1 binding sites (i.e., ets motifs) on the promoters of these genes was assessed via Alggen PROMO 3.0 online software [28]. Among the genes of interest for which human promoter sequences were available, the analysis was performed for human ELK-1 (TRANSFAC database accession no. T00250) binding, thereby limiting the number of promoters investigated, and out of these, promoters with at least one motif are listed (Supplement Table S3). Among the selected subset of genes, *SOX2* promoter was found to contain one ets motif with a dissimilarity score of 2.16, *NANOG* was found to contain one ets motif with a dissimilarity score of 2.3, and *POU5F1* contained one ets motif with a dissimilarity score of 3.12, among other potential ets binding sites, indicating a high probability of binding (Supplement Table S3). Other promoters of the microarray-determined set of putative Elk-1 target genes, whose promoters contained low dissimilarity score ets motifs, included transcription factors such as *RXRB*, *TCF7L1*, *MEF2B*, *PAX6*, *SOX10*, *CREB3*, *SMAD6*, *CREM*, and *HES7* and signal transduction pathway elements such as *RHO*, *IRAK3*, *WNT3A*, *LIFR*, *FRZB*, *NGFR*, *MAPK6*, *NOTCH4*, *FGF11*, and *NODAL*, among many others (Supplement Table S3).

### 3.2. Regulation of Nervous System Development Related Genes by Elk-1

**Figure 2:**
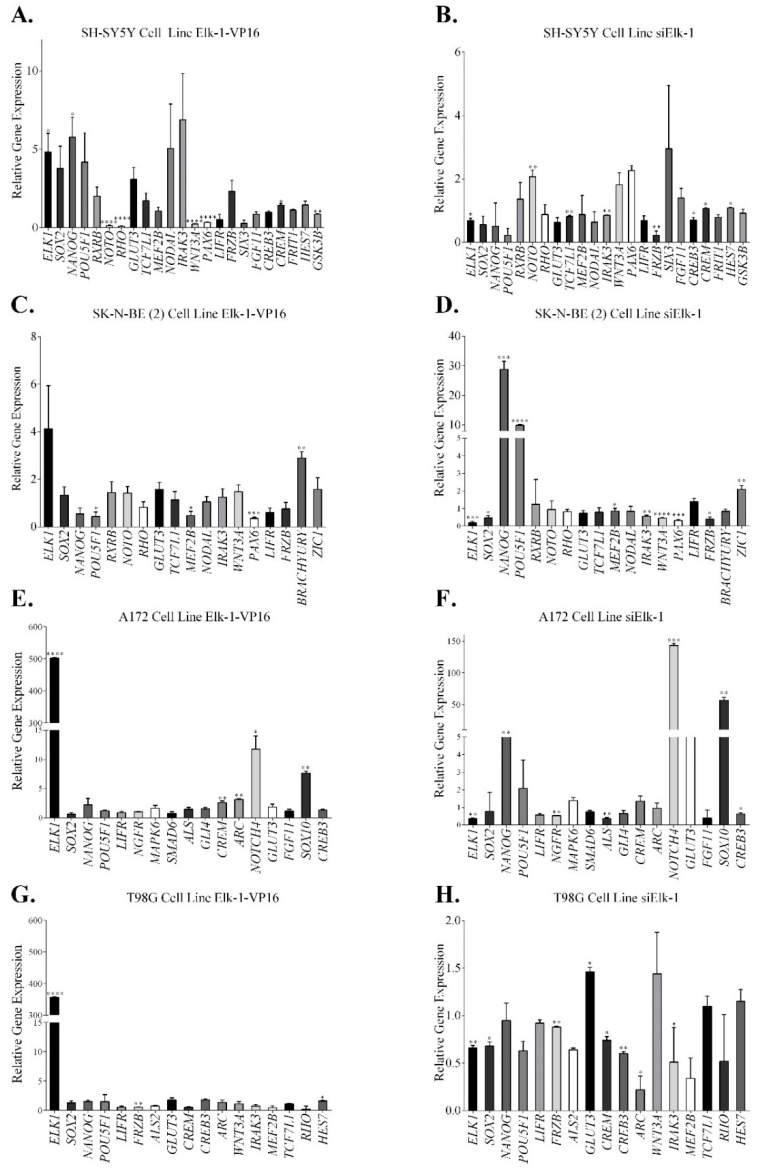
qPCR expression profiles of selected genes in different cell lines upon overexpression of Elk-1-VP16 (**A**,**C**,**E**,**G**) or knockdown of endogenous Elk-1 (**B**,**D**,**F**,**H**). Expression profiles after (**A**). over-expression with Elk1-VP16 and (**B**). after knock-down with siElk1 in SH-SY5Y neuroblastoma cell line; expression profiles after (**C**). over-expression with Elk1-VP16 and (**D**). after knock-down with siElk1 in SK-N-BE(2) neuroblastoma cell line; expression profiles after (**E**). over-expression with Elk1-VP16 and (**F**). after knock-down with siElk1 in A172 GBM cell line; expression profiles after (**G**). over-expression with Elk1-VP16 and (**H**). after knock-down with siElk1 in T98G GBM cell line. Unpaired t-test; **** *p* < 0.0001, *** *p* < 0.001, ** *p* < 0.01, * *p* < 0.05.

To validate regulation of selected candidate genes identified through microarray experiments by Elk-1 transcription factor, we have either overexpressed Elk-1-VP16 constitutively active fusion protein or knocked down endogenous Elk-1 expression in SH-SY5Y and SK-N-BE (2) neuroblastoma cell lines and A172 and T98G GBM cell lines (Figure 2).

qPCR results in SH-SY5Y cells were parallel to those obtained from the microarray analysis, especially in the genes related to pluripotency such as SOX2, NANOG, POU5F1, RXRB, GLUT3, TCF7L1, NODAL, and CREB3 (Figure 2A,B). SOX2 was upregulated in SH-SY5Y overexpressing Elk-1-VP16 protein, similar to microarray, but not in other cell types, while it was repressed when Elk-1 was knocked down (siElk-1) in all cell types (Figure 2). Similarly, NANOG and POU5F1 was upregulated in SH-SY5Y cell overexpressing Elk-1-VP16, but downregulated in cells transfected with siElk-1 plasmid (Figure 2A,B), whereas both genes were repressed in SK-N-BE (2) cells overexpressing Elk-1-VP16 and upregulated in siElk-1 knockdown (Figure 2C,D; Table 6), indicating a cell context-dependent regulation. TCF7L1 and NODAL expression increased in Elk-1-VP16 overexpressing SH-SY5Y and SK-N-BE (2) but decreased in siElk-1 silencing; BRACHYURY (T) expression was upregulated in Elk-1-VP16 overexpressing but decreased in siElk-1 silenced SK-N-BE (2) cells (Figure 2; Table 6). GLUT3 expression was upregulated in all cell types overexpressing Elk-1-VP16, and decreased in all cells with siElk-1 silencing, paralleling the microarray results (Figure 2, Table 6). The expression of ARC and CREB3 increased in A172 and T98G cells overexpressing Elk-1-VP16 but decreased in siElk-1 knockdown cells (Figure 2E–H; Table 6). GLI4 and ALS genes increased in A172 cells overexpressing Elk-1-VP16 and decreased with siElk-1 silencing (Figure 2E,F).

**Figure 3:**
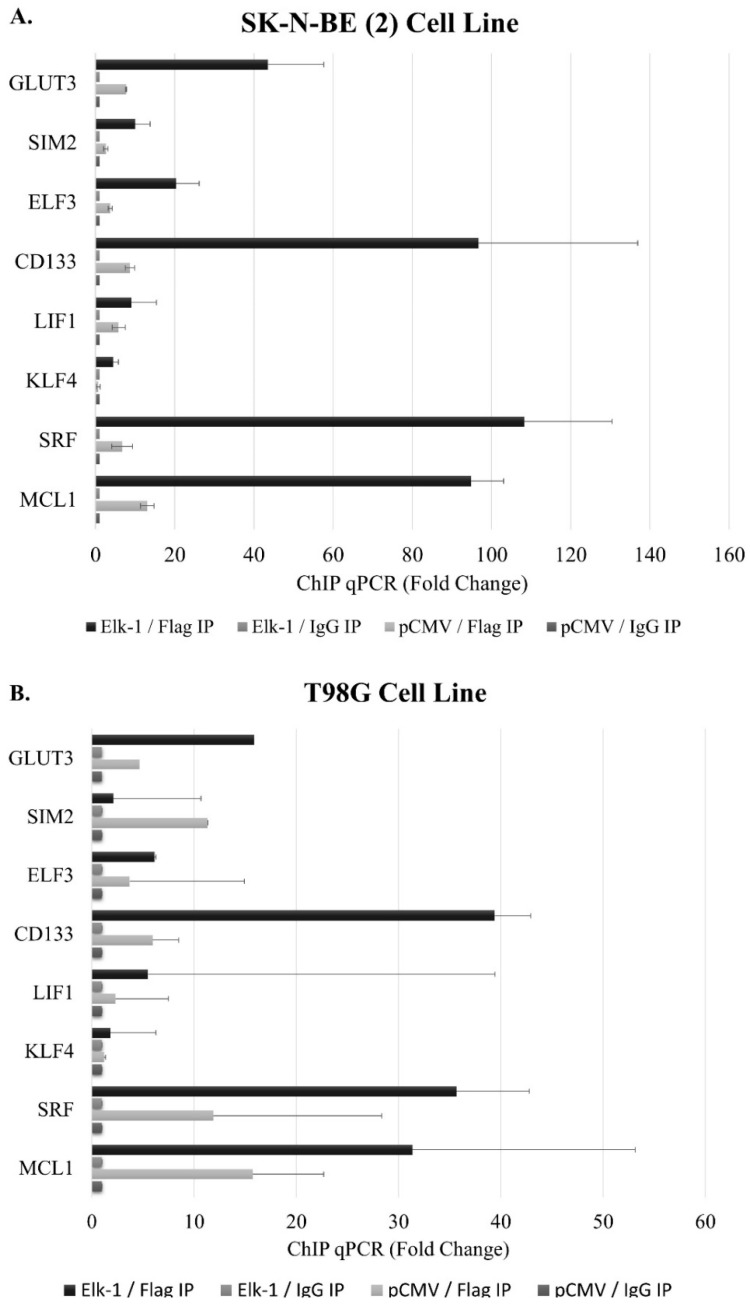
Chromatin immunoprecipitation assay for the identification of Elk-1 binding sites on the target gene promoters in pCMV-transfected (pCMV) vs. Elk-1 over-expressing cells (Elk-1) in (**A**). SK-N-BE (2) cells and (**B**). T98G cells. Lysates were immunoprecipitated with either Flag antibody (Flag IP) for exogenous Elk-1 or IgG (IgG IP) as control. qPCR results were analyzed as explained in Materials and Methods and reported as average fold change.

The promoters of a subset of genes have been selected for chromatin immunoprecipitation to address whether predicted binding sites were indeed binding to Elk-1 (Figure 3). To that end, we have transfected SK-N-BE (2) neuroblastoma and T98G GBM cell lines with Elk-1-Flag expression vector and pulled down exogenous Elk-1 using Flag-agarose beads. The known targets *SRF* (*p* = 0.0451) and *MCL1* (*p* = 0.0102) showed significant binding in SK-N-BE (2) cells, but the binding was not statistically significant in T98G cells (Figure 3A). Among the novel promoters identified in this study, *GLUT3* promoter showed Elk-1 binding in both cell types, albeit not to the same extent, while *KLF4* (*p* = 0.0496) only showed significant binding in T98G cells (Figure 3B). LIF1, however, did not show significant Elk-1 binding in either cell type.

### 3.3. Regulation of SOX2, NANOG, and POU5F1 by Elk-1 in CD133+ Cells

**Figure 4:**
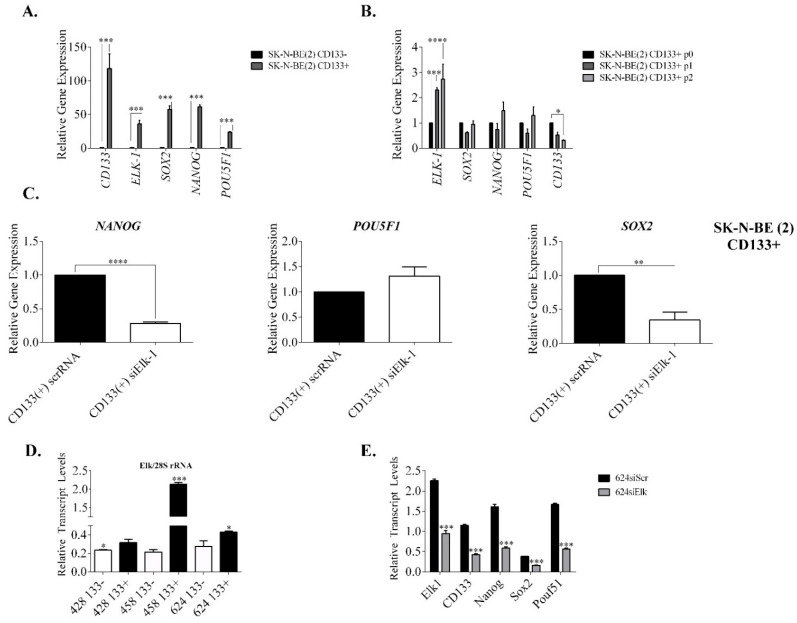
qPCR expression profiles of stemness genes in CD133− vs. CD133+ SK-N-BE(2) cells and in primary brain tumors. (**A**). Stemness gene expression analysis of SKNBE(2) passage 0, passage 1, and passage 2 cells (*** *p* < 0.001, two-way ANOVA w/Dunnett multiple comparison test); (**B**). Stemness gene expression analysis of SKNBE(2) CD133+ BTICs vs. CD133− spheroids (unpaired t-test, * *p* < 0.05, *** *p* < 0.001, **** *p* < 0.0001); (**C**). *left*, *NANOG*, *middle POU5F1* and *right*, *SOX2* gene expressions in CD133+ cells upon silencing of Elk-1 expression (unpaired t-test; **** *p* < 0.0001, ** *p* = 0.0051); (**D**). endogenous Elk-1 expression levels in CD133− and CD133+ primary brain tumor samples (sample no 428, 458 and 624); relative gene expression is reported as normalized to 28S rRNA level (unpaired t-tests; * *p* < 0.05, *** *p* < 0.0001); (**E**). primary brain tumor cells from sample no 624 were transfected with either scrRNA or siElk-1 plasmids and analyzed for expression level of endogenous *ELK-1*, *CD133*, *NANOG*, *SOX2*, and *POU5F1* normalized to 28S rRNA level (unpaired t-tests; *** *p* < 0.0001).

Since Elk-1 was previously shown to be important in human embryonic stem cells (hESCs) maintenance of self-renewal capacity through co-occupation of promoters with *ERK2* [29], and to regulate the promoter of CD133, a cell surface protein commonly used as a cancer stem cell marker [15], we addressed whether the cell context-dependent regulation was due to heterogenous nature of some cell lines used in terms of their tumorsphere forming abilities. SK-N-BE (2) neuroblastoma cells were shown to form CD133+ tumorspheres, unlike SH-SY5Y cells, hence we have first sorted CD133− and CD133+ SK-N-BE (2) cells and showed that expression of *CD133*, *ELK*-*1*, *SOX2*, *NANOG*, and *POU5F1* were all significantly more in CD133+ cells than in CD133− cells (Figure 4A). Intriguingly, ELK-1 levels increased in different passages (p1 and p2) of CD133+ sorted cells, while *CD133* levels declined with each passage; *NANOG* and *POU5F1* levels also increased slightly in p2 cells, albeit not significantly (Figure 4B). Both passages (p1 and p2) of CD133+ SK-N-BE (2) cells were shown to be Nestin+ (data not shown). To address whether this coexpression of ELK-1 with stemness genes studied is through direct regulation, we have silenced endogenous Elk-1 expression in CD133+ SK-N-BE (2) cells, and observed that *NANOG* and *SOX2* but not *POU5F1* were downregulated significantly upon silencing (Figure 4C). It must be noted, however, that overexpression of Elk-1-VP16 in CD133− cells did not yield upregulation of *NANOG*, *SOX2* or *POU5F1* in SK-N-BE (2) cells (data not shown).

To investigate whether similar regulation could be observed in primary GBM, primary brain tumor samples from three different patients (patient no. 428, 458, 624) were analyzed for ELK-1 expression in CD133− vs. CD133+ cells. Although there was variability between samples, in all three GBMs, CD133+ cells expressed significantly more ELK-1 than CD133− cells (Figure 4D). This was parallel to our analysis of GBM cell lines, where tumorspheres of A172, T98G, and U87 GBM cells expressed significantly more Elk-1 protein than the monolayer cultures did, whereas ELK-1 expression level did not alter significantly in SH-SY5Y tumorsphere vs. monolayer cultures (data not shown). Furthermore, when endogenous ELK-1 was silenced in the primary tumor culture of patient 624 (middle level of ELK-1 expression), *CD133*, *NANOG*, *SOX2*, and *POU5F1* levels were all downregulated as compared to scramble RNA control (Figure 4E).

### 3.4. Effect of Elk-1 Expression on Colony Formation of SK-N-BE (2) Cells on Soft Agar

The ability of transformed cells to grow in anchorage-free conditions is one of the hallmarks of cancer formation, and soft agar colony assay is a commonly used tool to assay for this feature [30]. It was shown in endometrial tumors, for instance, that CD133+ cells exhibited higher colony formation than CD133− cells in soft agar assay [31]. We have, therefore, addressed whether the same scenario was true for CD133+/CD133− SK-N-BE (2) cells, and whether overexpression of Elk-1-VP16 or silencing of endogenous Elk-1 would affect the number of colonies. To that end, we have sorted SK-N-BE (2) cells into CD133+ BTICs and CD133− cells, and both CD133+ and CD133− spheroids were grown in IPM culture conditions for three days in limiting dilution assay (LDA), and the frequency of spheroid formation was found to be almost tenfold more in CD133+ BTIC cells, indicating that sorting of cells was successful.

Next, the effects of Elk-1 overexpression or silencing were studied; to that end, we have transfected CD133− cells with Elk-1-VP16 expression vector, while CD133+ cells were transfected with siElk-1 silencing vector as described in Materials and Methods, and colony formation frequencies were determined in soft agar assay. In untransfected SK-N-BE (2) cells, CD133− cells and unsorted cells showed a similar number of colonies (24 ± 11 vs. 25 ± 11, respectively), whereas CD133+ BTICs had almost 50% more colonies formed (33 ± 9 colonies). CD133− cells transfected with either pCDNA3-Elk-1-VP16 (37 ± 10 colonies) or pCMV6-Flag-Elk-1-VP16 (50 ± 15 colonies) showed higher colony number than their counterparts transfected with empty vectors, pCDNA3.1 (32 ± 3 colonies) or pCMV-Flag (24 ± 10 colonies). On the other hand, CD133+ cells where endogenous Elk-1 was silenced by RNAi exhibited a decreased colony number (18 ± 5) compared to scrambled RNA control (26 ± 9) (Supplement Table S4).

### 3.5. Regulation of NANOG, POU5F1, and SOX2 Promoters by Elk-1

To assess whether the regulation of these genes by Elk-1 was direct or indirect, the promoters for *NANOG*, *POU5F1*, and *SOX2* were cloned to luciferase reporter vectors and tested for Elk-1 regulation in different cell lines.

**Figure 5:**
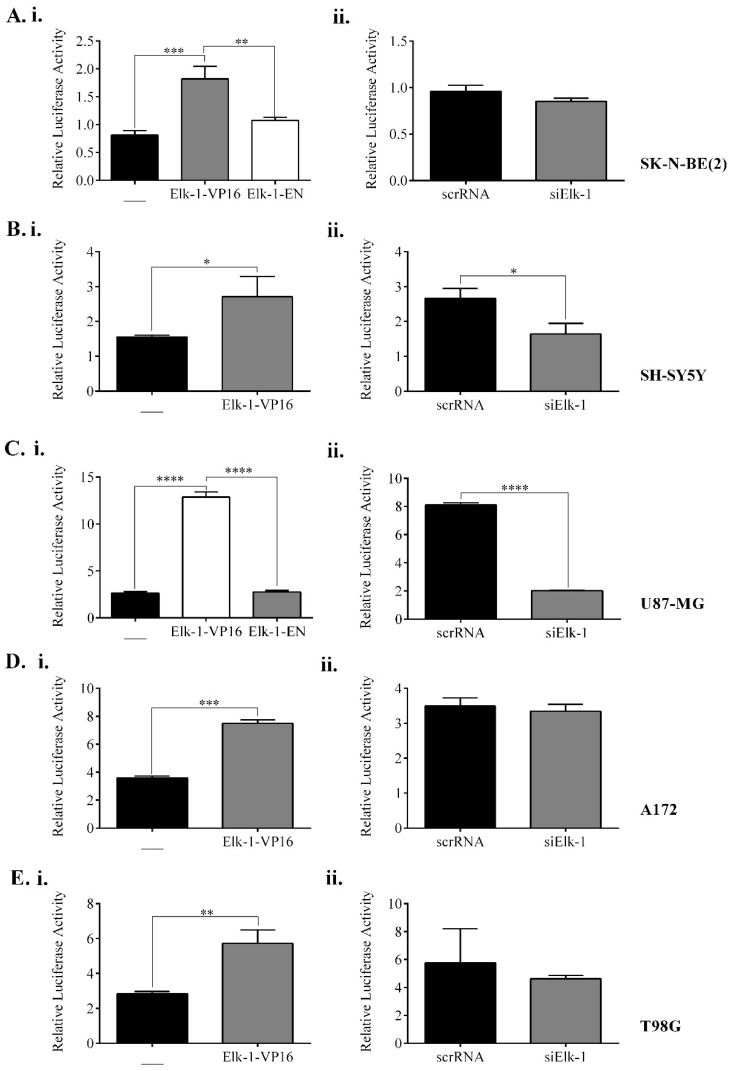
*SOX2* promoter activity analysis with respect to (**i**) Elk-1 variants over-expression and (**ii**) endogenous Elk-1 silencing in (**A**) SK-NBE (2), (**B**) SH-SY5Y, (**C**) U87-MG, (**D**) A172, and (**E**) T98G cell lines. Luminometric measurements were normalized to *Renilla*-luc activity. ANOVA, Tukey’s multiple comparative tests, ** *p* < 0.01, *** *p* < 0.001, **** *p* < 0.0001 for Ai, Ci; unpaired two-tailed t-test, * *p* < 0.5, ** *p* < 0.01, **** *p* < 0.0001 was done for Bi, Bii, Cii, Di, Ei.

Initially, SK-N-BE (2) (Figure 5A) and SH-SY5Y (Figure 5B) neuroblastoma cells and U87-MG (Figure 5C), A172 (Figure 5D), and T98G (Figure 5E) GBM cells were either transfected with constitutively active Elk-1-VP16 and/or dominant-negative Elk-1-EN fusion protein expression vectors for overexpression (i), or with siElk-1 or scrRNA vectors for silencing (ii) experiments to study the regulation of *SOX2* promoter by Elk-1 protein (Figure 5). Although there appear to be cell type-specific variations, *SOX2* promoter appeared to be upregulated upon Elk-1-VP16 overexpression in all cell types (Figure 5Ai–Ei), whereas only SH-SY5Y and U87 cells exhibited downregulation of SOX2-dependent luciferase activity upon the silencing of endogenous Elk-1, indicating that other proteins are involved in the regulation of this promoter (Figure 5Bii,Cii).

**Figure 6:**
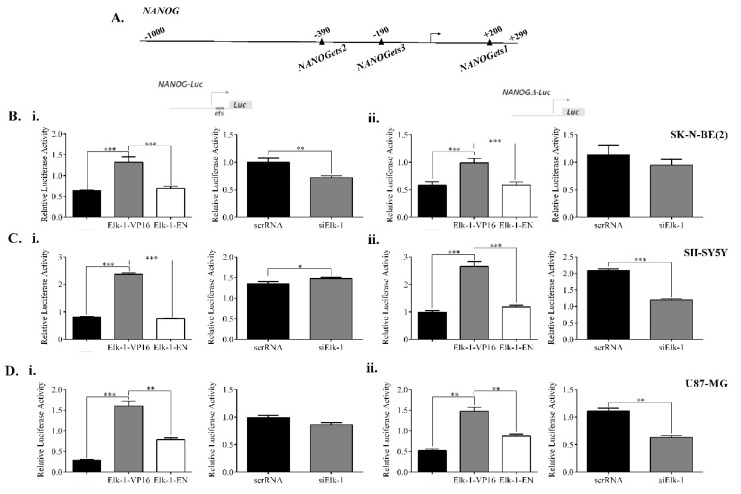
Regulation of *NANOG* promoter by Elk-1. (**A**) Schematic diagram of predicted *ets* motifs *ets1-3* on *NANOG* promoter. *Ets1* was predicted to be a stronger binding motif for Elk-1 and was deleted to generate *NANOG∆-*Luc reporter plasmid. (**B**) Luciferase assay for (**i**) wildtype *NANOG*-Luc and (**ii**) *NANOGΔ*-Luc reporters in SK-N-BE (2) cells after transfection of expression plasmids with Elk1-VP16, Elk1-EN, or empty control plasmid pCDNA3.1 (**left graphs**) or co-transfection of silencing plasmids for scrRNA control or siElk-1 (**right graphs**). Luminometric measurements were normalized to Renilla-luc activity. ANOVA, Tukey’s multiple comparative tests, *** *p* < 0.001 for (**i**) and (**ii**) left graphs; unpaired two-tailed t-test, ** *p* < 0.01 was done for (**i**) and (**ii**) right graphs. (**C**) Luciferase assay for (**i**) wildtype *NANOG*-Luc and (**ii**) *NANOGΔ*-Luc reporters in SH-SY5Y cells after transfection of expression plasmids with Elk1-VP16, Elk1-EN or empty control plasmid pCDNA3.1 (**left graphs**) or co-transfection of silencing plasmids for scrRNA control or siElk-1 (**right graphs**). Luminometric measurements were normalized to *Renilla*-luc activity. ANOVA, Tukey’s multiple comparative tests, *** *p* < 0.001 for (**i**) and (**ii**) left graphs; unpaired two-tailed t-test; * *p* < 0.5, *** *p* < 0.001 for (**i**) and (**ii**) right graphs. (**D**) Luciferase assay for (**i**) wildtype *NANOG*-Luc and (**ii**) *NANOGΔ*-Luc reporters in U87-MG cells after transfection of expression plasmids with Elk1-VP16, Elk1-EN, or empty control plasmid pCDNA3.1 (**left graphs**) or co-transfection of silencing plasmids for scrRNA control or siElk-1 (**right graphs**). Luminometric measurements were normalized to *Renilla*-luc activity. ANOVA, Tukey’s multiple comparative tests, ** *p* < 0.01, *** *p* < 0.001 for (**i**) and (**ii**) left graphs; unpaired *t*-test; ** *p* < 0.01 for (**i**) and (**ii**) right graphs.

We next studied *NANOG* promoter; while *SOX2* promoter was found to have 1 consensus Elk-1 binding motif with dissimilarity score (DS) of less than 1%, and 5 ets motifs with DS 1–10%, *NANOG* promoter was found to contain three consensus ets motifs (Figure 6A), two of which had DS of 1–10% (Table 2). We have constructed a wildtype *NANOG* promoter reporter vector (*NANOG*-Luc), and one where the higher similarity consensus ets motif (ets1) was deleted (*NANOG∆*-Luc), and studied the regulation of this promoter by Elk-1 in different cell lines (Figure 6).

Elk-1-VP16 overexpression in SK-N-BE (2) cells resulted in upregulation from wildtype *NANOG* promoter, but the upregulation was slightly less in *NANOG∆*-Luc reporter; Elk-1-EN repressed both promoter activities to control levels (Figure 6Bi vs. Figure 6Bii). Parallel to this, when Elk-1 was silenced using siElk-1, *NANOG*-Luc reporter activity was decreased (Figure 6Bi), whereas there was no significant change in NANOG*∆*-Luc activity in SK-N-BE (2) cells (Figure 6Bii). On the other hand, there was no significant difference between *NANOG* vs. *NANOG∆* promoter activity by Elk-1-VP16 overexpression in SH-SY5Y or U87 cells, while Elk-1-EN repressed both wildtype and mutant promoter activities (Figure 6C,D). There was a slight albeit significant increase in *NANOG* promoter activity in siElk-1 SH-SY5Y cells, whereas *NANOG∆* promoter activity was decreased upon siElk-1 silencing (Figure 6Ci vs. Figure 6Cii); in U87 silencing, endogenous Elk-1 did not significantly alter wildtype *NANOG*-Luc activity but resulted in a decrease in *NANOG∆*-Luc (Figure 6Di vs. Figure 6Dii). There was no significant change in either Elk-1-VP16 overexpression or siElk-1 silencing in A172 and T98G cells (data not shown).

**Figure 7:**
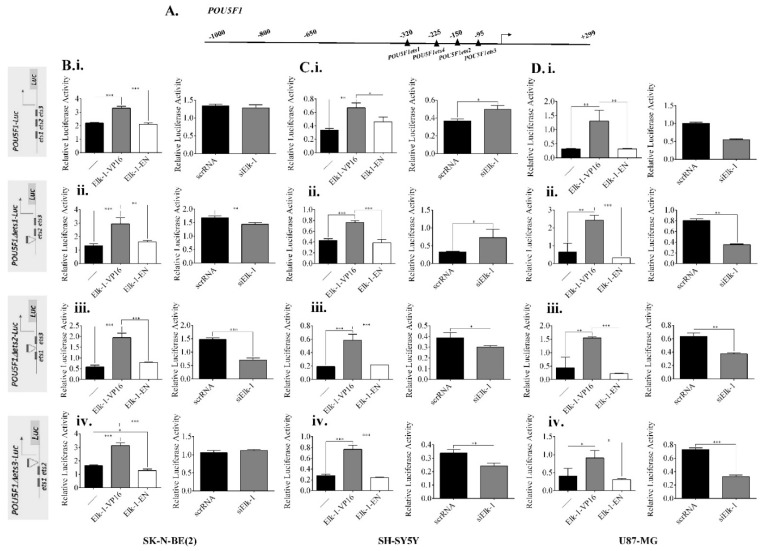
Regulation of *POU5F1* promoter by Elk-1. (**A**) Schematic diagram of predicted *ets* motifs *ets1-4* on *POU5F1* promoter. Motifs e*ts1-3* were predicted to be stronger binding motifs for Elk-1 and were individually deleted to generate *POU5F1*∆e*ts1-*Luc, *POU5F1∆*e*ts2-*Luc, and *POU5F1∆*e*ts3-*Luc reporter plasmids. (**B**) Luciferase assay for (**i**) wildtype *POU5F1-*Luc and its deletion mutant reporters (**ii**) *POU5F1∆*e*ts1-*Luc, (**iii**) *POU5F1∆*e*ts2-*Luc, and (**iv**) *POU5F1∆*e*ts3-*Luc in SK-N-BE (2) cells after transfection of expression plasmids with Elk1-VP16, Elk1-EN, or empty control plasmid pCDNA3.1 (**left graphs**) or co-transfection of silencing plasmids for scrRNA control or siElk-1 (**right graphs**). Luminometric measurements were normalized to Renilla-luc activity. ANOVA, Tukey’s multiple comparative tests, ** *p* < 0.01, *** *p* < 0.001 for left graphs (**i**–**iv**); unpaired two-tailed t-test, ** *p* < 0.01 and *** *p* < 0.001 for right graphs (**i**–**iv**). **C.** Luciferase assay for (**i**) wildtype *POU5F1-*Luc and its deletion mutant reporters (**ii**) *POU5F1*∆e*ts1-*Luc, (**iii**) *POU5F1*∆e*ts2-*Luc, and (**iv**) *POU5F1* e*ts3-*Luc in SH-SY5Y cells after transfection of expression plasmids with Elk1-VP16, Elk1-EN, or empty control plasmid pCDNA3.1 (**left graphs**) or co-transfection of silencing plasmids for scrRNA control or siElk-1 (**right graphs**). Luminometric measurements were normalized to Renilla-luc activity. ANOVA, Tukey’s multiple comparative tests, * *p* < 0.5, ** *p* < 0.01, *** *p* < 0.001 for left graphs (**i**–**iv**); unpaired two-tailed t-test, * *p* < 0.5, ** *p* < 0.01 for right graphs (**i**–**iv**). (**D**) Luciferase assay for (**i**) wildtype *POU5F1-*Luc and its deletion mutant reporters (**ii**) *POU5F1∆*e*ts1-*Luc, (**iii**) *POU5F1∆*e*ts2-*Luc, and (**iv**) *POU5F1∆*e*ts3-*Luc in U87-MG cells after transfection of expression plasmids with Elk1-VP16, Elk1-EN, or empty control plasmid pCDNA3.1 (**left graphs**) or co-transfection of silencing plasmids for scrRNA control or siElk-1 (**right graphs**). Luminometric measurements were normalized to Renilla-luc activity. ANOVA, Tukey’s multiple comparative tests, * *p* < 0.5, ** *p* < 0.01, *** *p* < 0.001 for left graphs (**i**–**iv**); unpaired two-tailed t-test, ** *p* < 0.01, *** *p* < 0.001 for right graphs (**i**–**iv**).

In *POU5F1* promoter, of the four predicted ets motifs, three of them were predicted to have DS score of 1-10% DS (Table 2; Figure 7A). Wildtype *POU5F1* promoter was cloned, and deletion constructs for these motifs (ets1-ets3) were generated for luciferase reporter assays as described in Materials and Methods. When wildtype *POU5F1* promoter activity was compared to deletion constructs in SK-N-BE (2) cells transfected with Elk-1-VP16 expression plasmid, *POU5F1∆ets2*-Luc deletion construct exhibited less upregulation (around 2.4 units) than wildtype, *POU5F1∆ets1*, and *POU5F1∆ets3* promoters (around 3 units), while Elk-1-EN overexpression resulted in similar level of activation to control in all cases (Figure 7B). On the other hand, siElk-1 silencing did not result in a significant change in wildtype *POU5F1* promoter activity or the *POU5F1∆ets3* deletion mutant, whereas it resulted in a decrease in luciferase activity in both *POU5F1∆ets1* and *POU5F1∆ets2* constructs in SK-N-BE (2) cells (Figure 7B).

Elk-1-VP16 overexpression upregulated wildtype *POU5F1*-Luc reporter activity, while Elk-1-EN repressed it in SH-SY5Y cells; there was no significant change in this profile in either of the three ets deletion constructs, indicating the regulation might be through a different motif or could be indirect (Figure 7C). Interestingly, siElk-1 silencing upregulated wildtype *POU5F1*-Luc and *POU5F1∆ets1*-Luc reporter activity, while decreasing *POU5F1∆ets2*-Luc and *POU5F1∆ets3*-Luc reporter activity (Figure 7C). In U87-MG GBM cells, however, wildtype *POU5F1*-Luc and *POU5F1∆ets2*-Luc reporters were upregulated to similar levels in Elk-1-VP16 overexpression (1.2 units in Figure 7Di and 1.5 units in Figure 7Diii), while *POU5F1∆ets1*-Luc was upregulated more (2.4 units, Figure 7Dii), and upregulation was significantly less in *POU5F1∆ets3*-Luc reporter (Figure 7Div). Elk-1-EN overexpression did not significantly alter promoter activity (Figure 7D), and while siElk-1 silencing did not cause any change in wildtype promoter, it resulted in a downregulation in all deletion constructs to a different extent (Figure 7D). Wildtype *POU5F1*-Luc promoter was upregulated by Elk-1-VP16 overexpression in both A172 and T98G cells, although siElk-1 silencing did not significantly change with respect to scrambled RNA control.

### 3.6. Binding of Elk-1 to Predicted ets Motifs on SOX2, NANOG, and POU5F1 Promoters

**Figure 8:**
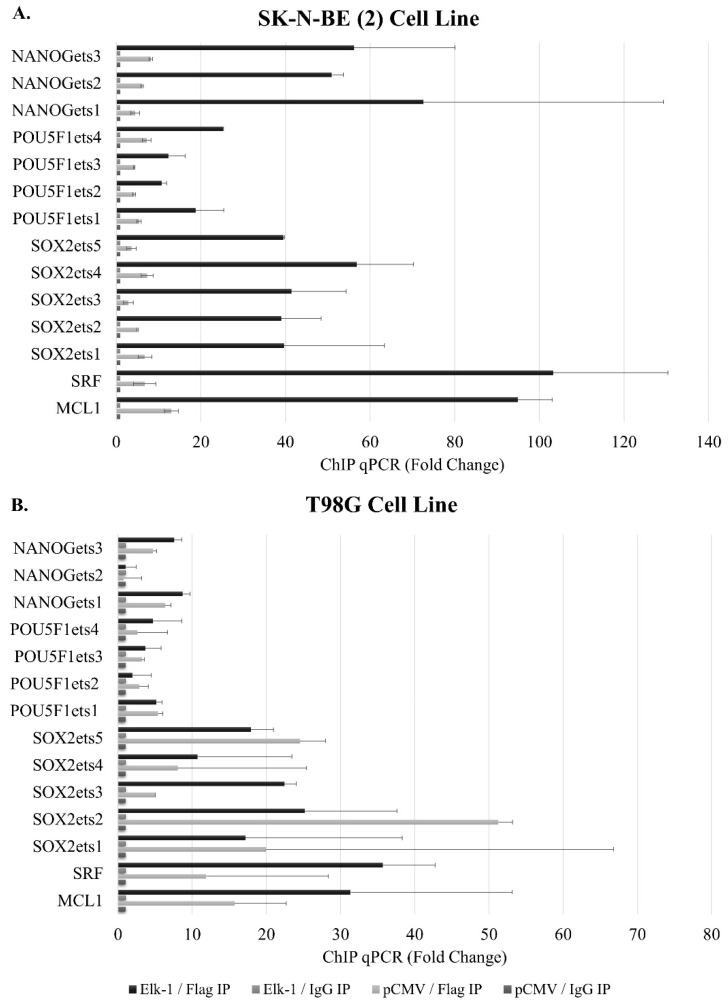
Chromatin immunoprecipitation assay for the identification of Elk-1 binding sites on the target gene promoters in pCMV-transfected (pCMV) vs. Elk-1 over-expressing cells (Elk-1) in (**A**). SK-N-BE (2) cells and (**B**). T98G cells. Lysates were immunoprecipitated with either Flag antibody (Flag IP) for exogenous Elk-1 or IgG (IgG IP) as control. qPCR results were analyzed as explained in Materials and Methods and reported as average fold change. All predicted *ets* motif sequences on *NANOG*, *SOX2*, and *POU5F1* promoters were screened for Elk-1 binding; *SRF* and *MCL1* promoter sequences were used as positive control.

Elk-1-VP16 overexpression was found to upregulate expression of *SOX2*, *NANOG*, and *POU5F1* expression in qPCR analysis, and wildtype promoter luciferase reporters were found to be upregulated by Elk-1-VP16 in a cell context-dependent manner, yet deletion of predicted ets motifs did not significantly change reporter activities, indicating that either there are other ets motifs in distal promoters that are not cloned in this study, or that the regulation is not through direct Elk-1 binding to these predicted ets motifs. To address this second point, we have carried out chromatin immunoprecipitation (ChIP) experiments in SK-N-BE (2) neuroblastoma and T98G GBM cell lines (Figure 8).

The cells were transfected with pCMV-Flag-Elk-1 (empty pCMV was used as control), and immunoprecipitation was carried out using Flag agarose beads (Flag IP); IgG beads were used as control (IgG IP). Elk-1 binding motifs on *SRF* and *MCL*-1 promoters were used as a positive control for Elk-1 binding. All three of the predicted ets motifs on the *NANOG* promoter exhibited Elk-1 binding in SK-N-BE (2) cells (Figure 8A) but not on T98G cells (Figure 8B). Similarly, all four predicted ets motifs on *POU5F1* promoter showed Elk-1 binding, albeit to different extents, in SK-N-BE (2) cells (Figure 8A) but not on T98G cells (Figure 8B). Likewise, all five predicted ets motifs showed Elk-1 binding in SK-N-BE (2) cells (Figure 8A), whereas only the ets3 motif showed significant binding to Elk-1 in T98G (Figure 8B). This indicates that, while Elk-1 is capable of binding to these predicted motifs, this binding is affected by cell-dependent circumstances, which may be a transcriptional partner or posttranslational modification status of the Elk-1 protein in that particular cell type.

## 4. Discussion

ETS transcription factors are involved in a number of biological processes in different tissues, and it was shown that in embryonic development, expression of several ETS proteins including Elf3 and SpiC increased after fertilization until the blastocyst stage, and silencing of ETS expression affected Oct3/4 gene expression [32]. It was shown in human pluripotent stem cells (hPSCs) with different X chromosome inactivation states (Xa, active, Xi, inactive) that Elk-1 overexpression mimicked XaXa in terms of decreased pluripotency, the differences being diminished in low oxygen [33].

One study has shown Elk-1 to be essential for human embryonic stem cells, and that it co-occupies promoters of genes in cell proliferation pathways with *ERK2*, and in the absence of *ERK2*, the promoters were repressed by Polycomb proteins [29]. In fact, Elk-1 was further found to be upregulated, while Nanog, Oct4, and Sox2 were found to be repressed during mesoderm differentiation of hESCs, and it was shown to bind to and activate promoters such as *EGR-1* while repressing a subset of promoters such as *FOSL1* [16]. Intriguingly, mice deficient for Elk-1 were viable albeit with mild neuronal impairment, indicating other Ets proteins may act redundantly and compensate for its embryonic functions [34]. During neuronal differentiation of mES cells, Sox2 chromatin interaction profiles were altered, and promoters of neuronal differentially expressed gene clusters were enriched in Elk-1, among other transcription factors [35]. Similarly, during reprogramming of fibroblasts into neural stem cells (NSCs) using pharmacological molecules, Elk-1 was found to be one of the transcription factors to regulate reprogramming, particularly through binding Sox2 promoter [36].

Another Ets protein, Pea3/ETV4, was shown to regulate Nanog and Oct4 expression in pluripotent NCCIT embryonic carcinoma cells [37,38]. Interestingly, members of the Pea3 subfamily of ETS proteins, ETV4 and ETV5, were found to be expressed in undifferentiated ES cells, and suppression of Oct3/4 was found to result in downregulation of their expression, and ETV4 and ETV5 were found to be important for proliferation of undifferentiated ES cells through regulation of stem cell-related genes such as Tcf15, Gbx2, and Zic3 [39]. A transcriptional partner of Elk-1, namely serum response factor (SRF), was shown to repress the reprogramming induced by ERK pathway inhibition, and to negatively regulate pluripotency [40], which may be independent of Elk-1 interaction.

CD133 is a cell surface protein that has been used alone [12] or in combination with CD15 [41] to isolate and culture brain-tumor-initiating cells from a variety of tumors. ERK/MAPK pathway was shown to be required for CD133 expression [42], and HIF-1α was shown to bind to the CD133 promoter through Elk-1 [15], which is supported in our study by overexpression of Elk-1 in CD133+ BTIC subpopulation.

In a genome-wide study in the human embryonic stem cell (hESC) population, ELK1 was found to be essential for hESCs, and some of the promoters bound by ELK1 were determined to be important in the maintenance of embryonic identity, spinal cord development, and neuron fate development [29]. Furthermore, induced neural stem cells were found to contain relatively high levels of phosphorylated Elk-1, along with Gli2, and both were shown to bind to *Sox2* promoter upon neural reprogramming [36], and distinct GABPA/Elk-1 motifs were found in Sox2 promoter, identified as a neuronal cluster gene involved in differentiation of embryonic stem cells to neuronal precursors [35]. It is intriguing whether tumorigenesis reactivates this mechanism in a cell context-dependent manner.

## 5. Conclusions

We propose that not only does ELK1 present a novel target for tumor therapy directed at eliminating BTIC population, but also can be used as a molecular diagnostic molecule to identify potential for tumor recurrence. It should be noted, however, that posttranslational modifications such as phosphorylation and SUMOylation regulate ELK1 protein, which can differ among gliomas and must be studied in more detail.

## Figures and Tables

**Table 1:** qPCR primers used.

Gene	Site	Sequence (5′-3′)
*GAPDH*	Frw	CAT CTT CCA GGA GCG AGA TCC
	Rev	AAA TGA GCC CCA GCC TTC TCC
*ACTB*	Frw	ACG AAA CTA CCT TCA ACT CC
	Rev	GAT CTT GAT CTT CAT TGT GCT GG
*ELK1*	Frw	GCT TCC TAC GCA TAC ATT GAC C
	Rev	ACT GGA TGG AAA CTG GAA GG
*SOX2*	Frw	GGG AAA TGG GAG GGG TGC AAA AGA GG
	Rev	TTG CGT GAG TGT GGA TGG GAT TGG TG
*POU5F1*	Frw	AAG GAT GTG GTC CGA GTG TGG
	Rev	CCT GAG AAA GGA GAC CCA GCA G
*NANOG*	Frw	TTC AGA GAC AGA AAT ACC TCA GCC
	Rev	CCT TCT GCG TCA CAC CAT TGC
*WNT3A*	Frw	GACAAAGCTACCAGGGAGTC
	Rev	CTGCTGCAGCCACAGAT
*IRAK3*	Frw	ACATACTAGAGTTGGCTGCATATT
	Rev	TGTCACCTACACACTGCAATC
*MEF2B*	Frw	CAACCGCCTCTTCCAGTATG
	Rev	TCAGCGTCTCGAGGATGT
*TCF7L1*	Frw	TGAGCGTGAAATCACCAGTC
	Rev	TGGCCCTCATCTCCTTCATA
*RHO*	Frw	CATGATGAACAAGCAGTTCCG
	Rev	AGAGTCCTAGGCAGGTCTTAG
*HES7*	Frw	CGGGATCGAGCTGAGAATAG
	Rev	GTTCCGGAGGTTCTGGTC
*NOTO*	Frw	GCTGGAAGAGTTGGAGAAAGT
	Rev	ACTCTCACCTGGTTCTCTGTA
*SIX3*	Frw	CAGCAAGAAACGCGAACTG
	Rev	GTGCTGGAGCCTGTTCTT
*CREB3*	Frw	ACCTGCATCTTGGTCCTACTA
	Rev	GGACAACACTCCATGCTCAG
*CREM*	Frw	ATCCCAGCATGATGGAAGTATAA
	Rev	ATTGCTGCTACCTGAGCTAAA
*LIFR*	Frw	GCTCTGGACAAGTTAAATCCATAC
	Rev	CCCTTTGAAGGACTGGCT
*FRZB*	Frw	AAGTTAAGCGCTGGGATATGA
	Rev	GGGATTTAGTTGCGTGCTTG
*GLUT3*	Frw	AGCTCTCTGGGATCAATGCTGTGT
	Rev	ATGGTGGCATAGATGGGCTCTTGA
*RXRB*	Frw	GATGTGAAGCCACCAGTCTTAG
	Rev	GTAGTGTTTGCCTGAGCTTCT
*NODAL*	Frw	TACATCCAGAGTCTGCTGAAAC
	Rev	CTAGGAGCACTCTGCCATTATC
*PAX6*	Frw	GTGAATGGGCGGAGTTATGA
	Rev	ATGAGTCCTGTTGAAGTGGTG
*GSK3B*	Frw	CCGAGGAGAACCCAATGTTT
	Rev	GCCAGCAGACCATACATCTATAC
*FGF11*	Frw	CAAAGGCATCGTCACCAAAC
	Rev	GATCAGGTTGAAGTGGGTGAA
*FRIT1*	Frw	GTGCAGGAAACCGAGTAGAA
	Rev	GCGCCTTTAGAGTGAGTGAA
*GLI4*	Frw	CTCGGAAGGTCCCAGGT
	Rev	CCCGGTGATGAGAGACTGA
*BRACHYURY*	Frw	GTAAACTCCACCAGTCCTACTTT
	Rev	TCTGTCCTTAACAGCTCAACTC
*NOTCH4*	Frw	GAGGATATCGATGAGTGCAGAAG
	Rev	TTCAAAGCCTGGGAGACAC
*ZIC1*	Frw	GAGCGACAAGCCCTATCTTT
	Rev	GGATTCGTGGACCTTCATGT
*ARC*	Frw	TCAGCTCATGACTCACCCA
	Rev	CTTGAGACCTGTTGTCACTCTC
*ALS2*	Frw	GGACTCAAAGAAGAGAAGCTCAA
	Rev	TGGCAATCTCTCTGGTGTTATG
*SOX10*	Frw	CTTCATGGTGTGGGCTCA
	Rev	CGTTCAGCAGCCTCCAG
*SMAD6*	Frw	CCTACCGTGTGCTGCAA
	Rev	GGAATCGGACAGATCCAGTG
*NGFR*	Frw	CATAGCCTTCAAGAGGTGGAAC
	Rev	CACTGTCGCTGTGGAGTTT
*MAPK6*	Frw	AGGAGCTTCTCAGCGTAATTC
	Rev	CCAGGAAATCCAGTGCTTCT
*NGFR*	Frw	CATAGCCTTCAAGAGGTGGAAC
	Rev	CACTGTCGCTGTGGAGTTT
*CD133*	Frw	GCGTCTTCCTCATGGTTGGAG
	Rev	CTTGCTCGTGTAAGGTTCACAG

**Table 2:** Number of ets motifs predicted on selected promoters and their dissimilarity score (DS) range; DS of 0% means perfect match to consensus; TRED, Transcriptional Regulatory Element Database; EPD, Eukaryotic Promoter Database. *

	Number of Predicted *ets* Binding Motifs with Different Dissimilarity Scores (DS) in Promo 3.0		
	DS: 0–1 Percent	DS: 1–5 Percent	DS: 5–10 Percent
*SRF*	-	2	1
*MCL1*	2	-	3
*LIF*	-	2	1
*SOX2 (TRED)*	-	1	1
*NANOG (TRED)*	-	1	-
*POU5F1 (TRED)*	-	1	2
*SOX2 (EPD)*	1	2	3
*NANOG (EPD)*	-	1	1
*POU5F1 (EPD)*	1	2	1

* For dissimilarity scores of individual ets motifs, see Supplementary Table S3 for URL of databases, please refer to text.

**Table 3:** Cloning primers for chosen Homo sapiens stemness gene promoters.

Promoter	Forward Primer (5′-3′)	RE Site	Reverse Primer (5′-3′)	RE Site	Product (bp)
*POU5F1*	AGACggtaccAGGGCTG TTGGCTTTGGACA	*Kpn*I	CTGTagatctAGCCATTTAA GAATTCCAGAGTAGG	*Bgl*II	993
*SOX2*	CTGTggtaccGGGGAGTG ATTATGGGAAGAA	*Kpn*I	CTGTagatctCACTAGACTG TCTTCATTCAACCGTAGC	*Bgl*II	993
*NANOG*	CTGTggtaccTTTCTGCC TAAACTAGCCA	*Kpn*I	CTGTagatctAGGTGAAGA TTCTTTACAGTCG	*Bgl*II	988

**Table 4:** Primers used for site-directed mutagenesis of cloned promoters.

Primer	Site	Oligo	Length	Tm (°C)	Ta (°C)
*NANOG-etsΔ*	Fwd	TACTAACATGAGTGTGGATC	20	59	58
	Rev	AGGAGGAAAAAATTTAAGAGG	21	57	
*POU5F1- etsΔ1*	Fwd	CCTTTCCCCCTGTCTCTG	18	64	65
	Rev	CAGGGAAAGGGACCGAGG	18	68	
*POU5F1- etsΔ2*	Fwd	GAATTGGGAACACAAAGG	18	57	57
	Rev	TGAATGAAGAACTTAATCCC	20	56	
*POU5F1- etsΔ3*	Fwd	GTGAAGTTCAATGATGCTCTTG	22	61	62
	Rev	AACCAGTTGCCCCAAACT	18	64	
*SOX2- etsΔ1*	Fwd	TTGAAATCACCCTCCCCC	18	64	65
	Rev	ATCCCACGGCACTGTATG	18	65	
*SOX2- etsΔ2*	Fwd	GTGTCTTTCCCCAGCCCC	18	69	68
	Rev	GGCGCTCAAAAGTGCAGG	18	67	

**Table 5:** The list of primers used in chromatin immunoprecipitation (ChIP) assay.

Name	Primer	Sequence	PCR Product Size
ChIP_MCL1	Frw Rev	GCCGCCCTAAAACCGTGATA CGCCTGGCTGAGAAAACTG	99
ChIP_SRF	Frw Rev	TGACAGCAACGAGTTCGGTA CCCCCATATAAAGAGATACAATGTT	130
ChIP_SOX2_ETS1	Frw Rev	TGGGAGGGAGTTTGTGACT AAAGTGCAGGCGATGGG	97
ChIP_SOX2_ETS2	Frw Rev	GTGGGATGCCAGGAAGTT GTCGTGCGGCTTTCAAATG	102
ChIP_SOX2_ETS3	Frw Rev	AGACAGTCTAGTGGGAGATGTG CGGACCATAAGGCAGACTCTA	138
ChIP_SOX2_ETS4	Frw Rev	CTTATGGTCCGAGCAGGATTT TCCCGACTAGAAGTTAGGAGAC	103
ChIP_SOX2_ETS5	Frw Rev	CGCACCTTAGCTGCTTCC GTCACACCACACGCCTTT	143
ChIP_NANOG_ETS1	Frw Rev	CTGGAGGTCCTATTTCTCTAACATC ATGCTTCAAAGCAAGGCAAG	155
ChIP_NANOG_ETS2	Frw Rev	GCAGAGGAGAATGAGTCAAAGA CCCAAACCCAACATTCAAGAAA	131
ChIP_NANOG_ETS3	Frw Rev	CTTAGTCCAGCCTGTTCCAAA AGTGAAAGACCAAAGGGAAGG	136
ChIP_POU5F1_ETS1	Frw Rev	CTTCACTGCACTGTACTCCTC CACCTCAGTTTGAATGCATGG	101
ChIP_POU5F1_ETS2	Frw Rev	GGAGTTTGTGCCAGGGTT CCCTCCAACCAGTTGCC	105
ChIP_POU5F1_ETS3	Frw Rev	GTTGGAGGGAAGGTGAAGTT TACTGTGTCCCAAGCTTCTTTAT	93
ChIP_POU5F1_ETS4	Frw Rev	CATCATCTCTGCTGGGATTACC CTGACTCTAGTTGACGTGTTGG	143
ChIP_KLF4	Frw Rev	CATAGCAACGATGGAAGGGA TCTCTCTGGTCGGGAAACT	149
ChIP_LIF1	Frw Rev	CTTCCATTCATAATTTCCTATGATGCAC CCTGATTTATATACTGGAGCCTGTG	111
ChIP_SIM2	Frw Rev	TCTTGGACCTGCAACACC TCTCGGGAAGGTCACCA	138
ChIP_ELF3	Frw Rev	CAGAGGGTGCGGGATGA GCCTTTAGACTGGGCTCCT	135
ChIP_GLUT3	Frw Rev	CCACCCTTTGCGGAGATTAT TCCTTCTCAGCAGCAAGTTT	115
ChIP_CD133	Frw Rev	TCTACAGGAAATGGATGCTGTC GGATCTGCCTCAGTCACTTAAA	131

**Table 6:** Summary of qPCR and microarray comparisons of selected potential Elk-1 target genes after Elk1-VP16 over-expression or siElk-1 silencing in neuroblastoma and glioblastoma cell lines.

	Elk-1-VP16 Overexpression					siElk-1 Silencing			
*Gene ID*	*SH-SY5Y*	*SK-N-BE (2)*	*T98G*	*A172*	*Microarray Data*	*SH-SY5Y*	*SK-N-BE (2)*	*T98G*	*A172*
*ALS2*	N/A*	N/A	−0.5	0.60	−6.06	N/A	N/A	−0.64	−1.44
*ARC*	N/A	N/A	0.44	1.67	−6.87	N/A	N/A	−2.17	−0.06
*BRACHYURY*	N/A	0.32	N/A	N/A	1.54	N/A	−0.25	N/A	N/A
*CREB3*	−0.01	N/A	0.83	0.46	−1.92	0.09	N/A	−0.74	−0.67
*CREM*	N/A	N/A	−0.91	1.40	N/A	−0.51	N/A	−0.43	0.44
*ELK-1*	1.50	2.04	8.48	8.98	13.11	−0.54	−2.36	−0.60	−1.49
*FGF11*	−0.23	N/A	N/A	0.28	−2.40	0.48	N/A	N/A	−1.31
*FRIT1*	0.17	N/A	N/A	N/A	1.82	−0.34	N/A	N/A	N/A
*FRZB*	1.21	−0.72	−0.86	N/A	1.91	−2.20	−1.34	−0.18	N/A
*GLI4*	N/A	N/A	N/A	0.695	−3.81	N/A	N/A	N/A	0.61
*GLUT3*	1.62	0.07	0.83	0.92	2.43	−0.67	−0.46	0.54	2.64
*GSK3B*	−0.22	N/A	N/A	N/A	−1.61	−0.12	N/A	N/A	N/A
*HES7*	N/A	N/A	0.68	N/A	−1.77	0.12	N/A	0.20	N/A
*IRAK3*	2.78	0.51	−0.46	N/A	1.70	−0.23	−0.81	−0.97	N/A
*LIFR*	−0.91	−1.52	−0.84	−0.08	−2.01	−0.53	0.48	−0.12	−0.82
*MAPK6*	N/A	N/A	N/A	0.76	1.64	N/A	N/A	N/A	N/A
*MEF2B*	0.09	−0.41	−1.19	N/A	−2.74	−0.19	−0.33	−1.57	N/A
*NANOG*	1.96	−0.91	0.58	1.18	2.54	−0.99	4.85	−0.07	3.12
*NODAL*	2.33	0.66	N/A	N/A	1.64	−0.66	−0.26	N/A	N/A
*NOTCH4*	N/A	N/A	N/A	3.56	3.39	N/A	N/A	N/A	7.16
*NOTO*	−2.94	0.18	−0.34	N/A	2.15	1.05	−0.08	−0.76	N/A
*PAX6*	−1.54	−0.28	N/A	N/A	2.61	1.18	−1.62	N/A	N/A
*POU5F1*	1.62	−1.20	0.56	0.32	3.68	−2.19	3.31	−0.67	1.06
*RHO*	−3.56	−1.07	−2.26	N/A	2.27	−0.20	−0.30	−0.93	N/A
*RXRB*	1.01	0.66	0.83	0.46	5.95	0.45	0.31	N/A	N/A
*SIX3*	−1.94	N/A	0.61	N/A	−5.72	1.56	N/A	0.41	N/A
*SMAD6*	N/A	N/A	N/A	−0.29	−3.78	N/A	N/A	N/A	−0.42
*SOX2*	1.54	0.41	0.37	−0.59	2.75	−0.84	−1.08	−0.56	−0.36
*SOX10*	N/A	N/A	N/A	2.94	2.41	N/A	N/A	N/A	5.83
*TCF7L1*	0.79	0.56	0.15	N/A	2.34	−0.29	−0.25	0.14	N/A
*WNT3A*	−2.11	0.53	0.11	N/A	2.25	0.86	−1.11	0.52	N/A
*ZIC1*	N/A	1.53	N/A	N/A	2.26	N/A	1.07	N/A	N/A

* N/A: the expression level is not available.

## Data Availability

Transcriptomics raw data have been uploaded to and available at EBI ArrayExpress, with accession number of E-MTAB-9938. The rest of the data are available upon request.

## Supplementary Materials

The following are available online at https://www.mdpi.com/2075-4426/11/2/125/s1, supppl1: Biological processes among enriched pathways that were upregulated in Elk-1-VP16-transfected SH-SY5Y cells, Table S2: Biological processes among enriched pathways that were downregulated in Elk-1-VP16-transfected SH-SY5Y cells, Table S3: Transcription factor binding site analysis for promoters of genes identified in microarray analysis, Table S4: Soft agar assay colony formation assay.

## References

[1] Davis S.P., Vanhoutte C., Pages J. (2000). Caboche and S. Laroche. The MAPK/ERK cascade targets both Elk-1 and cAMP response element-binding protein to control long-term potentiation-dependent gene expression in the dentate gyrus in vivo. J. Neurosci..

[2] Demir O., Ari O., Kurnaz I.A. (2012). Elk-1 interacts with dynein upon serum stimulation but independent of serine 383 phosphorylation. Cell. Mol. Neurobiol..

[3] Demir O., Korulu S., Yildiz A., Karabay A., Kurnaz I.A. (2009). Elk-1 interacts with neuronal microtubules and relocalizes to the nucleus upon phosphorylation. Mol. Cell. Neurosci..

[4] Kaminska B., Kaczmarek L., Zangenehpour S., Chaudhuri A. (1999). Rapid phosphorylation of Elk-1 transcription factor and activation of MAP kinase signal transduction pathways in response to visual stimulation. Mol. Cell. Neurosci..

[5] Sananbenesi F., Fischer A., Schrick C., Spiess J., Radulovic J. (2002). Phosphorylation of hippocampal Erk-1/2, Elk-1, and p90-Rsk-1 during contextual fear conditioning: Interactions between Erk-1/2 and Elk-1. Mol. Cell. Neurosci..

[6] Sgambato V., Vanhoutte P., Pages C., Rogard M., Hipskind R., Besson M.J., Caboche J. (1998). In vivo expression and regulation of Elk-1, a target of the extracellular-regulated kinase signaling pathway, in the adult rat brain. J. Neurosci..

[7] Yang S.H., Shore P., Willingham N., Lakey J.H., Sharrocks A.D. (1999). The mechanism of phosphorylation-inducible activation of the ETS-domain transcription factor Elk-1. EMBO J..

[8] Yang S.H., Whitmarsh A.J., Davis R.J., Sharrocks A.D. (1998). Differential targeting of MAP kinases to the ETS-domain transcription factor Elk-1. EMBO J..

[9] Sharrocks A.D. (2001). The ETS-domain transcription factor family. Nat. Rev. Mol. Cell Biol..

[10] Boros J., Donaldson I.J., O’donnell A., Odrowaz Z.A., Zeef L., Lupien M., Meyer C.A., Liu X.S., Brown M., Sharrocks A.D. (2009). Elucidation of the ELK1 target gene network reveals a role in the coordinate regulation of core components of the gene regulation machinery. Genome Res..

[11] Demir O., Aysit N., Onder Z., Turkel N., Ozturk G., Sharrocks A.D., Kurnaz I.A. (2011). ETS-domain transcription factor Elk-1 mediates neuronal survival: SMN as a potential target. Biochim. Biophys. Acta.

[12] Lenkiewicz M., Li N., Singh S.K. (2009). Culture and isolation of brain tumor initiating cells. Curr. Protoc. Stem Cell Biol..

[13] Mahller Y.Y., Williams J.P., Baird W.H., Mitton B., Grossheim J., Saeki Y., Cancelas J.A., Ratner N., Cripe T.P. (2009). Neuroblastoma cell lines contain pluripotent tumor initiating cells that are susceptible to a targeted oncolytic virus. PLoS ONE.

[14] Singh S.K., Hawkins C., Clarke I.D., Squire J.A., Bayani J., Hide T., Henkelman R.M., Cusimano M.D., Dirks P.B. (2004). Identification of human brain tumour initiating cells. Nature.

[15] Ohnishi S., Maehara O., Nakagawa K., Kameya A., Otaki K., Fujita H., Higashi R., Takagi K., Asaka M., Sakamoto N. (2013). hypoxia-inducible factors activate CD133 promoter through ETS family transcription factors. PLoS ONE.

[16] Prise I., Sharrocks A.D. (2019). ELK1 has a dual activating and repressive role in human embryonic stem cells. Wellcome Open Res..

[17] Vora P., Seyfrid M., Venugopal C., Qazi M.A., Salim S., Isserlin R., Subapanditha M., O’farrell E., Mahendram S., Singh M. (2019). Bmi1 regulates human glioblastoma stem cells through activation of differential gene networks in CD133+ brain tumor initiating cells. J. Neurooncol..

[18] Venugopal C., Hallett R., Vora P., Manoranjan B., Mahendram S., Qazi M.A., Mcfarlane N., Subapanditha M., Nolte S.M., Singh M. (2015). Pyrvinium Targets CD133 in Human Glioblastoma Brain Tumor-Initiating Cells. Clin. Cancer Res..

[19] Venugopal C., Mcfarlane N.M., Nolte S., Manoranjan B., Singh S.K. (2012). Processing of primary brain tumor tissue for stem cell assays and flow sorting. J. Vis. Exp..

[20] Kandemir B., Dag U., Bakir Gungor B., Durasi I.M., Erdogan B., Sahin E., Sezerman U., Aksan Kurnaz I. (2017). In silico analyses and global transcriptional profiling reveal novel putative targets for Pea3 transcription factor related to its function in neurons. PLoS ONE.

[21] Bolstad B.M., Irizarry R.A., Astrand M., Speed T.P. (2003). A comparison of normalization methods for high density oligonucleotide array data based on variance and bias. Bioinformatics.

[22] Kamburov A., Stelzl U., Lehrach H., Herwig R. (2013). The ConsensusPathDB interaction database: 2013 update. Nucleic Acids Res..

[23] Kanehisa M., Sato Y., Kawashima M., Furumichi M., Tanabe M. (2016). KEGG as a reference resource for gene and protein annotation. Nucleic Acids Res..

[24] Fabregat A., Sidiropoulos K., Garapati P., Gillespie M., Hausmann K., Haw R., Jassal B., Jupe S., Korninger F., Mckay S. (2016). The Reactome pathway Knowledgebase. Nucleic Acids Res..

[25] Nishimura A. (2001). BioCarta. Biotech. Softw. Internet Rep..

[26] Ashburner M., Ball C.A., Blake J.A., Botstein D., Butler H., Cherry J.M., Davis A.P., Dolinski K., Dwight S.S., Eppig J.T. (2000). Gene ontology: Tool for the unification of biology. The Gene Ontology Consortium. Nat. Genet..

[27] Fishilevich S., Zimmerman S., Kohn A., Iny Stein T., Olender T., Kolker E., Safran M., Lancet D. (2016). Genic insights from integrated human proteomics in GeneCards. Database.

[28] Messeguer X., Escudero R., Farre D., Nunez O., Martinez J., Alba M.M. (2002). PROMO: Detection of known transcription regulatory elements using species-tailored searches. Bioinformatics.

[29] Goke J., Chan Y.S., Yan J., Vingron M., Ng H.H. (2013). Genome-wide kinase-chromatin interactions reveal the regulatory network of ERK signaling in human embryonic stem cells. Mol. Cell.

[30] Borowicz S., Van Scoyk M., Avasarala S., Karuppusamy Rathinam M.K., Tauler J., Bikkavilli R.K., Winn R.A. (2014). The soft agar colony formation assay. J. Vis. Exp..

[31] Ding D.C., Liu H.W., Chang Y.H., Chu T.Y. (2017). Expression of CD133 in endometrial cancer cells and its implications. J. Cancer.

[32] Kageyama S., Liu H., Nagata M., Aoki F. (2006). The role of ETS transcription factors in transcription and development of mouse preimplantation embryos. Biochem. Biophys. Res. Commun..

[33] Bruck T., Yanuka O., Benvenisty N. (2013). Human pluripotent stem cells with distinct X inactivation status show molecular and cellular differences controlled by the X-Linked ELK-1 gene. Cell Rep..

[34] Cesari F., Brecht S., Vintersten K., Vuong L.G., Hofmann M., Klingel K., Schnorr J.J., Arsenian S., Schild H., Herdegen T. (2004). Mice deficient for the ets transcription factor elk-1 show normal immune responses and mildly impaired neuronal gene activation. Mol. Cell. Biol..

[35] Bunina D., Abazova N., Diaz N., Noh K.M., Krijgsveld J., Zaugg J.B. (2020). Genomic Rewiring of SOX2 Chromatin Interaction Network during Differentiation of ESCs to Postmitotic Neurons. Cell Syst..

[36] Zhang L., Gong Y., Zhao X., Zhou H. (2016). Comparative study between hypoxia and hypoxia mimetic agents on osteogenesis of bone marrow mesenchymal stem cells in mouse. Zhongguo Xiu Fu Chong Jian Wai Ke Za Zhi.

[37] Park S.W., Do H.J., Choi W., Song H., Chung H.J., Kim J.H. (2017). NANOG gene expression is regulated by the ETS transcription factor ETV4 in human embryonic carcinoma NCCIT cells. Biochem. Biophys. Res. Commun..

[38] Park S.W., Do H.J., Ha W.T., Han M.H., Park K.H., Song H., Kim N.H., Kim J.H. (2014). Transcriptional activation of OCT4 by the ETS transcription factor PEA3 in NCCIT human embryonic carcinoma cells. FEBS Lett..

[39] Akagi T., Kuure S., Uranishi K., Koide H., Costantini F., Yokota T. (2015). ETS-related transcription factors ETV4 and ETV5 are involved in proliferation and induction of differentiation-associated genes in embryonic stem(ES) cells. J. Biol. Chem..

[40] Huh S., Song H.R., Jeong G.R., Jang H., Seo N.H., Lee J.H., Yi J.Y., Lee B., Choi H.W., Do J.T. (2018). Suppression of the ERK-SRF axis facilitates somatic cell reprogramming. Exp. Mol. Med..

[41] Vora P., Venugopal C., Mcfarlane N., Singh S.K. (2015). Culture and Isolation of Brain Tumor Initiating Cells. Curr. Protoc. Stem Cell Biol..

[42] Tabu K., Kimura T., Sasai K., Wang L., Bizen N., Nishihara H., Taga T., Tanaka S. (2010). Analysis of an alternative human CD133 promoter reveals the implication of Ras/ERK pathway in tumor stem-like hallmarks. Mol. Cancer.

