# Supplementary material for: ETS-Domain Transcription Factor Elk-1 Regulates Stemness Genes in Brain Tumors and CD133+ BrainTumor-Initiating Cells
Source: J Pers Med. 2021 Feb 14;11(2):125. doi: 10.3390/jpm11020125 (PMC7917801; doi:10.3390/jpm11020125)
Supplement: Supplementary file 1 [file jpm-11-00125-s001.zip › SupplementaryTables-Kurnaz-20jan2021_son.docx]

**Supplementary Tables.**

**Supplementary Table S1.** Biological Processes among enriched pathways that were upregulated in Elk-1-VP16-transfected SH-SY5Y cells. GO: Gene ontology, R-HAS: Reactome, path:has: KEGG term. (Levels 2 and 3 are included in the Table)

| **GO ID** | **Term Level** | **Term Name** | **P-value** | **Q-value** | **Members** |
| --- | --- | --- | --- | --- | --- |
| GO:0048856 | 2 | anatomical structure development | 3,15E-09 | 2,22E-08 | PCDHB16; HIST1H4B; HIST1H4A; ZNF160; HIST1H4F; HIST1H4E; PCSK2; HIST1H4I; PDE6C; PCSK6; SCG2; HIST1H4L; JPH2; HIPK2; B2M; PCSK9; SOHLH2; NUP210L; AGT; HIF3A; FA2H; LHX1; OGDH; LHX4; BLOC1S2; LHX6; TYROBP; NR0B2; SOX6; CYP46A1; PIK3CD; NPHS1; PTGER3; ATPIF1; SUMO1; GAL3ST1; PARVA; LBX1; SLC6A17; IFNA13; IRAK3; IFNA10; IFNA17; IFNA16; IFNA14; ARHGEF10; NEUROD2; NPR2; NPR3; SLITRK3; GIP; ZNF683; DMP1; SP6; GPR37L1; EHF; NEUROG3; MEG3; OPA3; CSF3R; GATA4; CXCL12; CXCL14; ADRA1A; COL15A1; ADRA1B; SFRP4; LST1; VCX2; STAC3; LECT2; PTEN; DACT2; PLXDC1; MAG; MAK; MAL; MMP14; CD1D; ACTA1; IL27; RASGRP4; TNFSF14; TDG; CLDN11; RPS27A; ITGA4; MMP19; TWIST2; MYO18B; NACA; TPM1; SLC2A14; FARP2; CRYBA1; SERPINE1; APOA2; CRYBA4; DNAI1; APOD; TNNT2; LILRB3; LILRB2; LILRB1; DRP2; SOX13; SOX14; ADRB2; APOH; BVES; ZIC5; HRH2; JAK3; GDF6; GTF2I; SPRR2E; CCL24; TTN; GDF10; FOXJ1; HOXB8; HOXB9; FST; HOXB2; HOXB3; KCNQ4; HOXB1; HOXB6; HOXB7; DSP; HOXB5; SMTNL1; HOXC12; COL4A3; PPARA; HCK; CHST2; LHX5; ITGAX; SERF1B; MB; NOG; KRT17; IL12RB1; KRT15; KRT13; PRDM1; LHX9; NOV; FLNC; CRIP1; FOXC2; KRT19; KRT18; ACP5; GHRH; MYH14; MYH15; GHRL; THRB; KRT16; UPK1A; TNFRSF10C; SHH; DYRK3; POLB; ADCYAP1; FGF6; KLHL6; COL2A1; PADI2; MYF5; NCOA3; RPS4Y1; TRIM71; STRC; NR2F2; PCDHB13; ACAT1; SH3GL3; S100A9; S100A8; ATOH1; S100A7; GGN; S100A1; LAMA1; SRPX2; CCBE1; MYLK2; CHRDL2; BARX2; SPIC; LTA; BARX1; TRPV4; PLA2G10; UBE2V2; OLIG3; YAP1; IL2RA; F2; EDA; PGLYRP2; MSGN1; LMO2; EMX2; EMX1; VAV1; IFNA21; SSTR4; NDE1; MYL6B; LMOD1; H2BFS; NCKAP1L; KCNC1; MYF6; HNF4A; PSMD6; EGFR; CAT; PLCG2; PGLYRP1; ERG; AHSG; MYOC; GIPR; MYOG; RS1; EIF2S2; FRG1; SIM2; GFI1B; GJB1; PDE1B; GJB3; GJB2; GJB5; SULF1; CXCR3; KLF5; PROP1; CRYGD; H1FNT; APOL2; NUMBL; NOS1; ITGB3; ITGB2; ITGB4; LTBP1; DDX17; CRTAC1; SLC24A4; BFSP2; CD40; BFSP1; MST1; POGK; IGHD; CAMK2B; RPL29; PHOX2B; MMP21; STAT5A; TCHH; HOXB13; SOX21; ARSE; CA9; SNW1; BNC1; KLF15; PER2; GLRA2; DKK4; LCE5A; DKK1; CATSPER4; CDH23; ZNF541; CHRND; MT3; PTCHD3; GJE1; ZNF396; SLC1A3; SFTPD; WNT3A; TGM2; TGM3; COL11A2; ZFPM2; DNAI2; SPTB; GSTA1; GSTA2; TFAP2D; C5AR1; TNNC1; CHI3L1; RASAL1; MIP; DAB1; RPL22; CLSTN2; LBP; ADAMTS7; GATA5; PIWIL1; IQCF1; ADAMTS9; TIAL1; FGR; SLC12A5; EAF2; CLEC1B; CDH1; CDH3; RP1L1; CDH5; CDH4; RAX; WT1; IL31RA; SLCO4C1; TPO; LDB2; ESX1; CLCF1; KLK3; KLK4; KLK5; KLK6; KLK7; KLK8; KIRREL3; HRG; LELP1; GH1; RFX4; SYT2; C1QC; C1QB; SFN; PLEKHA5; UBA52; GNG5; MYCNOS; RHO; PLAGL2; SPRR4; CCL3; GHRHR; NHLH1; CCL5; SERINC5; TBR1; COLQ; MDGA1; FERD3L; LAMB3; IBSP; MESP2; AMELX; HSBP1; LCE1F; MYCN; LCE1D; NEK5; NEK2; LCE1C; SCGB1A1; LCE1A; IFNA7; CEL; CLEC5A; CEACAM1; DMBT1; EN2; CEACAM5; LCE1E; LRRTM2; TACSTD2; MT1G; CLDN4; ANGPT4; DPYSL2; POU3F4; FZD1; OXT; SPI1; PCDHA10; SPRR3; FBN3; IGFBP1; SPDEF; EIF4E; MYH6; PHEX; CLIC5; ZBTB16; GJA4; GJA8; SFRP5; CALML5; MYBPC3; RPL7L1; SCN5A; PROC; GAS7; COX17; CD74; SERF1A; MFNG; CHAD; ONECUT1; RPGRIP1; FOXE1; FOXE3; CCDC40; FABP5; TRPC6; NHLH2; CHAT; KIF17; PRM2; FLT4; TBPL2; MXD1; FLT1; ZFPM1; TTBK1; CYP1B1; MYH7; NOTCH2NL; IPMK; CDKL5; RPS6KB1; C1QBP; MMP20; IGF2BP1; DOCK2; OLIG1; TNFSF8; TBX19; PRELP; KLHL10; NTF3; NLGN3; FGD3; FGD2; KRTAP5-9; CLC; OTOP1; COL5A3; ASGR2; COL5A1; FOXL2; FOXL1; RPS4X; HAP1; IGF2BP2; TTC26; RAB25; LRRC38; CXADR; KEL; ITK; NCOA1; ACTL8; IRS2; FGF16; HOXA7; SFRP2; HOXA5; HOXA4; HOXA3; HOXA2; ANKRD2; AMIGO1; SLAMF6; AMIGO2; CRHR2; NEFL; HOXA9; ATP5F1; TGFBR3; TGFB2; ACSBG2; PTK7; CECR1; GRSF1; HRNR; LGI4; CYP17A1; SOX9; ALOX12; PITX2; PITX3; PCDHA11; PITX1; ITGB1BP1; FZD10; LOXL1; DRG1; NKX2-3; DMBX1; CEBPE; RND1; CAMP; FYN; ALX4; SCN2B; TOR1A; TNNI3; ABCB5; AURKC; HCLS1; PHLDA2; PACSIN1; FOXB1; DNAJC19; MUSTN1; CCKBR; MATN3; MATN2; DHH; PRKCA; CDC42EP1; CHRM3; LRRC10; PRKCG; CES1; MTSS1; EBF3; EBF2; VCX; VCY; IL6R; PBX2; BDH1; CHST11; SYT17; VAMP5; WFIKKN2; WFIKKN1; PFKFB1; GLDN; TLX1; AICDA; PRLR; HIST1H4K; MMP9; OTP; CALCA; CYP26B1; CMTM5; CMKLR1; LRRN3; DGAT2; DYNLL1; CRLF1; TBX21; TNS3; CD34; LIN7C; CD36; SOX15; RAPGEF5; PAX8; MYL2; PDPN; RLN2; RAPGEF3; APCDD1; TXNDC2; CAV3; AXL; CAV1; LTBR; PECAM1; CALM2; CALM1; CNGB1; TNFRSF13B; GADD45B; RORC; DCC; CBFA2T3; POU6F2; INHBB; MARCKS; INHBE; FMOD; MARVELD2; RIPK4; PCLO; COL17A1; PRTN3; H3F3B; ERBB2; ERBB3; AQP5; BMP8B; AQP1; EXTL1; CDH22; SNTG2; EVX1; WNK4; HOXD3; FLRT1; BGLAP; CTSZ; SEMA3B; REG3G; SETD3; FGFR1; CXCL1; ADCY1; NME2; DHODH; RUNX2; RUNX3; IHH; FOXA2; EDAR; CCL19; DLL4; C1GALT1C1; LRTM2; REG3A; CD3D; PAPPA2; HELT; PKP1; ADORA1; GNAO1; CRX; DUOX2; EPB42; ALPK3; CCKAR; TNFRSF17; VENTX; ITGA11; NPTX1; S100A10; TSHR; RGN; MARK4; SLC23A1; PCDHB9; SLC9A1; HOXA6; LIM2; CHODL; AFF2; PPARGC1B; PCDHB4; PLEKHB1; RAPH1; VIL1; OMP; NFE2; PRM1; IL20; RREB1; NPPB; ITGAM; PDCL3; ADAMTS4; FRZB; PRKACB; PAX5; PAX4; PAX7; PAX6; HOXB4; PAX3; LAMC2; SPRR2D; SPRR2G; SPRR2F; SPRR2A; PTPN6; PTPN5; SPRR2B; HEMGN; ANG; ESR2; BTF3; NANOS2; SMYD3; OTOF; ZNF135; ATP8A2; SMPD3; TDRD9; SLAMF1; SLCO2B1; MIXL1; IL13; MOG; LCK; DPF3; THEG; DMRTC2; DHRS3; KRT3; CCR1; KRT1; CCR3; CCR4; GNG12; CDSN; TBX5; BATF; CRYGB; KRT8; KCNA2; MEOX1; BCR; CD79A; LILRB4; WNT3; STAB2; HOXA13; HOXA11; HOXA10; PSMA2; PSMA3; PSMA1; KRT6B; PSMA4; CASR; C3; PSMA8; ALOX12B; KDR; EPB41L3; LHX2; TAL1; FBXO40; NDN; COMP; EFNA5; EFNA2; CARD11; SALL4; LGALS9; SALL1; PLA2G2D; SALL3; UCHL5; TNP2; FSCN3; LRP2; CYP1A1; CYP1A2; LRP1; LRP6; RPSA; LCE2D; IL10; WNT2; GNAS; RARRES2; MAEL; FGA; IL4; CNTNAP2; IL3; COL1A1; BLNK; MYOCD; CABP4; CDH15; CDH17; CRISPLD2; RASGRP1; ECM2; ECM1; LOR; SOX2; INSR; IL23R; PAEP; SOX3; LTA4H; LOX; SMOC1; KLF13; HIST4H4; GCNT1; WASF1; GCNT3; HLA-DOA; GDF3; GDF2; SPAG9; CATSPER1; GDF5; PPL; PHOSPHO1; OVOL2; NRG3; MBP; SPINK2; EYA2; RPS7; RTL1; ANXA4; HSPB1; SPANXB1; DKKL1; OSR2; MEST; MTPN; POU5F1; HPN; TMBIM1; GRHL1; PCDHA2; PCDHA3; VCX3A; PCDHA7; PCDHA4; PCDHA5; DMRTA2; PCDHA8; DRD3; CASP8; DRD1; PURB; NFAM1; NDUFS4; WNT9B; SCNN1B; COL6A3; BCL3; ALPL; TMOD4; YWHAQ; TIMP3; TIMP2; TBX6; MYH11; GLRX5; MC2R; CDX4; KLK14; CASP10; EPO; CASP14; CCK; NKX2-2; APLP1; DYX1C1; NKX2-6; NKX2-5; HYDIN; PKD1L1; NKX6-2; FSTL4; ITSN2; POU2F2; PTGIS; MICAL2; WNT16; TLE2; EDN3; UPK2; SLC4A5; TNP1; TBX4; CD28; SOD1; TNNI1; LY9; HTR6; FUT10; UMODL1; ABHD2; SEMA7A; NOX1; NKX6-1; SOCS5; CD4; SOCS7; BMP4; BARHL2; HAPLN2; IRX2; TNMD; CCR2; DDX5; HIST1H2BA; TBX1; PALLD; UBB; PENK; GRIP1; STC1; MYO15A; SOX10; RNF2; HTR5A; VDR; STMN1; KRT5; NRXN3; SYT4; OLFM4; CSF1; DPPA2; DPPA3; NOX5; DPPA5; SPRR1A; SPRR1B; PCDHB3; FLG; TGFA; CD79B; THBS2; IGSF10; SBDS; COL27A1; ADAM19; NR5A2; DLX4; PCDHB2; OSR1; GPRC5B; DLX1; DLX2; DLX3; NXF5; ODF1; ACVRL1; IL4R; NXF2; ODF4; SDK2; RHOQ; TLR5; LMX1A; LMX1B; CMA1; RHOH; SLC26A5; FGF9; FGF8; FGF7; EVPL; RHOA; MTNR1A; PLEK; BCAN; IL1RN; VTN; ETV2; ETV7; IVL; VAV2; HMOX1; HOXD13; HOXD10; RGMA; POU4F3; SPOCK1; BATF2; WWTR1; SOST; ACE; EPHA8; SLC32A1; NR2E3; NFATC2; EPHA1; LCE2B; ATP5J; SS18L1; KNDC1; CNR1; GBX2; FCER1G; NANOG; CCDC63; LCE3D; LCE3E; PCDHB11; SGCA; PCDHB6; PHIP; SRD5A2; FGF10; TCF21; PDCD1; NKD1; TBXA2R; CECR2; MACF1; PCDHB15; ESRRB; PDILT; KRT6A; HMGA2; ZFP36L1; PAQR7; PCDHB5; UNC13D; CHRNA1; SERPINF2; ZAP70; GHSR; ANKS1A; HEY2; CCL11; CCL13; RASGRF1; CCL17; ADAMTS12; IFNA2; IFNA1; ID2; IFNA6; IFNA4; LEP; NGRN; CTGF; EMP2; PRM3; EMP1; HSP90AA1; PSMB2; HIVEP3; HMX2; OLFML3; SP8; BMPER; CLPS; HAX1; LATS2; RIPK3; LENEP; TDGF1; GPR55; GNAT1; DSCAM; ELF3; P2RX2; MSC; CPNE9; BCL2L11; TNFRSF1B; GRHL2; GRHL3; SEMA6B; CPNE6; CPNE5; GLI2; WNT8A; CREB3L1; TG; MMD2; LDHA; HILS1; ZFP57; COL8A2; COL8A1; KCNJ10; ULK4; TNR; MLXIPL; CHRDL1; UMOD; TMPRSS6; FOXD3; TTLL7; AR; LUZP1; LRRC55; SSBP1; LCE1B; TNN; FOXN1; NPM2; PCDH8; DBX1; PTPRS; GFI1; RPL38; RHCG; PCDH1; BBS2; BBS5; UNC45B; DSPP; GDNF; TSHZ1; ALOX15B; NOTO; FOLR1; TNFRSF1A |
| GO:0008283 | 2 | cell proliferation | 6,43E-08 | 3,51E-07 | SCG2; HIPK2; AGT; BLOC1S2; LHX9; ATPIF1; IFNA13; IFNA10; IFNA17; IFNA16; IFNA14; SIRPG; LRP2; IGF1R; NPR3; CYP1A1; SP6; FTMT; EHF; TNS3; MEG3; NOX1; CXCL12; ADRA1A; ADRA1B; LST1; TNMD; PTEN; CTBP2; MMP14; CD1D; RASGRP4; TNFSF14; ALDH3A1; ACVRL1; IFIT3; BNIPL; APOD; HPSE2; LILRB2; LILRB1; SOX10; SOX15; APOH; JAK3; REG3G; CCL23; CCL24; REG3A; FOXJ1; IL5RA; HOXB4; COL4A3; BLK; HCK; TACR1; LHX5; NOG; TCIRG1; KRT16; PRDM1; NOV; CRIP1; FOXC2; GHRH; GHRL; TNFRSF10C; WFDC1; ADCYAP1; CKS1B; NR2F2; LTA; UBE2V2; YAP1; IL2RA; F2; EMX2; EMX1; IFNA21; SSTR4; EBI3; NCKAP1L; RPL23A; HLA-DPB1; HNF4A; EGFR; ERG; NDE1; MYOG; EIF2S2; GFI1B; SULF1; CXCR3; CDCA7; MNDA; CNOT6; KLF9; NUMBL; NOS1; ITGB3; CD40; IGHD; PHOX2B; BNC1; RAB25; MUSTN1; GDF2; SFTPD; WNT3A; C5AR1; FGR; EAF2; FGFBP1; CDH3; CDH5; WT1; IFITM1; IL31RA; HLA-DRB1; LGI4; CLCF1; KLK8; HRG; PYY; CSF1; SFN; GNG5; MYCNOS; TIAL1; CD300A; GHRHR; CCL5; ADRB2; PLA2G1B; AMELX; MYCN; SLC11A1; IHH; CEACAM1; DERL2; TACSTD2; ANGPT4; CLDN7; SHH; CKLF; JTB; RETNLB; AZGP1; SCN5A; CD74; FOXE3; FABP6; FABP3; FLT4; MXD1; FLT1; PODN; CYP1B1; ZBTB16; RPS6KB1; TPM1; DOCK2; TNFSF8; DUX4; TBX19; TGFBR3; NTF3; CKS2; CLC; DLL4; RPS4X; PDCD5; ITK; CD209; HHLA2; IRS2; HOXA5; HOXA3; SLAMF6; SLAMF1; TGFB2; CD244; SOX9; MYOCD; PITX2; ITGB1BP1; CAMP; FYN; VIPR2; ZAP70; VIPR1; HCLS1; RAC2; PRKCA; CHRM5; CHRM3; PYDC1; MTSS1; IL6R; CHST11; CLEC4G; PRAMEF4; SSR1; PRAMEF6; PRAMEF7; PRAMEF1; PRAMEF2; OTP; CALCA; PRAMEF8; CD38; CRLF1; CD33; CD34; MST1; IGF2BP1; IL24; IL27; RAPGEF3; CAV3; CAV1; LTBR; IL12RB1; TNFRSF13B; IL12RB2; CBFA2T3; MARVELD3; PRTN3; ERBB2; ERBB3; AQP1; TRIM24; SLC25A5; CGRRF1; PER2; MDM4; FGFR1; CXCL1; CXCL2; CXCL5; NME2; INCA1; PRAMEF10; RUNX2; RUNX3; APOBEC1; VTCN1; IFNA2; CCL26; KIFAP3; YME1L1; ADORA1; IFNA7; TNFRSF17; TSHR; ICOSLG; RGN; CCKBR; CDC16; STAT5A; PDPN; RNF139; NKX2-5; EPHB6; PDCL3; FRZB; LBX1; PAX7; PAX6; PTPN6; ANG; ESR2; TNFRSF1A; SLURP1; CDC14A; LCK; FGF21; RPS15A; CD4; CD5; CCR2; CCR3; PTAFR; TBX5; KCNA5; CD79A; MARK4; CASR; FTH1; PENK; MORF4L1; RPS27; TAL1; NDN; SALL4; LGALS9; PLA2G2D; PLA2G2F; TNFRSF1B; CD180; SFRP2; GPR37L1; LRP1; LRP6; SGK2; SFRP4; SFRP5; IL10; WNT2; IL13; PLG; IL4; IL3; ALOX12; TRIM71; ECM1; GNAT1; INSR; LACRT; IL23R; UCN2; KLF13; MT3; TFAP2D; GDF5; OVOL2; SPINK2; PDZK1; REG1B; OSR1; OSR2; HPN; DUSP15; ANKRD2; DMRTA2; DRD3; PURB; NDUFS4; PTGS1; TIMP2; EPO; CCK; S100A11; NKX2-3; NKX2-6; RREB1; ZP4; XCL1; WNT16; EDN3; CD28; ENPP7; TSPAN1; NKX6-1; BMP4; CD6; TBX1; STC1; CCL3L3; CCL3L1; VDR; RASSF5; FA2H; DPPA2; GLUL; NOX5; UBE2L3; TGFA; PLAC8; SBDS; NR5A2; RPGRIP1; CARD11; NACA; C1QL4; IL4R; RNF43; FGF9; FGF8; FGF7; FGF6; RHOA; SCGB1A1; CDC27; HMOX1; HOXD13; TSPAN32; AIF1; ACE; BTN3A1; NFATC2; EPHA1; GBX2; IL9R; NANOG; PHIP; FGF10; FGF16; SETMAR; GML; ATP8A2; KRT6A; HMGA2; ZFP36L1; SERPINF2; HEY2; CCL11; CCL14; RASGRF1; WWTR1; CCL19; IFNA1; ID2; IFNA6; IFNA4; LEP; CTGF; EMP2; KDR; EMP1; HMX2; BMPER; TFF1; RIPK3; TDGF1; HIST1H2AE; FAM83A; HIST1H2AC; HIST1H2AB; GRHL2; PRAMEF5; BLZF1; GLI2; SOX2; COL8A2; COL8A1; KLB; MLXIPL; UMOD; AR; NR2E3; NPM1; FOXN1; GDNF; ALOX15B |
| GO:0044767 | 2 | single-organism developmental process | 2,07E-07 | 1,08E-06 | PCDHB16; HIST1H4B; HIST1H4A; ZNF160; HIST1H4F; HIST1H4E; PCSK2; HIST1H4I; PDE6C; PCSK6; SCG2; HIST1H4L; JPH2; HIPK2; B2M; PCSK9; SOHLH2; NUP210L; AGT; HIF3A; FA2H; LHX1; OGDH; LHX4; BLOC1S2; LHX6; TYROBP; NR0B2; SOX6; CYP46A1; PIK3CD; ZNF706; NPHS1; PTGER3; ATPIF1; HIST1H4K; GAL3ST1; WWC1; PARVA; LBX1; SLC6A17; IFNA13; RPS3A; IFNA10; IFNA17; IFNA16; IFNA14; ARHGEF10; NEUROD2; NPR2; NPR3; SLITRK3; GIP; ZNF683; DMP1; SP6; GPR37L1; EHF; NEUROG3; MEG3; OPA3; CSF3R; GATA4; CXCL12; CXCL14; ADRA1A; COL15A1; ADRA1B; SFRP4; LST1; VCX2; STAC3; LECT2; FKBP6; PTEN; DACT2; PLXDC1; MAG; CTBP2; MAL; MMP14; CD1D; ACTA1; IL27; RASGRP4; TNFSF14; TDG; CLDN11; ALDH3A1; ITGA4; MMP19; TWIST2; MYO18B; NACA; TPM1; SLC2A14; FARP2; CRYBA1; SERPINE1; APOA2; CRYBA4; DNAI1; APOD; TNNT2; LILRB3; LILRB2; LILRB1; DRP2; SOX13; SOX14; ADRB2; APOH; BVES; ZIC5; HRH2; JAK3; GDF6; GTF2I; CCL24; TTN; GDF10; FOXJ1; HOXB8; HOXB9; FST; HOXB2; HOXB3; KCNQ4; HOXB1; HOXB6; HOXB7; DSP; HOXB5; SMTNL1; HOXC12; COL4A3; BLK; PPARA; HCK; CHST2; TACR3; LHX5; ITGAX; MB; NOG; KRT17; IL12RB1; KRT13; PRDM1; LHX9; NOV; FLNC; CRIP1; FOXC2; KRT19; HIST1H2BA; ACP5; GHRH; MYH14; MYH15; GHRL; THRB; KRT16; UPK1A; TNFRSF10C; SHH; DYRK3; DLK1; POLB; ADCYAP1; FGF6; COL2A1; ZCCHC11; PADI2; MYF5; NCOA3; RPS4Y1; TRIM71; CIITA; STRC; NR2F2; PCDHB13; ACAT1; SH3GL3; S100A9; S100A8; ATOH1; S100A7; GGN; S100A1; LAMA1; VCX; SRPX2; CCBE1; MYLK2; CHRDL2; BARX2; SPIC; LTA; BARX1; IRAK3; PLA2G10; UBE2V2; OLIG3; YAP1; IL2RA; F2; EDA; PGLYRP2; MSGN1; LMO2; EMX2; EMX1; VAV1; IFNA21; SSTR4; HSPE1; NDE1; MYL6B; LMOD1; H2BFS; NCKAP1L; KCNC1; MYF6; HMOX1; PSMD6; EGFR; CAT; PLCG2; PGLYRP1; ERG; AHSG; MYOC; GIPR; MYOG; RS1; EIF2S2; FRG1; SIM2; GFI1B; GJB1; PDE1B; GJB3; GJB2; GJB5; SULF1; CXCR3; KLF5; PROP1; CRYGD; H1FNT; APOL2; NUMBL; NOS1; ITGB3; ITGB2; ITGB4; LTBP1; DDX17; CRTAC1; SLC24A4; BFSP2; CD40; BFSP1; MST1; POGK; IGHD; CAMK2B; RPL29; PHOX2B; MMP21; STAT5A; TCHH; HOXB13; SOX21; ARSE; CA9; SNW1; HAP1; KLF15; PER2; GLRA2; DKK4; RAB25; LCE5A; DKK1; CATSPER4; CDH23; ZNF541; CHRND; MT3; PTCHD3; TRIM6; CLIC5; GJE1; ZNF396; SLC1A3; SFTPD; WNT3A; TGM2; TGM3; COL11A2; ZFPM2; DNAI2; SPTB; GSTA1; GSTA2; TFAP2D; C5AR1; TNNC1; CHI3L1; RASAL1; MIP; DAB1; RPL22; CLSTN2; LBP; ADAMTS7; GATA5; PIWIL1; IQCF1; ADAMTS9; RHO; FGR; SLC12A5; EAF2; CLEC1B; CDH1; CDH3; RP1L1; CDH5; CDH4; RAX; WT1; IFITM1; IL31RA; SLCO4C1; TPO; LDB2; ESX1; CLCF1; KLK3; KLK4; KLK6; KLK8; KIRREL3; HRG; LELP1; GH1; RFX4; SYT2; SLC2A4; C1QC; C1QB; SFN; PLEKHA5; UBA52; GNG5; MYCNOS; ELK4; PLAGL2; TRPV4; CCL3; GHRHR; NHLH1; CCL5; SERINC5; TBR1; COLQ; MDGA1; FERD3L; LAMB3; IBSP; MESP2; AMELX; HSBP1; LCE1F; MYCN; LCE1D; NEK5; NEK2; LCE1C; SCGB1A1; LCE1A; IFNA7; POLR2L; CEL; CLEC5A; CEACAM1; DMBT1; EN2; CEACAM5; LCE1E; LRRTM2; TACSTD2; MT1G; CLDN4; ANGPT4; STEAP4; DPYSL2; POU3F4; LGALS12; OXT; SPI1; PCDHA10; SPRR3; OSR1; IGFBP1; SPDEF; EIF4E; MYH6; PHEX; MAK; ZBTB16; GJA4; GJA8; SFRP5; MYBPC3; RPL7L1; SCN5A; PROC; GAS7; COX17; CD74; SERF1A; MFNG; CHAD; ONECUT1; RPGRIP1; FOXE1; FOXE3; CCDC40; SERF1B; TRPC6; NHLH2; CHAT; KIF17; PRM2; FLT4; TBPL2; FZD1; MXD1; FLT1; ZFPM1; TTBK1; CYP1B1; MYH7; NOTCH2NL; IPMK; CDKL5; SPRR4; RPS6KB1; C1QBP; MMP20; IGF2BP1; DOCK2; OLIG1; TNFSF8; TBX19; PRELP; IL20; KLHL10; NTF3; NLGN3; FGD3; FGD2; CLC; OTOP1; COL5A3; ASGR2; COL5A1; FOXL2; FOXL1; RPS4X; UPK2; TTC26; PCDHA7; LRRC38; CXADR; KEL; ITK; NCOA1; ACTL8; IRS2; FGF16; HOXA7; SFRP2; HOXA5; HOXA4; HOXA3; HOXA2; ANKRD2; AMIGO1; SLAMF6; AMIGO2; CRHR2; NEFL; HOXA9; ATP5F1; TGFBR3; TGFB2; ACSBG2; PTK7; CECR1; GRSF1; HRNR; LGI4; CYP17A1; SOX9; ALOX12; PITX2; PITX3; PCDHA11; PITX1; ITGB1BP1; FZD10; LOXL1; DRG1; NKX2-3; DMBX1; CEBPE; RND1; CAMP; FYN; ALX4; SCN2B; TOR1A; TNNI3; ABCB5; AURKC; HCLS1; USH1C; PHLDA2; PACSIN1; FOXB1; DNAJC19; MUSTN1; CCKBR; MATN3; MATN2; DHH; PRKCA; CDC42EP1; CHRM3; LRRC10; PRKCG; KRTAP4-7; CES1; MTSS1; EBF3; PADI4; FFAR2; VCY; IL6R; PBX2; BDH1; CHST11; SYT17; VAMP5; HNF4A; WFIKKN1; PFKFB1; GLDN; TLX1; AICDA; PRAMEF4; PRLR; PRAMEF6; PRAMEF7; PRAMEF1; PRAMEF2; MMP9; OTP; CALCA; CYP26B1; CMTM5; CMKLR1; LRRN3; DGAT2; DYNLL1; CRLF1; TBX21; TNS3; CD34; LIN7C; CD36; SOX15; RAPGEF5; PAX8; MYL2; PDPN; RLN2; RAPGEF3; APCDD1; TXNDC2; CAV3; AXL; CAV1; LTBR; PECAM1; PRAMEF8; CALM2; CALM1; CNGB1; TNFRSF13B; GADD45B; RORC; DCC; CBFA2T3; POU6F2; INHBB; MARCKS; INHBE; FMOD; MARVELD2; RIPK4; PCLO; PRTN3; H3F3B; ERBB2; ERBB3; AQP5; CETP; BMP8B; AQP1; EXTL1; CDH22; SNTG2; EVX1; WNK4; HOXD3; FLRT1; BGLAP; CTSZ; SEMA3B; REG3G; SETD3; FGFR1; CXCL1; ADCY1; NME2; DHODH; PRAMEF10; RUNX2; RUNX3; IHH; FOXA2; EDAR; CCL19; DLL4; C1GALT1C1; LRTM2; REG3A; CD3D; PAPPA2; HELT; PKP1; ADORA1; GNAO1; CRX; DUOX2; EPB42; ALPK3; CCKAR; TNFRSF17; VENTX; ITGA11; NPTX1; S100A10; TSHR; RGN; MARK4; SLC23A1; PCDHB9; SLC9A1; HOXA6; LIM2; CHODL; AFF2; PPARGC1B; PCDHB4; PLEKHB1; RAPH1; VIL1; OMP; NFE2; MADCAM1; PRM1; ANXA13; RREB1; NPPB; ITGAM; PDCL3; ADAMTS4; FRZB; PRKACB; PAX5; PAX4; PAX7; PAX6; HOXB4; PAX3; SPRR2E; SPRR2D; SPRR2G; SPRR2F; SPRR2A; PTPN6; PTPN5; SPRR2B; HEMGN; ANG; ESR2; BTF3; NANOS2; SMYD3; OTOF; ZNF135; ATP8A2; SMPD3; SLAMF1; SLCO2B1; MIXL1; IL13; MOG; LCK; DPF3; THEG; DMRTC2; DHRS3; KRT3; CCR1; KRT1; CCR3; CCR4; GNG12; CDSN; TBX5; BATF; CRYGB; KRT8; KCNA2; MEOX1; BCR; CD79A; LILRB4; WNT3; BCAP29; HOXA13; HOXA11; HOXA10; PSMA2; PSMA3; DMRTA2; PSMA1; PSMA4; CASR; C3; PSMA8; ALOX12B; KDR; EPB41L3; LHX2; TAL1; FBXO40; NDN; COMP; EFNA5; EFNA2; PLA2G2A; SALL4; LGALS9; SALL1; PLA2G2D; SALL3; UCHL5; TNP2; FSCN3; LRP2; CYP1A1; CYP1A2; LRP1; LRP6; RPSA; LCE2D; IL10; WNT2; GNAS; RARRES2; MAEL; FGA; IL4; CNTNAP2; IL3; COL1A1; BLNK; CALR3; MYOCD; CABP4; CDH15; CDH17; CRISPLD2; RASGRP1; ECM2; ECM1; LOR; SOX2; INSR; RUNX1T1; IL23R; PAEP; SOX3; LTA4H; LOX; SMOC1; KLF13; HIST4H4; GCNT1; WASF1; GCNT3; HLA-DOA; GDF3; GDF2; SPAG9; CATSPER1; GDF5; PPL; PHOSPHO1; OVOL2; NRG3; MBP; SPINK2; EYA2; RPS7; RTL1; ANXA4; HSPB1; SPANXB1; DKKL1; OSR2; MTPN; POU5F1; HPN; GRHL1; PCDHA2; PCDHA3; VCX3A; GRHL2; PCDHA4; PCDHA5; TBX1; FGF22; PCDHA8; GRHL3; DRD3; CASP8; DRD1; PURB; NFAM1; NDUFS4; WNT9B; SCNN1B; COL6A3; BCL3; ALPL; TMOD4; PTGS1; YWHAQ; TIMP3; TIMP2; TBX6; MYH11; GLRX5; MC2R; CDX4; KLK14; CASP10; EPO; CASP14; CCK; NKX2-2; APLP1; DYX1C1; NKX2-6; CANX; NKX2-5; HYDIN; PKD1L1; NKX6-2; FSTL4; ITSN2; POU2F2; ADAMTS20; PTGIS; MICAL2; WNT16; TIAL1; TLE2; EDN3; RNASEL; SLC4A5; TNP1; TDRD9; CD28; SPAG8; SOD1; TNNI1; LY9; HTR6; FUT10; UMODL1; ABHD2; SEMA7A; NOX1; NKX6-1; SOCS5; CD4; SOCS7; BMP4; BARHL2; HAPLN2; IRX2; TNMD; CCR2; NR1I3; DDX5; SERPING1; RRAS2; PALLD; UBB; PENK; GRIP1; STC1; MYO15A; SOX10; RNF2; HTR5A; VDR; STMN1; TBX4; NRXN3; SYT4; OLFM4; CSF1; DPPA2; DPPA3; NOX5; DPPA5; SPRR1A; SPRR1B; PCDHB3; FLG; TGFA; CD79B; THBS2; IGSF10; SBDS; COL27A1; ADAM19; NR5A2; DLX4; PCDHB2; GPRC5B; DLX1; DLX2; DLX3; NXF5; ODF1; ACVRL1; C1QL4; IL4R; NXF2; ODF4; SDK2; RHOQ; TLR5; LMX1A; LMX1B; CMA1; RHOH; STAB2; SLC26A5; FGF9; FGF8; FGF7; EVPL; RHOA; MTNR1A; PLEK; BCAN; IL1RN; VTN; ETV2; ETV7; IVL; MPO; VAV2; G6PC; HOXD13; HOXD10; RGMA; POU4F3; SPOCK1; BATF2; EBF2; WWTR1; SOST; ACE; EPHA8; SLC32A1; NR2E3; NFATC2; EPHA1; LCE2B; ATP5J; RPS27A; SS18L1; KNDC1; CNR1; GBX2; FCER1G; UCP3; NANOG; CCDC63; LCE3D; LCE3E; PCDHB11; SGCA; PCDHB6; PHIP; SRD5A2; FGF10; TCF21; PDCD1; NKD1; TBXA2R; CECR2; MACF1; PCDHB15; ESRRB; PDILT; KRT6A; HMGA2; ZFP36L1; PAQR7; PCDHB5; UNC13D; CHRNA1; SERPINF2; ZAP70; GHSR; ANKS1A; HEY2; WFIKKN2; CCL11; CCL13; RASGRF1; CCL17; ADAMTS12; IFNA2; IFNA1; ID2; IFNA6; IFNA4; LEP; NGRN; CTGF; EMP2; PRM3; EMP1; HSP90AA1; PSMB2; HIVEP3; HMX2; OLFML3; RPS15; SP8; BMPER; CLPS; HAX1; TFF1; LATS2; RIPK3; LENEP; TDGF1; GPR55; GNAT1; DSCAM; ELF3; P2RX2; MSC; CPNE9; BCL2L11; TNFRSF1B; CAND1; PRAMEF5; SEMA6B; CPNE6; CPNE5; GLI2; WNT8A; CREB3L1; TG; MMD2; LDHA; HILS1; ZFP57; COL8A2; COL8A1; KCNJ10; ULK4; TNR; CARD11; CHRDL1; UMOD; TMPRSS6; FOXD3; TTLL7; AR; LUZP1; LRRC55; SSBP1; LCE1B; TNN; NPM1; FOXN1; NPM2; PCDH8; H1FOO; DBX1; PTPRS; GFI1; RPL38; RHCG; PCDH1; BBS2; BBS5; UNC45B; DSPP; GDNF; TSHZ1; PLAC8; ALOX15B; NOTO; FOLR1; TNFRSF1A |
| GO:0002520 | 2 | immune system development | 6,04E-06 | 2,42E-05 | HIST1H4B; HIST1H4A; ZNF160; HIST1H4F; HIST1H4E; HIST1H4K; GLRX5; HIST1H4I; ZFPM1; CD34; HIST1H4L; CASP10; EPO; B2M; IL27; NKX2-3; ATPIF1; AXL; NKX2-5; LTBR; CYP26B1; IL12RB1; PIK3CD; CBFA2T3; TBX21; CLEC1B; IFNA13; IRAK3; IFNA10; IFNA17; IFNA16; PRTN3; IFNA14; ERBB2; CD28; IL31RA; SOD1; TPO; ZNF683; LY9; BGLAP; CLCF1; FUT10; CSF3R; KIRREL3; SOCS5; BMP4; SLAMF1; IL20; C1QC; CSF1; RUNX2; RUNX3; IHH; DACT2; CCL19; FST; CD3D; MMP14; CCL3; CD1D; RASGRP1; HIPK2; RASGRP4; CCL5; DOCK2; IFNA7; EPB42; TSHR; LEP; LILRB4; LILRB3; LILRB2; LILRB1; SOX13; HOXA5; PPARGC1B; CEACAM1; SBDS; JAK3; MT1G; HLA-DOA; FOXJ1; HOXB8; FARP2; IL4R; BVES; HOXB3; HOXB7; HOXB4; RHOH; SHH; PLEK; PTPN6; WNT3A; ETV2; GNAS; VAV1; TYROBP; PRDM1; BATF2; LCK; CD74; ACP5; MFNG; ACE; BATF; RORC; ONECUT1; FOXE1; CD4; CCR1; DYRK3; TBX1; CCR4; POLB; SMPD3; CD79B; MEOX1; CD79A; CLEC5A; FCER1G; SPI1; ZBTB16; TNFSF8; C3; FGF10; TCF21; KDR; PDCD1; TGFBR3; TAL1; ITGA4; ZFP36L1; CLC; LGALS9; BARX1; DLL4; PLA2G2D; FOXL1; MIXL1; TNFRSF13B; SFRP2; IL2RA; ITK; PGLYRP2; IL10; IFNA2; IFNA1; MB; ID2; IFNA6; IFNA4; HOXA7; IL4; IFNA21; HOXA3; IL3; SLAMF6; BLNK; HOXA9; TGFB2; NCKAP1L; CDH17; HAX1; PLCG2; PGLYRP1; IL23R; GPR55; PITX2; SOX6; KLF13; HIST4H4; GFI1B; CEBPE; PDE1B; RPL22; GLI2; ZAP70; HCLS1; PRKCA; CARD11; CD40; EFNA2; IGHD; BCL2L11; MMP21; FOXN1; RIPK3; GFI1; LTA; CASP8; PURB; NFAM1; MMP9; CALCA; POU2F2; AICDA; BCL3 |
| GO:0031128 | 2 | developmental induction | 0,000516 | 0,001629 | WNT3A; BMP4; WNT3; WNT2; HOXA11; DKK1; HIPK2; SOX9; POU5F1; GDNF; FGF8; SALL1; FGF10; FGFR1 |
| GO:0048589 | 2 | developmental growth | 0,001192 | 0,003489 | TIMP3; MST1; CPNE5; COL27A1; RASAL1; AGT; CAV3; NKX2-5; LHX1; LHX2; FSTL4; ITSN2; DCC; SLC12A5; WWC1; FMOD; FGFR1; CDH4; WT1; SOD1; FLRT1; KLK6; GATA4; CXCL12; SEMA7A; NKX6-1; ADRA1A; BMP4; GH1; SYT2; SYT4; CSF1; PTEN; STC1; HELT; GHRHR; DUOX2; ADRB2; COLQ; TSHR; APOD; PRLR; PLAC8; SOX15; RAPH1; VIL1; SYT17; NACA; DPYSL2; BARHL2; PAX7; IGFBP1; FGF9; FGF8; SHH; GNAS; NOG; G6PC; HOXD13; NOV; FOXC2; WNT3A; GHRH; GHRL; FGF7; CCR2; CHAT; TBX5; RGMA; MYH6; PTK7; CDKL5; RPS6KB1; HOXA11; C3; FGF10; TGFBR3; MACF1; NLGN3; NDN; ATP8A2; COMP; EFNA5; HMGA2; ZFP36L1; SALL4; FOXL2; CHRNA1; GHSR; HEY2; YAP1; SFRP2; CCL11; SEMA3B; LRP6; WNT3; WNT2; MAEL; EMX1; LEP; HOXA5; CRHR2; NKD1; TGFB2; MYF6; LATS2; INSR; SOX9; MYOCD; SOX2; DSCAM; MYOG; CPNE9; BCL2L11; DMBX1; GRHL2; MT3; SEMA6B; CPNE6; GDF5; GLI2; WWTR1; TNR; SBDS; MTPN; POU4F3; STAT5A; CHST11; MUSTN1; BBS2; DRD3; SOX10; CHRND; SALL1; GJE1 |
| GO:0048513 | 3 | animal organ development | 8,89E-13 | 5,34E-11 | HIST1H4B; HIST1H4A; ZNF160; HIST1H4F; HIST1H4E; HIST1H4K; HIST1H4I; PDE6C; HIST1H4L; JPH2; HIPK2; B2M; PCSK9; AGT; FA2H; LHX1; OGDH; LHX4; LHX5; LHX6; TYROBP; NR0B2; SOX6; PIK3CD; PTGER3; ATPIF1; SLC6A17; IFNA13; IFNA10; IFNA17; IFNA16; IFNA14; LRP2; NPR2; GIP; ZNF683; DMP1; SP6; GPR37L1; RPGRIP1; MEG3; CSF3R; GATA4; CXCL12; CXCL14; ADRA1A; VCX2; PTEN; MAG; MMP14; CD1D; ACTA1; RASGRP4; RPS27A; ITGA4; MMP19; MYO18B; NACA; FARP2; CRYBA1; APOA2; CRYBA4; DNAI1; APOD; TNNT2; LILRB3; LILRB2; LILRB1; SOX10; SOX13; IL4; APOH; BVES; HRH2; H3F3B; REG3G; TTN; REG3A; FOXJ1; HOXB8; HOXB9; HOXB2; HOXB3; KCNQ4; HOXB1; HOXB6; HOXB7; DSP; HOXB5; SMTNL1; COL4A3; PPARA; ITGAX; MB; NOG; KRT17; KRT16; KRT13; PRDM1; LHX9; NOV; CRIP1; FOXC2; KRT19; MYH11; GHRH; MYH14; MYH15; GHRL; THRB; NPHS1; SLC4A5; SHH; DYRK3; POLB; ADCYAP1; FGF6; COL2A1; PADI2; MYF5; NCOA3; SPI1; STRC; NR2F2; ACAT1; ATOH1; S100A7; PARVA; S100A1; MYLK2; CHRDL2; BARX2; LTA; BARX1; OLIG3; YAP1; IL2RA; NDE1; EDA; PGLYRP2; EMX2; EMX1; VAV1; IFNA21; SSTR4; MYL6B; H2BFS; NCKAP1L; KCNC1; MYF6; PSMD6; EGFR; CAT; PLCG2; PGLYRP1; ERG; AHSG; MYOC; GIPR; MYOG; RS1; EIF2S2; FRG1; SIM2; GFI1B; PDE1B; GJB3; GJB2; GJB5; SULF1; KLF5; PROP1; NUMBL; ITGB4; LTBP1; DDX17; CRTAC1; SLC24A4; BFSP2; PALLD; BFSP1; MST1; PHOX2B; MMP21; STAT5A; HOXB13; SOX21; LRRC10; HAP1; KLF15; GLRA2; DKK4; DKK1; CHRND; NEFL; GJE1; SFTPD; WNT3A; TGM2; TGM3; COL11A2; ZFPM2; ZFPM1; GDF6; C5AR1; TNNC1; CHI3L1; MIP; DAB1; RPL22; LBP; ADAMTS7; GATA5; DHRS3; GNG12; EAF2; CLEC1B; CDH1; GDF2; CDH3; RP1L1; KNDC1; FGA; RAX; WT1; IL31RA; TPO; LDB2; ESX1; CLCF1; KLK4; KIRREL3; LELP1; GH1; RFX4; C1QC; C1QB; SFN; UBA52; GNG5; RHO; CCL3; GHRHR; SMPD3; CCL5; TBR1; MDGA1; IBSP; AMELX; LCE1F; MYCN; LCE1D; LCE1E; LCE1B; LCE1C; HRNR; LCE1A; IFNA7; ESRRB; CEACAM1; EN2; TACSTD2; MT1G; CLDN4; PLXDC1; DPYSL2; FZD1; OXT; SPRR4; SPRR3; SPDEF; EIF4E; CYP1B1; PHEX; ZBTB16; GJA8; MYBPC3; SCN5A; COX17; CD74; MFNG; CHAD; ONECUT1; FOXE1; FOXE3; CCDC40; RASGRP1; CHAT; TTBK1; MYH6; MYH7; RPS6KB1; TPM1; MMP20; TNFSF8; TBX19; KLHL10; CLIC5; IL6R; COL5A3; ASGR2; COL5A1; FOXL2; FOXL1; IGF2BP1; CXADR; KEL; ITK; NCOA1; IRS2; FGF16; HOXA7; SFRP2; HOXA5; HOXA3; HOXA2; SLAMF6; CRHR2; MUSTN1; HOXA9; ATP5F1; TGFBR3; TGFB2; PTK7; CYP17A1; SOX9; MYOCD; PITX2; PITX3; SMOC1; PITX1; ITGB1BP1; DMBX1; CEBPE; YWHAQ; FYN; ALX4; TNNI3; ABCB5; TNNI1; HCLS1; PHLDA2; DNAJC19; DHH; PRKCA; CARD11; MTSS1; EBF2; VCX; VCY; BDH1; CHST11; VAMP5; PFKFB1; AICDA; PRLR; MMP9; OTP; CALCA; CYP26B1; POU2F2; DGAT2; DYNLL1; CRLF1; TBX21; CD34; SOX15; IL20; PAX8; MYL2; PDPN; IL27; RAPGEF3; APCDD1; CAV3; AXL; CAV1; LTBR; PECAM1; CALM2; CALM1; IL12RB1; TNFRSF13B; RORC; DCC; CBFA2T3; INHBB; MARCKS; PRTN3; JAK3; ERBB2; ERBB3; AQP5; BMP8B; AQP1; CDH22; CDH23; EVX1; WNK4; HOXD3; BGLAP; CTSZ; FGFR1; NME2; DHODH; RUNX2; RUNX3; IHH; FOXA2; EDAR; CCL19; DLL4; CD3D; PAPPA2; DOCK2; GNAO1; CRX; DUOX2; EPB42; ALPK3; CCKAR; ITGA11; NKX2-3; TSHR; RGN; CCKBR; SLC23A1; SLC9A1; HOXA6; LIM2; CHODL; AFF2; PPARGC1B; RREB1; LBX1; FRZB; TSHZ1; PAX5; PAX4; PAX7; PAX6; HOXB4; PAX3; SPRR2E; SPRR2D; SPRR2G; SPRR2F; SPRR2A; PTPN6; SPRR2B; ANG; ESR2; GNAS; OTOF; SLAMF1; SLCO2B1; MIXL1; LCK; CD4; CCR1; KRT1; TBX1; FST; CDSN; TBX5; CRYGB; KRT8; CD79B; MEOX1; BCR; CD79A; LILRB4; WNT3; HOXA11; HOXA10; PSMA2; PSMA3; PSMA1; PSMA4; CASR; PSMA8; ALOX12B; KDR; LHX2; TAL1; COMP; EFNA2; SALL4; LGALS9; SALL1; PLA2G2D; SALL3; UCHL5; NEUROD2; CYP1A1; CYP1A2; LRP1; LRP6; LCE2D; IL10; WNT2; IL13; RARRES2; ADRB2; IL3; COL1A1; BLNK; ALOX12; CABP4; CDH17; CRISPLD2; ECM1; LOR; SOX2; INSR; IL23R; SOX3; LTA4H; LOX; KLF13; HIST4H4; GCNT1; GCNT3; HLA-DOA; GDF3; MT3; TFAP2D; GDF5; PPL; PHOSPHO1; OVOL2; NRG3; MBP; SPINK2; OTOP1; STAC3; POU3F4; OSR1; OSR2; MTPN; POU5F1; HPN; VCX3A; ANKRD2; DMRTA2; CASP8; DRD1; PURB; NFAM1; NDUFS4; WNT9B; COL6A3; BCL3; ALPL; LCE5A; ACP5; GLRX5; MC2R; CDX4; CASP10; EPO; CASP14; NKX2-2; APLP1; NKX2-6; NKX2-5; NKX6-2; MICAL2; WNT16; TLE2; EDN3; ZAP70; CD28; SOD1; LY9; HTR6; FUT10; UMODL1; SEMA7A; NKX6-1; SOCS5; SOCS7; BMP4; IRX2; CCR2; DDX5; TCHH; UBB; TLR5; STC1; MYO15A; RNF2; HTR5A; VDR; STMN1; TBX4; SYT4; CSF1; DPPA2; SPRR1A; SPRR1B; TNS3; FLG; TGFA; CRYGD; SBDS; COL27A1; ADAM19; NR5A2; NEUROG3; DACT2; DLX1; DLX2; DLX3; ACVRL1; IL4R; CLEC5A; SDK2; FOXB1; LMX1A; CMA1; RHOH; SLC26A5; FGF9; FGF8; FGF7; EVPL; RHOA; MTNR1A; PLEK; BCAN; ETV2; ETV7; IVL; HOXD13; HOXD10; AR; BATF2; WWTR1; ACE; BATF; SLC32A1; LCE2B; ATP5J; HYDIN; GBX2; FCER1G; LCE3D; LCE3E; SGCA; SRD5A2; FGF10; TCF21; NKD1; CECR2; ATP8A2; HMGA2; ZFP36L1; CHRNA1; GHSR; HEY2; CCL11; SEMA3B; NUP210L; ADAMTS12; IFNA2; IFNA1; ID2; IFNA6; IFNA4; LEP; CTGF; PSMB2; HIVEP3; HMX2; BMPER; HAX1; LATS2; RIPK3; CNTNAP2; TDGF1; GPR55; GNAT1; DSCAM; ELF3; P2RX2; MSC; BCL2L11; GRHL2; GRHL3; SEMA6B; GLI2; WNT8A; TG; LDHA; COL8A2; COL8A1; TNR; CHRDL1; UMOD; PTGIS; POU4F3; LUZP1; NR2E3; FOXN1; DBX1; PTPRS; GFI1; RPL38; BBS2; BBS5; UNC45B; DSPP; GDNF; ALOX15B; NOTO; FOLR1 |
| GO:0009888 | 3 | tissue development | 1,12E-11 | 5,14E-10 | JPH2; STRC; AGT; LHX1; LHX2; SOX6; IRX2; LCE2B; DMP1; EHF; MEG3; GATA4; GATA5; RPSA; TNMD; IL10; MMP14; ACTA1; RPS27A; ITGA4; MYO18B; ACVRL1; FERD3L; APOD; TNNT2; SOX10; DGAT2; SOX15; HRH2; TTN; REG3G; REG3A; FOXJ1; HOXB2; HOXB3; HOXB7; DSP; HOXB5; PPARA; HCK; NOG; KRT17; KRT16; KRT15; PRDM1; NOV; CRIP1; FOXC2; MYH11; MYH14; MYH15; GHRL; THRB; NPHS1; SLC4A5; EVPL; COL2A1; RGMA; NCOA3; NR2F2; ACAT1; ATOH1; S100A7; MYLK2; CHRDL2; BARX2; BARX1; YAP1; EDA; MSGN1; EMX1; MYL6B; MYF5; MYF6; PSMD6; EGFR; CAT; ERG; AHSG; MYOG; DDX17; GJB2; GJB5; SULF1; KLF5; ITGB3; ITGB2; ITGB4; BFSP2; PALLD; BFSP1; PHOX2B; MMP20; HOXB13; SOX21; LRRC10; CA9; UPK2; KLF15; DKK4; DKK1; CHRND; TGM2; TGM3; COL11A2; ZFPM2; IPMK; GSTA1; GSTA2; TNNC1; CHI3L1; ADAMTS7; CDH3; CDH5; WT1; IL31RA; LDB2; KLK4; KLK5; KLK6; KLK7; LELP1; CSF1; SFN; UBA52; EYA2; CCL3; LAMB3; IBSP; AMELX; HSBP1; LCE1F; MYCN; LCE1D; LCE1E; LCE1B; LCE1C; HRNR; PTEN; CEACAM1; DMBT1; TACSTD2; FZD1; BVES; SPRR4; SPRR3; IGFBP1; SHH; MYH6; PHEX; ZBTB16; GJA4; TCHH; CALML5; MYBPC3; CYP26B1; PROC; WNT3A; ONECUT1; FOXE1; FOXE3; CCDC40; FABP5; CYP1B1; MYH7; RPS6KB1; LCE1A; TPM1; FOXB1; TGFBR3; CLIC5; KRTAP5-9; VTN; ASGR2; COL5A1; FOXL2; FOXL1; BNC1; RAB25; CXADR; KEL; ACTL8; HOXA7; HOXA5; HOXA3; CRHR2; MUSTN1; HAPLN2; TGFB2; PTK7; GRSF1; ANXA4; SOX9; ALOX12; PITX2; PITX3; PITX1; ITGB1BP1; LCE5A; ALX4; TOR1A; TNNI3; TNNI1; SBDS; CES1; MTSS1; EBF2; BDH1; CHST11; VAMP5; MMP9; DLL4; LAMC2; CRLF1; CD34; LIN7C; MST1; IL20; MYL2; RAPGEF3; APCDD1; CAV3; CAV1; PECAM1; RORC; UPK1A; MARCKS; MARVELD2; RIPK4; COL17A1; ERBB3; ZFPM1; BMP8B; WNK4; HOXD3; BGLAP; CTSZ; FGFR1; NME2; RUNX2; RUNX3; IHH; FOXA2; EDAR; DUOX2; SLC9A1; PRLR; VIL1; SCNN1B; NKX2-5; FRZB; LBX1; PAX5; PAX7; PAX6; HOXB4; PAX3; SPRR2E; SPRR2D; SPRR2G; SPRR2F; SPRR2A; PAX8; SPRR2B; ESR2; GNAS; MIXL1; RPS7; CCR1; ADAMTS12; FST; TBX4; TBX5; CRYGB; CRYGD; MEOX1; DACT2; HOXA11; PSMA2; PSMA3; PSMA1; KRT6B; PSMA4; CASR; PSMA8; KDR; TAL1; COMP; SALL4; SALL1; SFRP2; CYP1A1; LRP6; SFRP4; LCE2D; WNT3; WNT2; IL13; ADRB2; COL1A1; MYOCD; SPDEF; TRIM71; ECM1; LOR; SOX2; INSR; LTA4H; GCNT1; GCNT3; GDF3; GDF2; GDF6; GDF5; PPL; PHOSPHO1; ITGAM; OTOP1; STAC3; OSR1; OSR2; MEST; MTPN; POU5F1; HPN; ANKRD2; DMRTA2; WNT9B; ALPL; TIMP3; TBX6; KLK14; CASP14; NKX2-2; NKX2-3; NKX2-6; RREB1; MICAL2; EDN3; SOD1; UMODL1; SEMA7A; NKX6-1; KRT3; BMP4; DDX5; TBX1; UBB; STC1; CDSN; VDR; KRT5; FA2H; DPPA2; SPRR1A; SPRR1B; FLG; COL27A1; NR5A2; NEUROG3; DLX2; DLX3; NACA; FGF9; FGF8; FGF7; FGF6; RHOA; ETV2; IVL; HOXD13; HOXD10; HYDIN; GBX2; NANOG; LCE3D; LCE3E; FGF10; TCF21; NKD1; CECR2; KRT6A; HMGA2; ZFP36L1; CHRNA1; GHSR; HEY2; CCL11; SEMA3B; NUP210L; WWTR1; ID2; LEP; CTGF; EMP1; PSMB2; HIVEP3; BMPER; LATS2; OVOL2; ELF3; P2RX2; MSC; PRKACB; GRHL1; GRHL2; GRHL3; SEMA6B; GLI2; WNT8A; COL8A1; UMOD; PTGIS; POU4F3; LUZP1; WNT16; FOXN1; PCDH8; PTPRS; RPL38; RHCG; BBS2; BBS5; DSPP; GDNF; ALOX15B; NOTO; FOLR1; TNFRSF1A |
| GO:0048731 | 3 | system development | 1,92E-10 | 6,28E-09 | PCDHB16; HIST1H4B; HIST1H4A; ZNF160; HIST1H4F; HIST1H4E; PCSK2; HIST1H4I; PDE6C; SCG2; HIST1H4L; JPH2; HIPK2; B2M; PCSK9; NUP210L; AGT; FA2H; LHX1; OGDH; LHX4; BLOC1S2; LHX6; TYROBP; NR0B2; SOX6; CYP46A1; PIK3CD; NPHS1; PTGER3; ATPIF1; HIST1H4K; GAL3ST1; SLC6A17; IFNA13; IRAK3; IFNA10; IFNA17; IFNA16; IFNA14; ARHGEF10; NEUROD2; NPR2; NPR3; SLITRK3; GIP; ZNF683; DMP1; SP6; GPR37L1; RPGRIP1; MEG3; CSF3R; GATA4; CXCL12; CXCL14; ADRA1A; COL15A1; LST1; VCX2; LECT2; PTEN; DACT2; PLXDC1; MAG; MAL; MMP14; CD1D; ACTA1; IL27; RASGRP4; LRRN3; RPS27A; ITGA4; MMP19; MYO18B; NACA; FARP2; CRYBA1; SERPINE1; APOA2; CRYBA4; DNAI1; APOD; TNNT2; LILRB3; LILRB2; LILRB1; DRP2; SOX13; SOX14; IL4; APOH; BVES; ZIC5; HRH2; H3F3B; GDF6; GTF2I; CCL24; TTN; GDF10; FOXJ1; HOXB8; HOXB9; FST; HOXB2; HOXB3; KCNQ4; HOXB1; HOXB6; HOXB7; DSP; HOXB5; SMTNL1; COL4A3; PPARA; LHX5; ITGAX; MB; NOG; KRT17; IL12RB1; KRT13; PRDM1; LHX9; NOV; CRIP1; FOXC2; KRT19; ACP5; GHRH; MYH14; MYH15; GHRL; THRB; KRT16; SLC4A5; SHH; DYRK3; POLB; ADCYAP1; FGF6; COL2A1; PADI2; MYF5; NCOA3; SPI1; TRIM71; STRC; NR2F2; PCDHB13; ACAT1; SH3GL3; S100A9; S100A8; ATOH1; S100A7; PARVA; S100A1; SRPX2; CCBE1; MYLK2; CHRDL2; BARX2; LTA; BARX1; TRPV4; PLA2G10; UBE2V2; OLIG3; YAP1; IL2RA; F2; EDA; PGLYRP2; EMX2; EMX1; VAV1; IFNA21; SSTR4; NDE1; MYL6B; H2BFS; NCKAP1L; KCNC1; MYF6; PSMD6; EGFR; CAT; PLCG2; PGLYRP1; ERG; AHSG; MYOC; GIPR; MYOG; RS1; EIF2S2; FRG1; SIM2; GFI1B; GJB1; PDE1B; GJB3; GJB2; GJB5; SULF1; CXCR3; KLF5; PROP1; CRYGD; NUMBL; NOS1; ITGB3; ITGB2; ITGB4; LTBP1; DDX17; CRTAC1; SLC24A4; BFSP2; CD40; BFSP1; MST1; IGHD; CAMK2B; PHOX2B; MMP21; STAT5A; TCHH; HOXB13; SOX21; ARSE; SNW1; HAP1; KLF15; PER2; GLRA2; DKK4; LCE5A; DKK1; RND1; CDH23; CHRND; GDF2; GJE1; ZNF396; SLC1A3; SFTPD; WNT3A; TGM2; TGM3; COL11A2; ZFPM2; SPTB; RGMA; C5AR1; TNNC1; CHI3L1; RASAL1; MIP; DAB1; RPL22; CLSTN2; LBP; ADAMTS7; GATA5; DHRS3; GNG12; SLC12A5; EAF2; CLEC1B; CDH1; CDH3; RP1L1; CDH5; CDH4; RAX; WT1; IL31RA; TPO; LDB2; ESX1; CLCF1; KLK3; KLK4; KLK6; KLK8; KIRREL3; HRG; LELP1; GH1; RFX4; SYT2; C1QC; C1QB; SFN; PLEKHA5; UBA52; GNG5; RHO; CCL3; GHRHR; NHLH1; CCL5; SERINC5; TBR1; COLQ; MDGA1; FERD3L; IBSP; AMELX; LCE1F; MYCN; LCE1D; LCE1E; IL20; LCE1C; HRNR; LCE1A; IFNA7; CEL; CEACAM1; EN2; LRRTM2; TACSTD2; MT1G; CLDN4; ANGPT4; DPYSL2; POU3F4; FZD1; OXT; SPRR4; PCDHA10; SPRR3; SPDEF; EIF4E; MYH6; PHEX; CLIC5; ZBTB16; GJA4; GJA8; MYBPC3; SCN5A; GAS7; COX17; CD74; SERF1A; MFNG; CHAD; ONECUT1; FOXE1; FOXE3; CCDC40; SERF1B; TRPC6; NHLH2; CHAT; KIF17; FLT4; FLT1; ZFPM1; TTBK1; CYP1B1; MYH7; IPMK; CDKL5; RPS6KB1; TPM1; MMP20; DOCK2; OLIG1; TNFSF8; TBX19; PRELP; KLHL10; NTF3; NLGN3; CLC; COL5A3; ASGR2; COL5A1; FOXL2; FOXL1; IGF2BP1; LRRC38; CXADR; KEL; ITK; NCOA1; IRS2; FGF16; HOXA7; SFRP2; HOXA5; OMP; HOXA3; HOXA2; ANKRD2; AMIGO1; SLAMF6; AMIGO2; CRHR2; NEFL; HOXA9; ATP5F1; TGFBR3; TGFB2; PTK7; STAB2; LGI4; CYP17A1; SOX9; ALOX12; PITX2; PITX3; PCDHA11; PITX1; ITGB1BP1; FZD10; LOXL1; DMBX1; CEBPE; CAMP; FYN; ALX4; SCN2B; TOR1A; TNNI3; ABCB5; TNNI1; HCLS1; PHLDA2; PACSIN1; DNAJC19; MUSTN1; CCKBR; MATN3; MATN2; DHH; PRKCA; CARD11; CHRM3; LRRC10; PRKCG; MTSS1; EBF2; VCX; VCY; IL6R; BDH1; CHST11; VAMP5; WFIKKN2; WFIKKN1; PFKFB1; GLDN; AICDA; PRLR; MMP9; OTP; CALCA; CYP26B1; CMKLR1; DGAT2; DYNLL1; CRLF1; TBX21; TNS3; CD34; SOX15; RAPGEF5; PAX8; MYL2; PDPN; RLN2; RAPGEF3; APCDD1; CAV3; AXL; CAV1; LTBR; PECAM1; CALM2; CALM1; CNGB1; TNFRSF13B; RORC; DCC; CBFA2T3; POU6F2; INHBB; MARCKS; FMOD; PCLO; PRTN3; JAK3; ERBB2; ERBB3; AQP5; BMP8B; AQP1; EXTL1; CDH22; SNTG2; EVX1; WNK4; HOXD3; FLRT1; BGLAP; CTSZ; REG3G; FGFR1; CXCL1; ADCY1; NME2; DHODH; RUNX2; RUNX3; IHH; FOXA2; EDAR; CCL19; DLL4; LRTM2; REG3A; CD3D; PAPPA2; HELT; ADORA1; GNAO1; CRX; DUOX2; EPB42; ALPK3; CCKAR; ITGA11; NPTX1; NKX2-3; TSHR; RGN; MARK4; SLC23A1; PCDHB9; SLC9A1; HOXA6; LIM2; CHODL; AFF2; PPARGC1B; PCDHB4; RAPH1; SYT17; RREB1; NPPB; LBX1; PDCL3; ADAMTS4; FRZB; TSHZ1; PAX5; PAX4; PAX7; PAX6; HOXB4; PAX3; SPRR2E; SPRR2D; SPRR2G; SPRR2F; SPRR2A; PTPN6; PTPN5; SPRR2B; ANG; ESR2; GNAS; OTOF; SMPD3; SLAMF1; SLCO2B1; MIXL1; HAPLN2; MOG; LCK; DPF3; CD4; CCR1; KRT1; CCR3; CCR4; HIF3A; CDSN; TBX5; BATF; CRYGB; KRT8; KCNA2; MEOX1; BCR; CD79A; LILRB4; WNT3; HOXA13; HOXA11; HOXA10; PSMA2; PSMA3; PSMA1; PSMA4; CASR; C3; PSMA8; ALOX12B; KDR; EPB41L3; LHX2; TAL1; NDN; COMP; EFNA5; EFNA2; SALL4; LGALS9; SALL1; PLA2G2D; SALL3; UCHL5; LRP2; CYP1A1; CYP1A2; LRP1; LRP6; LCE2D; IL10; WNT2; IL13; RARRES2; FGA; ADRB2; IL3; COL1A1; BLNK; MYOCD; CABP4; CDH17; CRISPLD2; RASGRP1; ECM2; ECM1; LOR; SOX2; INSR; IL23R; SOX3; LTA4H; LOX; SMOC1; KLF13; HIST4H4; GCNT1; GCNT3; HLA-DOA; GDF3; MT3; SPAG9; TFAP2D; GDF5; PPL; PHOSPHO1; OVOL2; NRG3; MBP; SPINK2; OTOP1; STAC3; HSPB1; OSR1; OSR2; MTPN; POU5F1; HPN; PCDHA2; PCDHA3; VCX3A; PCDHA7; PCDHA4; PCDHA5; DMRTA2; PCDHA8; DRD3; CASP8; DRD1; PURB; NFAM1; NDUFS4; WNT9B; COL6A3; BCL3; ALPL; YWHAQ; TIMP3; TIMP2; TBX6; MYH11; GLRX5; MC2R; CDX4; CASP10; EPO; CASP14; CCK; NKX2-2; APLP1; DYX1C1; NKX2-6; NKX2-5; HYDIN; NKX6-2; FSTL4; ITSN2; POU2F2; PTGIS; MICAL2; WNT16; TLE2; EDN3; ZAP70; CD28; SOD1; LY9; HTR6; FUT10; UMODL1; SEMA7A; NOX1; NKX6-1; SOCS5; RPS7; SOCS7; BMP4; BARHL2; IRX2; TNMD; CCR2; DDX5; TBX1; PALLD; UBB; PENK; GRIP1; STC1; MYO15A; SOX10; RNF2; HTR5A; VDR; STMN1; TBX4; NRXN3; SYT4; MACF1; CSF1; DPPA2; NOX5; SPRR1A; SPRR1B; PCDHB3; FLG; TGFA; CD79B; THBS2; SBDS; COL27A1; ADAM19; NR5A2; NEUROG3; PCDHB2; GPRC5B; DLX1; DLX2; DLX3; ACVRL1; IL4R; CLEC5A; SDK2; FOXB1; TLR5; LMX1A; LMX1B; CMA1; RHOH; SLC26A5; FGF9; FGF8; FGF7; EVPL; RHOA; MTNR1A; PLEK; BCAN; SFRP5; VTN; ETV2; ETV7; IVL; VAV2; HMOX1; HOXD13; HOXD10; POU4F3; SPOCK1; BATF2; WWTR1; CLDN11; ACE; EPHA8; SLC32A1; NR2E3; EPHA1; LCE2B; ATP5J; SS18L1; KNDC1; CNR1; GBX2; FCER1G; LCE3D; LCE3E; PCDHB11; SGCA; PCDHB6; SRD5A2; FGF10; TCF21; PDCD1; NKD1; TBXA2R; CECR2; PCDHB15; ESRRB; ATP8A2; HMGA2; ZFP36L1; PCDHB5; CHRNA1; SERPINF2; GHSR; ANKS1A; HEY2; CCL11; SEMA3B; RASGRF1; ADAMTS12; IFNA2; IFNA1; ID2; IFNA6; IFNA4; LEP; NGRN; CTGF; EMP2; PRKACB; HSP90AA1; PSMB2; HIVEP3; HMX2; BMPER; HAX1; LATS2; RIPK3; CNTNAP2; TDGF1; GPR55; GNAT1; DSCAM; ELF3; P2RX2; MSC; CPNE9; BCL2L11; GRHL2; GRHL3; SEMA6B; CPNE6; CPNE5; GLI2; WNT8A; TG; LDHA; COL8A2; COL8A1; KCNJ10; ULK4; TNR; CHRDL1; UMOD; TMPRSS6; TTLL7; AR; LUZP1; LRRC55; LCE1B; TNN; FOXN1; MMD2; DBX1; PTPRS; GFI1; RPL38; PCDH1; BBS2; BBS5; UNC45B; DSPP; GDNF; ALOX15B; NOTO; FOLR1 |
| GO:0007275 | 3 | multicellular organism development | 9,15E-10 | 2,35E-08 | PCDHB16; HIST1H4B; HIST1H4A; ZNF160; HIST1H4F; HIST1H4E; PCSK2; HIST1H4I; PDE6C; PCSK6; SCG2; HIST1H4L; JPH2; HIPK2; B2M; PCSK9; SOHLH2; NUP210L; AGT; FA2H; LHX1; OGDH; LHX4; BLOC1S2; LHX6; TYROBP; NR0B2; SOX6; CYP46A1; PIK3CD; NPHS1; PTGER3; ATPIF1; HIST1H4K; GAL3ST1; PARVA; LBX1; SLC6A17; IFNA13; IRAK3; IFNA10; IFNA17; IFNA16; IFNA14; ARHGEF10; NEUROD2; NPR2; NPR3; SLITRK3; GIP; ZNF683; DMP1; SP6; GPR37L1; EHF; NEUROG3; MEG3; CSF3R; GATA4; CXCL12; CXCL14; ADRA1A; COL15A1; ADRA1B; LST1; VCX2; LECT2; PTEN; DACT2; PLXDC1; MAG; MAK; MAL; MMP14; CD1D; ACTA1; IL27; RASGRP4; TDG; CLDN11; RPS27A; ITGA4; MMP19; TWIST2; MYO18B; NACA; SLC2A14; FARP2; CRYBA1; SERPINE1; APOA2; CRYBA4; DNAI1; APOD; TNNT2; LILRB3; LILRB2; LILRB1; DRP2; SOX13; SOX14; IL4; APOH; BVES; ZIC5; HRH2; JAK3; GDF6; GTF2I; CCL24; TTN; GDF10; FOXJ1; HOXB8; HOXB9; FST; HOXB2; HOXB3; KCNQ4; HOXB1; HOXB6; HOXB7; DSP; HOXB5; SMTNL1; HOXC12; COL4A3; PPARA; CHST2; LHX5; ITGAX; MB; NOG; KRT17; IL12RB1; KRT13; PRDM1; LHX9; NOV; CRIP1; FOXC2; KRT19; ACP5; GHRH; MYH14; MYH15; GHRL; THRB; KRT16; TNFRSF10C; SHH; DYRK3; POLB; ADCYAP1; FGF6; COL2A1; PADI2; MYF5; NCOA3; RPS4Y1; TRIM71; STRC; NR2F2; PCDHB13; ACAT1; SH3GL3; S100A9; S100A8; ATOH1; S100A7; GGN; S100A1; LAMA1; SRPX2; CCBE1; MYLK2; CHRDL2; BARX2; SPIC; LTA; BARX1; TRPV4; PLA2G10; UBE2V2; OLIG3; YAP1; IL2RA; F2; EDA; PGLYRP2; MSGN1; LMO2; EMX2; EMX1; VAV1; IFNA21; SSTR4; NDE1; MYL6B; H2BFS; NCKAP1L; KCNC1; MYF6; HNF4A; PSMD6; EGFR; CAT; PLCG2; PGLYRP1; ERG; AHSG; MYOC; GIPR; MYOG; RS1; EIF2S2; FRG1; SIM2; GFI1B; GJB1; PDE1B; GJB3; GJB2; GJB5; SULF1; CXCR3; KLF5; PROP1; CRYGD; H1FNT; APOL2; NUMBL; NOS1; ITGB3; ITGB2; ITGB4; LTBP1; DDX17; CRTAC1; SLC24A4; BFSP2; CD40; BFSP1; MST1; IGHD; CAMK2B; RPL29; PHOX2B; MMP21; STAT5A; TCHH; HOXB13; SOX21; ARSE; SNW1; HAP1; KLF15; PER2; GLRA2; DKK4; LCE5A; DKK1; CATSPER4; CDH23; ZNF541; CHRND; MT3; GJE1; ZNF396; SLC1A3; SFTPD; WNT3A; TGM2; TGM3; COL11A2; ZFPM2; DNAI2; SPTB; TFAP2D; C5AR1; TNNC1; CHI3L1; RASAL1; MIP; DAB1; RPL22; CLSTN2; LBP; ADAMTS7; GATA5; PIWIL1; ADAMTS9; DHRS3; GNG12; SLC12A5; EAF2; CLEC1B; CDH1; CDH3; RP1L1; CDH5; CDH4; RAX; WT1; IL31RA; TPO; LDB2; ESX1; CLCF1; KLK3; KLK4; KLK6; KLK8; KIRREL3; HRG; LELP1; GH1; RFX4; SYT2; C1QC; C1QB; SFN; PLEKHA5; UBA52; GNG5; MYCNOS; RHO; PLAGL2; SPRR4; CCL3; GHRHR; NHLH1; CCL5; SERINC5; TBR1; COLQ; MDGA1; FERD3L; LAMB3; IBSP; MESP2; AMELX; HSBP1; LCE1F; MYCN; LCE1D; LCE1E; NEK2; LCE1C; SCGB1A1; LCE1A; IFNA7; CEL; CLEC5A; CEACAM1; DMBT1; EN2; LRRTM2; TACSTD2; MT1G; CLDN4; ANGPT4; DPYSL2; POU3F4; FZD1; OXT; SPI1; PCDHA10; SPRR3; SPDEF; EIF4E; MYH6; PHEX; CLIC5; ZBTB16; GJA4; GJA8; SFRP5; MYBPC3; RPL7L1; SCN5A; PROC; GAS7; COX17; CD74; SERF1A; MFNG; CHAD; ONECUT1; RPGRIP1; FOXE1; FOXE3; CCDC40; SERF1B; TRPC6; NHLH2; CHAT; KIF17; PRM2; FLT4; TBPL2; MXD1; FLT1; ZFPM1; TTBK1; CYP1B1; MYH7; NOTCH2NL; IPMK; CDKL5; RPS6KB1; C1QBP; MMP20; IGF2BP1; DOCK2; OLIG1; TNFSF8; TBX19; PRELP; KLHL10; NTF3; NLGN3; POGK; CLC; COL5A3; ASGR2; COL5A1; FOXL2; FOXL1; RPS4X; UPK2; LRRC38; CXADR; KEL; ITK; NCOA1; IRS2; FGF16; HOXA7; SFRP2; HOXA5; HOXA4; HOXA3; HOXA2; ANKRD2; AMIGO1; SLAMF6; AMIGO2; CRHR2; NEFL; HOXA9; ATP5F1; TGFBR3; TGFB2; ACSBG2; PTK7; CECR1; GRSF1; LGI4; CYP17A1; SOX9; ALOX12; PITX2; PITX3; PCDHA11; PITX1; ITGB1BP1; FZD10; LOXL1; DRG1; DMBX1; CEBPE; RND1; CAMP; FYN; ALX4; SCN2B; TOR1A; TNNI3; ABCB5; TNNI1; HCLS1; PHLDA2; PACSIN1; DNAJC19; MUSTN1; CCKBR; MATN3; MATN2; DHH; PRKCA; CARD11; CHRM3; LRRC10; PRKCG; MTSS1; EBF3; EBF2; VCX; VCY; IL6R; PBX2; BDH1; CHST11; SYT17; VAMP5; WFIKKN2; WFIKKN1; PFKFB1; GLDN; TLX1; AICDA; PRLR; MMP9; OTP; CALCA; CYP26B1; CMKLR1; LRRN3; DGAT2; DYNLL1; CRLF1; TBX21; TNS3; CD34; SOX15; RAPGEF5; PAX8; MYL2; PDPN; RLN2; RAPGEF3; APCDD1; TXNDC2; CAV3; AXL; CAV1; LTBR; PECAM1; CALM2; CALM1; CNGB1; TNFRSF13B; GADD45B; RORC; DCC; CBFA2T3; POU6F2; INHBB; MARCKS; FMOD; PCLO; PRTN3; H3F3B; ERBB2; ERBB3; AQP5; BMP8B; AQP1; EXTL1; CDH22; SNTG2; EVX1; WNK4; HOXD3; FLRT1; BGLAP; CTSZ; REG3G; FGFR1; CXCL1; ADCY1; NME2; DHODH; RUNX2; RUNX3; IHH; FOXA2; EDAR; CCL19; DLL4; LRTM2; REG3A; CD3D; PAPPA2; HELT; PKP1; ADORA1; GNAO1; CRX; DUOX2; EPB42; ALPK3; CCKAR; TNFRSF17; VENTX; ITGA11; NPTX1; NKX2-3; TSHR; RGN; MARK4; SLC23A1; PCDHB9; SLC9A1; HOXA6; LIM2; CHODL; AFF2; PPARGC1B; PCDHB4; PLEKHB1; RAPH1; SLCO4C1; OMP; NFE2; PRM1; IL20; RREB1; NPPB; TSHZ1; PDCL3; ADAMTS4; FRZB; PRKACB; PAX5; PAX4; PAX7; PAX6; HOXB4; PAX3; SPRR2E; SPRR2D; SPRR2G; SPRR2F; SPRR2A; PTPN6; PTPN5; SPRR2B; HEMGN; ANG; ESR2; BTF3; NANOS2; OTOF; ATP8A2; SMPD3; SLAMF1; SLCO2B1; MIXL1; IL13; MOG; LCK; DPF3; THEG; CD4; CCR1; KRT1; CCR3; CCR4; HIF3A; CDSN; TBX5; BATF; CRYGB; KRT8; KCNA2; MEOX1; BCR; CD79A; LILRB4; WNT3; STAB2; HOXA13; HOXA11; HOXA10; PSMA2; PSMA3; PSMA1; PSMA4; CASR; C3; PSMA8; ALOX12B; KDR; EPB41L3; LHX2; TAL1; NDN; COMP; EFNA5; EFNA2; SALL4; LGALS9; SALL1; PLA2G2D; SALL3; UCHL5; TNP2; LRP2; CYP1A1; CYP1A2; LRP1; LRP6; SFRP4; LCE2D; IL10; WNT2; GNAS; RARRES2; MAEL; FGA; ADRB2; CNTNAP2; IL3; COL1A1; BLNK; MYOCD; CABP4; CDH17; CRISPLD2; RASGRP1; ECM2; ECM1; LOR; SOX2; INSR; IL23R; PAEP; SOX3; LTA4H; LOX; SMOC1; KLF13; HIST4H4; GCNT1; GCNT3; HLA-DOA; GDF3; GDF2; SPAG9; CATSPER1; GDF5; PPL; PHOSPHO1; OVOL2; NRG3; MBP; SPINK2; EYA2; OTOP1; RTL1; STAC3; HSPB1; OSR1; OSR2; MTPN; POU5F1; HPN; GRHL1; PCDHA2; PCDHA3; VCX3A; PCDHA7; PCDHA4; PCDHA5; DMRTA2; PCDHA8; DRD3; CASP8; DRD1; PURB; NFAM1; NDUFS4; WNT9B; COL6A3; BCL3; ALPL; YWHAQ; TIMP3; TIMP2; TBX6; MYH11; GLRX5; MC2R; CDX4; CASP10; EPO; CASP14; CCK; NKX2-2; APLP1; DYX1C1; NKX2-6; NKX2-5; HYDIN; PKD1L1; NKX6-2; FSTL4; ITSN2; POU2F2; PTGIS; MICAL2; WNT16; TLE2; EDN3; SLC4A5; TNP1; TDRD9; CD28; SOD1; TPM1; LY9; HTR6; FUT10; UMODL1; SEMA7A; NOX1; NKX6-1; SOCS5; RPS7; SOCS7; BMP4; BARHL2; HAPLN2; IRX2; TNMD; CCR2; DDX5; TBX1; PALLD; UBB; PENK; GRIP1; STC1; MYO15A; SOX10; RNF2; HTR5A; VDR; STMN1; TBX4; NRXN3; SYT4; MACF1; CSF1; DPPA2; DPPA3; NOX5; DPPA5; SPRR1A; SPRR1B; PCDHB3; FLG; TGFA; CD79B; THBS2; IGSF10; SBDS; COL27A1; ADAM19; NR5A2; DLX4; PCDHB2; GPRC5B; DLX1; DLX2; DLX3; NXF5; ODF1; ACVRL1; IL4R; NXF2; ODF4; SDK2; FOXB1; TLR5; LMX1A; LMX1B; CMA1; RHOH; SLC26A5; FGF9; FGF8; FGF7; EVPL; RHOA; MTNR1A; PLEK; BCAN; IL1RN; VTN; ETV2; ETV7; IVL; VAV2; HMOX1; HOXD13; HOXD10; RGMA; POU4F3; SPOCK1; BATF2; WWTR1; SOST; ACE; EPHA8; SLC32A1; NR2E3; EPHA1; LCE2B; ATP5J; SS18L1; KNDC1; CNR1; GBX2; FCER1G; NANOG; LCE3D; LCE3E; PCDHB11; SGCA; PCDHB6; SRD5A2; FGF10; TCF21; PDCD1; NKD1; TBXA2R; CECR2; OLFML3; PCDHB15; ESRRB; PDILT; HMGA2; ZFP36L1; PAQR7; PCDHB5; CHRNA1; SERPINF2; ZAP70; GHSR; ANKS1A; HEY2; CCL11; SEMA3B; RASGRF1; CCL17; ADAMTS12; IFNA2; IFNA1; ID2; IFNA6; IFNA4; LEP; NGRN; CTGF; EMP2; PRM3; EMP1; HSP90AA1; PSMB2; HIVEP3; HMX2; SP8; BMPER; CLPS; HAX1; LATS2; RIPK3; LENEP; TDGF1; GPR55; GNAT1; DSCAM; ELF3; P2RX2; MSC; CPNE9; BCL2L11; TNFRSF1B; GRHL2; GRHL3; SEMA6B; CPNE6; CPNE5; GLI2; WNT8A; CREB3L1; TG; MMD2; LDHA; HILS1; ZFP57; COL8A2; COL8A1; KCNJ10; ULK4; TNR; HRNR; CHRDL1; UMOD; TMPRSS6; FOXD3; TTLL7; AR; LUZP1; LRRC55; LCE1B; TNN; FOXN1; NPM2; PCDH8; DBX1; PTPRS; GFI1; RPL38; PCDH1; BBS2; BBS5; UNC45B; DSPP; GDNF; ALOX15B; NOTO; FOLR1; TNFRSF1A |
| GO:0070661 | 3 | leukocyte proliferation | 1,08E-08 | 2,23E-07 | CD38; SFTPD; EPO; VTCN1; IL27; IL12RB1; ZP4; XCL1; IFNA13; IFNA10; IFNA17; IFNA16; IFNA14; ERBB2; HLA-DPB1; NPR3; HLA-DRB1; CLCF1; BMP4; LST1; CD6; HHLA2; IHH; CCL19; CD300A; CD1D; TNFSF14; DOCK2; CSF1; CD209; ICOSLG; LILRB2; LILRB1; SLC11A1; JAK3; FOXJ1; EPHB6; SHH; SCGB1A1; PTPN6; TACR1; PRDM1; AIF1; CD74; ACE; BTN3A1; NFATC2; CD4; CCR2; CD79A; CCL5; FGF10; CLC; LGALS9; PLA2G2D; PLA2G2F; CD180; TNFRSF13B; IL2RA; IL10; IFNA2; IFNA1; IL13; IFNA7; IFNA6; IRS2; IFNA4; LEP; IL4; IFNA21; IL3; SLAMF6; EBI3; SLAMF1; NCKAP1L; CD244; RIPK3; CD28; IL23R; FYN; WNT3A; ZAP70; MNDA; RAC2; CARD11; CD40; PYDC1; IGHD; CLEC4G |
| GO:0021536 | 3 | diencephalon development | 4,23E-07 | 6,92E-06 | GHRHR; GHRH; KCNC1; DUOX2; SOX2; CNTNAP2; ADCYAP1; PITX2; SOX3; PITX1; NKX2-6; GBX2; OGDH; NCOA1; GLI2; PROP1; CDH1; SRD5A2; FGF10; RAX; FOXB1; HMGA2; HAP1; PAX6; SALL1; TBX19; FGF8; SHH; LRP6; BMP4; ESR2; NOG; OTP; CRHR2 |
| GO:0048534 | 3 | hematopoietic or lymphoid organ development | 1,51E-06 | 2,07E-05 | HIST1H4B; HIST1H4A; ZNF160; HIST1H4F; HIST1H4E; HIST1H4K; GLRX5; HIST1H4I; ZFPM1; CD34; HIST1H4L; CASP10; EPO; B2M; IL27; NKX2-3; ATPIF1; AXL; NKX2-5; LTBR; CYP26B1; IL12RB1; PIK3CD; CBFA2T3; TBX21; CLEC1B; IFNA13; IFNA10; IFNA17; IFNA16; PRTN3; IFNA14; ERBB2; CD28; IL31RA; SOD1; TPO; ZNF683; LY9; BGLAP; CLCF1; FUT10; CSF3R; KIRREL3; SOCS5; BMP4; SLAMF1; IL20; C1QC; CSF1; RUNX2; RUNX3; IHH; DACT2; CCL19; CD3D; MMP14; CCL3; CD1D; RASGRP1; HIPK2; RASGRP4; CCL5; DOCK2; IFNA7; ITGA4; TSHR; HOXA7; LILRB4; LILRB3; LILRB2; LILRB1; SOX13; HOXA5; PPARGC1B; CEACAM1; SBDS; JAK3; MT1G; HLA-DOA; FOXJ1; HOXB8; FARP2; IL4R; BVES; HOXB3; HOXB7; HOXB4; RHOH; SHH; PLEK; PTPN6; WNT3A; ETV2; GNAS; VAV1; TYROBP; PRDM1; BATF2; LCK; CD74; ACP5; MFNG; ACE; BATF; RORC; ONECUT1; FOXE1; CD4; CCR1; DYRK3; TBX1; FST; POLB; SMPD3; CD79B; MEOX1; CD79A; CLEC5A; FCER1G; SPI1; ZBTB16; TNFSF8; FGF10; TCF21; KDR; TGFBR3; TAL1; EPB42; ZFP36L1; LTA; BARX1; DLL4; PLA2G2D; FOXL1; MIXL1; TNFRSF13B; SFRP2; IL2RA; ITK; PGLYRP2; IL10; IFNA2; IFNA1; MB; ID2; IFNA6; IFNA4; LEP; IL4; IFNA21; HOXA3; IL3; SLAMF6; BLNK; HOXA9; TGFB2; NCKAP1L; CDH17; HAX1; PLCG2; PGLYRP1; IL23R; GPR55; PITX2; SOX6; KLF13; HIST4H4; GFI1B; CEBPE; PDE1B; RPL22; GLI2; ZAP70; HCLS1; PRKCA; CARD11; EFNA2; BCL2L11; MMP21; FOXN1; RIPK3; GFI1; LGALS9; CASP8; PURB; NFAM1; MMP9; CALCA; POU2F2; AICDA; BCL3 |
| GO:0050673 | 3 | epithelial cell proliferation | 1,63E-05 | 0,000184 | CD34; SCG2; MST1; TNMD; C5AR1; SULF1; ATPIF1; RREB1; EAF2; MARVELD3; CDH3; ERBB2; EHF; KLK8; CXCL12; NKX6-1; BMP4; NME2; SFN; PTEN; IHH; TBX1; REG3A; MMP14; VDR; CCL5; COL8A1; GLUL; NOX5; RGN; TGFA; RUNX3; HOXA5; APOH; CEACAM1; TACSTD2; REG3G; CAV1; CCL24; ANGPT4; CCL26; ACVRL1; PDCL3; FGF7; PAX6; FGF9; WFDC1; TACR1; ANG; ESR2; NOG; HMOX1; NOV; SCN5A; WNT3A; GHRL; FOXE3; SHH; CCR3; FLT4; NR2F2; FGF10; FGF16; KDR; TGFBR3; DLL4; YAP1; SFRP2; CCL11; WNT2; ID2; LEP; STAT5A; LACRT; BMPER; TGFB2; SLURP1; EGFR; ECM1; SOX9; OVOL2; SOX2; ITGB1BP1; GDF2; GDF5; CXCR3; KLF9; COL8A2; ITGB3; PRKCA; OSR1; OSR2; MTSS1; HPN; CALCA; FOXN1; WNT16 |
| GO:0048869 | 3 | cellular developmental process | 1,64E-05 | 0,000184 | HIST1H4B; HIST1H4A; PCSK9; HIST1H4F; HIST1H4E; HIST1H4K; HIST1H4I; PARVA; HIST1H4L; HIPK2; B2M; SOHLH2; NUP210L; AGT; LHX1; OGDH; LHX4; BLOC1S2; LHX6; TYROBP; LHX9; SOX6; PIK3CD; NPHS1; PTGER3; ATPIF1; IFNA13; IFNA10; IFNA17; IFNA16; IFNA14; ARHGEF10; SFRP2; NPR2; SLITRK3; GPR37L1; ZNF683; CRTAC1; CAMK2B; EHF; RPGRIP1; MEG3; OPA3; CSF3R; GATA4; CXCL12; CXCL14; COL15A1; SFRP4; LST1; TNMD; STAC3; SFRP5; FKBP6; PTEN; DACT2; MAG; CTBP2; MAL; MMP14; CD1D; ACTA1; RASGRP4; TNFSF14; C1GALT1C1; ITGA4; MMP19; MYO18B; NACA; SLC2A14; FARP2; FZD10; SERPINE1; APOD; LILRB4; LILRB3; LILRB2; LILRB1; SOX10; SOX13; SOX14; IL4; ZIC5; HRH2; JAK3; REG3G; CCL24; GDF10; FOXJ1; HOXB8; FST; HOXB3; HOXB7; DSP; HOXB5; BLK; PPARA; HCK; LHX5; MB; NOG; KRT17; PDE6C; PRDM1; NOV; FLNC; FOXC2; KRT19; HIST1H2BA; MYH11; MYH14; GHRL; THRB; KRT16; UPK1A; CARD11; SHH; DYRK3; DLK1; ADCYAP1; FGF6; COL2A1; GPRC5B; NCOA3; SPI1; NR2F2; S100A9; S100A8; ATOH1; S100A7; GGN; MYLK2; CHRDL2; BARX2; SPIC; LTA; BARX1; IL12RB1; UBE2V2; OLIG3; YAP1; IL2RA; F2; EDA; PGLYRP2; MSGN1; EMX2; EMX1; VAV1; IFNA21; HSPE1; NDE1; LMOD1; NCKAP1L; MYF5; MYF6; EGFR; CAT; PLCG2; PGLYRP1; ERG; LRRC10; MYOC; MYOG; SIM2; PDE1B; TWIST2; GJB5; SULF1; KLF5; PROP1; H1FNT; NUMBL; NOS1; ITGB3; ITGB2; ITGB4; DDX17; BFSP2; PALLD; BFSP1; PHOX2B; MMP21; HOXB13; SOX21; SNW1; HAP1; RAB25; PER2; MUSTN1; DKK1; CATSPER4; ZNF541; GDF2; PTCHD3; GJE1; SLC1A3; WNT3A; TGM3; COL11A2; ZFPM2; SPTB; GSTA1; GSTA2; GDF6; RASAL1; DAB1; RPL22; ADAMTS7; GATA5; IQCF1; ADAMTS9; TIAL1; FGR; SLC12A5; CLEC1B; CDH1; CDH3; RP1L1; CDH5; CDH4; WT1; IFITM1; MT3; IL31RA; SLCO4C1; CLCF1; KLK6; KLK8; KIRREL3; LELP1; SYT2; SLC2A4; C1QC; CSF1; SFN; ELK4; EYA2; CCL3; GHRHR; SMPD3; CCL5; TBR1; MDGA1; LAMB3; IBSP; AMELX; HSBP1; LCE1F; MYCN; LCE1D; NEK5; LCE1B; LCE1C; HRNR; LCE1A; IFNA7; CLEC5A; CEACAM1; DMBT1; EN2; CEACAM5; LCE1E; TACSTD2; MT1G; DPYSL2; LGALS12; BVES; SPRR4; SPRR3; OSR1; SPDEF; EIF4E; MAK; PLA2G10; CYP26B1; PROC; GAS7; TRIM6; CD74; MFNG; PIWIL1; ONECUT1; FOXE3; KIF17; TRPC6; NHLH2; NHLH1; FZD1; FLT1; TTBK1; MYH6; ZBTB16; NOTCH2NL; CDKL5; RPS6KB1; C1QBP; OLIG1; TNFSF8; TBX19; PRELP; KLHL10; NTF3; NLGN3; FGD3; FGD2; IL6R; COL5A1; FOXL2; FOXB1; UPK2; TTC26; LRRC38; CXADR; KEL; ITK; NCOA1; ACTL8; HOXA7; HOXA5; OMP; RPS3A; HOXA2; ANKRD2; AMIGO1; SLAMF6; CRHR2; HOXA9; HAPLN2; TGFBR3; TGFB2; ACSBG2; PTK7; LGI4; CYP17A1; SOX9; MYOCD; PITX2; PITX3; SMOC1; PITX1; ITGB1BP1; FERD3L; CEBPE; RND1; LCE5A; FYN; ATP8A2; TOR1A; TNNI3; ABCB5; AURKC; HCLS1; USH1C; PACSIN1; MATN2; DHH; PRKCA; CDC42EP1; CES1; MTSS1; EBF2; FFAR2; VTN; CHST11; VAMP5; WFIKKN2; WFIKKN1; AICDA; PRAMEF4; PRAMEF5; PRAMEF6; PRAMEF7; PRAMEF1; PRAMEF2; MMP9; OTP; CALCA; CMTM5; POU2F2; TBX21; CD34; CD36; SOX15; IL20; MYL2; PDPN; IL27; RAPGEF3; APCDD1; TXNDC2; CAV3; AXL; CAV1; LTBR; PRAMEF8; CNGB1; TNFRSF13B; GADD45B; RORC; DCC; CBFA2T3; POU6F2; INHBB; MARCKS; INHBE; FMOD; MARVELD2; TRPV4; PRTN3; TTN; ERBB2; ERBB3; ZFPM1; CETP; BMP8B; CDH23; EVX1; HOXD3; FLRT1; BGLAP; SEMA3B; SETD3; FGFR1; ADCY1; NME2; PRAMEF10; RUNX2; RUNX3; IHH; FOXA2; EDAR; CCL19; DLL4; LRTM2; REG3A; CD3D; HELT; DOCK2; GNAO1; CRX; RRAS2; EPB42; SDK2; CCKAR; ITGA11; NPTX1; NKX2-3; TSHR; TTLL7; SLC9A1; GLDN; PRLR; CHODL; PPARGC1B; PLEKHB1; RAPH1; VIL1; SYT17; EMP2; ANXA13; RREB1; FRZB; LBX1; PAX5; PAX4; PAX7; PAX6; HOXB4; PAX3; SPRR2E; SPRR2D; SPRR2G; SPRR2F; SPRR2A; PTPN6; PTPN5; SPRR2B; HEMGN; ANG; ESR2; GNAS; NANOS2; SMYD3; STEAP4; SLAMF1; STRC; FGF22; LCK; THEG; DMRTC2; KRT3; CCR1; CCR2; TBX1; CCR4; CDSN; TBX5; CRYGB; KRT8; CD79B; MEOX1; CD79A; WNT3; BCAP29; HOXA11; PAX8; KRT6A; PENK; KDR; EPB41L3; LHX2; TAL1; FBXO40; NDN; EFNA5; EFNA2; PLA2G2A; LGALS9; SALL1; PLA2G2D; SALL3; TNP2; FSCN3; NEUROD2; CYP1A1; LRP1; LRP6; RPSA; LCE2D; IL10; WNT2; IL13; RARRES2; MAEL; FGA; ADRB2; IL3; COL1A1; BLNK; CALR3; ALOX12; CABP4; CDH15; CDH17; RASGRP1; ECM2; LOR; SOX2; INSR; RUNX1T1; IL23R; SOX3; LTA4H; KLF13; HIST4H4; WASF1; KLF15; HLA-DOA; GDF3; NEFL; SPAG9; CATSPER1; GDF5; PPL; OVOL2; NRG3; MBP; SPINK2; ITGAM; RPS7; ANXA4; POU3F4; SPANXB1; DKKL1; OSR2; MTPN; POU5F1; HPN; GRHL2; DMRTA2; DRD3; CASP8; DRD1; PURB; NFAM1; WNT9B; CLIC5; BCL3; ALPL; TMOD4; TIMP2; TBX6; ACP5; CDX4; CASP10; EPO; CASP14; CCK; NKX2-2; S100A10; DYX1C1; NKX2-6; NKX2-5; HYDIN; NKX6-2; FSTL4; ITSN2; ADAMTS20; WNT16; EDN3; RNASEL; SLC4A5; TNP1; TDRD9; CD28; SPAG8; SOD1; TPM1; LY9; ABHD2; SEMA7A; NKX6-1; SOCS5; CD4; SOCS7; BMP4; BARHL2; NR1I3; DDX5; TCHH; UBB; GRIP1; STC1; RNF2; BATF; VDR; STMN1; NRXN3; SYT4; OLFM4; FA2H; SPRR1A; SPRR1B; FLG; CRYGD; IGSF10; COL27A1; NR5A2; NEUROG3; RGMA; DLX1; DLX2; DLX3; ODF1; ACVRL1; C1QL4; IL4R; ODF4; RHOQ; LMX1A; LMX1B; RHOH; SLC26A5; FGF9; FGF8; MMD2; EVPL; RHOA; PLEK; ETV2; ETV7; IVL; HNF4A; HOXD10; AR; SPOCK1; BATF2; WWTR1; ACE; EPHA8; NR2E3; NFATC2; EPHA1; LCE2B; SS18L1; KNDC1; CNR1; GBX2; FCER1G; NANOG; CCDC63; LCE3D; LCE3E; PHIP; SRD5A2; FGF10; TCF21; NKD1; CECR2; MACF1; PDILT; HMGA2; ZFP36L1; PAQR7; UNC13D; SERPINF2; ZAP70; MIXL1; ANKS1A; HEY2; CCL11; CCL13; RASGRF1; CCL17; ADAMTS12; IFNA2; IFNA1; ID2; IFNA6; IFNA4; LEP; NGRN; CTGF; PRM1; PRM3; PRM2; HSP90AA1; ZNF135; HIVEP3; HMX2; RPS15; BMPER; HAX1; TFF1; LATS2; RIPK3; CNTNAP2; TDGF1; GPR55; GNAT1; DSCAM; ELF3; P2RX2; CPNE9; CAND1; SEMA6B; CPNE6; CPNE5; GLI2; WNT8A; HILS1; COL8A1; KCNJ10; ULK4; TNR; CHRDL1; POU4F3; LRRC55; SSBP1; TNN; NPM1; FOXN1; NPM2; H1FOO; DBX1; PTPRS; GFI1; RHCG; CMKLR1; BBS2; UNC45B; CREB3L1; GDNF; PLAC8; ALOX15B; FOLR1; TNFRSF1A |
| GO:0001568 | 3 | blood vessel development | 1,75E-05 | 0,000194 | HIF3A; ZFPM2; CD34; CDX4; HIPK2; C5AR1; CHI3L1; RAPGEF3; AGT; GATA4; CAV1; PECAM1; SRPX2; CDH5; TNNI3; ERBB2; SFRP2; ANG; SCG2; ESX1; KLK3; MEG3; NKX2-5; HRG; FGFR1; COL15A1; BMP4; TNMD; NRXN3; CCR2; PTEN; TBX1; PLXDC1; MMP14; RLN2; CCL5; COL8A1; NOX1; MYO18B; NOX5; PARVA; SERPINE1; TGFA; HOXA7; APOD; ATPIF1; THBS2; HOXA5; APOH; CEACAM1; HOXA3; GTF2I; NPPB; CCL24; ANGPT4; DLX3; ACVRL1; PDCL3; HOXB3; CMA1; PAX6; COL4A3; PTGIS; FGF9; FGF8; SHH; FGF6; RHOA; ETV2; GJA4; VAV2; HMOX1; PRDM1; NOV; FOXC2; GHRL; EPHA1; KRT1; CCR3; TBX4; TBX5; FLT4; FLT1; GBX2; SPI1; CYP1B1; STAB2; NR2F2; C3; S100A7; FGF10; TCF21; KDR; TBXA2R; TAL1; CCBE1; ZFP36L1; MMP19; COL5A1; DLL4; SERPINF2; HEY2; YAP1; LRP2; CCL11; LRP1; WNT2; OVOL2; FGF16; LEP; CTGF; EMP2; COL1A1; MYOCD; TGFB2; PTK7; WT1; BMPER; ECM1; ALOX12; PITX2; LOX; ITGB1BP1; LOXL1; GDF2; CAMP; SULF1; CXCR3; KLF5; PROP1; TDGF1; ADRB2; COL8A2; ITGB3; ITGB2; LTBP1; HSPB1; PRKCA; OSR1; TMPRSS6; LUZP1; MMP21; FOXN1; HOXB13; AQP1; FOLR1 |
| GO:0048568 | 3 | embryonic organ development | 2,05E-05 | 0,00022 | ZFPM2; ZFPM1; CDX4; HIPK2; NKX2-6; NKX2-5; LHX1; MICAL2; LBX1; SOD1; TPO; HOXD3; ESX1; GATA4; FGFR1; BMP4; RUNX2; IHH; MYO15A; MMP14; ID2; TSHR; MYCN; ESRRB; FRZB; EN2; DLX2; HOXA2; HOXB8; HOXB1; HOXB2; HOXB3; KCNQ4; TSHZ1; HOXB6; HOXB7; HOXB4; HOXB5; PAX5; PAX6; FGF9; FGF8; SHH; PAX8; GNAS; NOG; HOXD10; PRDM1; STRC; FOXC2; KRT19; WNT3A; SALL1; FOXE1; CCDC40; TBX1; KRT8; COL2A1; BCR; GBX2; NCOA1; HOXA11; NR2F2; ATOH1; FGF10; TCF21; KDR; CLIC5; TAL1; ATP8A2; ZFP36L1; FOXL2; HEY2; YAP1; IL10; WNT2; RARRES2; HOXA7; HOXA6; HOXA5; HOXA3; IL3; TGFB2; HMX2; MYF5; PTK7; EGFR; SOX9; OVOL2; PITX2; GRHL2; GRHL3; GDF3; GJB5; ALX4; GLI2; POU3F4; OSR1; OSR2; POU4F3; HPN; CHST11; RPL38; BBS5; CASP8; WNT9B; WNT16; NOTO; FOLR1 |
| GO:0050793 | 3 | regulation of developmental process | 0,000132 | 0,001205 | HIST1H4B; HIST1H4A; HIST1H4F; HIST1H4E; HIST1H4K; HIST1H4I; HIST1H4L; JPH2; HIPK2; B2M; AGT; LHX1; LHX5; TYROBP; SOX6; ZNF706; PTGER3; IRAK3; NEUROD2; NPR2; SLITRK3; SP6; MEG3; CSF3R; GATA4; CXCL12; CXCL14; LST1; TNMD; CARD11; PTEN; MAG; MMP14; RASGRP1; IL27; TNFSF14; LRRN3; RPS27A; ACVRL1; FERD3L; SERPINE1; PHLDA2; LILRB4; LILRB3; LILRB2; LILRB1; SOX10; SOX13; SOX14; SOX15; APOH; OXT; JAK3; REG3G; GTF2I; CCL24; REG3A; FOXJ1; HOXB8; HOXB3; HOXB7; HOXB4; COL4A3; PPARA; HCK; NOG; KRT17; PRDM1; NOV; FOXC2; SYT4; GHRH; MYH14; GHRL; SHH; ADCYAP1; GPRC5B; NCOA3; NCOA1; ATOH1; PARVA; LAMA1; SRPX2; CCBE1; LTA; ITGB1BP1; UBE2V2; YAP1; IL2RA; F2; IL4; EMX1; NCKAP1L; MYF5; MYF6; HNF4A; PSMD6; PGLYRP2; PGLYRP1; ERG; AHSG; MYOC; MYOG; DDX17; TWIST2; SULF1; CXCR3; KLF5; NUMBL; NOS1; ITGB3; ITGB2; CD40; CAMK2B; PHOX2B; MMP20; SNW1; HAP1; PER2; DKK4; DKK1; GDF2; CMTM5; WNT3A; ZFPM2; ZFPM1; C5AR1; CHI3L1; RASAL1; DAB1; CLSTN2; ADAMTS7; ADAMTS9; FGR; EAF2; CDH3; CDH5; CDH4; WT1; IFITM1; MT3; CLCF1; KLK3; KLK6; KLK8; HRG; GH1; RFX4; SYT2; C1QC; CSF1; SFN; UBA52; GNG5; CCL3; GHRHR; CCL5; TBR1; COLQ; AMELX; MYCN; NEK5; CEACAM1; CEACAM5; LRRTM2; TACSTD2; ANGPT4; DPYSL2; FZD1; BVES; OSR1; EIF4E; CYP1B1; PLA2G10; CYP26B1; PROC; TRIM6; CD74; CHAD; FOXE3; TRPC6; C1QBP; FLT4; FLT1; TTBK1; MYH6; ZBTB16; CDKL5; RPS6KB1; TPM1; TBX19; NTF3; NLGN3; FGD3; FGD2; CLC; COL5A1; DLL4; KEL; HOXA7; HOXA5; HOXA2; AMIGO1; AMIGO2; HOXA9; TGFB2; PTK7; SOX9; ALOX12; SOX2; SOX3; SMOC1; SEMA6B; CAMP; FYN; ZAP70; HCLS1; PACSIN1; PRKCA; CDC42EP1; MMD2; IL6R; PRAMEF4; PRAMEF5; PRAMEF6; PRAMEF7; PRAMEF1; PRAMEF2; OTP; CALCA; CMKLR1; TBX21; CD34; CD36; MST1; IL20; PDPN; RLN2; RAPGEF3; CAV3; AXL; CAV1; IL12RB1; RORC; DCC; MARCKS; FMOD; TRPV4; ERBB2; CETP; AQP1; HOXD3; FLRT1; BGLAP; CCL13; SETD3; FGFR1; NME2; DHODH; PRAMEF10; RUNX2; RUNX3; IHH; FOXA2; IFNA2; LRTM2; GDF10; CRX; EPB42; TSHR; RGN; IL10; PRLR; CHODL; STAT5A; PPARGC1B; PLEKHB1; VIL1; SYT17; NKX2-5; NPPB; PDCL3; FRZB; LBX1; HEMGN; PAX4; PAX7; PAX6; PTPN6; PTPN5; GNAS; ZNF135; MIXL1; WWC1; CCR1; KRT1; CCR3; FST; TBX5; BCR; HOXA11; PAX8; PSMA2; PSMA3; PSMA1; PSMA4; C3; PSMA8; KDR; EPB41L3; TAL1; EFNA5; PLA2G2A; LGALS9; SFRP2; GPR37L1; LRP1; LRP6; SFRP4; SFRP5; WNT3; WNT2; IL13; RARRES2; MAEL; FGA; ADRB2; IL3; COL1A1; MYOCD; SPDEF; CDH15; ECM1; INSR; RUNX1T1; IL23R; KLF13; HIST4H4; HLA-DOA; GDF3; NEFL; SOD1; GDF6; GDF5; PHOSPHO1; NRG3; MBP; HSPB1; DKKL1; OSR2; MTPN; POU5F1; HPN; TMBIM1; ANKRD2; DMRTA2; DRD3; CASP8; PURB; NFAM1; WNT9B; TIMP2; EPO; NKX2-2; S100A10; RREB1; NKX6-2; FSTL4; ITSN2; EDN3; CD28; SPAG9; UMODL1; ADAMTS20; SEMA7A; NKX6-1; SOCS5; BMP4; BARHL2; CCR2; DDX5; TBX1; UBB; TBX6; VDR; NRXN3; OLFM4; PLAC8; THBS2; NEUROG3; RGMA; DLX1; DLX2; NACA; C1QL4; IL4R; RHOQ; LMX1A; CMA1; SPI1; SLC26A5; FGF9; FGF8; FGF7; RHOA; IL1RN; ETV2; HMOX1; HOXD13; SPOCK1; SOST; ACE; NFATC2; EPHA1; SS18L1; KNDC1; CNR1; LGALS12; NANOG; PHIP; FGF10; FGF16; PDCD1; NKD1; TBXA2R; MACF1; ATP8A2; HMGA2; UNC13D; SERPINF2; GHSR; HEY2; CCL11; SEMA3B; CCL17; ADAMTS12; CCL19; ID2; LEP; CTGF; EMP2; PRKACB; PSMB2; HAX1; LATS2; OVOL2; GPR55; DSCAM; CPNE9; BCL2L11; GRHL2; GRHL3; CPNE6; CPNE5; GLI2; WWTR1; TG; ULK4; TNR; PTGIS; AR; FOXN1; H1FOO; GFI1; PRAMEF8; BBS2; GDNF; ALOX15B; TNFRSF1A |
| GO:0030900 | 3 | forebrain development | 0,000232 | 0,002061 | IGF2BP1; DAB1; NKX2-6; AXL; LHX1; OGDH; LHX5; LHX6; GNG12; PTGER3; CDH1; RAX; AQP1; CRTAC1; HTR6; BMP4; KIRREL3; CXCL12; SEMA7A; FGFR1; SOCS7; FUT10; RFX4; PTEN; HTR5A; GHRHR; GNAO1; DUOX2; TBR1; TNR; NEUROG3; DLX1; DLX2; DPYSL2; LMX1A; LHX2; PAX5; PAX6; FGF8; SHH; BCAN; ESR2; NOG; SCN5A; WNT3A; GHRH; SLC32A1; CCR2; ADCYAP1; GBX2; NCOA1; NR2F2; ATOH1; SRD5A2; FGF10; HMGA2; EFNA2; SALL1; SALL3; TBX19; UCHL5; PTPRS; LRP2; NDE1; LRP1; LRP6; ID2; CCKAR; EMX1; SSTR4; CRHR2; KCNC1; EGFR; CYP17A1; CNTNAP2; PITX2; SOX3; PITX1; NEFL; FYN; GLI2; SOX2; PROP1; NRG3; NUMBL; POU3F4; FOXB1; DKK1; HAP1; DMRTA2; BBS2; DRD1; OTP; EMX2 |
| GO:0030901 | 3 | midbrain development | 0,000285 | 0,002445 | UCHL5; WNT3A; DYNLL1; ATP5J; PITX3; FZD1; CALM2; CALM1; YWHAQ; TFAP2D; EN2; FOXB1; S100A1; LDHA; H2BFS; LMX1A; CMA1; PADI2; FGF9; SHH; RHOA; TTBK1; FGFR1; SFRP2; LRP6; RFX4; WNT2; DKK1; MAG; MBP; ATP5F1 |
| GO:0021854 | 3 | hypothalamus development | 0,000656 | 0,005135 | NCOA1; ESR2; NKX2-6; FOXB1; HAP1; OTP; PROP1; SOX3; PITX2; SRD5A2; CRHR2; RAX |
| GO:0003205 | 3 | cardiac chamber development | 0,000909 | 0,006612 | PTK7; ZFPM2; ZFPM1; TBX1; MYL2; MYOCD; TBX5; PITX2; CAV3; NKX2-5; FZD1; TNNT2; MYH6; MYH7; GRHL2; DHRS3; TPM1; TNNI3; TNNI1; OVOL2; PARVA; TGFBR3; SFRP2; NACA; LTBP1; DSP; SALL4; SALL1; LUZP1; FGF8; GATA4; HEY2; LRP2; BMP4; WNT2; ID2; MYBPC3; PRDM1; DLL4; SCN5A; FOXC2; TNNC1 |
| GO:0035295 | 3 | tube development | 0,001559 | 0,010301 | SFTPD; TGM2; CRLF1; ZFPM2; IPMK; MST1; CHI3L1; NKX2-2; NKX2-3; DAB1; AGT; NKX2-6; CAV3; NKX2-5; LHX1; LHX2; THRB; MICAL2; IRX2; WT1; CYP1A1; WNK4; TNS3; CTSZ; GATA4; GATA5; FGFR1; BMP4; CSF1; IHH; ADAMTS12; EDAR; MMP14; VDR; ID2; DPPA2; FERD3L; CCKBR; MYCN; SOX10; CTGF; PDPN; HRH2; TACSTD2; DACT2; FOXJ1; ACVRL1; FZD1; LBX1; HOXB7; PAX7; PAX6; FGF9; FGF8; FGF7; RHOA; PAX8; NOG; HOXD13; PRDM1; FOXC2; WNT3A; FOXE1; CCDC40; RPS7; SHH; TBX1; TBX4; TBX5; RGMA; GBX2; TRIM71; EIF4E; HOXA11; ACAT1; CASR; FOXB1; FGF10; TCF21; CECR2; ZFP36L1; SALL4; SALL1; FOXL1; MIXL1; YAP1; SFRP2; CCL11; CYP1A2; EDA; LRP6; SFRP5; WWTR1; WNT2; IL13; RARRES2; SLC23A1; HOXA5; PRKACB; OVOL2; TGFB2; SPDEF; PTK7; CRISPLD2; BMPER; EGFR; CAT; PITX2; SOX9; MYOCD; LTA4H; LOX; ITGB1BP1; SIM2; BCL2L11; GRHL2; GRHL3; GDF2; ALX4; GLI2; KLF5; SPINK2; GDNF; ITGB3; ITGB4; UMOD; OSR1; MTSS1; LUZP1; BBS5; WNT9B; DLL4; NOTO; FOLR1 |
| GO:0007420 | 3 | brain development | 0,002279 | 0,014391 | DYNLL1; IGF2BP1; C5AR1; RAPGEF3; NKX2-2; DAB1; NKX2-6; AXL; LHX1; OGDH; CALM2; LHX5; LHX6; CALM1; GNG12; PTGER3; MARCKS; SLC6A17; CDH1; KNDC1; TTBK1; H2BFS; NEUROD2; AQP1; CDH22; CRTAC1; HTR6; FUT10; KIRREL3; CXCL12; SEMA7A; FGFR1; SOCS7; BMP4; VCX2; RFX4; SYT4; PTEN; MAG; HTR5A; GHRHR; STMN1; GNAO1; DUOX2; TBR1; MDGA1; APOD; TNR; AFF2; EN2; H3F3B; DRD1; NEUROG3; DLX1; DLX2; SSTR4; FOXJ1; DPYSL2; FZD1; HOXB2; HOXB3; HOXB1; LMX1A; LHX2; PAX5; CMA1; PAX6; FGF9; FGF8; SHH; RHOA; BCAN; ESR2; NOG; SCN5A; COX17; WNT3A; GHRH; LRP2; SLC32A1; ATP5J; CCR2; ADCYAP1; RAX; BCR; HYDIN; GBX2; NCOA1; NR2F2; ACAT1; ATOH1; SRD5A2; FGF10; S100A1; HMGA2; EFNA2; SALL1; SALL3; TBX19; UCHL5; HAP1; SFRP2; GPR37L1; NDE1; LRP1; LRP6; WNT2; ID2; CCKAR; IRS2; EMX1; SLC23A1; HOXA2; CRHR2; ATP5F1; HMX2; KCNC1; EGFR; CYP17A1; CNTNAP2; SOX3; PITX2; PITX3; PITX1; BCL2L11; DMBX1; GRHL2; MT3; YWHAQ; FYN; TFAP2D; GLI2; SOX2; PROP1; NRG3; MBP; LDHA; NUMBL; POU3F4; FOXB1; MTPN; PADI2; VCX; VCY; PHOX2B; BDH1; VCX3A; PTPRS; DMRTA2; BBS2; DKK1; NDUFS4; NEFL; OTP; EMX2 |
| GO:0048736 | 3 | appendage development | 0,002893 | 0,017502 | SALL3; SOX9; GDF5; TBX4; TBX5; PITX2; SMOC1; PITX1; COL2A1; MYCN; BCL2L11; ZBTB16; GRHL2; HOXA11; ALX4; GLI2; HOXA10; FGF10; RAX; SP8; CHST11; SALL4; ITGB4; COMP; OSR1; OSR2; NR2F2; SALL1; FGF9; FGF8; SHH; PBX2; FOXN1; FGFR1; SFRP2; BMP4; GNAS; WNT3; NOG; HOXD13; HOXD10; DKK1; RUNX2; IHH; CYP26B1; HOXA9 |
| GO:0021546 | 3 | rhombomere development | 0,003061 | 0,018215 | HOXB2; GBX2; HOXB1; HOXA2; HOXB3 |
| GO:0060322 | 3 | head development | 0,003888 | 0,022743 | DYNLL1; IGF2BP1; C5AR1; RAPGEF3; NKX2-2; DAB1; NKX2-6; AXL; LHX1; OGDH; CALM2; LHX5; LHX6; CALM1; GNG12; PTGER3; MARCKS; SLC6A17; CDH1; KNDC1; TTBK1; H2BFS; NEUROD2; AQP1; CDH22; CRTAC1; HTR6; FUT10; KIRREL3; CXCL12; SEMA7A; FGFR1; SOCS7; BMP4; VCX2; RFX4; SYT4; PTEN; MAG; HTR5A; GHRHR; STMN1; GNAO1; DUOX2; TBR1; MDGA1; APOD; TNR; AFF2; EN2; H3F3B; DRD1; NEUROG3; DLX1; DLX2; SSTR4; FOXJ1; DPYSL2; FZD1; HOXB2; HOXB3; HOXB1; LMX1A; LHX2; PAX5; CMA1; PAX6; FGF9; FGF8; SHH; RHOA; BCAN; ESR2; NOG; SCN5A; COX17; WNT3A; GHRH; LRP2; SLC32A1; ATP5J; CCR2; TBX1; ADCYAP1; RAX; BCR; HYDIN; GBX2; NCOA1; NR2F2; ACAT1; ATOH1; SRD5A2; FGF10; S100A1; HMGA2; EFNA2; SALL1; SALL3; TBX19; UCHL5; HAP1; SFRP2; GPR37L1; NDE1; LRP1; LRP6; WNT3; WNT2; ID2; CCKAR; IRS2; EMX1; SLC23A1; HOXA2; COL1A1; CRHR2; ATP5F1; TGFB2; HMX2; KCNC1; CRISPLD2; EGFR; CYP17A1; CNTNAP2; SOX3; PITX2; PITX3; PITX1; BCL2L11; DMBX1; GRHL2; MT3; YWHAQ; FYN; TFAP2D; GLI2; SOX2; PROP1; NRG3; MBP; LDHA; NUMBL; POU3F4; FOXB1; OSR2; MTPN; PADI2; VCX; VCY; PHOX2B; BDH1; VCX3A; PTPRS; DMRTA2; BBS2; DKK1; NDUFS4; NEFL; OTP; EMX2 |
| GO:0060023 | 3 | soft palate development | 0,004741 | 0,026876 | TSHZ1; TBX1; FOXE1; COL11A2 |
| GO:0021537 | 3 | telencephalon development | 0,007439 | 0,039716 | UCHL5; HTR5A; ID2; SLC32A1; EGFR; CYP17A1; CNTNAP2; KIRREL3; NDE1; DAB1; DRD1; LHX1; OGDH; NCOA1; LHX5; LHX6; NEFL; TNR; GNG12; IGF2BP1; DMRTA2; ATOH1; SRD5A2; NRG3; DLX1; DLX2; NUMBL; LHX2; DPYSL2; AQP1; CRTAC1; FOXB1; LMX1A; EFNA2; HTR6; LRP1; SALL1; SALL3; FGF8; SHH; PTPRS; CXCL12; SEMA7A; FUT10; WNT3A; FGFR1; BCAN; PAX5; SOCS7; BMP4; LRP6; RFX4; BBS2; EMX2; EMX1; PTEN; PAX6; SCN5A |
| GO:0021516 | 3 | dorsal spinal cord development | 0,007881 | 0,041418 | WNT3A; LHX1; HOXB8; TAL1; LHX5; RFX4; LBX1; PAX7; MDGA1 |
| GO:0048732 | 3 | gland development | 0,007949 | 0,041473 | TGM2; PCSK9; MST1; RAPGEF3; NKX2-3; CAV3; RREB1; LBP; EAF2; CDH1; FGA; WT1; SOD1; HOXD3; NKX2-5; FGFR1; BMP4; DHODH; FA2H; CCL11; PTEN; HOXB13; GHRHR; VDR; DUOX2; ADRB2; CSF1; RGN; TGFA; PRLR; STAT5A; CEACAM1; HRH2; CAV1; HOXB9; EDAR; FRZB; HOXB3; PAX6; FGF8; SHH; PAX8; ESR2; NOG; HOXD13; SLCO2B1; CRIP1; WNT3A; GHRH; ONECUT1; FOXE1; CCDC40; FGF7; TBX1; POLB; ADCYAP1; NCOA1; ACAT1; TBX19; FGF10; TCF21; TGFBR3; HMGA2; SALL1; FOXB1; CYP1A1; EDA; WNT3; WNT2; ID2; RIPK3; IRS2; HOXA5; HOXA3; HOXA9; TGFB2; EGFR; CYP17A1; INSR; SOX9; TDGF1; PITX2; SOX3; ELF3; PITX1; BCL2L11; SULF1; GLI2; SOX2; PROP1; TG; NRG3; CCKBR; AR; HPN; FOXN1; BDH1; SOX10; OTP; ALOX15B |
| GO:0048857 | 3 | neural nucleus development | 0,008077 | 0,041814 | H2BFS; MAG; YWHAQ; CALM2; KCNC1; CALM1; HOXB2; S100A1; HOXB1; ATP5J; PADI2; FGF9; KIRREL3; PITX2; DYNLL1; RHOA; PHOX2B; MBP; LDHA; TTBK1; ATP5F1 |
| GO:0048565 | 3 | digestive tract development | 0,008189 | 0,041814 | TGFB2; RARRES2; FOXE1; CCDC40; NKX2-2; MYOCD; PITX2; NKX2-3; DAB1; NKX2-6; MIXL1; CCKBR; SOX10; ALX4; GLI2; KLF5; HRH2; FGF10; TCF21; ITGB4; EGFR; SALL1; FGF9; FOXL1; SHH; GATA4; GATA5; SFRP2; CYP1A1; BMP4; SFRP5; SPDEF; ID2; HOXD13; HOXA5; PRDM1; OVOL2 |
| GO:0021984 | 3 | adenohypophysis development | 0,010976 | 0,054128 | GHRHR; GHRH; DUOX2; SOX2; PROP1; FGF8; PITX2 |
| GO:0007423 | 3 | sensory organ development | 0,017013 | 0,07524 | HIPK2; CABP4; MIP; NKX2-6; LHX1; LHX2; RP1L1; RAX; WT1; AQP5; SOD1; CDH23; CXCL14; FGFR1; BMP4; C1QB; RHO; DLL4; MYO15A; DUOX2; TSHR; CRYBA1; CRYBA4; MYCN; LIM2; COL2A1; CYP26B1; RPGRIP1; SDK2; ACVRL1; FRZB; KCNQ4; TSHZ1; PAX4; PAX6; SLC26A5; FGF9; FGF8; SHH; MTNR1A; PAX8; GJA8; NOG; OTOF; PDE6C; KRT13; PRDM1; STRC; FOXC2; WNT3A; SALL1; MYH15; FOXE3; SLC4A5; TBX1; CRYGB; CRYGD; BCR; GBX2; CYP1B1; ATOH1; FGF10; NKD1; CECR2; CLIC5; ATP8A2; CHRDL1; COL5A1; FOXL2; HEY2; CYP1A1; LRP6; WNT2; IL4; HOXA2; TGFB2; HMX2; MYF5; PTK7; EGFR; GNAT1; SOX9; BMPER; DSCAM; PITX2; PITX3; SMOC1; RS1; BCL2L11; GRHL2; GRHL3; GDF3; GLI2; SOX2; ABCB5; COL8A2; COL8A1; POU3F4; BFSP2; OSR1; OSR2; BFSP1; SOX3; POU4F3; HPN; NR2E3; PHOX2B; RPL38; UNC45B; WNT9B; WNT16; GJE1 |
| GO:0021517 | 3 | ventral spinal cord development | 0,017033 | 0,07524 | LHX1; DBX1; LHX4; LBX1; EVX1; HOXD10; GLI2; SHH; PAX6; DLL4; NKX2-2; DAB1; NKX6-2; OLIG3; NKX6-1 |
| GO:0061061 | 3 | muscle structure development | 0,019644 | 0,083692 | TMOD4; ZFPM2; ZFPM1; SMYD3; JPH2; MYL2; TNNC1; AGT; NKX2-6; CAV3; NKX2-5; MYH15; SOX6; WT1; SPAG9; MEG3; GATA4; CXCL14; FGFR1; BMP4; DDX5; MMP14; NFATC2; ACTA1; TNFSF14; ADRB2; MYO18B; ITGA11; TNNT2; SLC9A1; CHODL; SOX15; CYP26B1; CEACAM5; TTN; CAV1; NACA; IL4R; BVES; LBX1; DSP; PAX5; PAX7; PAX3; FGF8; SHH; FGF6; SMTNL1; LMOD1; NOG; HOXD10; MYBPC3; NOV; FLNC; FOXC2; KRT19; WNT3A; MYH11; MYH14; FGF9; NPHS1; TBX1; TPM1; TBX5; KRT8; FZD1; MYH6; MYH7; RPS6KB1; SGCA; NR2F2; FGF10; TCF21; TGFBR3; FBXO40; MYLK2; BARX2; FOXL2; CHRNA1; HEY2; CXADR; KEL; CCL17; WNT2; IL4; MYL6B; CRHR2; HIVEP3; NKD1; CDH15; MYF5; MYF6; UNC45B; SOX9; MYOCD; PITX2; PITX1; MYOG; FRG1; DDX17; P2RX2; ANKRD2; GDF3; MSC; ALX4; WNT8A; KLF5; TNNI3; TNNI1; NOS1; STAC3; MTPN; NEK5; PHOX2B; LRRC10; VAMP5; WFIKKN2; WFIKKN1; DMRTA2; DKK1; SETD3; CHRND; DLL4; COL6A3; CMTM5 |
| GO:0021543 | 3 | pallium development | 0,026134 | 0,102821 | WNT3A; HTR5A; SLC32A1; EGFR; CYP17A1; CNTNAP2; KIRREL3; NDE1; DAB1; DRD1; OGDH; NCOA1; LHX5; LHX6; NEFL; GNG12; ATOH1; SRD5A2; DLX1; DLX2; LMX1A; LHX2; PAX5; PAX6; FGF8; IGF2BP1; PTPRS; FUT10; FGFR1; BCAN; HTR6; SOCS7; LRP1; LRP6; DMRTA2; BBS2; EMX2; EMX1; PTEN |
| GO:0090102 | 3 | cochlea development | 0,026755 | 0,104694 | CECR2; PTK7; POU3F4; FRZB; GRHL3; OTOF; GLI2; SOX9; HPN; TBX1; TSHR; HEY2; SLC26A5 |
| GO:0001942 | 3 | hair follicle development | 0,027058 | 0,10474 | TGFB2; TGM3; FOXE1; FGF7; SOX9; FST; APCDD1; LHX2; ALX4; CDH3; FGF10; EGFR; LDB2; SHH; FOXN1; SOX21; EDA; GNAS; DKK4; DKK1; KRT17; EDAR |
| GO:0060560 | 3 | developmental growth involved in morphogenesis | 0,02783 | 0,105811 | WNT3A; ITSN2; PTK7; MACF1; HOXD13; CCR2; SOX9; RASAL1; DSCAM; SEMA6B; RGMA; LHX1; LHX2; CDKL5; MT3; FSTL4; TNR; DCC; CPNE5; RAPH1; SEMA3B; FMOD; FGF10; CDH4; SYT17; NKD1; DPYSL2; NLGN3; NDN; CPNE6; EFNA5; CPNE9; FLRT1; MST1; POU4F3; SALL1; SHH; CXCL12; SEMA7A; NKX6-1; SFRP2; CCL11; BMP4; LRP6; BARHL2; WNT3; SYT2; SYT4; CSF1; EMX1; YAP1 |
| GO:0021794 | 3 | thalamus development | 0,029089 | 0,106938 | GBX2; OGDH; LRP6; CNTNAP2; SHH; FOXB1 |
| GO:0030324 | 3 | lung development | 0,031296 | 0,113805 | MMP14; SFTPD; NOG; TBX4; PTK7; CRISPLD2; ZFPM2; THRB; CCDC40; DPPA2; PITX2; SOX9; MYOCD; CHI3L1; TBX5; LTA4H; LOX; FZD1; MYCN; GRHL2; EIF4E; HOXA5; PDPN; GLI2; TNS3; YAP1; FGF10; TCF21; FOXJ1; SIM2; CTSZ; FGF9; FGF8; FGF7; FGFR1; CYP1A2; BMP4; WNT2; IL13; SHH; SPDEF; SLC23A1; CTGF |
| GO:0032835 | 3 | glomerulus development | 0,031629 | 0,114435 | FOXJ1; BMP4; LHX1; KLF15; PECAM1; WWTR1; NOG; CD34; OSR1; SULF1; TCF21; MTSS1; COL4A3; WT1; NPHS1; KIRREL3; FOXC2 |
| GO:0072006 | 3 | nephron development | 0,032391 | 0,11539 | WT1; NPHS1; CD34; SOX9; SULF1; AGT; PECAM1; KLF15; HOXA11; ACAT1; TACSTD2; TCF21; WNT9B; FOXJ1; LHX1; UMOD; HOXB7; WNK4; OSR1; MTSS1; SALL1; FGF8; SHH; KIRREL3; PAX8; YAP1; BMP4; WWTR1; NOG; GDNF; COL4A3; IRX2; FOXC2 |
| GO:0061034 | 3 | olfactory bulb mitral cell layer development | 0,033335 | 0,11539 | OGDH; SALL1 |
| GO:0061378 | 3 | corpora quadrigemina development | 0,033335 | 0,11539 | FOXB1; TFAP2D |
| GO:0061379 | 3 | inferior colliculus development | 0,033335 | 0,11539 | FOXB1; TFAP2D |
| GO:0061323 | 3 | cell proliferation involved in heart morphogenesis | 0,041164 | 0,134487 | NACA; GNG5; BMP4; TBX5; PITX2; SOX9 |
| GO:0035988 | 3 | chondrocyte proliferation | 0,041164 | 0,134487 | MMP14; MUSTN1; HMGA2; CTGF; STC1; NOV |
| GO:0030323 | 3 | respiratory tube development | 0,043519 | 0,139882 | MMP14; SFTPD; NOG; TBX4; PTK7; CRISPLD2; ZFPM2; THRB; CCDC40; DPPA2; PITX2; SOX9; MYOCD; CHI3L1; TBX5; LTA4H; LOX; FZD1; MYCN; GRHL2; EIF4E; HOXA5; PDPN; GLI2; TNS3; YAP1; FGF10; TCF21; FOXJ1; SIM2; CTSZ; FGF9; FGF8; FGF7; FGFR1; CYP1A2; BMP4; WNT2; IL13; SHH; SPDEF; SLC23A1; CTGF |
| GO:0003228 | 3 | atrial cardiac muscle tissue development | 0,045403 | 0,14401 | PITX2; WNT2; MYH6 |
| GO:0048729 | 3 | tissue morphogenesis | 0,000315 | 0,002637 | TGM2; TGM3; ZFPM2; IPMK; LIN7C; KLK14; MST1; MYL2; TNNC1; AGT; CAV3; RREB1; LHX1; LHX2; MICAL2; TTN; WT1; ZFPM1; WNK4; CTSZ; GATA4; FGFR1; BMP4; CSF1; WNT3; IHH; ADAMTS12; UBB; DLL4; STC1; MMP14; ACTA1; RPS27A; MYCN; TNNT2; TRIM71; SOX10; UBA52; CEACAM1; FRZB; SCNN1B; TACSTD2; NKX2-5; ACVRL1; FZD1; HOXB2; LBX1; HOXB7; DSP; PAX7; HOXB4; FGF8; SHH; RHOA; PAX8; MYH7; ETV2; NOG; HOXD13; KRT17; KRT16; MYBPC3; FOXC2; WNT3A; FOXE1; CCDC40; RPS7; FGF7; TBX1; TBX6; TPM1; TBX4; TBX5; RGMA; GBX2; MYH6; PTK7; WNT9B; HOXA11; PSMA2; PSMA3; PSMA1; PSMA4; CASR; PSMA8; FGF10; TCF21; TGFBR3; CECR2; TAL1; KRT6A; HMGA2; SALL4; COL5A1; SALL1; FOXL1; HEY2; YAP1; SFRP2; CCL11; LRP6; IL10; WNT2; HOXA5; PRKACB; IRX2; PSMB2; RIPK4; NKD1; TGFB2; MYF5; MYF6; GRSF1; PSMD6; EGFR; SOX9; OVOL2; PITX2; GCNT1; GCNT3; GRHL3; GDF2; SULF1; GLI2; TOR1A; TNNI3; TNNI1; EYA2; ITGB3; ITGB4; MYLK2; OSR1; MTSS1; VDR; LUZP1; FOXN1; HOXB13; PCDH8; CA9; GRHL2; BBS5; DKK1; GDNF; WNT16; NOTO; FOLR1 |

**Supplementary Table S2.** Biological Processes among enriched pathways that were downregulated in Elk-1-VP16-transfected SH-SY5Y cells. GO: Gene ontology, R-HAS: Reactome, path:has: KEGG term. (Levels 2 and 3 are included in the Table)

| **GO ID** | **Term Level** | **Term Name** | **P-value** | **Q-value** | **Members** |
| --- | --- | --- | --- | --- | --- |
| GO:0044767 | 2 | single-organism developmental process | 1,26E-07 | 7,85E-07 | SMARCC2; PGAP1; ASXL2; RNF10; TUBD1; AP1M1; FKBP4; CD8A; ARFRP1; ANP32B; MFAP2; L1CAM; DBN1; TYK2; LEMD2; SEMA4F; SEMA4G; DLG3; DLG2; PALMD; CAMK1; CDC42SE2; APBB1; TAZ; SEMA6A; DMPK; RNF111; THY1; IRAK1; DHX9; ZNF689; TCOF1; DIAPH2; CAMK2D; ARHGEF19; MUC1; CAMK2G; MTCH1; SLC45A3; CRB2; APRT; SMARCD3; ATF5; SMARCD1; OPA1; ZFYVE27; GATA2; GBA2; AKAP6; PPP1R9B; DBNL; TRIOBP; WDTC1; CCND1; MAX; LITAF; ARHGAP12; FMNL3; CTBP1; LRP8; TNFSF13; CAPZB; MMP16; EVL; ELAVL1; ACVR1B; ALDH3A2; NOVA1; ITGA3; POLR1B; PUM1; RAD1; SFXN1; UROD; SEMA4B; TCF3; GAP43; SOX12; DLD; GLG1; NF2; ZNF513; WDR74; NR1H2; PDLIM7; DNASE1L2; KCNQ2; MRAS; CDK5RAP2; NFASC; SDF4; COL4A2; VEGFB; GRN; WDR77; CHST3; GDI1; PRKD2; ARNT2; PLXNC1; POLR2E; RPS6KA2; CSNK1A1; VASH1; TNFSF12; MTHFD1L; PSAP; PSMD13; CHD8; PDE6B; FRAT2; GPX4; FLOT2; MYBBP1A; IL11RA; HES1; FLNB; EIF4A1; CRIP2; ACP6; DNMT1; SLC26A6; ACP2; STIM1; TLE3; DEAF1; SMAD6; TK1; SMAD1; RET; SRGAP2; SMO; TNFRSF10B; POLE; PRKCSH; FOXM1; AKAP13; SLC4A2; POLM; DCTN1; TSC2; EFHC1; HTRA2; TSC1; HMBS; MDK; SEZ6L2; NR2F6; PCDHB14; ARF6; ELAVL3; DIP2A; IGF2R; BRWD3; AP3D1; BSG; ATOH8; PTPRU; FBXO31; LAMA4; LAMA5; CTF1; MECP2; NASP; MCAM; EIF4ENIF1; OPRM1; SUPT6H; FAIM2; MARCH5; F3; COL18A1; APBA3; APBA2; RIC8A; AK2; FAM20C; TBCC; BPNT1; ABT1; CXXC4; MME; CDK5RAP3; CDK5RAP1; SLC38A3; NBEAL2; CDC42; CTSH; SIDT2; CTTN; IGSF8; MAP2K5; MC1R; HMGB3; HMGB1; CPEB1; TMIE; CTNNB1; TEAD3; BAD; HAND2; SCAP; MITF; ERH; BIN3; CAD; PGF; RGS20; WDR1; SLC37A4; PFN1; MAD1L1; BIN1; NUDC; DCLRE1C; RAI1; NEO1; SLIT3; ETNK2; HOMER1; CNOT2; SIPA1L3; ANKRD11; PSMC4; PSMC5; TTLL5; CNTF; ITGB5; ARIH2; E4F1; ABR; FXN; ATN1; FEZ1; SUFU; TTBK2; MMP24; PRMT5; NRP2; RAB21; E2F4; ARSA; E2F1; BNC2; IFT172; CALR; TOB2; IER2; RND2; ANK3; MBD1; MBD3; CTNNBIP1; ARHGEF18; STK4; PPP1R13L; CMTM7; EEF2; TRIM11; FRYL; TUSC2; PDGFRB; SHARPIN; PPP2R3A; SF1; IKBKB; SFMBT1; PTK2B; HPS6; SCRT1; FBLN1; CLSTN1; USP21; EHD2; LSR; RARG; ADAMTS2; TSSK2; ATP6AP2; NFE2L1; CLOCK; ARHGEF2; JAM3; ADRA2C; CDC42EP2; RELA; SPIN1; HMG20B; CDKN2C; CDKN2A; NCKIPSD; HPCAL4; THOC6; STRA6; ACIN1; RAMP1; INPP5B; RAMP2; LDB1; PALM; SSH3; NMT1; ATP6V1B1; FBLIM1; THOC2; ZFYVE19; SNAI1; PAFAH1B1; TUFT1; THOC5; KIAA1161; RAB27A; SETD2; FN1; POMT2; PARK2; ARID1A; RFX2; RFX3; DDX41; CDON; DIXDC1; WFS1; PAK1; GON4L; RECQL4; SNX1; FOXP1; NSDHL; CRELD1; EVI5; TMEM107; SLC29A1; MYCBPAP; SMARCB1; EXOSC6; BANP; NEK6; ADORA2A; NEK3; YPEL3; TULP3; CCNE1; TIMELESS; NEK8; EHMT1; SUV39H1; STK25; PRKG1; ANAPC2; BMPR2; AKR1C3; CSK; ENG; EBP; MRAP; PPHLN1; ZNF24; SHB; IGFBP4; SCMH1; PHLDB1; SARM1; TUBB2B; IRF1; PRRX2; IRF7; IRF6; MEF2C; MAOB; DTYMK; PCDH17; ZNF335; FPGS; TRIM3; ALKBH5; ISLR2; GAS6; EFNB2; EFNB3; HDAC3; EFNB1; HDAC5; PSMB10; HDAC9; NODAL; EIF2B4; ATM; AHI1; IL27RA; STXBP1; PHB; SPRY1; SMPD1; PRPF40A; AKR1C1; MMP11; MLF1; USF2; IFT140; PLEKHO1; ZBTB17; RAB18; MYH9; VKORC1; TRADD; FANCG; CDKN1C; CYB5D2; AAMP; FANCA; RAPGEFL1; RAB13; FBXW4; TNFRSF6B; ZNF430; PHACTR4; FGD1; CLIC1; UGDH; NAGLU; ASCL1; CELSR3; COL5A2; ENO1; NPY2R; ENO3; SND1; BBX; CLU; TRIM62; PDCD4; TDRKH; RAB26; LLGL1; DLC1; TFIP11; PARD3; MFN2; BASP1; AATF; CDK5; PLXNB1; CDK9; NPFF; SH3PXD2A; SDCBP2; NEU1; MAD2L2; TGFB1; GTF3C5; PHB2; ERCC1; ERCC2; PHF19; AGRN; MEGF8; NDRG1; NLGN2; SEMA6D; PRKX; SMARCA4; SEMA6C; POMZP3; IMPACT; CEBPB; CEBPA; LOXL3; PYGO2; INPPL1; ALX3; TNFRSF12A; BID; KLHL17; NLGN1; KIF13B; DVL3; ARNT; NARFL; ABCB6; AURKB; ARHGDIA; UCN; PAF1; ASF1B; VASN; HEG1; SOX7; DAB2IP; GNB1; NBR1; ENDOG; RDH10; CHRM1; CDC42EP4; CPLX2; MGAT1; MPST; ENAH; AP2A2; KRIT1; LMNA; CTNS; CTNNA2; ROBO3; NOTCH4; ABI1; NRIP1; TLX2; NOTCH3; SCRIB; GSTP1; CUL4B; SLC1A2; NCDN; PTCH2; UNC13B; TGIF2; ACADVL; ADD2; UNC13A; AGER; MYL6; TSNAXIP1; TPI1; FURIN; CALM3; RDH13; MAP3K3; FMNL1; ATP6AP1; MPP5; MAP3K5; DCX; WDR62; ZIC2; ATP6V0D1; CUL7; CLN8; BRCA1; FLCN; TRIM28; HOXD9; VRK3; SHC3; PRMT2; CLCN2; LRRC6; PRMT7; CCDC78; KCNAB2; GLRX2; HMGCR; RFNG; DLL3; NQO1; FGFR2; ROR2; SETD6; IQCG; DNAJA3; ADCY6; PCYT1B; INPP5E; INPP5F; CNN2; KIF5A; KIF5C; LYL1; PQBP1; KIT; FREM1; UTRN; BST1; EPC1; CHRFAM7A; HDAC10; CHAC1; TACC2; ZBTB24; EMD; AES; GBA; HRAS; HSPG2; CARM1; KIF26B; SEPT4; RICTOR; TIMM8A; ETS1; ZSCAN2; DAXX; AP2S1; KEAP1; USP22; MKS1; TMEM79; JUP; XPA; TROVE2; CDK5R2; BSCL2; MAFG; CHEK2; POLL; EPHB2; PLCB1; MAP1B; UNC5A; ITGB1BP2; STK40; UNC5B; VGF; WWOX; EPN1; TUBB3; MAN2A1; CCM2; HERC1; TAGLN3; ASB1; DTNBP1; DNER; ABCA12; SCUBE1; TP53; LGMN; CHST8; DDR2; DDR1; XAB2; NUP62; EIF4EBP1; STK36; HIF1AN; PPP2R5D; SIX6; PPP2R5B; DGCR2; RASIP1; MKNK2; CRYGS; H2AFY2; CTDP1; TBX2; OLFM2; PLCE1; SLC39A1; ARHGEF1; DGCR6; POFUT2; CD9; POFUT1; SNX17; H2AFX; APH1A; EHMT2; STAT3; ARHGAP22; ZHX3; CD83; RPS14; CSNK1D; FHL2; FHL3; DHTKD1; FOSL2; FLII; USP2; IFT20; FBXO45; SRC; DHFR; CD63; RPS6KA1; EFNA4; ATRN; SRR; SMTN; FLT3LG; VPS4A; LGALS3; QARS; PIR; GPR68; RPS6KA5; MAEA; SLC27A4; TOLLIP; LRP4; GALNT11; PLXNA3; PLXNA2; PLXNA1; GSTM3; CHURC1; PPP1R15A; CHRD; COL1A2; WDR18; PDE4D; PPIB; GPSM1; PPP2R1A; ANKH; CD276; IL1RAPL1; PSMD11; SPATA20; CORO1B; MAP2K2; BMPR1B; CORO1A; AEBP1; MAP4; GPD2; AACS; BCL9L; SPTBN2; ALDH1A2; ALDH1A3; RAP1GAP; WASF2; CBR1; NPAS2; MLLT3; MAPKAP1; VPS33A; GAK; RGS2; ATG5; NRG1; SERPINI1; TERT; RGS9; GAA; FLOT1; NPHP4; ESRRA; NRGN; PKN1; MSI1; PIGT; FLNA; ALDOA; ALDOC; TAGLN; CASP6; SQSTM1; FMN1; PACRG; AXIN1; PPP1CA; PRDM8; MPPED2; DRD2; CASP9; RAD54L; BAZ1B; BCL9; SECISBP2; MAPT; SLC9A3R2; COL6A1; UFD1L; BCL2L1; GSS; TNFRSF25; TIMP1; PHGDH; ISL2; QDPR; DHCR7; AKT1; LRFN1; BOK; SPG7; SCO2; CREBL2; DISC1; S100A13; BOC; OGG1; GSN; SMAP1; SDCCAG8; TTC7A; ZNF148; GRK5; CIAPIN1; NTRK1; NTRK2; NTRK3; NAV1; SPHK2; CAPN5; CSNK1E; GOLGA4; LIG1; CAPN1; LIG3; PTBP1; SOD2; SUZ12; SYNCRIP; BAMBI; MYLIP; VAPA; CACNA2D2; PMS2; NXN; BMP2; NPHP3; PCGF2; KCTD15; BMP1; CSPG5; PPP2CA; HYAL2; EIF4G1; ROBO2; GSK3A; HYAL3; CCNF; GRB2; MAGED1; ELP3; HYAL1; MOSPD3; VPS72; RYBP; GPR161; ULK3; HLA-B; NFKBIA; HLA-G; HOXC6; TMEM119; ULK1; RNH1; FBXO9; CCHCR1; LUC7L; MAPKAPK2; PLXNB2; PLXNB3; CHD2; CLASP1; CHD7; PREX1; ASXL1; THRA; UBE3A; MNT; THBS3; TFE3; NR4A3; PCOLCE; TGFB1I1; IFT122; LZTS2; LZTS1; DCHS1; SIRT2; NR4A1; GNPNAT1; TRO; RAB3GAP1; CDC25B; CBX2; RNF41; GYS1; HEXIM1; DAG1; POLR2C; GAS2L1; NELL1; SART1; NAPA; KIF3B; KIF3A; FAM57B; NLE1; LRIG2; PEX7; USP13; SDC1; VAV3; RBM4; NCL; ITM2B; PRKCZ; SHC4; SNCA; POMT1; EGFL8; RTF1; MCPH1; GAMT; RAD23B; FJX1; ACD; MAST2; EPHA2; CPNE1; VAX2; BCL7B; MAPK12; ABL1; PARVB; UCP2; FZD2; RAB11FIP4; FZD4; BTG1; POLDIP2; TEC; BCL10; AP2M1; MAPK1; FASN; MAPK7; PML; DUSP4; CITED1; FGF17; APAF1; DUSP2; PAQR8; SLC7A5; LRRC8A; ELL; NDRG4; HOXC5; STOX2; HPS4; RCC2; HMGA1; FEV; SEZ6; AMFR; CHRNA7; SERPINF1; PLCD3; PLCD1; FADS1; RBM19; MAPK8IP2; RGS14; FHOD3; ITFG2; TACC1; PKD1; TACC3; ID3; RGS19; STAT5B; SREBF1; PRKACA; DGUOK; ZNF74; CEP152; FBL; KATNB1; NUMA1; KALRN; TRIB3; BCL11A; RIPK1; DUSP22; NNAT; SRF; EIF2S1; P2RX5; ELF4; P2RX7; CRABP2; PRPS1; PLOD3; SLC9A3R1; KCNK2; KCNK3; DOK4; MSX1; VPS13A; DGKD; SRA1; LRP10; LRP5L; DNMT3A; TMEM106B; CLASP2; CNP; PIM1; PRPS1L1; SDHA; GPC3; RXRB; CFL1; NDEL1; ARMC6; SSBP3; USP19; PTPRM; NFIB; ACTN1; SLC40A1; TRIM16; SLC39A3; AARS; UNC45A; SYNGR3; PDE2A; PTPRF; GNA12; DGCR14; GNA11; PTPRO; SERPINH1; ATF3; GSTK1 |
| GO:0048856 | 2 | anatomical structure development | 6,54E-07 | 3,67E-06 | SMARCC2; PGAP1; RNF10; TUBD1; FKBP4; CD8A; ARFRP1; ANP32B; MFAP2; L1CAM; DBN1; SEMA4B; LEMD2; SEMA4F; SEMA4G; DLG3; DLG2; PALMD; CAMK1; CDC42SE2; APBB1; TAZ; DMPK; RNF111; THY1; PPHLN1; ZNF689; TCOF1; DIAPH2; CAMK2D; ARHGEF19; MUC1; CAMK2G; MTCH1; SLC45A3; CRB2; APRT; SMARCD3; ATF5; SMARCD1; OPA1; GATA2; GBA2; AKAP6; PPP1R9B; DBNL; TRIOBP; WDTC1; CCND1; MAX; RAB11FIP4; ARHGAP12; LRP8; TNFSF13; CAPZB; MMP16; EVL; ELAVL1; ACVR1B; ALDH3A2; NOVA1; ITGA3; POLR1B; RAD1; SFXN1; UROD; DUSP22; PHLDA3; TCF3; GAP43; SOX12; DLD; GLG1; NF2; ZNF513; WDR74; PDLIM7; DNASE1L2; KCNQ2; MRAS; CDK5RAP2; NFASC; SDF4; COL4A2; VEGFB; GRN; WDR77; CHST3; GDI1; PRKD2; ARNT2; PLXNC1; RPS6KA1; RPS6KA2; CSNK1A1; VASH1; TNFSF12; MTHFD1L; PSAP; PSMD13; CHD8; PDE6B; FRAT2; GPX4; FLOT2; FMNL3; IL11RA; HES1; FLNB; EIF4A1; CRIP2; ACP6; DNMT1; ACP2; STIM1; TLE3; DEAF1; SMAD6; TK1; SMAD1; RET; SRGAP2; SMO; TNFRSF10B; POLE; PRKCSH; AKAP13; SLC4A2; POLM; DCTN1; TSC2; EFHC1; HTRA2; TSC1; HMBS; MDK; SEZ6L2; NR2F6; PCDHB14; ARF6; ELAVL3; DIP2A; IGF2R; BRWD3; AP3D1; BSG; ATOH8; PTPRU; FBXO31; LAMA4; LAMA5; CTF1; MECP2; NASP; MCAM; EIF4ENIF1; OPRM1; SUPT6H; FAIM2; MARCH5; F3; ZFYVE27; APBA3; APBA2; RIC8A; AK2; FAM20C; TBCC; BPNT1; ABT1; CXXC4; MME; CDK5RAP3; CDK5RAP1; SLC38A3; NBEAL2; CDC42; CTSH; CTTN; IGSF8; MAP2K5; MC1R; HMGB3; HMGB1; CPEB1; TMIE; CTNNB1; TEAD3; BAD; HAND2; MITF; BIN1; BIN3; CAD; PGF; RGS20; WDR1; SLC37A4; PFN1; MAD1L1; NUDC; DCLRE1C; RAI1; NEO1; SLIT3; ETNK2; HOMER1; CNOT2; SIPA1L3; ANKRD11; PSMC4; PSMC5; TTLL5; CNTF; ITGB5; ARIH2; E4F1; ABR; FXN; ATN1; FEZ1; SUFU; TTBK2; MMP24; PRMT5; NRP2; RAB21; E2F4; ARSA; E2F1; BNC2; IFT172; CALR; TOB2; IER2; RND2; ANK3; MBD1; MBD3; CTNNBIP1; ARHGEF18; STK4; PPP1R13L; CMTM7; EEF2; TRIM11; FRYL; TUSC2; EFNB3; SHARPIN; PPP2R3A; SF1; IKBKB; SFMBT1; PTK2B; ZNF335; SCRT1; FBLN1; CLSTN1; USP21; EHD2; LSR; RARG; ADAMTS2; TSSK2; ATP6AP2; NFE2L1; CLOCK; ARHGEF2; JAM3; ADRA2C; CDC42EP2; RELA; SPIN1; HMG20B; CDKN2C; CDKN2A; NCKIPSD; HPCAL4; THOC6; STRA6; ACIN1; RAMP1; INPP5B; RAMP2; LDB1; PALM; SSH3; NMT1; ATP6V1B1; FBLIM1; THOC2; ZFYVE19; SNAI1; PAFAH1B1; TUFT1; THOC5; KIAA1161; VAT1; FN1; PARK2; ARID1A; RFX2; RFX3; DDX41; CDON; DIXDC1; WFS1; PAK1; RECQL4; SNX1; FOXP1; NSDHL; CRELD1; EVI5; TMEM107; SLC29A1; MYCBPAP; PINK1; SMARCB1; EXOSC6; BANP; ADORA2A; NEK3; TULP3; CCNE1; TIMELESS; NEK8; EHMT1; COL18A1; STK25; PRKG1; ANAPC2; BMPR2; AKR1C3; CSK; ENG; EBP; ZNF24; SHB; IGFBP4; SCMH1; PHLDB1; SARM1; TUBB2B; IRF1; PRRX2; IRF7; IRF6; MEF2C; MAOB; DTYMK; PCDH17; FPGS; TRIM3; ISLR2; GAS6; EFNB2; PDGFRB; HDAC3; EFNB1; HDAC5; PSMB10; HDAC9; NODAL; EIF2B4; ATM; AHI1; IL27RA; STXBP1; SPRY1; SMPD1; PRPF40A; AKR1C1; MMP11; MLF1; USF2; IFT140; PLEKHO1; ZBTB17; RAB18; MYH9; VKORC1; TRADD; FANCG; CDKN1C; CYB5D2; AAMP; FANCA; RAPGEFL1; RAB13; FBXW4; TNFRSF6B; ZNF430; PHACTR4; FGD1; KLHL17; UGDH; NAGLU; ASCL1; CELSR3; COL5A2; ENO1; NPY2R; ENO3; BBX; CLU; TRIM62; RAB26; LLGL1; DLC1; TFIP11; PARD3; MFN2; BASP1; AATF; CDK5; PLXNB1; CDK9; NPFF; SH3PXD2A; SDCBP2; NEU1; MAD2L2; TGFB1; GTF3C5; PHB2; ERCC1; ERCC2; AGRN; MEGF8; NDRG1; NLGN2; SEMA6D; PRKX; SMARCA4; SEMA6C; POMZP3; IMPACT; CEBPB; CEBPA; LOXL3; PYGO2; INPPL1; ALX3; TNFRSF12A; BID; NLGN1; KIF13B; DVL3; ARNT; NARFL; ABCB6; AURKB; ARHGDIA; UCN; PAF1; ASF1B; VASN; HEG1; SOX7; DAB2IP; GNB1; NBR1; ENDOG; RDH10; CHRM1; CDC42EP4; CPLX2; MGAT1; MPST; ENAH; AP2A2; KRIT1; LMNA; CTNS; CTNNA2; ROBO3; NOTCH4; ABI1; NRIP1; TLX2; NOTCH3; SCRIB; GSTP1; CUL4B; SLC1A2; NCDN; PTCH2; UNC13B; TGIF2; ACADVL; ADD2; UNC13A; AGER; MYL6; TPI1; CALM3; RDH13; MAP3K3; FMNL1; ATP6AP1; MPP5; MAP3K5; DCX; WDR62; ZIC2; ATP6V0D1; CUL7; CLN8; BRCA1; FLCN; TRIM28; HOXD9; VRK3; SHC3; PRMT2; CLCN2; LRRC6; CEP152; CCDC78; KCNAB2; HMGCR; RFNG; DLL3; SIDT2; FGFR2; ROR2; PARVB; IQCG; DNAJA3; ADCY6; PCYT1B; INPP5E; INPP5F; CNN2; KIF5A; KIF5C; LYL1; PQBP1; KIT; FREM1; UTRN; BST1; EPC1; CHRFAM7A; HDAC10; CHAC1; LZTR1; TACC2; ZBTB24; EMD; AES; GBA; HRAS; HSPG2; CARM1; KIF26B; MESDC2; SEPT4; RICTOR; TIMM8A; ETS1; ZSCAN2; DAXX; AP2S1; KEAP1; USP22; MKS1; TMEM79; JUP; ZNF74; CDK5R2; MAFG; POLL; EPHB2; PLCB1; MAP1B; UNC5A; ITGB1BP2; STK40; SMAP1; UNC5B; VGF; WWOX; EPN1; TUBB3; MAN2A1; CCM2; HERC1; TAGLN3; ASB1; DTNBP1; DNER; ABCA12; SCUBE1; TP53; TOLLIP; CHST8; DDR2; DDR1; XAB2; EIF4EBP1; STK36; HIF1AN; PPP2R5D; SIX6; PPP2R5B; RASIP1; MKNK2; CRYGS; H2AFY2; CTDP1; TBX2; OLFM2; PLCE1; SLC39A1; ARHGEF1; DGCR6; POFUT2; CD9; POFUT1; SNX17; H2AFX; APH1A; EHMT2; STAT3; ARHGAP22; CD83; RPS14; CSNK1D; FHL2; FHL3; DHTKD1; FOSL2; FLII; USP2; IFT20; FBXO45; SRC; DHFR; CD63; EFNA4; ATRN; SRR; SMTN; FLT3LG; VPS4A; LGALS3; QARS; PIR; GPR68; RPS6KA5; MAEA; SLC27A4; LRP4; GALNT11; PLXNA3; PLXNA2; PLXNA1; GSTM3; CHURC1; PPP1R15A; CHRD; COL1A2; AGPAT2; WDR18; PDE4D; PPIB; GPSM1; PPP2R1A; ANKH; CD276; IL1RAPL1; PSMD11; SPATA20; CORO1B; MAP2K2; BMPR1B; CORO1A; AEBP1; MAP4; GPD2; AACS; BCL9L; SPTBN2; ALDH1A2; ALDH1A3; RAP1GAP; WASF2; CBR1; NPAS2; MLLT3; MAPKAP1; VPS33A; GAK; ATG5; NRG1; SERPINI1; SEMA6A; RGS9; GAA; FLOT1; NPHP4; ESRRA; NRGN; PKN1; MSI1; PIGT; FLNA; ALDOA; ALDOC; TAGLN; CASP6; FMN1; PACRG; AXIN1; PPP1CA; PRDM8; MPPED2; DRD2; CASP9; RAD54L; BAZ1B; BCL9; SECISBP2; MAPT; SLC9A3R2; COL6A1; UFD1L; BCL2L1; GSS; TNFRSF25; TIMP1; PHGDH; ISL2; QDPR; DHCR7; AKT1; LRFN1; BOK; SPG7; SCO2; DISC1; S100A13; BOC; TROVE2; GSN; SLC39A13; SDCCAG8; TTC7A; ZNF148; DGCR2; CIAPIN1; NTRK1; NTRK2; NTRK3; NAV1; SPHK2; CAPN5; CSNK1E; GOLGA4; LIG1; CAPN1; LIG3; PTBP1; SETD2; SOD2; BAMBI; MYLIP; VAPA; CACNA2D2; PMS2; NXN; BMP2; NPHP3; PCGF2; KCTD15; BMP1; CSPG5; PPP2CA; HYAL2; EIF4G1; ROBO2; GSK3A; HYAL3; CCNF; GRB2; MAGED1; ELP3; HYAL1; MOSPD3; EGFL8; RYBP; GPR161; HLA-B; NFKBIA; HLA-G; HOXC6; TMEM119; ULK1; RNH1; CCHCR1; LUC7L; MAPKAPK2; PLXNB2; PLXNB3; CHD2; CLASP1; CHD7; PREX1; ASXL1; THRA; UBE3A; MNT; THBS3; TFE3; NR4A3; PCOLCE; TGFB1I1; IFT122; LZTS2; LZTS1; DCHS1; SIRT2; NR4A1; GNPNAT1; TRO; RAB3GAP1; CDC25B; CBX2; RNF41; GYS1; HEXIM1; DAG1; SLC26A6; GAS2L1; NELL1; SART1; NAPA; KIF3B; KIF3A; NLE1; LRIG2; PEX7; SDC1; VAV3; RBM4; NCL; ITM2B; PRKCZ; POMT1; POMT2; RTF1; MCPH1; GAMT; RAD23B; FJX1; ACD; EPHA2; CPNE1; VAX2; GON4L; MAPK12; ABL1; UCP2; FZD2; FZD4; BTG1; POLDIP2; TEC; BCL10; AP2M1; MAPK1; FASN; MAPK7; PML; DUSP4; CITED1; FGF17; APAF1; DUSP2; PAQR8; SLC7A5; LRRC8A; ELL; NDRG4; HOXC5; STOX2; RCC2; FEV; SEZ6; CHRNA7; SERPINF1; PLCD3; PLCD1; TSNAXIP1; RBM19; MAPK8IP2; RGS14; FHOD3; ITFG2; TACC1; PKD1; TACC3; ID3; RGS19; STAT5B; SREBF1; PRKACA; DGUOK; KATNB1; NUMA1; KALRN; BCL11A; RIPK1; NNAT; SRF; P2RX5; P2RX7; CRABP2; PRPS1; PLOD3; SLC9A3R1; KCNK2; KCNK3; DOK4; MSX1; VPS13A; DGKD; LRP10; LRP5L; DNMT3A; TMEM106B; CLASP2; CNP; PIM1; PRPS1L1; SDHA; GPC3; RXRB; CFL1; NDEL1; ARMC6; CLTCL1; SSBP3; USP19; PTPRM; NFIB; ACTN1; SLC40A1; TRIM16; SLC39A3; AARS; UNC45A; SYNGR3; PDE2A; PTPRF; GNA12; DGCR14; GNA11; PTPRO; SERPINH1; ATF3; GSTK1 |
| GO:0048589 | 2 | developmental growth | 0,001838 | 0,00638 | CCNE1; PPP2R3A; DHCR7; L1CAM; SEMA4B; SEMA4F; GSN; RARG; FGFR2; PARK2; GOLGA4; DCX; TRIM28; PRMT2; STRA6; XPA; STK4; CACNA2D2; PAFAH1B1; TBX2; AKAP6; WDTC1; FN1; GSK3A; BMPR2; FOXP1; CDKN1C; FMN1; CARM1; KIF26B; TNFRSF12A; ARIH2; PLXNB1; GAP43; UBE3A; THBS3; DISC1; CAMK2D; HEG1; SEMA4G; PLCB1; MAP1B; STK40; CCM2; ANAPC2; PLXNC1; LGMN; DDR2; PSAP; MEF2C; FXN; ISLR2; GAMT; SMAD1; CTDP1; SMO; SPRY1; ABL1; NTRK3; HTRA2; EHMT2; STAT3; TEC; SRF; PYGO2; ATRN; ENO3; UNC13A; LRP4; APBA2; PLXNA3; PLXNA2; PLXNA1; BMPR1B; STAT5B; BASP1; CDK5; PDE4D; PPIB; CDC42; TGFB1; CTTN; ERCC1; ERCC2; BCL11A; AGRN; MEGF8; GPD2; SEMA6D; SPTBN2; BIN3; SEMA6A; IMPACT; CRABP2; NDRG4; CPNE1; KCNK2; SEMA6C; RAI1; SLIT3; ETNK2; NRG1; RDH10; ANKRD11; ULK1; CLASP2; PRKCZ; RTF1; NDEL1; NRP2; RAB21; CHD7; BNC2; DRD2; RND2; BCL9; MAPT; ATF5; PPP1R13L; SLC1A2 |
| GO:0008283 | 2 | cell proliferation | 0,002154 | 0,007263 | RNF10; DBN1; TYK2; DLG3; DISC1; BRCA1; IRAK1; LARP1; CAMK2D; SMARCD3; GATA2; CCND1; CCND3; CTBP1; TCF19; TNFSF13; TNFSF12; MMP16; CDKN1C; DUSP22; CSK; NF2; TMEM127; VEGFB; ING4; ING5; ARNT2; RPS6KA1; RPS6KA2; TFAP4; TP53I11; FRAT2; HES1; CRIP2; CAPNS1; SMAD6; SMAD1; SRGAP2; SMO; TNFRSF10B; FOXM1; DCTN2; TSC2; TSC1; RBM10; ATOH8; LAMA5; CTF1; MECP2; NASP; OPRM1; CDCA7L; F3; LMO1; ADARB1; CDK5RAP3; PRKD2; CDC42; SPHK2; IGSF8; WDR6; HMGB1; CPEB1; CTNNB1; BAD; HAND2; BIN1; PGF; TOB2; MAD1L1; CDK10; NUDC; PELO; SLIT3; VWCE; CNTF; MCM10; ARIH2; HGS; E4F1; FXN; FPGS; NRP2; E2F4; E2F1; IFT172; CALR; CTNNBIP1; STK4; WDR77; TUSC2; SF1; IKBKB; PES1; PTK2B; ZNF335; FBLN1; CHRFAM7A; RARG; ARHGEF2; SHMT2; RELA; HDGF; CDKN2C; CDKN2A; CDKN2D; TNFRSF25; PAFAH1B1; DBH; FN1; SCARB1; TRAIP; CDON; GNG2; DIXDC1; PAK1; PDXK; ESRRA; TIMELESS; PKN1; BMPR2; PPP1R9B; TCF3; ENG; IGFBP4; TK1; BCAR1; EGLN3; IRF1; IRF6; MEF2C; GAS8; DTYMK; ALKBH3; GAS6; EFNB2; PDGFRB; EFNB1; PSMB10; NODAL; ATM; SPRY1; MXD4; AKR1C3; NUP62; CCPG1; FANCA; SIPA1; FBXW4; TNFRSF6B; ASCL1; GNB1; CLU; ARNT; DLC1; MFN2; CDK3; CDK5; CDK9; NEU1; TGFB1; PHB2; ERCC1; ERCC2; PRKX; SOX7; SERTAD1; CEBPA; INPPL1; BID; ETS1; AURKB; XRCC6; DAB2IP; NR4A1; CHRM1; PRKCZ; KRIT1; DPH1; SHC4; ABI1; NOTCH3; SCRIB; GSTP1; EEF2; AGER; KIF2C; FURIN; DNMT1; DEAF1; CUL5; CTSH; FLCN; WNK2; CREB3; PRMT5; HMGCR; SIDT2; FGFR2; ROR2; DNAJA3; RBBP9; CNN2; PHB; RNF187; KIT; BST1; EMD; HRAS; MED25; CARM1; NDRG1; RICTOR; NDRG4; EIF5A; JUP; B4GALT7; ZNF580; TP53; DDR2; DDR1; RHBDF1; TBX2; GRN; PLCE1; ARHGEF1; CD9; RPS9; STAT3; CD81; FOSL2; IFT20; MARCKSL1; SRC; SRF; PDAP1; COMT; TPD52L2; LGALS3; VASH1; PPP1R15A; CHRD; NMB; CD276; BIRC5; MAP2K2; BMPR1B; CORO1A; MAP2K5; TEAD3; ALDH1A2; RAP1GAP; NRG1; TERT; SMARCB1; CEBPB; COL18A1; CLEC11A; MCM7; DRD2; BCL2L1; MTBP; TIMP1; PLK1; DHCR7; AKT1; BOK; S100A13; LDOC1; MAFG; DGCR8; GRK5; NTRK1; NTRK2; NTRK3; CGREF1; CAPN1; EVI5; SOD2; SUZ12; FNTB; IFI30; BAMBI; BMP2; MAGED1; HYAL1; TBRG1; HLA-A; NFKBIA; HLA-G; TMEM119; TMEM115; PLXNB2; PLXNB3; PLXNB1; MNT; NR4A3; TGFB1I1; IFT122; LZTS2; SIRT2; CDC25B; CDC25A; RNF41; TXLNA; NELL1; RHOG; KIF3A; TAF6; VAV3; ASCC3; RNF126; EPHA2; FOXP1; ABL1; GNAI2; TBC1D8; TICAM1; OGFOD1; BTG1; TEC; MAPK1; PML; CITED1; FGF17; PYGO2; TBRG4; HMGA1; CHRNA7; SERPINF1; PLCD3; PLCD1; TACC1; TACC2; TACC3; STAT5B; PRKACA; CSE1L; CD59; ELF4; P2RX7; SLC9A3R1; KCNK2; MSX1; PIM1; USP13; GPC3; NDEL1; SSBP3; PTPRM; NFIB; PTPRU; PEMT; TINF2; MXI1; SRA1; ATF5; FLT3LG; BAP1; ATF3 |
| GO:0048731 | 3 | system development | 6,53E-08 | 1,11E-06 | SMARCC2; PGAP1; RNF10; CHD8; FKBP4; CD8A; ANP32B; MFAP2; L1CAM; DBN1; SEMA4B; LEMD2; SEMA4F; SEMA4G; DLG3; DLG2; DISC1; CAMK1; APBB1; TAZ; NAPA; THY1; PPHLN1; ZNF689; TCOF1; WDR1; CAMK2D; ARHGEF19; CAMK2G; MTCH1; SLC45A3; CRB2; APRT; SMARCD3; ATF5; SMARCD1; OPA1; GATA2; GBA2; AKAP6; GPR161; DBNL; CCND1; MAX; RAB11FIP4; LRP8; TNFSF13; CAPZB; MMP16; EVL; ACVR1B; ALDH3A2; NOVA1; ITGA3; RAD1; SFXN1; UROD; TCF3; GAP43; SOX12; JAM3; GLG1; NF2; ZNF513; DNASE1L2; KCNQ2; MRAS; CDK5RAP2; NFASC; SDF4; COL4A2; VEGFB; GRN; WDR77; CHST3; GDI1; PRKD2; ARNT2; PLXNC1; RPS6KA1; RPS6KA2; RPS6KA5; VASH1; TNFSF12; MTHFD1L; PSMD11; PSMD13; PDE6B; FLOT1; FMNL3; HES1; FLNB; EIF4A1; CRIP2; ACP6; ACP2; SPHK2; TLE3; DEAF1; SMAD6; SMAD1; RET; SRGAP2; SMO; POLE; PRKCSH; AKAP13; SLC4A2; POLM; DCTN1; TSC2; EFHC1; HTRA2; TSC1; HMBS; MDK; NR2F6; ARF6; ELAVL3; PCDHB14; IGF2R; AP3D1; BSG; ATOH8; LAMA4; LAMA5; CTF1; MECP2; NASP; MCAM; EIF4ENIF1; OPRM1; SUPT6H; FAIM2; F3; ZFYVE27; APBA2; RIC8A; AK2; FAM20C; ABT1; MME; CDK5RAP3; CDK5RAP1; NBEAL2; CDC42; CTSH; CTTN; IGSF8; MAP4; DHFR; HMGB1; CPEB1; TMIE; CTNNB1; TEAD3; BAD; HAND2; MITF; BIN1; BIN3; CAD; PGF; SLC38A3; SLC37A4; PFN1; MAD1L1; DCLRE1C; RAI1; NEO1; SLIT3; ETNK2; HOMER1; ATP6V1B1; SIPA1L3; ANKRD11; PSMC4; PSMC5; CNTF; ARIH2; ABR; FPGS; FEZ1; SUFU; MMP24; PRMT5; NRP2; RAB21; E2F4; ARSA; E2F1; BNC2; IFT172; CALR; TOB2; IER2; RND2; ANK3; MBD1; MBD3; CTNNBIP1; STK4; PPP1R13L; CMTM7; EEF2; TRIM11; FRYL; TUSC2; EFNB3; SHARPIN; PPP2R3A; SF1; IKBKB; SFMBT1; PTK2B; ZNF335; SCRT1; CLSTN1; USP21; CHRFAM7A; LSR; RARG; ADAMTS2; ATP6AP2; NFE2L1; CLOCK; ARHGEF2; ADRA2C; RELA; HMG20B; CDKN2C; CDKN2A; NCKIPSD; HPCAL4; THOC6; STRA6; ACIN1; RAMP1; RAMP2; LDB1; PALM; SSH3; PHGDH; THOC2; SNAI1; PAFAH1B1; TUFT1; THOC5; KIAA1161; SETD2; FN1; ARID1A; RFX3; CDON; DIXDC1; WFS1; PAK1; FOXP1; NSDHL; CRELD1; TMEM107; SLC29A1; SMARCB1; EXOSC6; ADORA2A; NEK3; TULP3; CCNE1; TIMELESS; NEK8; SLC9A3R1; COL18A1; STK25; PRKG1; PPP1R9B; CSK; ENG; EBP; ZNF24; SHB; IGFBP4; TK1; PHLDB1; SARM1; TUBB2B; IRF1; IRF7; IRF6; MEF2C; MAOB; PCDH17; ATN1; TRIM3; ISLR2; GAS6; EFNB2; PDGFRB; EFNB1; HDAC5; PSMB10; HDAC9; NODAL; EIF2B4; ATM; AHI1; IL27RA; STXBP1; SPRY1; SMPD1; AKR1C3; MLF1; USF2; IFT140; GAS2L1; RAB18; MYH9; VKORC1; TRADD; FANCG; CDKN1C; CYB5D2; AAMP; FANCA; RAPGEFL1; RAB13; FBXW4; ENAH; ZNF430; PHACTR4; FGD1; KLHL17; NAGLU; ASCL1; CELSR3; COL5A2; NPY2R; ENO3; BBX; CLU; TRIM62; RAB26; LLGL1; DLC1; TFIP11; PARD3; MFN2; BASP1; CDK5; NPFF; SH3PXD2A; SDCBP2; NEU1; MAD2L2; TGFB1; GTF3C5; PHB2; ERCC1; ERCC2; AGRN; MEGF8; NDRG1; NLGN2; SEMA6D; PRKX; SMARCA4; SEMA6C; SEMA6A; IMPACT; CEBPB; CEBPA; LOXL3; INPPL1; ALX3; TNFRSF12A; BID; NLGN1; KIF13B; DVL3; ARNT; NARFL; ABCB6; ARHGDIA; UCN; PAF1; VASN; HEG1; DAB2IP; GNB1; NBR1; RDH10; CHRM1; CPLX2; PRKCZ; AP2A2; KRIT1; LMNA; CTNS; CTNNA2; ROBO3; NOTCH4; ABI1; NRIP1; TLX2; NOTCH3; SCRIB; GSTP1; CUL4B; SLC1A2; NCDN; PTCH2; UNC13B; TGIF2; ADD2; UNC13A; AGER; MYL6; CALM3; RDH13; MAP3K3; DNMT1; ATP6AP1; MPP5; PARK2; DCX; WDR62; ZIC2; ATP6V0D1; CUL7; CLN8; BRCA1; FLCN; TRIM28; HOXD9; SHC3; CLCN2; LRRC6; KCNAB2; HMGCR; RFNG; DLL3; SIDT2; FGFR2; ROR2; DNAJA3; ADCY6; PCYT1B; INPP5E; INPP5F; CNN2; KIF5A; KIF5C; PQBP1; KIT; FREM1; UTRN; BMPR2; EPC1; LYL1; HDAC10; CHAC1; TACC2; ZBTB24; EMD; AES; GBA; HRAS; HSPG2; CARM1; KIF26B; SEPT4; TIMM8A; ETS1; AP2S1; MKS1; TMEM79; JUP; CDK5R2; POLL; EPHB2; PLCB1; MAP1B; UNC5A; STK40; UNC5B; VGF; WWOX; EPN1; TUBB3; MAN2A1; CCM2; HERC1; TAGLN3; ASB1; DTNBP1; DNER; ABCA12; SCUBE1; CHST8; DDR2; DDR1; XAB2; EIF4EBP1; STK36; HIF1AN; PPP2R5D; SIX6; PPP2R5B; RASIP1; MKNK2; CRYGS; H2AFY2; CTDP1; TBX2; PLCE1; SLC39A1; ARHGEF1; DGCR6; POFUT2; CD9; POFUT1; SNX17; H2AFX; APH1A; ANAPC2; STAT3; CD83; NUMA1; MPST; FHL2; FHL3; DHTKD1; FOSL2; USP2; IFT20; FBXO45; SRC; SRF; EFNA4; ATRN; SRR; SMTN; FLT3LG; LGALS3; QARS; GPR68; MAEA; SLC27A4; LRP4; GALNT11; PLXNA3; PLXNA2; PLXNA1; GSTM3; PPP1R15A; CHRD; COL1A2; PPIB; GPSM1; STIM1; ANKH; CD276; IL1RAPL1; PSAP; MOSPD3; MAP2K2; BMPR1B; AEBP1; MAP2K5; GPD2; AACS; BCL9L; SPTBN2; ALDH1A2; ALDH1A3; RAP1GAP; WASF2; NPAS2; MLLT3; MAPKAP1; VPS33A; GAK; ATG5; NRG1; SERPINI1; ITGB1BP2; RGS9; GAA; FBXO31; NPHP4; ESRRA; NRGN; PKN1; MSI1; PIGT; TAGLN; FMN1; BPNT1; AXIN1; PPP1CA; PRDM8; MPPED2; DRD2; CASP9; BAZ1B; BCL9; SECISBP2; MAPT; SLC9A3R2; UFD1L; BCL2L1; GSS; TIMP1; ISL2; DHCR7; AKT1; LRFN1; BOK; SPG7; SCO2; BOC; TROVE2; GSN; SMAP1; SDCCAG8; TTC7A; ZNF148; DGCR2; CIAPIN1; NTRK1; NTRK2; NTRK3; NAV1; ARHGAP22; CAPN5; CSNK1E; GOLGA4; LIG1; CAPN1; LIG3; PIR; SOD2; BAMBI; MYLIP; VAPA; PMS2; NXN; NPHP3; PCGF2; BMP2; BMP1; CSPG5; PPP2CA; HYAL2; EIF4G1; ROBO2; GSK3A; HYAL3; CCNF; GRB2; MAGED1; ELP3; HYAL1; HLA-B; NFKBIA; HLA-G; HOXC6; TMEM119; ULK1; RNH1; LUC7L; MAPKAPK2; PLXNB2; PLXNB3; CHD2; CLASP1; CHD7; PREX1; ASXL1; THRA; UBE3A; THBS3; TFE3; NR4A3; TGFB1I1; IFT122; LZTS2; LZTS1; DCHS1; SIRT2; NR4A1; GNPNAT1; RAB3GAP1; RNF41; GYS1; HEXIM1; DAG1; NELL1; SART1; SEZ6L2; KIF3A; NLE1; LRIG2; PEX7; SDC1; VAV3; NCL; ITM2B; GPC3; MCPH1; GAMT; RAD23B; FJX1; ACD; EPHA2; VAX2; GON4L; MAPK12; ABL1; UCP2; FZD2; FZD4; BTG1; BCL10; AP2M1; MAPK1; FASN; MAPK7; PML; CITED1; FGF17; APAF1; SLC7A5; LRRC8A; PYGO2; NDRG4; QDPR; STOX2; PTPRU; FEV; SEZ6; CHRNA7; SERPINF1; PLCD3; PLCD1; MAPK8IP2; RGS14; FHOD3; ITFG2; TACC1; PKD1; TACC3; ID3; STAT5B; SREBF1; PRKACA; DGUOK; KATNB1; RPS14; KALRN; BCL11A; RIPK1; NNAT; P2RX5; P2RX7; CRABP2; PRPS1; PLOD3; CPNE1; KCNK2; KCNK3; DOK4; MSX1; VPS13A; LRP10; DNMT3A; TMEM106B; CLASP2; CNP; PRPS1L1; SDHA; PLXNB1; RXRB; CFL1; NDEL1; ARMC6; SSBP3; USP19; SERPINH1; NFIB; ACTN1; SLC40A1; TRIM16; SLC39A3; AARS; UNC45A; SYNGR3; PDE2A; PTPRF; DGCR14; GNA11; PTPRO; PTPRM; ATF3 |
| GO:0007275 | 3 | multicellular organism development | 1,50E-07 | 2,37E-06 | SMARCC2; PGAP1; RNF10; TUBD1; FKBP4; CD8A; ARFRP1; ANP32B; MFAP2; L1CAM; DBN1; SEMA4B; LEMD2; SEMA4F; SEMA4G; DLG3; DLG2; DISC1; CAMK1; APBB1; TAZ; NAPA; RNF111; THY1; PPHLN1; ZNF689; TCOF1; DIAPH2; CAMK2D; ARHGEF19; MUC1; CAMK2G; MTCH1; SLC45A3; CRB2; APRT; SMARCD3; ATF5; SMARCD1; OPA1; GATA2; GBA2; AKAP6; PPP1R9B; DBNL; WDTC1; CCND1; MAX; RAB11FIP4; EHMT1; LRP8; TNFSF13; CAPZB; MMP16; EVL; ELAVL1; ACVR1B; ALDH3A2; NOVA1; ITGA3; POLR1B; RAD1; SFXN1; UROD; DUSP22; TCF3; GAP43; SOX12; DLD; GLG1; NF2; ZNF513; WDR74; PDLIM7; DNASE1L2; KCNQ2; MRAS; CDK5RAP2; NFASC; SDF4; COL4A2; VEGFB; GRN; WDR77; CHST3; GDI1; PRKD2; ARNT2; PLXNC1; RPS6KA1; RPS6KA2; RPS6KA5; VASH1; TNFSF12; MTHFD1L; PSAP; KCTD15; PSMD13; CHD8; PDE6B; FRAT2; GPX4; FMNL3; IL11RA; HES1; FLNB; EIF4A1; CRIP2; ACP6; ACP2; SPHK2; TLE3; DEAF1; SMAD6; TK1; SMAD1; RET; SRGAP2; SMO; TNFRSF10B; POLE; PRKCSH; AKAP13; SLC4A2; POLM; DCTN1; TSC2; EFHC1; HTRA2; TSC1; HMBS; MDK; SEZ6L2; NR2F6; ARF6; ELAVL3; DIP2A; IGF2R; AP3D1; BSG; ATOH8; PCDHB14; FBXO31; LAMA4; LAMA5; CTF1; MECP2; NASP; MCAM; EIF4ENIF1; OPRM1; SUPT6H; FAIM2; F3; ZFYVE27; APBA3; APBA2; RIC8A; AK2; FAM20C; PHGDH; ABT1; CXXC4; MME; CDK5RAP3; CDK5RAP1; SLC38A3; NBEAL2; CDC42; CTSH; CTTN; IGSF8; MAP2K5; MC1R; HMGB3; HMGB1; CPEB1; TMIE; CTNNB1; TEAD3; BAD; HAND2; MITF; BIN1; BIN3; CAD; PGF; RGS20; WDR1; SLC37A4; PFN1; MAD1L1; NUDC; DCLRE1C; RAI1; NEO1; SLIT3; ETNK2; HOMER1; CNOT2; SIPA1L3; ANKRD11; PSMC4; PSMC5; CNTF; ITGB5; ARIH2; E4F1; ABR; FXN; FPGS; FEZ1; SUFU; MMP24; PRMT5; NRP2; RAB21; E2F4; ARSA; E2F1; BNC2; IFT172; CALR; TOB2; IER2; RND2; ANK3; MBD1; MBD3; CTNNBIP1; STK4; PPP1R13L; CMTM7; EEF2; TRIM11; FRYL; TUSC2; EFNB3; SHARPIN; PPP2R3A; SF1; IKBKB; SFMBT1; PTK2B; ZNF335; SCRT1; FBLN1; CLSTN1; USP21; CHRFAM7A; LSR; RARG; ADAMTS2; ATP6AP2; NFE2L1; CLOCK; ARHGEF2; JAM3; ADRA2C; RELA; SPIN1; HMG20B; CDKN2C; CDKN2A; NCKIPSD; HPCAL4; THOC6; STRA6; ACIN1; RAMP1; INPP5B; RAMP2; LDB1; PALM; SSH3; NMT1; ATP6V1B1; THOC2; ZFYVE19; SNAI1; PAFAH1B1; TUFT1; THOC5; KIAA1161; SETD2; FN1; ARID1A; RFX3; DDX41; CDON; DIXDC1; WFS1; PAK1; RECQL4; FOXP1; NSDHL; CRELD1; EVI5; TMEM107; SLC29A1; MYCBPAP; SMARCB1; EXOSC6; BANP; ADORA2A; NEK3; TULP3; CCNE1; TIMELESS; NEK8; SLC9A3R1; COL18A1; STK25; PRKG1; BMPR2; AKR1C3; CSK; ENG; EBP; ZNF24; SHB; IGFBP4; SCMH1; PHLDB1; SARM1; TUBB2B; IRF1; PRRX2; IRF7; IRF6; MEF2C; MAOB; PCDH17; ATN1; TRIM3; ISLR2; GAS6; EFNB2; PDGFRB; EFNB1; HDAC5; PSMB10; HDAC9; NODAL; EIF2B4; ATM; AHI1; IL27RA; STXBP1; SPRY1; SMPD1; CBX2; MMP11; MLF1; USF2; IFT140; ZBTB17; RAB18; MYH9; VKORC1; TRADD; FANCG; CDKN1C; CYB5D2; AAMP; FANCA; RAPGEFL1; RAB13; FBXW4; TNFRSF6B; ZNF430; PHACTR4; FGD1; KLHL17; UGDH; NAGLU; ASCL1; CELSR3; COL5A2; ENO1; NPY2R; ENO3; BBX; CLU; TRIM62; RAB26; LLGL1; DLC1; TFIP11; PARD3; MFN2; BASP1; AATF; CDK5; NPFF; SH3PXD2A; SDCBP2; NEU1; MAD2L2; TGFB1; GTF3C5; PHB2; ERCC1; ERCC2; AGRN; MEGF8; NDRG1; NLGN2; SEMA6D; PRKX; SMARCA4; SEMA6C; SEMA6A; IMPACT; CEBPB; CEBPA; LOXL3; PYGO2; INPPL1; ALX3; TNFRSF12A; BID; NLGN1; KIF13B; DVL3; ARNT; NARFL; ABCB6; AURKB; ARHGDIA; UCN; PAF1; ASF1B; VASN; HEG1; SOX7; DAB2IP; GNB1; NBR1; ENDOG; RDH10; CHRM1; CPLX2; MGAT1; PRKCZ; ENAH; AP2A2; KRIT1; LMNA; CTNS; CTNNA2; ROBO3; NOTCH4; ABI1; NRIP1; TLX2; NOTCH3; SCRIB; GSTP1; CUL4B; SLC1A2; NCDN; PTCH2; UNC13B; TGIF2; ADD2; UNC13A; AGER; MYL6; TPI1; CALM3; RDH13; MAP3K3; DNMT1; ATP6AP1; MPP5; PARK2; DCX; WDR62; ZIC2; ATP6V0D1; CUL7; CLN8; BRCA1; FLCN; TRIM28; HOXD9; SHC3; CLCN2; LRRC6; KCNAB2; HMGCR; RFNG; DLL3; SIDT2; FGFR2; ROR2; DNAJA3; ADCY6; PCYT1B; INPP5E; INPP5F; CNN2; KIF5A; KIF5C; PQBP1; KIT; FREM1; UTRN; BST1; EPC1; LYL1; HDAC10; CHAC1; TACC2; ZBTB24; EMD; AES; GBA; HRAS; HSPG2; CARM1; KIF26B; SEPT4; RICTOR; TIMM8A; ETS1; ZSCAN2; AP2S1; KEAP1; USP22; MKS1; TMEM79; JUP; ZNF74; CDK5R2; MAFG; POLL; EPHB2; PLCB1; MAP1B; UNC5A; STK40; UNC5B; VGF; WWOX; EPN1; TUBB3; MAN2A1; CCM2; HERC1; TAGLN3; ASB1; DTNBP1; DNER; ABCA12; SCUBE1; TP53; CHST8; DDR2; DDR1; XAB2; EIF4EBP1; STK36; HIF1AN; PPP2R5D; SIX6; PPP2R5B; RASIP1; MKNK2; CRYGS; H2AFY2; CTDP1; TBX2; PLCE1; SLC39A1; ARHGEF1; DGCR6; POFUT2; CD9; POFUT1; SNX17; H2AFX; APH1A; ANAPC2; STAT3; CD83; RPS14; MPST; FHL2; FHL3; DHTKD1; FOSL2; FLII; USP2; IFT20; FBXO45; SRC; DHFR; SRF; EFNA4; ATRN; SRR; SMTN; FLT3LG; VPS4A; LGALS3; QARS; GPR68; MAEA; SLC27A4; LRP4; GALNT11; PLXNA3; PLXNA2; PLXNA1; GSTM3; CHURC1; PPP1R15A; CHRD; COL1A2; WDR18; PPIB; GPSM1; STIM1; ANKH; CD276; IL1RAPL1; PSMD11; SPATA20; MOSPD3; MAP2K2; BMPR1B; AEBP1; MAP4; GPD2; AACS; BCL9L; SPTBN2; ALDH1A2; ALDH1A3; RAP1GAP; WASF2; NPAS2; MLLT3; MAPKAP1; VPS33A; GAK; ATG5; NRG1; SERPINI1; ITGB1BP2; RGS9; GAA; FLOT1; NPHP4; ESRRA; NRGN; PKN1; MSI1; PIGT; TAGLN; FMN1; BPNT1; AXIN1; PPP1CA; PRDM8; MPPED2; DRD2; CASP9; RAD54L; BAZ1B; BCL9; SECISBP2; MAPT; SLC9A3R2; COL6A1; UFD1L; BCL2L1; GSS; TNFRSF25; TIMP1; ISL2; QDPR; DHCR7; AKT1; LRFN1; BOK; SPG7; SCO2; BOC; TROVE2; GSN; SMAP1; SDCCAG8; TTC7A; ZNF148; DGCR2; CIAPIN1; NTRK1; NTRK2; NTRK3; NAV1; ARHGAP22; CAPN5; CSNK1E; GOLGA4; LIG1; CAPN1; LIG3; PIR; SOD2; BAMBI; MYLIP; VAPA; PMS2; NXN; NPHP3; PCGF2; BMP2; BMP1; CSPG5; PPP2CA; HYAL2; EIF4G1; ROBO2; GSK3A; HYAL3; CCNF; GRB2; MAGED1; ELP3; HYAL1; EGFL8; RYBP; GPR161; HLA-B; NFKBIA; HLA-G; HOXC6; TMEM119; ULK1; RNH1; CCHCR1; LUC7L; MAPKAPK2; PLXNB2; PLXNB3; PLXNB1; CLASP1; CHD7; PREX1; ASXL1; THRA; UBE3A; MNT; THBS3; TFE3; NR4A3; PCOLCE; TGFB1I1; IFT122; LZTS2; LZTS1; DCHS1; SIRT2; NR4A1; GNPNAT1; TRO; RAB3GAP1; RNF41; GYS1; HEXIM1; DAG1; GAS2L1; NELL1; SART1; KIF3B; KIF3A; NLE1; LRIG2; PEX7; SDC1; VAV3; NCL; ITM2B; GPC3; POMT1; POMT2; RTF1; MCPH1; GAMT; RAD23B; FJX1; ACD; EPHA2; VAX2; GON4L; MAPK12; ABL1; UCP2; FZD2; FZD4; BTG1; BCL10; AP2M1; MAPK1; FASN; MAPK7; PML; DUSP4; CITED1; FGF17; APAF1; DUSP2; PAQR8; SLC7A5; LRRC8A; ELL; NDRG4; HOXC5; STOX2; PTPRU; FEV; SEZ6; CHRNA7; SERPINF1; PLCD3; PLCD1; TSNAXIP1; RBM19; MAPK8IP2; RGS14; FHOD3; ITFG2; TACC1; PKD1; TACC3; ID3; RGS19; STAT5B; SREBF1; PRKACA; DGUOK; KATNB1; NUMA1; KALRN; BCL11A; RIPK1; NNAT; TSSK2; P2RX5; P2RX7; CRABP2; PRPS1; PLOD3; CPNE1; KCNK2; KCNK3; DOK4; MSX1; VPS13A; DGKD; LRP10; LRP5L; DNMT3A; TMEM106B; CLASP2; CNP; PIM1; PRPS1L1; SDHA; CHD2; RXRB; CFL1; NDEL1; ARMC6; SSBP3; USP19; PTPRM; NFIB; ACTN1; SLC40A1; TRIM16; SLC39A3; AARS; UNC45A; SYNGR3; PDE2A; PTPRF; GNA12; DGCR14; GNA11; PTPRO; SERPINH1; ATF3 |
| GO:0097190 | 3 | apoptotic signaling pathway | 4,08E-07 | 5,64E-06 | BCL2L1; TM2D1; FBXO18; FHIT; MEIS3; RAD9A; AKT1; BOK; KIAA0141; GSN; HYOU1; ARHGEF2; PARK2; PLA2G6; CUL5; RELA; CTSH; FAIM2; SOD2; CDKN2D; MUC1; CREB3; NRG1; STK4; NMT1; OPA1; SNAI1; PTTG1IP; DBH; PCGF2; P4HB; HYAL2; GSK3A; MAGED1; WFS1; TNFSF12; TRIM39; ACVR1B; HRAS; TRIB3; PINK1; SEPT4; TNFRSF12A; PHLDA3; DAXX; MADD; TRAP1; TRAF7; MNT; UACA; STK25; XPA; CHEK2; TGFB1; UNC5B; ERP29; RNF41; ING5; PTPN1; CHAC1; TP53; TFAP4; BCL2A1; TNFRSF25; MYBBP1A; DPF2; PPP2R1A; MKNK2; PPM1F; ATM; EPHA2; RET; TNFRSF10B; DIABLO; RHOT2; ABL1; MAP3K5; SART1; HTRA2; TICAM1; NOC2L; TRADD; SYVN1; MAPK7; PML; TNFRSF6B; APAF1; ZNF346; SRC; GABARAP; DAB2IP; LGALS3; CLU; NOL3; BCAP31; CTNNB1; HSPA1A; BAD; PPP1R15A; DIDO1; WWOX; PPIF; BRCA1; CTTN; PLAUR; RIPK1; BMPR1B; MAP2K5; P2RX4; P2RX7; CEBPB; PLEKHF1; BID; GCLC; FASTK; DAP; YWHAB; MSX1; TIMM50; TERT; GRINA; TRAF2; ANXA6; PDIA3; PDIA2; NLE1; FXN; LMNA; BCL10; E2F1; PPP1CA; CASP9; GSTP1; MAPT; ATF4; DEDD2; SLC9A3R1; ATF3 |
| GO:0060322 | 3 | head development | 3,11E-05 | 0,00031 | SMARCC2; PGAP1; SHARPIN; AGER; BOK; ANP32B; SDF4; CALM3; RARG; ZNF148; DNMT1; NTRK2; THRA; DCX; WDR62; ZIC2; ATP6V0D1; PLXNA2; BRCA1; SLC38A3; STRA6; AHI1; LDB1; PHGDH; SETD2; FGFR2; PAFAH1B1; BMP2; ARID1A; ROBO2; H2AFX; CDON; BMPR2; AK2; TACC2; PLCB1; RAD1; SEPT4; NDRG4; PLXNB2; CHD7; TULP3; UBE3A; MKS1; DISC1; PRKG1; NF2; CDK5R2; PPP1R9B; EPHB2; SIRT2; CSK; RAB3GAP1; DIXDC1; HERC1; EFHC1; SEZ6L2; ARNT2; KIF3A; MAOB; STK36; IL11RA; HES1; MCPH1; ZNF335; VAX2; SPHK2; NODAL; ATM; H2AFY2; SMAD1; SRGAP2; SMO; ABL1; ATP6AP2; CD9; HTRA2; TSC1; MDK; FZD4; RAB18; MAPK1; NRP2; SEZ6; CITED1; DLC1; APAF1; ZNF430; FBXO45; SRC; SRF; NAPA; PYGO2; GATA2; NAGLU; ASCL1; DAB2IP; ATRN; SRR; ENO3; QARS; FAIM2; PLXNA3; TACC1; LRP8; TACC3; BASP1; CHRD; CDK5; CDK5RAP2; CDK5RAP3; CDK5RAP1; XAB2; CDC42; TGFB1; CTNNB1; MAP2K2; BAD; NNAT; SEMA6D; SPTBN2; ALDH1A2; ALDH1A3; AXIN1; BID; KLHL17; KCNK3; MAPKAP1; ETS1; SLIT3; ABCB6; NRG1; MSX1; ANKRD11; MECP2; MBD3; CNP; NRGN; NR4A3; ABR; FPGS; NDEL1; SSBP3; CTNS; CTNNA2; NFIB; E2F1; IFT172; AARS; DRD2; SYNGR3; NOTCH3; SCRIB; SECISBP2; MAPT; CHD8; ATF5; SLC1A2 |
| GO:0007420 | 3 | brain development | 6,22E-05 | 0,00057 | SMARCC2; PGAP1; SHARPIN; AGER; BOK; ANP32B; SDF4; CALM3; ZNF148; DNMT1; NTRK2; THRA; NAPA; WDR62; ZIC2; ATP6V0D1; PLXNA2; BRCA1; SLC38A3; AHI1; LDB1; PHGDH; SETD2; FGFR2; PAFAH1B1; BMP2; ARID1A; ROBO2; H2AFX; CDON; BMPR2; AK2; TACC2; PLCB1; RAD1; SEPT4; NDRG4; PLXNB2; CHD7; TULP3; UBE3A; DISC1; PRKG1; NF2; CDK5R2; PPP1R9B; EPHB2; SIRT2; CSK; RAB3GAP1; DIXDC1; HERC1; EFHC1; SEZ6L2; ARNT2; KIF3A; MAOB; STK36; HES1; MCPH1; ZNF335; VAX2; NODAL; ATM; H2AFY2; SMAD1; SRGAP2; SMO; ABL1; CD9; HTRA2; TSC1; MDK; FZD4; RAB18; CTNNA2; SEZ6; CITED1; DLC1; APAF1; ZNF430; FBXO45; SRC; SRF; DCX; PYGO2; GATA2; NAGLU; ASCL1; DAB2IP; ATRN; SRR; ENO3; QARS; FAIM2; PLXNA3; TACC1; LRP8; TACC3; BASP1; CHRD; CDK5; CDK5RAP2; CDK5RAP3; CDK5RAP1; XAB2; CDC42; SPHK2; CTNNB1; BAD; NNAT; SEMA6D; SPTBN2; ALDH1A2; ALDH1A3; AXIN1; BID; KLHL17; KCNK3; MAPKAP1; ETS1; SLIT3; ABCB6; NRG1; MSX1; MECP2; MBD3; CNP; NRGN; NR4A3; ABR; FPGS; NDEL1; SSBP3; CTNS; NRP2; NFIB; E2F1; IFT172; AARS; DRD2; SYNGR3; NOTCH3; SCRIB; SECISBP2; MAPT; CHD8; ATF5; SLC1A2 |
| GO:0048588 | 3 | developmental cell growth | 0,000166 | 0,001442 | CTTN; UNC13A; BCL11A; CTDP1; MEGF8; L1CAM; ABL1; SEMA4B; PARK2; TNFRSF12A; SEMA6C; SEMA4F; ARIH2; IMPACT; PLXNB1; CRABP2; DISC1; SEMA4G; CPNE1; NTRK3; PRMT2; GOLGA4; DCX; NRG1; SLIT3; SEMA6A; MAP1B; ULK1; SRF; CLASP2; CAMK2D; PRKCZ; NDEL1; ANAPC2; PAFAH1B1; NRP2; PLXNC1; RAB21; AKAP6; RARG; FN1; PLXNA3; PLXNA2; PLXNA1; GSK3A; SEMA6D; RND2; BMPR2; CDK5; MAPT; ISLR2 |
| GO:0050793 | 3 | regulation of developmental process | 0,00018 | 0,001522 | RNF10; FKBP4; ANP32B; L1CAM; DBN1; SEMA4B; SEMA4F; SEMA4G; DISC1; CAMK1; CDC42SE2; APBB1; DMPK; THY1; WDR1; ARHGEF18; ARHGEF19; SLC45A3; CRB2; SMARCD3; OPA1; GATA2; AKAP6; CCND1; PLXNA1; TNFSF13; CAPZB; ELAVL1; ACVR1B; ITGA3; NF2; NR1H2; PDLIM7; COL4A2; VEGFB; GRN; GDI1; PLXNC1; RPS6KA1; CSNK1A1; VASH1; PSMD11; PSMD13; FBXO31; FLOT2; HES1; DEAF1; SMAD1; RET; SRGAP2; SMO; PRKCSH; FOXM1; AKAP13; THRA; HTRA2; ARF6; HMGB3; SEZ6; BRWD3; AP3D1; ATOH8; LAMA4; LAMA5; MECP2; RXRB; OPRM1; SUPT6H; F3; TRIOBP; FAM20C; CDK5RAP2; CDK5RAP3; CDK5RAP1; PRKD2; CDC42; BRCA1; RBM4; ANKH; HMGB1; CPEB1; CTNNB1; BAD; HAND2; MITF; BIN1; PGF; TOB2; CLOCK; RAI1; SLIT3; CNOT2; PSMC4; PSMC5; CNTF; E4F1; ABR; FXN; SUFU; TTBK2; PLEKHO1; RAB21; PARD3; E2F1; CALR; RND2; MBD1; CTNNBIP1; STK4; PPP1R13L; WDR77; TRIM11; TRIM16; PPP2R3A; IKBKB; SFMBT1; PTK2B; SCRT1; FBLN1; CLSTN1; CHRFAM7A; RARG; PSMB10; ARHGEF2; ADRA2C; RELA; HMG20B; CDKN2A; NCKIPSD; ACIN1; RAMP2; LDB1; PALM; SSH3; SNAI1; PAFAH1B1; VAT1; FN1; RFX3; CDON; DIXDC1; FOXP1; TRIB3; PINK1; NEK6; YPEL3; TULP3; CCNE1; PALMD; STK25; GPR161; TCF3; ENG; PHLDB1; SARM1; IRF1; IRF7; MEF2C; PFN1; ISLR2; GAS6; EFNB2; PDGFRB; HDAC3; ZNF335; HDAC5; TNFSF12; HDAC9; NODAL; AHI1; SPRY1; PRPF40A; MMP11; GAS2L1; MYH9; TRADD; CYB5D2; NLGN2; CLIC1; PAF1; ASCL1; CELSR3; COL5A2; DLL3; CLU; TRIM62; PDCD4; ARNT; KEAP1; BASP1; CDK5; CDK9; NEU1; MAD2L2; TGFB1; PHB2; USP2; AGRN; MEGF8; FGD1; SEMA6D; PRKX; SEMA6C; SEMA6A; IMPACT; CEBPB; CEBPA; AXIN1; NDRG4; NLGN1; KIF13B; DVL3; ETS1; SUZ12; ARHGDIA; VASN; DAB2IP; CDC42EP2; NBR1; CDC42EP4; PRKCZ; AP2A2; KRIT1; LMNA; CTNNA2; FEZ1; KATNB1; TLX2; NOTCH3; PTCH2; TGIF2; AGER; FMNL3; FMNL1; ATP6AP1; PARK2; EIF4ENIF1; CUL7; CTSH; FLCN; VRK3; PRMT5; HMGCR; FGFR2; ROR2; MARCH5; ADCY6; INPP5E; INPP5F; GLG1; PQBP1; KIT; BMPR2; CARM1; FBLIM1; TNFRSF12A; DAXX; AP2S1; CTTN; MKS1; TMEM79; MAFG; EPHB2; PLCB1; MAP1B; MAN2A1; LGMN; DDR2; MAP3K5; CD276; PPP2R5B; H2AFY2; CTDP1; TBX2; OLFM2; ARHGEF1; POFUT2; ANAPC2; STAT3; ZHX3; CD83; SRC; SRF; ATRN; FLT3LG; LGALS3; GPR68; LRP4; PLXNA3; PLXNA2; LRP8; PPP1R15A; CHRD; PPIB; PPP2R1A; IL1RAPL1; CORO1B; MAP2K2; BMPR1B; CORO1A; MAP2K5; BCL9L; RAP1GAP; MLLT3; GAK; NRG1; TERT; FLOT1; ESRRA; PKN1; FLNA; ALDOA; DRD2; MAPT; CPNE1; TIMP1; ISL2; AKT1; NOTCH4; CREBL2; S100A13; BOC; GSN; SMAP1; HIF1AN; NTRK1; NTRK2; NTRK3; CSNK1D; CSNK1E; GOLGA4; PTBP1; SOD2; BAMBI; CACNA2D2; NPHP3; BMP2; BMP1; PPP2CA; MAGED1; EIF4G1; ROBO2; GSK3A; HYAL1; HLA-B; NFKBIA; HLA-G; TMEM119; RNH1; LUC7L; PLXNB2; PLXNB3; PLXNB1; CHD7; PREX1; ASXL1; ASXL2; TFE3; TGFB1I1; IFT122; LZTS1; SIRT2; RNF41; DAG1; NELL1; SART1; FAM57B; GAMT; EHD2; EPHA2; MAPK12; ABL1; FZD2; FZD4; BTG1; AP2M1; MAPK1; MAPK7; PML; CITED1; DLC1; PYGO2; HMGA1; PARVB; CHRNA7; SERPINF1; UNC13A; FADS1; RBM19; RGS14; ID3; STAT5B; PRKACA; DGUOK; BCL11A; RIPK1; P2RX7; CRABP2; SLC9A3R1; KCNK2; MSX1; CLASP2; CLASP1; ATF5; GPC3; CFL1; NDEL1; SSBP3; USP19; NFIB; RCC2; IL27RA; PTPRF; GNA12; GNA11; PTPRO; PTPRM |
| GO:0048513 | 3 | animal organ development | 0,000256 | 0,002045 | SMARCC2; PGAP1; IFT20; FKBP4; CD8A; ANP32B; MFAP2; SEMA4B; LEMD2; SEMA4F; SEMA4G; SDF4; DISC1; TAZ; NAPA; THY1; PPHLN1; ZNF689; SLC38A3; CAMK2D; ARHGEF19; CRB2; APRT; SMARCD3; GATA2; AKAP6; CCND1; MAX; LRP8; MMP16; EVL; ACVR1B; CDKN1C; NOVA1; ITGA3; RAD1; SFXN1; UROD; CSK; SOX12; JAM3; ZIC2; NF2; ZNF513; DNASE1L2; MME; VEGFB; WDR77; SLC39A3; ARNT2; RPS6KA1; RPS6KA2; MTHFD1L; PSMD11; PSMD13; PDE6B; FLOT1; HES1; FLNB; EIF4A1; CRIP2; ACP6; SPHK2; TLE3; DEAF1; SMAD6; SMAD1; RET; SRGAP2; SMO; POLE; PRKCSH; AKAP13; NPHP4; POLM; IGF2R; TSC2; EFHC1; HTRA2; TSC1; HMBS; MDK; ARF6; ANKH; SEZ6; AP3D1; BSG; LAMA5; CTF1; MECP2; NASP; SLC27A4; FAIM2; RAB11FIP4; AK2; FAM20C; ABT1; CDK5RAP2; CDK5RAP3; CDK5RAP1; NBEAL2; CDC42; BRCA1; IGSF8; TTC7A; GNB1; HMGB1; TMIE; CTNNB1; BAD; HAND2; MITF; GLG1; BIN3; CAD; PGF; SLC37A4; PFN1; MAD1L1; DCLRE1C; SLIT3; ETNK2; HOMER1; ATP6V1B1; SIPA1L3; ANKRD11; PSMC4; PSMC5; CNTF; ARIH2; ABR; FPGS; SUFU; PRMT5; NRP2; E2F4; E2F1; BNC2; IFT172; CALR; TOB2; MBD1; MBD3; CTNNBIP1; STK4; PPP1R13L; CMTM7; SLC1A2; TUSC2; TRIM16; PPP2R3A; SF1; DHTKD1; SFMBT1; PTK2B; ZNF335; VAX2; ASB1; RARG; ADAMTS2; NFE2L1; CLOCK; RELA; HMG20B; CDKN2A; STRA6; ACIN1; RAMP2; LDB1; PHGDH; SNAI1; PAFAH1B1; TUFT1; THOC5; KIAA1161; ARID1A; RFX3; CDON; DIXDC1; WFS1; FOXP1; NSDHL; CRELD1; SLC29A1; TULP3; CCNE1; TIMELESS; NEK8; COL18A1; PRKG1; PPP1R9B; TCF3; ENG; MRAS; TK1; PHLDB1; IRF1; IRF7; IRF6; MEF2C; MAOB; GAS6; EFNB2; PDGFRB; EFNB1; HDAC5; PSMB10; HDAC9; NODAL; EIF2B4; ATM; AHI1; SPRY1; SETD2; AKR1C3; MLF1; USF2; IFT140; GAS2L1; MYH9; VKORC1; TRADD; FANCG; RAB18; ZNF430; FANCA; RAB13; FBXW4; PHACTR4; FGD1; KLHL17; NAGLU; ASCL1; CELSR3; COL5A2; NPY2R; ENO3; BBX; CLU; TRIM62; ARNT; TFIP11; MFN2; BASP1; CDK5; NPFF; SH3PXD2A; XAB2; MAD2L2; TGFB1; GTF3C5; PHB2; ERCC1; ERCC2; MEGF8; LYL1; SEMA6D; PRKX; SMARCA4; SEMA6C; SEMA6A; CEBPB; CEBPA; LOXL3; AXIN1; ALX3; BID; DVL3; ETS1; NARFL; ABCB6; EBP; PAF1; VASN; HEG1; DAB2IP; NR4A3; NBR1; RDH10; NLE1; PRKCZ; AP2A2; LMNA; CTNS; CTNNA2; NOTCH4; ABI1; NRIP1; NOTCH3; SCRIB; GSTP1; DLL3; EEF2; PTCH2; TGIF2; ADD2; AGER; MYL6; CALM3; RDH13; DNMT1; ATP6AP1; THRA; DCX; WDR62; LSR; ATP6V0D1; CUL7; CLN8; CTSH; FLCN; TRIM28; HOXD9; CLCN2; LRRC6; KCNAB2; HMGCR; RFNG; SIDT2; FGFR2; ROR2; DNAJA3; PCYT1B; CNN2; KIT; FREM1; UTRN; BMPR2; TACC2; ZBTB24; EMD; AES; GBA; HRAS; CARM1; KIF26B; SEPT4; NDRG4; AP2S1; MKS1; TMEM79; JUP; CDK5R2; EPHB2; PLCB1; STK40; VGF; EPN1; MAN2A1; CCM2; HERC1; DNER; ABCA12; SCUBE1; DDR2; DDR1; EIF4EBP1; STK36; SIX6; MKNK2; CRYGS; H2AFY2; CTDP1; TBX2; PLCE1; SLC39A1; DGCR6; POFUT2; CD9; POFUT1; SNX17; APH1A; STAT3; CD83; NUMA1; MPST; FHL2; FHL3; FOSL2; USP2; FBXO45; SRC; SRF; ATRN; SRR; SMTN; FLT3LG; LGALS3; QARS; GPR68; MAEA; LRP4; GALNT11; PLXNA3; PLXNA2; PLXNA1; CHRD; COL1A2; PPIB; STIM1; CD276; PSAP; MOSPD3; MAP2K2; BMPR1B; AEBP1; MAP2K5; GPD2; AACS; BCL9L; SPTBN2; ALDH1A2; ALDH1A3; RAP1GAP; WASF2; MLLT3; MAPKAP1; VPS33A; ATG5; NRG1; ITGB1BP2; GAA; ESRRA; NRGN; PKN1; TAGLN; FMN1; INPPL1; PPP1CA; DRD2; CASP9; BAZ1B; BCL9; SECISBP2; MAPT; SLC9A3R2; BCL2L1; TIMP1; ISL2; DHCR7; AKT1; BOK; SCO2; SMAP1; ZNF148; DGCR2; CIAPIN1; NTRK1; NTRK2; NTRK3; CAPN5; CAPN1; PIR; SOD2; BAMBI; NPHP3; PCGF2; BMP2; BMP1; HYAL3; HYAL2; HYAL1; ROBO2; GSK3A; PPP2CA; CCNF; GRB2; MAGED1; H2AFX; NFKBIA; HLA-G; TMEM119; LUC7L; MAPKAPK2; PLXNB2; CHD2; CLASP1; CHD7; PREX1; ASXL1; CHD8; UBE3A; THBS3; TFE3; TGFB1I1; IFT122; LZTS2; DCHS1; SIRT2; NR4A1; GNPNAT1; RAB3GAP1; RNF41; GYS1; HEXIM1; DAG1; NELL1; SART1; SEZ6L2; KIF3A; PEX7; SDC1; NCL; GPC3; MCPH1; GAMT; RAD23B; FJX1; EPHA2; GON4L; MAPK12; ABL1; UCP2; FZD2; FZD4; AP2M1; MAPK1; FASN; HLA-B; PML; CITED1; DLC1; APAF1; LRRC8A; PYGO2; QDPR; STOX2; PTPRU; SERPINF1; PLCD3; PLCD1; FHOD3; ITFG2; TACC1; PKD1; TACC3; ID3; STAT5B; SREBF1; WWOX; RPS14; RIPK1; NNAT; P2RX7; PRPS1; PLOD3; SLC9A3R1; KCNK2; KCNK3; MSX1; LRP10; CLASP2; CNP; PRPS1L1; ATF5; PLXNB1; NDEL1; ARMC6; SSBP3; USP19; SERPINH1; NFIB; ACTN1; SLC40A1; AARS; UNC45A; SYNGR3; PDE2A; SHARPIN; GNA11; PTPRO; PTPRM; ATF3 |
| GO:0060560 | 3 | developmental growth involved in morphogenesis | 0,000266 | 0,002071 | TGFB1; CTTN; PPP2R3A; FMN1; KIF26B; TBX2; MEGF8; UNC13A; L1CAM; ABL1; TRIM28; SEMA4B; PARK2; TNFRSF12A; SEMA6C; SEMA4F; SEMA6A; IMPACT; PLXNB1; CRABP2; FGFR2; CPNE1; NTRK3; DISC1; GOLGA4; BIN3; DCX; NRG1; SLIT3; SEMA4G; MAP1B; ULK1; SRF; CLASP2; BCL11A; RDH10; PRKCZ; NDEL1; ANAPC2; PAFAH1B1; NRP2; PLXNC1; RAB21; RARG; FN1; PLXNA3; PLXNA2; PLXNA1; SPRY1; SEMA6D; RND2; BMPR2; CDK5; MAPT; ISLR2 |
| GO:0021915 | 3 | neural tube development | 0,000673 | 0,004611 | NODAL; EPHA2; SMO; TSC2; ALDH1A2; TSC1; PLXNB2; FZD2; IFT172; RARG; TULP3; DEAF1; ATP6AP2; MKS1; DVL3; IFT122; GPR161; DLC1; APAF1; DCHS1; PHACTR4; IFT140; STK4; PHGDH; SUFU; SSBP3; SETD2; TMEM107; BCL10; KIF3A; NPHP3; PLXNA2; PKD1; MTHFD1L; SCRIB; PFN1; CHRD; PRKACA; PLOD3; HES1; ARID1A |
| GO:0048869 | 3 | cellular developmental process | 0,000694 | 0,004706 | SMARCC2; RNF10; AP1M1; FKBP4; CD8A; ANP32B; L1CAM; DBN1; TYK2; LEMD2; SEMA4F; SEMA4G; SDF4; PALMD; CAMK1; CDC42SE2; APBB1; DMPK; THY1; DHX9; ZNF689; DIAPH2; CAMK2D; MUC1; CAMK2G; MTCH1; SLC45A3; CRB2; SMARCD3; ATF5; OPA1; GATA2; AKAP6; DBNL; CCND1; CTBP1; PLXNA1; CAPZB; EVL; MMP11; ACVR1B; CDKN1C; ITGA3; PUM1; GTF3C5; SFXN1; SEMA4B; CSK; GAP43; SOX12; DLD; ZIC2; NF2; NR1H2; PDLIM7; DNASE1L2; MME; NFASC; COL4A2; GRN; CHST3; GDI1; PLXNC1; RPS6KA1; RPS6KA2; CSNK1A1; VASH1; TNFSF12; PSAP; FLOT1; FLOT2; MYBBP1A; FLNA; FLNB; PRDM8; ACP6; DNMT1; SDCCAG8; STIM1; DEAF1; SMAD6; SMAD1; RET; SRGAP2; SMO; PRKCSH; FOXM1; AKAP13; NPHP4; POLM; MAP3K5; HTRA2; TSC1; HMBS; MDK; NR2F6; ARF6; ELAVL3; SEZ6; BRWD3; AP3D1; BSCL2; ATOH8; PTPRU; LAMA4; LAMA5; CTF1; MECP2; EIF4ENIF1; OPRM1; SUPT6H; FAIM2; MARCH5; ZFYVE27; TRIOBP; FAM20C; TBCC; ABT1; CDK5RAP2; CDK5RAP3; CDK5RAP1; NBEAL2; CDC42; RBM4; PPHLN1; HMGB3; HMGB1; CPEB1; CTNNB1; TEAD3; BAD; HAND2; MITF; BIN1; BIN3; PGF; RGS20; WDR1; SLC37A4; TOB2; ERH; DCLRE1C; ARHGEF18; NEO1; SLIT3; HOMER1; CNOT2; SIPA1L3; TTLL5; CNTF; ITGB5; ARIH2; FEZ1; SUFU; TTBK2; MMP24; PRMT5; NRP2; RAB21; E2F4; E2F1; IFT172; CALR; IER2; RND2; ANK3; MBD1; CTNNBIP1; STK4; PPP1R13L; CMTM7; EEF2; TRIM11; FRYL; TUSC2; EFNB3; SHARPIN; PPP2R3A; SF1; IKBKB; SFMBT1; PTK2B; HPS6; SCRT1; FBLN1; USP21; VAX2; SETD2; RARG; TSSK2; NFE2L1; CLOCK; ARHGEF2; JAM3; ADRA2C; RELA; HMG20B; CDKN2C; CDKN2A; NCKIPSD; ACIN1; RAMP2; LDB1; PALM; SSH3; PHGDH; FBLIM1; THOC2; SNAI1; PAFAH1B1; THOC5; KIAA1161; RAB27A; FN1; ARID1A; RFX2; RFX3; CDON; DIXDC1; PAK1; SNX1; FOXP1; MYCBPAP; ESRRA; NEK6; NEK3; YPEL3; TULP3; CCNE1; CBX2; COL18A1; STK25; PRKG1; ANAPC2; TUBD1; PPP1R9B; TCF3; ENG; MRAP; SHB; PHLDB1; SARM1; TUBB2B; IRF1; IRF7; IRF6; MEF2C; DTYMK; ALKBH5; ISLR2; GAS6; EFNB2; PDGFRB; HDAC3; EFNB1; HDAC5; PSMB10; HDAC9; NODAL; EIF2B4; ATM; AHI1; STXBP1; PHB; AKR1C1; AKR1C3; MLF1; IFT140; PLEKHO1; GAS2L1; MYH9; PRPF40A; FANCG; CYB5D2; AAMP; SLC39A3; RAB13; ENAH; PHACTR4; FGD1; CLIC1; NAGLU; ASCL1; CELSR3; COL5A2; DLL3; SND1; CLU; TRIM62; PDCD4; TDRKH; LLGL1; PARD3; KEAP1; BASP1; CDK5; CDK9; SH3PXD2A; NEU1; MAD2L2; TGFB1; ERCC1; ERCC2; PHF19; AGRN; MEGF8; NDRG1; LYL1; SEMA6D; PRKX; SMARCA4; SEMA6C; POMZP3; IMPACT; CEBPB; CEBPA; LOXL3; AXIN1; NDRG4; NLGN1; KIF13B; ARNT; NARFL; SUZ12; ARHGDIA; UCN; PAF1; ASF1B; VASN; HEG1; DAB2IP; NR4A3; NBR1; RDH10; CDC42EP4; CPLX2; PRKCZ; LMNA; CTNNA2; NOTCH4; ABI1; TLX2; NOTCH3; SCRIB; GSTP1; CUL4B; WDR77; NCDN; PTCH2; TGIF2; ACADVL; AGER; FADS1; FMNL3; FMNL1; ATP6AP1; MPP5; PARK2; DCX; WDR62; LSR; CUL7; CLN8; SHC4; FLCN; TRIM28; HOXD9; VRK3; PRMT2; CLCN2; PRMT7; CCDC78; KCNAB2; HMGCR; RFNG; SIDT2; FGFR2; ROR2; SETD6; IQCG; DNAJA3; ADCY6; INPP5E; INPP5F; GLG1; KIF5A; KIF5C; PQBP1; KIT; BMPR2; EPC1; ZNF335; HDAC10; CHAC1; ZBTB24; EMD; HRAS; CARM1; KIF26B; SEPT4; TNFRSF12A; ETS1; ZSCAN2; DAXX; CTTN; MKS1; TMEM79; CDK5R2; MAFG; CHEK2; EPHB2; PLCB1; MAP1B; UNC5A; UNC5B; WWOX; TUBB3; MAN2A1; CCM2; HERC1; DTNBP1; DNER; ABCA12; SCUBE1; TP53; TOLLIP; DDR2; CD276; SEMA6A; PPP2R5B; RASIP1; H2AFY2; CTDP1; TBX2; OLFM2; FBXO9; ARHGEF1; POFUT2; CD9; H2AFX; EHMT2; STAT3; ZHX3; CD83; NUMA1; CSNK1D; FHL2; DHTKD1; FOSL2; BCL7B; IFT20; FBXO45; SRC; DHFR; SRF; EFNA4; MAST2; FLT3LG; LGALS3; PIR; GPR68; RPS6KA5; MAEA; LRP4; PLXNA3; PLXNA2; LRP8; GSTM3; CHRD; PDE4D; GPSM1; PPP2R1A; IL1RAPL1; PSMD11; SPATA20; CORO1B; MAP2K2; BMPR1B; CORO1A; MAP4; AACS; BCL9L; SPTBN2; ALDH1A2; RAP1GAP; WASF2; CBR1; VPS33A; GAK; RGS2; ATG5; NRG1; TERT; FBXO31; SMARCB1; SUV39H1; PIGT; HES1; ALDOA; ALDOC; TAGLN; CASP6; SQSTM1; PACRG; DRD2; CASP9; BCL9; SECISBP2; MAPT; RDH13; COL6A1; SLC9A3R1; BCL2L1; ISL2; GLRX2; DHCR7; AKT1; LRFN1; BOK; CREBL2; DISC1; S100A13; BOC; GSN; SMAP1; HIF1AN; GRK5; NTRK1; NTRK2; NTRK3; NAV1; ARHGAP22; CAPN5; CSNK1E; GOLGA4; PTBP1; SOD2; SYNCRIP; BAMBI; VAPA; CACNA2D2; NXN; NPHP3; BMP2; BMP1; CSPG5; PPP2CA; HYAL2; EIF4G1; ROBO2; GSK3A; GRB2; ELP3; ROBO3; HLA-B; NFKBIA; HLA-G; TMEM119; ULK1; CCHCR1; PLXNB2; PLXNB3; CHD2; CLASP1; CHD7; PREX1; ASXL1; THRA; ASXL2; MNT; TFE3; CDC42EP2; TGFB1I1; GBA2; NUP62; LZTS1; DCHS1; SIRT2; NR4A1; CDC25B; RNF41; DAG1; SLC26A6; NELL1; SART1; NAPA; KIF3A; FAM57B; NLE1; PEX7; SDC1; HPS4; GPC3; EHD2; EPHA2; GON4L; MAPK12; ABL1; FZD2; FZD4; BTG1; POLDIP2; TEC; MAPK1; FASN; MAPK7; PML; CITED1; DLC1; APAF1; PAQR8; SLC7A5; LRRC8A; PYGO2; FMN1; RCC2; HMGA1; FEV; PARVB; SERPINF1; UNC13A; TSNAXIP1; MAPK8IP2; RGS14; FHOD3; ITFG2; ID3; RGS19; STAT5B; SREBF1; PRKACA; DGUOK; CEP152; FBL; KATNB1; RPS14; TRIB3; BCL11A; RIPK1; NNAT; CD63; ELF4; P2RX7; CRABP2; PLOD3; CPNE1; MSX1; ULK3; DNMT3A; TMEM106B; CLASP2; CNP; USP13; PLXNB1; RTF1; CFL1; NDEL1; ARMC6; SSBP3; SERPINH1; NFIB; ACTN1; TRIM16; UNC45A; PDE2A; PTPRF; GNA12; SRA1; GNA11; PTPRO; PTPRM; ATF3; GSTK1 |
| GO:0048468 | 3 | cell development | 0,000941 | 0,006136 | RNF10; FKBP4; L1CAM; DBN1; SEMA4B; SEMA4F; SEMA4G; CAMK1; APBB1; THY1; DIAPH2; CAMK2D; MTCH1; SLC45A3; IQCG; OPA1; GATA2; AKAP6; DBNL; PLXNA1; CAPZB; EVL; CDKN1C; ITGA3; GAP43; DLD; LSR; NF2; DNASE1L2; NFASC; GRN; CHST3; ANAPC2; PLXNC1; RPS6KA2; RPS6KA5; FLOT1; FLNA; FLNB; PRDM8; DEAF1; RET; SRGAP2; SMO; PRKCSH; AKAP13; NPHP4; HTRA2; NR2F6; ARF6; SEZ6; LAMA5; CTF1; MECP2; OPRM1; ZFYVE27; TRIOBP; FAM20C; CDK5RAP2; CDK5RAP3; CDK5RAP1; NBEAL2; CDC42; CTTN; DHFR; HMGB1; CPEB1; CTNNB1; BAD; HAND2; BIN1; BIN3; WDR1; NEO1; SLIT3; HOMER1; SIPA1L3; TTLL5; CNTF; ARIH2; NRP2; RAB21; PARD3; CALR; KIF13B; ANK3; MBD1; SMARCD3; TRIM11; FRYL; TUSC2; EFNB3; SIDT2; IKBKB; PTK2B; EFNB1; SCRT1; FBLN1; USP21; RARG; ARHGEF2; ADRA2C; RELA; HMG20B; NCKIPSD; RAMP2; PALM; SSH3; PHGDH; THOC2; PAFAH1B1; KIAA1161; FN1; RFX2; RFX3; CDON; DIXDC1; PAK1; FOXP1; NEK3; ENG; DISC1; STK25; PRKG1; PPP1R9B; TCF3; JAM3; SARM1; IRF6; MEF2C; ISLR2; EFNB2; PDGFRB; ZNF335; CLOCK; HDAC9; NODAL; EIF2B4; ATM; AHI1; STXBP1; IFT140; MYH9; FANCG; CYB5D2; RAB13; ENAH; PHACTR4; NLGN1; NAGLU; ASCL1; CELSR3; DLL3; CLU; LLGL1; CDK5; NEU1; TGFB1; ERCC1; ERCC2; AGRN; MEGF8; NDRG1; SEMA6D; SMARCA4; SEMA6C; SEMA6A; IMPACT; CEBPA; AXIN1; NDRG4; RND2; ARHGDIA; UCN; HEG1; DAB2IP; PRKCZ; LMNA; CTNNA2; FEZ1; KATNB1; TLX2; NOTCH3; SCRIB; GSTP1; CUL4B; NCDN; TGIF2; AGER; ATP6AP1; MPP5; PARK2; DCX; EIF4ENIF1; CUL7; HOXD9; PRMT2; PRMT5; SETD2; FGFR2; ADCY6; INPP5E; INPP5F; KIF5A; KIF5C; PQBP1; KIT; BMPR2; HDAC10; HRAS; CARM1; KIF26B; SEPT4; TNFRSF12A; MKS1; TMEM79; EPHB2; MAP1B; UNC5B; UNC5A; TUBB3; MAN2A1; CCM2; HERC1; DTNBP1; DNER; PPP2R5B; CTDP1; ARHGEF1; CD9; GDI1; STAT3; E2F4; FHL2; FOSL2; IFT20; FBXO45; SRC; SRF; EFNA4; GPR68; MAEA; LRP4; PLXNA3; PLXNA2; LRP8; GSTM3; PDE4D; PPP2R1A; IL1RAPL1; MAP2K2; BMPR1B; MAP4; SPTBN2; ALDH1A2; RAP1GAP; WASF2; VPS33A; GAK; ATG5; NRG1; POMZP3; FBXO31; COL18A1; HES1; PACRG; DRD2; CASP9; RDH10; SECISBP2; MAPT; RDH13; CPNE1; BCL2L1; ISL2; AKT1; LRFN1; BOC; GSN; NTRK1; NTRK2; NTRK3; GOLGA4; SOD2; VAPA; CACNA2D2; BMP2; BMP1; EIF4G1; ROBO2; GSK3A; GRB2; ROBO3; FMN1; PLXNB2; PLXNB3; PLXNB1; CHD7; PREX1; NR4A3; GBA2; LZTS1; SIRT2; CDC25B; EHMT2; DAG1; SLC26A6; KIF3A; SDC1; VAX2; EPHA2; ABL1; FZD2; FZD4; MAPK1; PAQR8; PYGO2; ACTN1; FEV; SERPINF1; UNC13A; MAPK8IP2; RGS14; FHOD3; NOTCH4; PRKACA; DGUOK; ABI1; BCL11A; TSSK2; CRABP2; PLOD3; SLC9A3R1; ULK1; TMEM106B; CLASP2; CNP; PTPRO; CFL1; NDEL1; SERPINH1; NFIB; RCC2; PDE2A; PTPRF; ATF5; PTPRM |
| GO:0021987 | 3 | cerebral cortex development | 0,001716 | 0,010298 | MCPH1; SRGAP2; SMO; BAD; TSC1; MDK; NTRK2; DISC1; DCX; CDK5R2; WDR62; PPP1R9B; FBXO45; PLCB1; ASCL1; DAB2IP; NDEL1; EFHC1; PAFAH1B1; NRP2; CTNNB1; TACC1; LRP8; TACC3; H2AFX; XAB2; CDON; CDK5; DIXDC1; TACC2 |
| GO:0097194 | 3 | execution phase of apoptosis | 0,002163 | 0,01243 | BCL2L1; CLSPN; HMGB1; BCAP31; AKT1; BOK; KPNB1; MADD; HTRA2; DLC1; APAF1; SIRT2; CDKN2A; ACIN1; ENDOG; DFFA; DFFB; CASP6; TP53; CAPN10; CDK5RAP3; DEDD2; SHARPIN |
| GO:0035295 | 3 | tube development | 0,00466 | 0,024752 | CHD8; AGER; DHCR7; RARG; ADAMTS2; GATA2; DEAF1; ATP6AP2; CTSH; STRA6; SMAD1; STK4; PHGDH; SETD2; FGFR2; TBX2; BMP2; MAGED1; ARID1A; ROBO2; KIT; BMPR2; PAK1; FOXP1; ITGA3; FMN1; KIF26B; NDRG4; PLXNB2; CHD7; ASXL1; TULP3; TIMELESS; MKS1; SREBF1; IFT122; GPR161; DCHS1; STK40; ENG; IFT140; MAN2A1; CCM2; DAG1; TK1; KIF3A; ABCA12; SDC1; DDR1; NPHP3; MEF2C; PFN1; EIF4EBP1; HES1; EFNB2; RASIP1; SDCCAG8; NODAL; SMAD6; EPHA2; RET; SMO; SPRY1; SLC4A2; THRA; TSC2; TSC1; FZD2; TMEM107; MAPK1; PML; CITED1; DLC1; APAF1; ATOH8; SRC; SRF; LAMA5; ASCL1; DAB2IP; AHI1; PLXNA2; PKD1; BASP1; CHRD; PRKACA; PRKD2; CDC42; TGFB1; MTHFD1L; NUMA1; PHB2; HMGB1; CTNNB1; MAP2K2; MEGF8; HAND2; PRKX; ALDH1A2; PGF; CEBPA; PLOD3; DVL3; PHACTR4; HEG1; NR4A3; GPC3; SUFU; SSBP3; BCL10; NFIB; PPP1CA; NOTCH4; IFT172; SCRIB; RDH10; CTNNBIP1 |
| GO:0048066 | 3 | developmental pigmentation | 0,005433 | 0,028334 | RAB27A; CD63; HPS4; AP1M1; KIT; MEF2C; USP13; VPS33A; HPS6; GNA11; MITF; SOD2; CITED1; ATP6AP2; AP3D1 |
| GO:0061061 | 3 | muscle structure development | 0,010374 | 0,048507 | IFT20; PPP2R3A; MYL6; AKT1; BOC; LEMD2; CAMK1; HIF1AN; TAZ; THRA; DMPK; PTBP1; HMG20B; ZNF689; HOXD9; WDR1; CAMK2D; STRA6; RAMP2; HOMER1; SMARCD3; HMGCR; CACNA2D2; FGFR2; KIAA1161; SMO; AKAP6; BMP2; SFMBT1; ARID1A; GSK3A; KIT; CDON; UTRN; CAPZB; FOXP1; PLCB1; GTF3C5; LUC7L; DAXX; CHD2; TGFB1; SIRT2; TCF3; MRAS; DNER; SUPT6H; SDC1; MEF2C; DTYMK; FLOT1; FLOT2; HES1; FLNB; EFNB2; PDGFRB; HDAC3; HDAC5; EHD2; EMD; HDAC9; CTDP1; TBX2; OLFM2; AKAP13; MAPK12; ABL1; MAP3K5; TSC1; FZD2; PLEKHO1; BTG1; MYH9; FHL2; FHL3; SRF; LAMA5; CTF1; SMTN; FLT3LG; FHOD3; ID3; BASP1; CDK5; CDK9; CDC42; STIM1; RBM4; IGSF8; USP2; CTNNB1; AEBP1; BCL9L; BIN1; BIN3; ITGB1BP2; AXIN1; KCNK2; ATG5; NRG1; MSX1; CNTF; HEG1; NR4A1; LMNA; USP19; CTNNA2; TAGLN; CALR; UNC45A; EPC1; BCL9; ATF3; EEF2 |
| GO:0009888 | 3 | tissue development | 0,01177 | 0,05285 | ACADVL; SEMA6A; PHGDH; TIMP1; TRIM16; SHARPIN; PPP2R3A; AGER; SIDT2; IKBKB; AKT1; PTK2B; RASIP1; SEMA4B; VASN; LEMD2; SEMA4F; GSN; SLC39A13; DLG3; SETD2; RARG; TFIP11; GATA2; CLOCK; DEAF1; NTRK1; TAZ; NTRK3; CAPN5; POFUT2; CD276; RELA; CUL7; HMG20B; CTSH; PPHLN1; CRYGS; TRIM28; HOXD9; SCUBE1; CAMK2D; ARHGEF19; MUC1; CRB2; CCDC78; SMAD1; HOMER1; BAMBI; LDB1; STK4; HMGCR; SNAI1; FGFR2; ROR2; BMP2; PAFAH1B1; MYL6; AKAP6; GPR161; TGFB1; BMP1; FN1; GLG1; CCND1; HYAL2; HYAL1; ROBO2; GSK3A; RFX3; PPP2CA; CDON; GRB2; BMPR2; MAGED1; KIAA1161; PAK1; EMD; MESDC2; FAM20C; NSDHL; ACVR1B; ALDH3A2; ITGA3; FMN1; CARM1; KIF26B; ID3; POFUT1; TMEM119; TMEM107; GTF3C5; ITGB5; LUC7L; ARHGAP12; HEG1; PLXNB2; AP2S1; GAP43; BNC2; TULP3; TIMELESS; THBS3; DAB2IP; MKS1; TMEM79; CHRD; NF2; MPP5; TGFB1I1; IFT122; MAFG; AGPAT2; DCHS1; SIRT2; NBR1; BIN3; ENG; DNASE1L2; STRA6; IFT140; CCM2; COL4A2; ARID1A; NELL1; KIF3A; ABCA12; HYAL3; IRF6; TOLLIP; DDR2; SDC1; DDR1; PSAP; PSMD13; MEF2C; PFN1; FLOT1; FLOT2; FLNA; FLNB; GAS6; EFNB2; PDGFRB; EFNB1; SUFU; VAX2; PSMB10; HDAC9; NODAL; SMAD6; ATM; H2AFY2; EPHA2; RET; CTDP1; SMO; SPRY1; FOXP1; AKAP13; PHLDB1; ABL1; THRA; TSC2; AKR1C1; TSC1; AP2M1; FZD2; ZBTB17; BTG1; SEMA4G; TEC; CHD7; TRADD; FHL2; ANKH; DAG1; MAPK1; PTCH2; FOSL2; PML; DUSP4; CITED1; BSG; FBXW4; USP2; APAF1; ATOH8; PHACTR4; GSTK1; CD63; LAMA5; PYGO2; PAF1; ASCL1; CELSR3; COL5A2; ENO3; TUFT1; LGALS3; TRIM62; RAB13; SLC40A1; CEP152; FHOD3; ZNF689; LRP4; AHI1; PLXNA2; PKD1; GSTM3; MEGF8; BAD; RGS19; STAT5B; BASP1; PLXNA1; PRKACA; CDK5; FZD4; DUSP2; PDE4D; IFT20; PRKD2; CDC42; AKR1C3; STIM1; ABI1; IGSF8; SCRIB; NUMA1; PHB2; PSMD11; ERCC2; TBX2; NPHP3; CTNNB1; MAP2K2; BMPR1B; DLC1; SSBP3; HAND2; BCL9L; AACS; SRF; SEMA6D; PRKX; NDRG4; SEMA6C; P2RX7; ALDH1A3; PGF; CEBPB; DNER; RGS20; CRABP2; PLOD3; SLC9A3R1; CBR1; KCNK2; MLLT3; DVL3; ATP6AP2; ALDH1A2; SOX7; ATG5; NRG1; MSX1; SIPA1L3; MAD2L2; PSMC4; PSMC5; GAA; KEAP1; MTHFD1L; ESRRA; CLASP2; CLASP1; CRELD1; COL18A1; NR4A3; NR4A1; RDH10; SRC; NLE1; PTPRO; GPC3; RTF1; AP2A2; ALDOC; USP19; BCL10; NRP2; TAGLN; CASP6; NFIB; E2F4; AXIN1; WDR77; PPP1CA; NOTCH4; IFT172; CALR; AARS; TLX2; PDE2A; LMNA; DLL3; BCL9; ATF3; MBD3; CTNNBIP1; COL6A1; SERPINH1; HES1; LOXL3; EEF2; PPP1R13L |
| GO:0060348 | 3 | bone development | 0,012202 | 0,053932 | MCPH1; TGFB1; MMP16; FAM20C; VKORC1; SMAD1; GLG1; MEGF8; FOXP1; AKAP13; ANKRD11; PLXNB1; INPPL1; WASF2; ASXL1; FGFR2; ATP6AP1; THBS3; MSX1; DCHS1; SRC; SRF; RARG; CARM1; BBX; PAFAH1B1; GPR68; BMP2; PEX7; TMEM119; IFT172; ABI1; BMPR1B; KIT; MEF2C; FREM1; BMPR2; TULP3; SERPINH1; PPIB |
| GO:0048598 | 3 | embryonic morphogenesis | 0,01225 | 0,053932 | ARFRP1; MFAP2; SETD2; RARG; TMEM107; DEAF1; FGFR2; POFUT2; TRIM28; HOXD9; STRA6; SMAD1; LDB1; STK4; MSX1; SNAI1; GATA2; ROR2; SMO; PCGF2; FN1; ARID1A; CDON; GRB2; BMPR2; HYAL1; MMP16; CDKN1C; ITGA3; NDRG4; PLXNB2; CHD7; TULP3; DLD; MKS1; NF2; TGFB1I1; IFT122; EPHB2; RIC8A; ENG; COL4A2; PHLDB1; SLC39A3; KIF3A; MTHFD1L; NPHP3; MEF2C; PFN1; HES1; DAG1; VAX2; ACD; NODAL; EPHA2; RET; TBX2; SLC39A1; TSC2; TSC1; FZD2; ZBTB17; MAPK1; MAPK7; DUSP4; DLC1; APAF1; ATOH8; PHACTR4; SRF; LAMA5; UGDH; NAGLU; COL5A2; AHI1; LRP4; FBXW4; CHRD; PRKACA; DUSP2; MAP2K5; TMIE; CTNNB1; MEGF8; HAND2; SOX7; ALDH1A2; ALDH1A3; CRABP2; ALX3; SLC9A3R1; DVL3; ATP6V1B1; PAF1; ITGB5; CLASP2; CLASP1; NR4A3; RDH10; ABR; GPC3; RTF1; SUFU; CRB2; BCL10; AXIN1; IFT172; TLX2; SCRIB; GNA12; COL6A1; TGIF2 |
| GO:0030323 | 3 | respiratory tube development | 0,015706 | 0,066143 | FOXP1; NUMA1; NODAL; HMGB1; ITGA3; AGER; DHCR7; CTNNB1; MAP2K2; SPRY1; ALDH1A2; STK40; CEBPA; ADAMTS2; ASXL1; TULP3; TIMELESS; THRA; MAPK1; EIF4EBP1; HEG1; CTSH; SRF; LAMA5; STRA6; ASCL1; RDH10; MAN2A1; GPC3; DAG1; FGFR2; NFIB; ABCA12; PPP1CA; PKD1; NPHP3; SREBF1; BMPR2; PLOD3; HES1; CDC42 |
| GO:0030324 | 3 | lung development | 0,017567 | 0,071751 | FOXP1; NUMA1; NODAL; HMGB1; ITGA3; AGER; DHCR7; CTNNB1; MAP2K2; SPRY1; ALDH1A2; STK40; CEBPA; ADAMTS2; ASXL1; PLOD3; TIMELESS; THRA; MAPK1; EIF4EBP1; HEG1; CTSH; SRF; LAMA5; STRA6; ASCL1; RDH10; MAN2A1; GPC3; DAG1; FGFR2; NFIB; ABCA12; PPP1CA; PKD1; NPHP3; SREBF1; BMPR2; HES1; CDC42 |
| GO:0048729 | 3 | tissue morphogenesis | 0,021697 | 0,084542 | SUFU; GSN; DLG3; SETD2; RARG; FGFR2; DEAF1; MPP5; POFUT2; CTSH; TRIM28; ARHGEF19; SMAD1; STK4; SNAI1; PAFAH1B1; ROR2; SMO; BMP2; MAGED1; ARID1A; GRB2; BMPR2; PKD1; FOXP1; ITGA3; FMN1; KIF26B; NDRG4; HEG1; PLXNB2; AP2S1; TULP3; TIMELESS; MKS1; TMEM79; NF2; TGFB1I1; IFT122; DCHS1; ENG; CCM2; DAG1; KIF3A; DDR1; PSMD11; PSMD13; PAK1; MEF2C; PFN1; FLNA; EFNB2; RASIP1; PSMB10; NODAL; CRYGS; EPHA2; RET; TBX2; SPRY1; ABL1; TSC2; TSC1; FZD2; FZD4; AP2M1; PML; CITED1; DLC1; APAF1; PHACTR4; SRF; LAMA5; CELSR3; AHI1; PLXNA1; CHRD; PRKACA; PRKD2; CDC42; TGFB1; MTHFD1L; SRC; PHB2; NPHP3; CTNNB1; MEGF8; HAND2; PRKX; ALDH1A2; ALDH1A3; PGF; AXIN1; PLOD3; SLC9A3R1; MLLT3; DVL3; NRG1; MSX1; PSMC4; PSMC5; CLASP2; CLASP1; NR4A3; GPC3; AP2A2; CRB2; BCL10; NRP2; ARHGAP12; PPP1CA; NOTCH4; IFT172; TLX2; SCRIB; RDH10; CTNNBIP1; HES1 |
| GO:0048568 | 3 | embryonic organ development | 0,022338 | 0,086108 | MMP16; GAMT; RAD23B; SCRIB; SUFU; CDKN1C; ERCC1; PLCD1; EPHA2; TBX2; TMIE; SMO; AKT1; MEGF8; VAX2; MFAP2; HAND2; TRIM28; NDRG4; ALDH1A2; ALDH1A3; STK4; CEBPB; CEBPA; IFT122; NSDHL; RARG; ALX3; FGFR2; SLC9A3R1; CHD7; THRA; MKS1; DVL3; NR4A3; MAPK1; SLC39A3; NODAL; ATP6V1B1; CITED1; EPHB2; PHACTR4; SRF; HOXD9; ENG; POLE; NAGLU; CRB2; EPN1; FZD2; AHI1; ABR; FLT3LG; PLCD3; MSX1; ARID1A; SLC39A1; SNAI1; GATA2; ROR2; THOC5; KIF3A; CTNNB1; SETD2; PCGF2; AXIN1; ARNT; IFT172; HYAL1; MTHFD1L; ID3; NPHP3; KIT; MEF2C; GRB2; RDH10; MBD3; TULP3; STRA6; HES1; PPP1R13L; PKD1 |
| GO:0016358 | 3 | dendrite development | 0,02603 | 0,095397 | IL1RAPL1; FMN1; CARM1; SRGAP2; DBN1; PREX1; CAMK1; ARF6; NTRK2; DISC1; PRKG1; MAPK8IP2; DCX; NRG1; CUL7; LZTS1; EPHB2; MAP1B; FBXO31; TMEM106B; NLGN1; BCL11A; MECP2; DAB2IP; SEZ6; PALM; CFL1; OPA1; GRN; ANAPC2; PAFAH1B1; SARM1; CTNNA2; RAB21; PPP1R9B; LRP4; LRP8; PQBP1; TLX2; MEF2C; CDK5 |
| GO:0021543 | 3 | pallium development | 0,028225 | 0,101252 | MCPH1; SRGAP2; SMO; BAD; TSC1; MDK; NTRK2; DISC1; NF2; DCX; CDK5R2; WDR62; PPP1R9B; FBXO45; PLCB1; SRF; NR4A3; ASCL1; DAB2IP; NDEL1; EFHC1; PAFAH1B1; NRP2; KIF3A; CTNNB1; PLXNA3; TACC1; LRP8; TACC3; H2AFX; XAB2; CDON; CDK5; DIXDC1; TACC2 |
| GO:0021537 | 3 | telencephalon development | 0,029643 | 0,10578 | MCPH1; FBXO45; TACC3; DRD2; SRGAP2; SMO; BAD; ALDH1A3; MDK; CHD7; NTRK2; DISC1; CTNNB1; SLIT3; NF2; DCX; CDK5R2; NRG1; TSC1; SECISBP2; EPHB2; PLCB1; SRF; NRGN; NR4A3; ASCL1; DAB2IP; WDR62; NDEL1; EFHC1; PAFAH1B1; NRP2; KIF3A; NFIB; PPP1R9B; BMP2; PLXNA3; TACC1; LRP8; ROBO2; H2AFX; XAB2; CDON; CDK5; DIXDC1; ATF5; HES1; TACC2; SLC1A2 |
| GO:0007423 | 3 | sensory organ development | 0,031178 | 0,110097 | PPP2R3A; ANP32B; SCO2; VAX2; RARG; FGFR2; SIX6; NTRK2; NTRK3; THY1; CLN8; CLCN2; EPHA2; SMARCD3; MSX1; GATA2; ROR2; PAFAH1B1; BMP2; MAX; ARID1A; CDON; BMPR2; MFAP2; EPHB2; CDKN1C; MAPKAPK2; CHD7; TULP3; MKS1; NF2; ZNF513; IFT122; DCHS1; RAB3GAP1; STRA6; IFT140; MAN2A1; CCM2; FZD4; KIF3A; DDR1; PDE6B; HES1; PDGFRB; FJX1; CRYGS; AHI1; RET; TBX2; NPHP4; FZD2; STAT3; RAB18; MAPK1; PHACTR4; SRF; PYGO2; NAGLU; ASCL1; GNB1; COL5A2; SERPINF1; RAB11FIP4; MFN2; TGFB1; HMGB1; TMIE; CTNNB1; BMPR1B; HAND2; GPD2; SMARCA4; ALDH1A2; ALDH1A3; CEBPA; AXIN1; SLC9A3R2; SLC9A3R1; KCNK2; KCNK3; DVL3; ATG5; ATP6V1B1; SIPA1L3; LRP10; CNTF; NR4A3; ABR; CRB2; CTNS; BNC2; SCRIB; RDH10; RDH13; PTPRM; PPP1R13L; TGIF2 |
| GO:1904888 | 3 | cranial skeletal system development | 0,033212 | 0,115475 | MEF2C; MMP16; SLC39A3; TGFB1; ALX3; NODAL; MTHFD1L; FREM1; CTNNB1; MEGF8; RDH10; TULP3; SLC39A1; SETD2; FGFR2 |
| GO:0007507 | 3 | heart development | 0,034806 | 0,118233 | IFT20; LEMD2; SETD2; GATA2; TAZ; NTRK3; SOD2; CAMK2D; STRA6; RAMP2; BAZ1B; STK4; SNAI1; FGFR2; SMO; AKAP6; BMP2; ARID1A; GSK3A; FOXP1; CRELD1; NDRG4; CHD7; ASXL1; NEK8; IFT122; POFUT1; DCHS1; ENG; FZD2; CCM2; GYS1; HEXIM1; VEGFB; KIF3A; RPS6KA2; SCUBE1; NPHP3; MEF2C; HES1; EFNB2; PDGFRB; HDAC9; NODAL; SMAD6; ATM; SMAD1; CTDP1; TBX2; PLCE1; AKAP13; TSC2; TSC1; SNX17; IFT140; FHL2; MAPK1; DLC1; ITGA3; SRF; MECP2; NPY2R; MOSPD3; FHOD3; AHI1; GALNT11; PKD1; ID3; BASP1; CDC42; TGFB1; MAP2K5; CTNNB1; MAP2K2; MEGF8; HAND2; ALDH1A2; CAD; KCNK2; DVL3; ATG5; NRG1; MSX1; GAA; HEG1; GPC3; SUFU; LMNA; NRP2; IFT172; CALR; MBD1; MBD3; GNA11; SMARCD3; PPP1R13L |
| GO:0001822 | 3 | kidney development | 0,035144 | 0,118233 | EFNB2; PDGFRB; IFT20; HYAL2; CDKN1C; SMAD6; ITGA3; FMN1; SMAD1; RET; SMO; SPRY1; PLCE1; PRKX; ALDH1A2; TSC1; PGF; APH1A; THRA; MPST; CITED1; DCHS1; CTSH; FLCN; LZTS2; LAMA5; PYGO2; STRA6; AHI1; NLE1; PTPRO; GPC3; SERPINF1; KIF26B; FGFR2; KIF3A; CTNNB1; TGFB1; BMP2; LRP4; SDC1; PKD1; ROBO2; ID3; NPHP3; NOTCH3; MEF2C; BASP1; RDH10; CTNNBIP1; MME; HES1; WFS1; MAGED1 |
| GO:0010463 | 3 | mesenchymal cell proliferation | 0,035226 | 0,118233 | SMO; FOXP1; BMP2; NFIB; FBXW4; CHRD; CTNNB1; GPC3; HAND2; CTNNBIP1; MSX1; FGFR2 |
| GO:0048167 | 3 | regulation of synaptic plasticity | 0,037326 | 0,12345 | HRAS; RAB3GAP1; STXBP1; PTK2B; DBN1; NLGN1; NTRK2; MAPK1; SLC8A2; MECP2; LZTS1; EPHB2; SRF; NRGN; VGF; SNCA; CPLX2; PRKCZ; UNC13B; UNC13A; ITPR3; ANAPC2; RGS14; VAMP2; SYNGR1; DRD2; KIT; MEF2C; CDK5; NCDN; DGKI |
| GO:0048857 | 3 | neural nucleus development | 0,038872 | 0,127319 | NFIB; SCRIB; CALM3; CNP; SIRT2; ZNF148; SYNGR3; ASCL1; MAPKAP1; MAOB; BASP1; ENO3; ZNF430; RAD1; CDK5R2; ALDH1A3; CDC42 |
| GO:0060041 | 3 | retina development in camera-type eye | 0,041847 | 0,135105 | PDGFRB; FJX1; AHI1; RET; BMPR1B; NPHP4; SMARCA4; VAX2; IFT140; FZD4; RARG; NTRK2; ZNF513; THY1; CLN8; CLCN2; GNB1; MAN2A1; CRB2; SMARCD3; CHD7; RAB11FIP4; MAX; PDE6B; CDON; BMPR2; RDH13; SERPINF1; PTPRM; TGIF2 |
| GO:0001892 | 3 | embryonic placenta development | 0,047956 | 0,151229 | CEBPB; CEBPA; SETD2; TRIM28; NSDHL; ARNT; CDKN1C; GATA2; PKD1; PLCD1; GRB2; AKT1; MAPK1; STK4; PLCD3; NODAL; HES1; CITED1; SNAI1; FGFR2 |

**Supplementary Table S3.** Transcription factor binding site analysis for promoters of genes identified in microarray analysis. Promoters that were available in promoter databases and that contained at least one motif with a dissimilarity lower than 15 % are listed; putative binding motifs with dissimilarity score lower than 5 % are indicated in boldface.

| **Gene** | **Entrez Gene Name** | **Promoter ID (https://cb.utdallas.edu/cgi-bin/TRED/tred.cgi?process=searchPromForm)** | **Start position** | **End position** | **String** | **Dissimilarity**  **Score**  **(%)** |
| --- | --- | --- | --- | --- | --- | --- |
| ACVR2B | activin A receptor, type IIB | 29253 | **-90** | **-82** | **CGCAGGAAG** | **0,13** |
|  |  |  | **-64** | **-56** | **CGAAGGAAG** | **0,96** |
|  |  |  | -54 | -46 | AGCGGGAAG | 8,93 |
| ADRBK1 | adrenergic, beta, receptor kinase 1 | 6794 | **-7** | **1** | **CTTCCTTGA** | **3,12** |
|  |  |  | **59** | **67** | **CTTCCTGGA** | **2,3** |
|  |  |  | 59 | 67 | CTTCCCCTT | 11,1 |
|  |  |  | 65 | 73 | CTTCCCCGG | 11,1 |
|  |  |  | 188 | 196 | CTTCCCACC | 10,44 |
| ARNT | aryl hydrocarbon receptor nuclear translocator | 3024 | **-184** | **-176** | **CTTCCTCGC** | **2,16** |
| AXIN2 | axin 2 | 18200 | **-666** | **-658** | **GTTAGGAAG** | **4,89** |
|  |  |  | -137 | -129 | CCGGGGAAG | 11,1 |
|  |  |  | 147 | 185 | GGGTGGAAG | 8,8 |
| BCAR1 | breast cancer anti-estrogen resistance 1 | 15827 | **-555** | **-547** | **CCCAGGAAG** | **2,3** |
|  |  |  | **-519** | **-511** | **CTTCCTTTG** | **3,12** |
|  |  |  | **-334** | **-326** | **GAAAGGAAG** | **2,99** |
|  |  |  | -303 | -295 | CTTCCAGCC | 8,8 |
|  |  |  | -155 | -147 | CTTCCCTGG | 11,92 |
|  |  |  | -71 | -63 | CTTCCGCCG | 6,73 |
| BID | BH3 interacting domain death agonist | 29041 | -620 | -612 | GGTGGGAAG | 10,44 |
|  |  |  | **-39** | **-31** | **AGGAGGAAG** | **0,13** |
|  |  |  | **-14** | **-6** | **AGGAGGAAG** | **0,13** |
| BMP6 | bone morphogenetic protein 6 | 34934 | -447 | -439 | CTTCCAGCA | 8,93 |
|  |  |  | **-382** | **-374** | **CTAAGGAAG** | **4,2** |
|  |  |  | **-346** | **-338** | **CTTCCTCAG** | **3,38** |
|  |  |  | **125** | **133** | **CTTCCTTCC** | **0,82** |
|  |  |  | 129 | 137 | CTTCCCATC | 12,61 |
|  |  |  | **289** | **297** | **AGAAGGAAG** | **0,96** |
| BMPR1B | bone morphogenetic protein receptor, type IB | 31608 | -320 | -312 | CTTGGGAAG | 13,82 |
| BTRC | beta-transducin repeat containing | 4883 | -484 | -476 | CTTCCTAAA | 5,03 |
|  |  |  | -164 | -156 | TGGGGGAAG | 8,93 |
|  |  |  | -53 | -45 | GGGGGGAAG | 8,8 |
|  |  |  | **-49** | **-41** | **GGAAGGAAG** | **0,82** |
|  |  |  | 76 | 84 | CTTCCGGAG | 9,98 |
|  |  |  | 277 | 285 | CTGGGGAAG | 12,18 |
| CCND1 | cyclin D1 | 6842 | **-327** | **-319** | **CTTCCTAGT** | **3,94** |
|  |  |  | -234 | -226 | CTTCCCTGG | 11,92 |
|  |  |  | **190** | **198** | **CCCAGGAAG** | **2,3** |
| CDYL | chromodomain protein, Y-like | 34916 | 52 | 60 | TCCCGGAAG | 8,9 |
|  |  |  | **143** | **151** | **GAGAGGAAG** | **2,16** |
| CHRM3 | cholinergic receptor, muscarinic 3 | 2062 | -695 | -687 | CTTCCAGTC | 10,96 |
|  |  |  | **-315** | **-307** | **AGGAGGAAG** | **0,13** |
|  |  |  | **-262** | **-254** | **CAGAGGAAG** | **2,3** |
|  |  |  | 119 | 127 | TCTGGGAAG | 12,74 |
|  |  |  | 213 | 221 | GAAGGGAAG | 11,78 |
| CNGA3 | cyclic nucleotide gated channel alpha 3 | 23570 | **-14** | **-6** | **GTCAGGAAG** | **3,25** |
| CNR1 | cannabinoid receptor 1 (brain) | 36466 | 30 | 38 | CTTCCCCTT | 11,1 |
|  |  |  | 36 | 44 | CTTCCGGCT | 6,73 |
|  |  |  | 70 | 78 | CCGCGGAAG | 8,9 |
|  |  |  | **242** | **250** | **AGGAGGAAG** | **0,13** |
| CREB3 | cAMP responsive element binding protein 3 | 41455 | -498 | -490 | CTTCCGTCC | 7,42 |
|  |  |  | **-428** | **-420** | **GGCAGGAAG** | **0** |
|  |  |  | **-362** | **-354** | **GGCAGGAAG** | **0** |
|  |  |  | **-287** | **-279** | **CTTCCTTTT** | **3,12** |
|  |  |  | **136** | **144** | **CTTCCTGCT** | **0,13** |
| CREM | cAMP responsive element modulator | 4403 | **-530** | **-522** | **GTTAGGAAG** | **4,89** |
|  |  |  | -426 | -418 | CTTCCGGTT | 8,9 |
|  |  |  | -394 | -386 | CTTCCGCCC | 6,6 |
|  |  |  | **-344** | **-336** | **CTTCCTTCT** | **0,96** |
|  |  |  | -302 | -294 | CTTCCCGCC | 8,8 |
| CXCR2 | C-X-C motif chemokine receptor 2 | 24157 | -543 | -535 | CTTCCAGAT | 12,18 |
|  |  |  | **-389** | **-381** | **AGTAGGAAG** | **1,78** |
|  |  |  | **-385** | **-377** | **GGAAGGAAG** | **0,82** |
|  |  |  | **-340** | **-332** | **GTGAGGAAG** | **3,25** |
|  |  |  | **-124** | **-116** | **AGAAGGAAG** | **0,96** |
|  |  |  | **-62** | **-54** | **CTTCCTTGT** | **3,12** |
|  |  |  | **-50** | **-42** | **AAAAGGAAG** | **3,12** |
|  |  |  | **41** | **49** | **GACAGGAAG** | **2,16** |
| DLL1 | delta-like 1 (Drosophila) | 36027 | **-479** | **-471** | **GGGAGGAAG** | **0** |
|  |  |  | -433 | -425 | GCGGGGAAG | 10,96 |
|  |  |  | **119** | **127** | **GGGAGGAAG** | **0** |
|  |  |  | **221** | **229** | **ACCAGGAAG** | **2,3** |
|  |  |  | **257** | **265** | **GGGAGGAAG** | **0** |
| DRD2 | dopamine receptor D2 | 7494 | **-683** | **-675** | **CTTCCTCTC** | **2,16** |
|  |  |  | -85 | -77 | TCCTGGAAG | 11,1 |
| DRD3 | dopamine receptor D3 | 30601 | -698 | -690 | CATGGGAAG | 12,74 |
|  |  |  | **-644** | **-636** | **GTTAGGAAG** | **4,89** |
|  |  |  | -604 | -596 | CTTCCCATC | 12,61 |
|  |  |  | **-463** | **-455** | **CTTCCTGCT** | **0,13** |
|  |  |  | **-397** | **-389** | **CTTCCTGAT** | **3,38** |
|  |  |  | -185 | -177 | AAAGGGAAG | 11,92 |
|  |  |  | **-6** | **2** | **CACAGGAAG** | **2,3** |
| DTX1 | deltex homolog 1 (Drosophila) | 9363 | **-404** | **-396** | **CTTCCTCCT** | **0,13** |
|  |  |  | -55 | -47 | CTGGGGAAG | 12,18 |
|  |  |  | 71 | 79 | CTTCCCGGC | 10,96 |
|  |  |  | 123 | 131 | CGGGGGAAG | 8,93 |
|  |  |  | 128 | 136 | GAAGGGAAG | 11,78 |
|  |  |  | 204 | 212 | CTTCCCTCC | 9,62 |
| DTX2 | deltex homolog 2 (Drosophila) | 37884 | **-161** | **-153** | **CGAAGGAAG** | **0,96** |
|  |  |  | -114 | -106 | CTTCCCCGC | 10,96 |
|  |  |  | -20 | -12 | GCTCGGAAG | 10,41 |
|  |  |  | 134 | 142 | GGCGGGAAG | 8,8 |
|  |  |  | 213 | 221 | GACCGGAAG | 8,76 |
| DUSP1 | dual specificity phosphatase 1 | 33996 | **-467** | **-459** | **CACAGGAAG** | **2,3** |
|  |  |  | -426 | -418 | ACGGGGAAG | 11,1 |
|  |  |  | 245 | 253 | TCATGGAAG | 11,92 |
| DUSP2 | dual specificity phosphatase 2 | 25344 | **-399** | **-391** | **CAGAGGAAG** | **2,3** |
|  |  |  | -231 | -223 | CTTCCCTTC | 11,78 |
|  |  |  | -226 | -218 | CTTCCCTTA | 11,92 |
|  |  |  | -170 | -162 | CTTCCCCCT | 8,93 |
|  |  |  | **-77** | **-69** | **CTTCCTGCT** | **0,13** |
|  |  |  | **57** | **65** | **GAAAGGAAG** | **2,99** |
|  |  |  | **63** | **71** | **AAGAGGAAG** | **2,3** |
|  |  |  | **187** | **195** | **CTTCCTGGC** | **2,16** |
| DUSP4 | dual specificity phosphatase 4 | 41090 | -570 | -562 | GCGGGGAAG | 10,96 |
|  |  |  | **-327** | **-319** | **CTTCCTCTC** | **2,16** |
|  |  |  | **221** | **229** | **GGGAGGAAG** | **0** |
|  |  |  | 233 | 241 | ACAGGGAAG | 11,92 |
| DVL2 | dishevelled, dsh homolog 2 (Drosophila) | 19128 | -679 | -671 | CTTCCCCTA | 11,1 |
|  |  |  | **-627** | **-619** | **GTGAGGAAG** | **3,25** |
|  |  |  | **17** | **25** | **TGGAGGAAG** | **0,13** |
| EGLN1 | egl nine homolog 1 (C. elegans) | 2205 | **-485** | **-477** | **CTTCCTGTT** | **2,3** |
|  |  |  | -292 | -284 | CCTTGGAAG | 12,74 |
|  |  |  | -115 | -107 | AAAGGGAAG | 11,92 |
| ELF2 | E74-like factor 2 | 32204 | -379 | -371 | CTTCCAGGA | 11,1 |
|  |  |  | **-377** | **-369** | **TCCAGGAAG** | **2,3** |
|  |  |  | -318 | -310 | CTTCCGCAG | 9,98 |
|  |  |  | **-293** | **-285** | **CTTCCTGCC** | **0** |
|  |  |  | **-187** | **-179** | **CTTCCTGTT** | **2,3** |
|  |  |  | -170 | -162 | GGGCGGAAG | 6,6 |
|  |  |  | **-22** | **-14** | **CTTCCTGTC** | **2,16** |
| ELF5 | E74-like factor 5 | 8146 | **-249** | **-241** | **TTAAGGAAG** | **4,2** |
|  |  |  | -227 | -219 | CTTCCCAAT | 13,82 |
|  |  |  | **-197** | **-189** | **TTGAGGAAG** | **3,38** |
|  |  |  | **-38** | **-30** | **GTCAGGAAG** | **3,25** |
|  |  |  | 17 | 25 | CTTCCCCTC | 10,96 |
|  |  |  | 225 | 233 | CTTCCATGC | 11,78 |
| EPO | erythropoietin | 38027 | **-528** | **-520** | **GGGAGGAAG** | **0** |
|  |  |  | -439 | -431 | TGTGGGAAG | 10,58 |
|  |  |  | -393 | -385 | CTTCCAGAC | 12,04 |
|  |  |  | 144 | 152 | CTTCCCGGG | 11,1 |
| ETS1 | v-ets erythroblastosis virus E26 oncogene homolog 1 (avian) | 7353 | -303 | -295 | CTTCCCGCC | 8,8 |
|  |  |  | **-218** | **-210** | **CTTCCTTTC** | **2,99** |
|  |  |  | 125 | 133 | CTTCCCCTG | 11,1 |
|  |  |  | 194 | 202 | TTTGGGAAG | 13,82 |
|  |  |  | 225 | 233 | CTTCCCCCT | 8,93 |
| ETS2 | v-ets erythroblastosis virus E26 oncogene homolog 2 (avian) | 27401 | -616 | -608 | CTTCCCGGG | 11,1 |
|  |  |  | **-557** | **-549** | **TACAGGAAG** | **2,3** |
|  |  |  | -221 | -213 | GGAGGGAAG | 9,62 |
|  |  |  | -208 | -200 | GGCCGGAAG | 6,6 |
|  |  |  | -94 | -86 | CTTCCCTCT | 9,75 |
|  |  |  | -54 | -46 | CTTCCCCTC | 10,96 |
|  |  |  | -28 | -20 | CTTCCCTCC | 9,62 |
|  |  |  | **37** | **45** | **CTTCCTCCT** | **0,13** |
|  |  |  | **105** | **113** | **CTTCCTCCA** | **0,13** |
|  |  |  | -245 | -237 | CTTCCAAGA | 12,74 |
|  |  |  | -88 | -80 | CTTCCCAGG | 12,74 |
| FGF8 | fibroblast growth factor 8 (androgen-induced) | 5410 | **-570** | **-562** | **CCGAGGAAG** | **2,3** |
|  |  |  | -526 | -518 | CAGGGGAAG | 11,1 |
|  |  |  | **-341** | **-333** | **TCTAGGAAG** | **3,94** |
|  |  |  | -296 | -288 | CTTCCCTGT | 11,92 |
|  |  |  | 79 | 87 | AGCGGGAAG | 8,93 |
|  |  |  | 242 | 250 | GAAGGGAAG | 11,78 |
|  |  |  | 283 | 291 | CTGGGGAAG | 12,18 |
| FGF9 | fibroblast growth factor 9 | 10702 | -686 | -678 | CAGGGGAAG | 11,1 |
|  |  |  | -499 | -491 | CTTCCCCAC | 12,04 |
|  |  |  | -345 | -337 | GGAGGGAAG | 9,62 |
|  |  |  | -49 | -41 | CTTCCACGT | 11,1 |
|  |  |  | **-23** | **-15** | **AGCAGGAAG** | **0,13** |
|  |  |  | 91 | 99 | CTTCCCCGA | 11,1 |
| FGF11 | fibroblast growth factor 11 | 16802 | -655 | -647 | TTCTGGAAG | 12,18 |
|  |  |  | -536 | -528 | CTTCCAGAA | 12,18 |
|  |  |  | -326 | -318 | CTTCCCCCA | 8,93 |
|  |  |  | **-216** | **-208** | **CTTCCTACC** | **1,65** |
|  |  |  | **-187** | **-179** | **CTTCCTGTC** | **2,16** |
|  |  |  | 32 | 40 | CTTCCCACA | 10,58 |
|  |  |  | 135 | 143 | CTTCCCGAG | 12,18 |
|  |  |  | 168 | 176 | GGGGGGAAG | 8,8 |
|  |  |  | **274** | **282** | **AAAAGGAAG** | **3,12** |
| FGF21 | fibroblast growth factor 21 | 21072 | -681 | -673 | GAGCGGAAG | 8,76 |
|  |  |  | -615 | -607 | CTTAGGAAG | 5,03 |
|  |  |  | **-343** | **-335** | **GAGAGGAAG** | **2,16** |
|  |  |  | -281 | -273 | GGCTGGAAG | 8,8 |
|  |  |  | -16 | -8 | CTTCCGGTG | 8,9 |
| FGFR1 | fibroblast growth factor receptor 1 | 41052 | **-641** | **-633** | **CTTCCTAGA** | **3,94** |
|  |  |  | -611 | -603 | TTCTGGAAG | 12,18 |
|  |  |  | -517 | -509 | CAAGGGAAG | 11,92 |
|  |  |  | **-178** | **-170** | **TGTAGGAAG** | **1,78** |
|  |  |  | 167 | 175 | CTTCCCCGG | 11,1 |
| FGFR3 | fibroblast growth factor receptor 3 | 31175 | -247 | -239 | CTTCCCGCT | 8,93 |
|  |  |  | -188 | -180 | GGAGGGAAG | 9,62 |
|  |  |  | **-14** | **-6** | **CTTCCTCCT** | **0,13** |
| FOXC1 | forkhead box C1 | 34872 | **-579** | **-571** | **GTGAGGAAG** | **3,25** |
|  |  |  | -439 | -431 | CTTCCCCCA | 8,93 |
|  |  |  | **-297** | **-289** | **CTTCCTGTC** | **2,16** |
|  |  |  | -231 | -223 | CTTCCGTGC | 9,59 |
|  |  |  | -108 | -100 | CTGGGGAAG | 12,18 |
|  |  |  | 288 | 296 | CTTCCCCAC | 12,04 |
| FRAT1 | frequently rearranged in advanced T-cell lymphomas | 4795 | **-462** | **-454** | **CTTCCTTTC** | **2,99** |
|  |  |  | -264 | -256 | GTGTGGAAG | 12,04 |
|  |  |  | 55 | 63 | CTTCCGCGT | 8,9 |
|  |  |  | **205** | **213** | **AGGAGGAAG** | **0,13** |
|  |  |  | **211** | **219** | **AAGAGGAAG** | **2,3** |
|  |  |  | **238** | **246** | **GGGAGGAAG** | **0** |
|  |  |  | **260** | **268** | **CTTCCTCCT** | **0,13** |
| FRZB | frizzled-related protein | 24847 | -697 | -689 | CTCGGGAAG | 12,18 |
|  |  |  | -452 | -444 | CTTCCCCCC | 8,8 |
|  |  |  | **-312** | **-304** | **CTTCCTCGC** | **2,16** |
|  |  |  | -221 | -213 | CTTCCGAAG | 11,63 |
|  |  |  | **-123** | **-115** | **CCAAGGAAG** | **3,12** |
|  |  |  | **271** | **279** | **CTTCCTCTG** | **2,3** |
| FURIN | furin (paired basic amino acid cleaving enzyme) | 13678 | -501 | -493 | AGCTGGAAG | 8,93 |
|  |  |  | **-475** | **-467** | **CTGAGGAAG** | **3,38** |
|  |  |  | **-411** | **-403** | **CTTCCTATG** | **3,94** |
|  |  |  | -273 | -265 | CCATGGAAG | 11,92 |
|  |  |  | -237 | -229 | TGGCGGAAG | 6,73 |
|  |  |  | **-233** | **-225** | **GGAAGGAAG** | **0,82** |
|  |  |  | 2 | 10 | AACTGGAAG | 11,1 |
|  |  |  | 63 | 71 | GCGGGGAAG | 10,96 |
| FZD2 | frizzled homolog 2 (Drosophila) | 17320 | -559 | -551 | CCCGGGAAG | 11,1 |
|  |  |  | **-121** | **-113** | **AGGAGGAAG** | **0,13** |
|  |  |  | 52 | 60 | GCGGGGAAG | 10,96 |
| FZD4 | frizzled homolog 4 (Drosophila) | 7606 | **-361** | **-353** | **GACAGGAAG** | **2,16** |
| FZD5 | frizzled homolog 5 (Drosophila) | 24718 | **-597** | **-589** | **GCCAGGAAG** | **2,16** |
|  |  |  | -524 | -516 | CTTCCCGCC | 8,8 |
|  |  |  | -300 | -292 | CTTCCCGGC | 10,96 |
|  |  |  | **40** | **48** | **CTTCCTCCC** | **0** |
| FZD10 | frizzled homolog 10 (Drosophila) | 9511 | -599 | -591 | CTTTGGAAG | 13,82 |
|  |  |  | -508 | -500 | TACGGGAAG | 11,1 |
|  |  |  | -436 | -428 | CGGGGGAAG | 8,93 |
|  |  |  | **-125** | **-117** | **CTTCCTATA** | **3,94** |
|  |  |  | -7 | 1 | CTTCCCGCG | 8,93 |
|  |  |  | 34 | 42 | CTTCCCGCC | 8,8 |
|  |  |  | 50 | 58 | CTTCCCGCC | 8,8 |
|  |  |  | **200** | **208** | **CTCAGGAAG** | **3,38** |
| GABBR1 | gamma-aminobutyric acid (GABA) B receptor, 1 | 37015 | **-589** | **-581** | **CTTCCTCTT** | **2,3** |
|  |  |  | -503 | -495 | CTTCCCTCC | 9,62 |
|  |  |  | **-487** | **-479** | **CTTCCTGCC** | **0** |
|  |  |  | **-307** | **-299** | **CGTAGGAAG** | **1,78** |
|  |  |  | -293 | -285 | CTTCCCTGC | 11,78 |
| GATA4 | GATA binding protein 4 | 39727 | **-519** | **-511** | **CTTCCTGAC** | **3,25** |
|  |  |  | -456 | -448 | CCCGGGAAG | 11,1 |
|  |  |  | -369 | -361 | CTTCCCGGC | 10,96 |
|  |  |  | -306 | -298 | GCGTGGAAG | 10,96 |
|  |  |  | **-201** | **-193** | **AGGAGGAAG** | **0,13** |
|  |  |  | -8 | 0 | CTTCCGGAA | 9,98 |
| GNA15 | guanine nucleotide binding protein (G protein), alpha 15 | 20145 | **-196** | **-188** | **AGGAGGAAG** | **0,13** |
|  |  |  | **38** | **46** | **CTTCCTGGG** | **2,3** |
|  |  |  | **55** | **63** | **GCAAGGAAG** | **2,99** |
| GRB2 | growth factor receptor-bound protein 2 | 18124 | **-634** | **-626** | **CTTCCTCAG** | **3,38** |
|  |  |  | **-569** | **-561** | **CTTCCTAGA** | **3,94** |
|  |  |  | -360 | -352 | TTCTGGAAG | 12,18 |
|  |  |  | **-347** | **-339** | **CTTCCTCCC** | **0** |
|  |  |  | -278 | -270 | CGGGGGAAG | 8,93 |
|  |  |  | -82 | -74 | CGGGGGAAG | 8,93 |
|  |  |  | -18 | -10 | GAGCGGAAG | 8,76 |
| GRM2 | glutamate receptor, metabotropic 2 | 29419 | -672 | -664 | CTTCCCCAC | 12,04 |
|  |  |  | -108 | -100 | TCCGGGAAG | 11,1 |
|  |  |  | -101 | -93 | AGCGGGAAG | 8,93 |
|  |  |  | **107** | **115** | **CTTCCTGCC** | **0** |
|  |  |  | 122 | 130 | CTTCCGTCT | 7,56 |
|  |  |  | **213** | **221** | **CTTCCTCCC** | **0** |
| GRM4 | glutamate receptor, metabotropic 4 | 36772 | **-696** | **-688** | **CTTCCTTCC** | **0,82** |
|  |  |  | -692 | -684 | CTTCCCTCC | 9,62 |
|  |  |  | -582 | -574 | CTGGGGAAG | 12,18 |
|  |  |  | -548 | -540 | CTTCCAACT | 10,58 |
|  |  |  | -478 | -470 | CTGGGGAAG | 12,18 |
|  |  |  | -430 | -422 | CTTCCATCC | 9,62 |
|  |  |  | -356 | -348 | TGAGGGAAG | 9,75 |
|  |  |  | **-309** | **-301** | **ATGAGGAAG** | **3,38** |
|  |  |  | **-181** | **-173** | **GGGAGGAAG** | **0** |
|  |  |  | -18 | -10 | GGGTGGAAG | 8,8 |
|  |  |  | 72 | 80 | CTTCCCTTG | 11,92 |
|  |  |  | 170 | 178 | TCCCGGAAG | 8,9 |
| GSK3B | glycogen synthase kinase 3 beta | 30585 | **-685** | **-677** | **GGGAGGAAG** | **0** |
|  |  |  | -643 | -635 | GTGGGGAAG | 12,04 |
|  |  |  | **-529** | **-521** | **CAAAGGAAG** | **3,12** |
|  |  |  | **-525** | **-517** | **GGAAGGAAG** | **0,82** |
|  |  |  | **-521** | **-513** | **GGAAGGAAG** | **0,82** |
|  |  |  | **-452** | **-444** | **CTTCCTGAG** | **3,38** |
|  |  |  | -371 | -363 | GGACGGAAG | 7,42 |
|  |  |  | -14 | -6 | CTTCCGCCG | 6,73 |
|  |  |  | -5 | 3 | CTTCCCTTC | 11,78 |
| HES7 | hairy and enhancer of split 7 (Drosophila) | 19084 | **-446** | **-438** | **ACTAGGAAG** | **3,94** |
|  |  |  | -205 | -197 | CTTCCCGCA | 8,93 |
|  |  |  | -176 | -168 | CTTCCCCTC | 10,96 |
|  |  |  | 135 | 143 | GGGTGGAAG | 8,8 |
|  |  |  | **181** | **189** | **GAGAGGAAG** | **2,16** |
|  |  |  | **-131** | **-123** | **CTTCCTAGG** | **3,94** |
|  |  |  | -127 | -119 | CTAGGGAAG | 13 |
|  |  |  | 230 | 238 | CTTCCCCAG | 12,18 |
| HRH2 | histamine receptor H2 | 33754 | -203 | -195 | CTTCCAGCC | 8,8 |
|  |  |  | **-171** | **-163** | **CTTCCTGGG** | **2,3** |
|  |  |  | -47 | -39 | CCTGGGAAG | 12,74 |
|  |  |  | **111** | **119** | **GGGAGGAAG** | **0** |
| HSPB2 | heat shock 27kDa protein 2 | 7060 | **-553** | **-545** | **CTTCCTAGG** | **3,94** |
|  |  |  | -512 | -504 | CCTTGGAAG | 12,74 |
| HTR1D | 5-hydroxytryptamine receptor 1D | 113493 | -638 | -630 | TGTTGGAAG | 10,58 |
|  |  |  | -551 | -543 | AGAGGGAAG | 9,75 |
|  |  |  | -401 | -393 | CTTCCACTG | 11,1 |
|  |  |  | **-243** | **-235** | **CTTCCTTCT** | **0,96** |
|  |  |  | -236 | -228 | CTTCCCCAA | 12,18 |
| HTR5A | 5-hydroxytryptamine (serotonin) receptor 5A | 38515 | **-693** | **-685** | **CTCAGGAAG** | **3,38** |
|  |  |  | -506 | -498 | TAAGGGAAG | 11,92 |
|  |  |  | -454 | -446 | CTTCCGGAG | 9,98 |
|  |  |  | **-400** | **-392** | **TGCAGGAAG** | **0,13** |
|  |  |  | 94 | 102 | CTTCCCTAG | 13 |
| IFNA1 | interferon, alpha 1 | 42928 | **-20** | **-12** | **TGCAGGAAG** | **0,13** |
|  |  |  | **213** | **221** | **CTTCCTCCT** | **0,13** |
| IFNA5 | interferon, alpha 5 | 42932 | **-151** | **-143** | **CAGAGGAAG** | **2,3** |
|  |  |  | 217 | 225 | AATGGGAAG | 12,74 |
|  |  |  | 245 | 253 | CTTCCCCAG | 12,18 |
| IFNAR1 | interferon (alpha, beta and omega) receptor 1 | 27329 | **-534** | **-526** | **CTTCCTCCT** | **0,13** |
|  |  |  | -527 | -519 | CTTCCAGCC | 8,8 |
|  |  |  | -376 | -368 | ATGGGGAAG | 12,18 |
|  |  |  | **-231** | **-223** | **GTGAGGAAG** | **3,25** |
| IL1R1 | interleukin 1 receptor, type I | 23598 | -579 | -571 | TTGTGGAAG | 12,18 |
|  |  |  | -491 | -483 | CTTCCAGAG | 12,18 |
|  |  |  | **-104** | **-96** | **CTTCCTGTT** | **2,3** |
|  |  |  | **-12** | **-4** | **CTTCCTTTG** | **3,12** |
| IRAK3 | interleukin-1 receptor-associated kinase 3 | 9134 | -672 | -664 | TTTGGGAAG | 13,82 |
|  |  |  | **-411** | **-403** | **AAAAGGAAG** | **3,12** |
|  |  |  | **-398** | **-390** | **GAAAGGAAG** | **2,99** |
|  |  |  | -393 | -385 | GAAGGGAAG | 11,78 |
|  |  |  | **-386** | **-378** | **AGAAGGAAG** | **0,96** |
|  |  |  | **-382** | **-374** | **GGAAGGAAG** | **0,82** |
|  |  |  | -42 | -34 | TCGTGGAAG | 11,1 |
| ITGA3 | integrin, alpha 3 (antigen CD49C, alpha 3 subunit of VLA-3 receptor) | 17448 | -457 | -449 | CCCCGGAAG | 8,9 |
|  |  |  | **-212** | **-204** | **GGCAGGAAG** | **0** |
|  |  |  | -189 | -181 | CTTAGGAAG | 5,03 |
|  |  |  | -11 | -3 | CTTCCGCTG | 8,9 |
| KLK3 | kallikrein 3 | 21176 | -150 | -142 | CTTCCACAG | 12,18 |
|  |  |  | **50** | **58** | **CTTCCTCAC** | **3,25** |
| KREMEN2 | kringle containing transmembrane protein 2 | 14675 | -608 | -600 | AGCTGGAAG | 8,93 |
|  |  |  | -361 | -353 | CTTCCCCCG | 8,93 |
|  |  |  | -112 | -104 | CCAGGGAAG | 11,92 |
|  |  |  | **41** | **49** | **CTTCCTCCG** | **0,13** |
|  |  |  | 251 | 259 | CTTCCGTCC | 7,42 |
|  |  |  | 287 | 295 | CTTCCCACT | 10,58 |
| LIF | leukemia inhibitory factor | 28861 | -579 | -571 | CTTCCAGCG | 8,93 |
|  |  |  | -149 | -141 | CTTCCATTC | 11,78 |
|  |  |  | **-112** | **-104** | **CTTCCTGGA** | **2,3** |
| LIFR | leukemia inhibitory factor receptor | 34694 | -532 | -524 | CTCTGGAAG | 12,18 |
|  |  |  | **-349** | **-341** | **TCTAGGAAG** | **3,94** |
|  |  |  | 101 | 109 | CCAGGGAAG | 11,92 |
| MAP3K1 | mitogen-activated protein kinase kinase kinase 1, E3 ubiquitin protein ligase | 33060 | **-622** | **-614** | **GAAAGGAAG** | **2,99** |
|  |  |  | **-618** | **-610** | **GGAAGGAAG** | **0,82** |
|  |  |  | -416 | -408 | GCAGGGAAG | 11,78 |
|  |  |  | **-337** | **-329** | **CTTCCTGGC** | **2,16** |
|  |  |  | **-221** | **-213** | **CTTCCTCGC** | **2,16** |
|  |  |  | 131 | 199 | TTTCGGAAG | 11,63 |
|  |  |  | **247** | **255** | **GAGAGGAAG** | **2,16** |
| MAP3K2 | mitogen-activated protein kinase kinase kinase 2 | 117737 | **13** | **21** | **AAAAGGAAG** | **3,12** |
| MAP3K3 | mitogen-activated protein kinase kinase kinase 3 | 17578 | **-439** | **-431** | **CTTCCTTCT** | **0,96** |
|  |  |  | 287 | 295 | CCGGGGAAG | 11,1 |
| MAP3K5 | mitogen-activated protein kinase kinase kinase 5 | 36258 | -680 | -672 | CTTAGGAAG | 5,03 |
|  |  |  | **-584** | **-576** | **CTTCCTGGA** | **2,3** |
|  |  |  | **-505** | **-497** | **TCTAGGAAG** | **3,94** |
|  |  |  | **34** | **42** | **CTTCCTTGC** | **2,99** |
|  |  |  | 81 | 89 | CTTCCCATG | 12,74 |
|  |  |  | 97 | 105 | CTTCCATGA | 11,92 |
|  |  |  | 117 | 125 | AGAGGGAAG | 9,75 |
| MAP3K14 | mitogen-activated protein kinase kinase kinase 14 | 18485 | **-529** | **-521** | **GAGAGGAAG** | **2,16** |
|  |  |  | **-426** | **-418** | **CTTCCTTCC** | **0,82** |
|  |  |  | -422 | -414 | CTTCCCGTA | 11,1 |
|  |  |  | -231 | -223 | AGCGGGAAG | 8,93 |
| MAPK6 | mitogen-activated protein kinase 6 | 13305 | -641 | -633 | CTTCCCAAA | 13,82 |
|  |  |  | **-484** | **-476** | **CTTCCTCCA** | **0,13** |
|  |  |  | -408 | -400 | CTTCCAGCC | 8,8 |
|  |  |  | **-379** | **-371** | **CTTCCTGGA** | **2,3** |
|  |  |  | **-359** | **-351** | **CTTCCTCTC** | **2,16** |
|  |  |  | **-238** | **-230** | **CTTCCTCCC** | **0** |
|  |  |  | **-30** | **-22** | **CTTCCTCGC** | **2,16** |
| MC3R | melanocortin 3 receptor | 26400 | -679 | -671 | CTTCCAAGT | 12,74 |
|  |  |  | -495 | -487 | CTTCCACAG | 12,18 |
|  |  |  | **-44** | **-36** | **CTTCCTCCT** | **0,13** |
|  |  |  | **-13** | **-5** | **CTTCCTCCA** | **0,13** |
|  |  |  | **23** | **31** | **CTTCCTCTT** | **2,3** |
|  |  |  | 85 | 93 | CTTCCACCC | 8,8 |
|  |  |  | 106 | 114 | TCGGGGAAG | 11,1 |
| MEF2B | MADS box transcription enhancer factor 2, polypeptide B | 22310 | -648 | -640 | AGAGGGAAG | 9,75 |
|  |  |  | -635 | -627 | TGGTGGAAG | 8,93 |
|  |  |  | **-211** | **-203** | **CTTCCTTAT** | **4,2** |
|  |  |  | 264 | 272 | GTCTGGAAG | 12,04 |
| MEF2C | MADS box transcription enhancer factor 2, polypeptide C | 34461 | -382 | -374 | CTTCCCTAC | 12,87 |
|  |  |  | **-254** | **-246** | **CTTCCTTCC** | **0,82** |
|  |  |  | **-250** | **-242** | **CTTCCTGGA** | **2,3** |
|  |  |  | **-156** | **-148** | **CTTCCTCCT** | **0,13** |
|  |  |  | **-143** | **-135** | **CTTCCTCCT** | **0,13** |
|  |  |  | -125 | -117 | CTTCCGAGC | 10,41 |
|  |  |  | **214** | **222** | **CTTCCTTCA** | **0,96** |
| MEF2D | MADS box transcription enhancer factor 2, polypeptide D | 2826 | -473 | -465 | TTTGGGAAG | 13,82 |
|  |  |  | **-321** | **-313** | **CTTCCTTGA** | **3,12** |
|  |  |  | -297 | -289 | CTTCCGAGA | 10,54 |
|  |  |  | 6 | 14 | CTTCCGAGG | 10,54 |
|  |  |  | **24** | **32** | **GAAAGGAAG** | **2,99** |
| MFNG | manic fringe homolog (Drosophila) | 28754 | -423 | -415 | TCACGGAAG | 9,72 |
|  |  |  | **-413** | **-405** | **CTTCCTGGA** | **2,3** |
|  |  |  | -196 | -188 | CTTCCACCG | 8,93 |
|  |  |  | **-166** | **-158** | **CTTCCTCCC** | **0** |
|  |  |  | 73 | 81 | CTTCCCTCC | 9,62 |
|  |  |  | 140 | 148 | CTTCCCCTC | 10,96 |
| MMP2 | matrix metallopeptidase 2 (gelatinase A, 72kDa gelatinase, 72kDa type IV collagenase) | 15206 | **-543** | **-535** | **GTAAGGAAG** | **4,07** |
|  |  |  | -523 | -515 | CTTCCACTG | 11,1 |
|  |  |  | -386 | -378 | CTTCCCGTT | 11,1 |
|  |  |  | **17** | **25** | **CTTCCTCAG** | **3,38** |
| MMP19 | matrix metallopeptidase 19 | 10097 | -617 | -609 | CTTCCCAGG | 12,74 |
|  |  |  | -147 | -139 | TGAGGGAAG | 9,75 |
|  |  |  | -118 | -110 | CTTCCGCTC | 8,76 |
|  |  |  | **144** | **152** | **CTTCCTACT** | **1,78** |
| MMP20 | matrix metallopeptidase 20 | 116369 | -653 | -645 | CTTCCATTT | 11,92 |
|  |  |  | **-544** | **-536** | **AGAAGGAAG** | **0,96** |
|  |  |  | -398 | -390 | CTTCCAAAG | 13,82 |
|  |  |  | -350 | -342 | CTTCCAATT | 12,74 |
|  |  |  | **-176** | **-168** | **AGGAGGAAG** | **0,13** |
| MMP28 | matrix metallopeptidase 28 | 18766 | **-242** | **-234** | **CTTCCTTCA** | **0,96** |
|  |  |  | -142 | -134 | GAACGGAAG | 9,59 |
|  |  |  | **-46** | **-38** | **CTTCCTCCC** | **0** |
| NANOG | Nanog homeobox | 8675 | -472 | -464 | CTTCCAGAG | 12,18 |
|  |  |  | 134 | 142 | CTTCCAGAA | 12,18 |
|  |  |  | **200** | **208** | **CTTCCTCTA** | **2,3** |
| NFATC1 | nuclear factor of activated T-cells, cytoplasmic, calcineurin-dependent 1 | 19607 | **-627** | **-619** | **GCCAGGAAG** | **2,16** |
|  |  |  | **-334** | **-326** | **AGGAGGAAG** | **0,13** |
|  |  |  | **-28** | **-20** | **CGGAGGAAG** | **0,13** |
|  |  |  | **-4** | **4** | **AGGAGGAAG** | **0,13** |
|  |  |  | 274 | 282 | GGCGGGAAG | 8,8 |
| NGFR | nerve growth factor receptor | 17440 | -471 | -463 | ACTGGGAAG | 12,74 |
|  |  |  | **-334** | **-326** | **CGGAGGAAG** | **0,13** |
|  |  |  | -105 | -97 | CTTCCCACC | 10,44 |
|  |  |  | 99 | 107 | AGCTGGAAG | 8,93 |
| NLK | nemo like kinase | 17003 | -578 | -570 | CTTCCCTAA | 13 |
|  |  |  | -347 | -339 | CTTCCAGCT | 8,93 |
|  |  |  | **-314** | **-306** | **CTTCCTGCT** | **0,13** |
|  |  |  | -129 | -121 | CCCCGGAAG | 8,9 |
|  |  |  | 159 | 167 | GGACGGAAG | 7,42 |
| NODAL | nodal growth differentiation factor | 5694 | -686 | -678 | TCGGGGAAG | 11,1 |
|  |  |  | -569 | -561 | CTTCCCCAG | 12,18 |
|  |  |  | **-432** | **-424** | **CTTCCTGCT** | **0,13** |
|  |  |  | **-353** | **-345** | **GTAAGGAAG** | **4,07** |
|  |  |  | -94 | -86 | CTTCCCCAG | 12,18 |
|  |  |  | **20** | **28** | **CTTCCTTCT** | **0,96** |
|  |  |  | 262 | 270 | CTCGGGAAG | 12,18 |
| NOS1 | nitric oxide synthase 1 (neuronal) | 9735 | **-569** | **-561** | **GAAAGGAAG** | **2,99** |
|  |  |  | -564 | -556 | GAAGGGAAG | 11,78 |
|  |  |  | -540 | -532 | CTTCCCTCC | 9,62 |
| NOS2 | nitric oxide synthase 2, inducible | 18883 | -669 | -661 | TAAGGGAAG | 11,92 |
|  |  |  | **-561** | **-553** | **CTTCCTTCC** | **0,82** |
|  |  |  | **-557** | **-549** | **CTTCCTTTC** | **2,99** |
|  |  |  | -547 | -539 | CTTCCAAAA | 13,82 |
|  |  |  | **-424** | **-416** | **CTTCCTGGA** | **2,3** |
|  |  |  | **138** | **146** | **CTTCCTGGT** | **2,3** |
|  |  |  | 207 | 215 | CTGGGGAAG | 12,18 |
|  |  |  | **241** | **249** | **CTTCCTCTT** | **2,3** |
| NOTCH3 | notch 3 | 22400 | **-683** | **-675** | **CAGAGGAAG** | **2,3** |
|  |  |  | -182 | -174 | CTGGGGAAG | 12,18 |
|  |  |  | 259 | 267 | CCAGGGAAG | 11,92 |
| NOTCH4 | notch 4 | 36860 | **-599** | **-591** | **AACAGGAAG** | **2,3** |
|  |  |  | -195 | -187 | CTTCCCCCT | 8,93 |
|  |  |  | -185 | -177 | CTTCCCCAG | 12,18 |
|  |  |  | **-123** | **-115** | **CTTCCTCCG** | **0,13** |
|  |  |  | **-40** | **-32** | **AGGAGGAAG** | **0,13** |
|  |  |  | 206 | 214 | TGGGGGAAG | 8,93 |
| NTF3 | neurotrophin 3 | 8613 | -606 | -598 | TGCGGGAAG | 8,93 |
|  |  |  | **-469** | **-461** | **CTGAGGAAG** | **3,38** |
|  |  |  | -271 | -263 | CTTCCCACC | 10,44 |
|  |  |  | -63 | -55 | CTTCCCAGT | 12,74 |
|  |  |  | **3** | **11** | **GGGAGGAAG** | **0** |
|  |  |  | 50 | 58 | CTCGGGAAG | 12,18 |
|  |  |  | 81 | 89 | CTTCCGGGC | 8,76 |
|  |  |  | 287 | 295 | GTGGGGAAG | 12,04 |
| NTRK3 | neurotrophic tyrosine kinase, receptor, type 3 | 13873 | **-436** | **-428** | **CTTCCTACC** | **1,65** |
|  |  |  | **-18** | **-10** | **GGGAGGAAG** | **0** |
|  |  |  | **3** | **11** | **CCGAGGAAG** | **2,3** |
|  |  |  | **114** | **122** | **CTTCCTTCT** | **0,96** |
|  |  |  | **123** | **131** | **CTTCCTTTC** | **2,99** |
| NUMBL | numb homolog (Drosophila)-like | 22010 | **-429** | **-421** | **CTTCCTTGC** | **2,99** |
|  |  |  | -362 | -354 | AGCGGGAAG | 8,93 |
|  |  |  | -159 | -151 | CCGGGGAAG | 11,1 |
| ONECUT1 | one cut domain, family member 1 | 14193 | -501 | -493 | GCAGGGAAG | 11,78 |
|  |  |  | -108 | -100 | GAGGGGAAG | 10,96 |
|  |  |  | -89 | -81 | AGAGGGAAG | 9,75 |
|  |  |  | **8** | **16** | **GAGAGGAAG** | **2,16** |
|  |  |  | **12** | **20** | **GGAAGGAAG** | **0,82** |
|  |  |  | 143 | 151 | CCATGGAAG | 11,92 |
| OPRD1 | opioid receptor, delta 1 | 450 | -665 | -657 | CCAGGGAAG | 11,92 |
|  |  |  | **-548** | **-540** | **CCCAGGAAG** | **2,3** |
| OPRM1 | opioid receptor, mu 1 | 35924 | **-678** | **-670** | **TCGAGGAAG** | **2,3** |
|  |  |  | -393 | -385 | GTAGGGAAG | 12,87 |
|  |  |  | -273 | -265 | GGATGGAAG | 9,62 |
|  |  |  | 1 | 9 | CTTCCAGCC | 8,8 |
|  |  |  | **209** | **217** | **GAAAGGAAG** | **2,99** |
| PAX6 | paired box gene 6 | 8176 | -605 | -597 | CTTCCAAGG | 12,74 |
|  |  |  | **-30** | **-22** | **AGGAGGAAG** | **0,13** |
|  |  |  | 121 | 129 | CTTCCCTCA | 9,75 |
|  |  |  | 194 | 202 | CTTCCCAGG | 12,74 |
|  |  |  | -289 | -281 | GGCGGGAAG | 8,8 |
|  |  |  | **-83** | **-75** | **GAAAGGAAG** | **2,99** |
|  |  |  | 61 | 69 | GGTTGGAAG | 10,44 |
| PDE4C | phosphodiesterase 4C, cAMP-specific (phosphodiesterase E1 dunce homolog, Drosophila) | 22338 | **-520** | **-512** | **CTTCCTGGG** | **2,3** |
|  |  |  | -457 | -449 | CTTCCAGAC | 12,04 |
|  |  |  | -430 | -422 | CTGGGGAAG | 12,18 |
| PDE6C | phosphodiesterase 6C, cGMP-specific, cone, alpha prime | 4773 | -352 | -344 | CTTCCGAGG | 10,54 |
|  |  |  | **-329** | **-321** | **CTTCCTATA** | **3,94** |
|  |  |  | **-248** | **-240** | **CTTCCTTGC** | **2,99** |
|  |  |  | **-84** | **-76** | **CTTCCTGAA** | **3,38** |
|  |  |  | 0 | 8 | CTTTGGAAG | 13,82 |
|  |  |  | **210** | **218** | **GACAGGAAG** | **2,16** |
| PDE6G | phosphodiesterase 6G, cGMP-specific, rod, gamma | 17955 | -614 | -606 | CTTCCATCT | 9,75 |
|  |  |  | **-573** | **-565** | **CTTCCTGTG** | **2,3** |
|  |  |  | -481 | -473 | CTTGGGAAG | 13,82 |
|  |  |  | -256 | -248 | CTTCCATCT | 9,75 |
|  |  |  | -251 | -243 | ATCTGGAAG | 12,18 |
|  |  |  | **-169** | **-161** | **CTTCCTTGG** | **3,12** |
|  |  |  | 83 | 91 | CTTCCCCCA | 8,93 |
|  |  |  | 254 | 262 | CTTCCACCA | 8,93 |
| PDGFRB | platelet-derived growth factor receptor, beta polypeptide | 34118 | -49 | -41 | AAGGGGAAG | 11,1 |
|  |  |  | **-22** | **-14** | **AGGAGGAAG** | **0,13** |
|  |  |  | 111 | 119 | CTTCCCCAC | 12,04 |
| PDPK1 | 3-phosphoinositide dependent protein kinase-1 | 14659 | **-121** | **-113** | **TGTAGGAAG** | **1,78** |
|  |  |  | **-104** | **-96** | **TGCAGGAAG** | **0,13** |
|  |  |  | **20** | **28** | **CTTCCTGAG** | **3,38** |
| PGK1 | phosphoglycerate kinase 1 | 43460 | -575 | -567 | CTTCCAACT | 10,58 |
|  |  |  | **-264** | **-256** | **CTTCCTGCT** | **0,13** |
|  |  |  | -245 | -237 | GTCGGGAAG | 12,04 |
|  |  |  | -203 | -195 | AAACGGAAG | 9,72 |
|  |  |  | -77 | -69 | GCCGGGAAG | 10,96 |
|  |  |  | 127 | 135 | AAAGGGAAG | 11,92 |
| PIK3C2B | phosphatidylinositol-4-phosphate 3-kinase, catalytic subunit type 2 beta | 2443 | -656 | -648 | GCTCGGAAG | 10,41 |
|  |  |  | -610 | -602 | CTTCCAGAA | 12,18 |
|  |  |  | **-600** | **-592** | **GGGAGGAAG** | **0** |
|  |  |  | **-596** | **-588** | **GGAAGGAAG** | **0,82** |
|  |  |  | -281 | -273 | CTTCCCCCA | 8,93 |
|  |  |  | -214 | -206 | AGGGGGAAG | 8,93 |
|  |  |  | -37 | -29 | CTTCCCCTC | 10,96 |
| PKIG | protein kinase (cAMP-dependent, catalytic) inhibitor gamma | 26303 | -93 | -85 | CTTCCCAAA | 13,82 |
|  |  |  | **127** | **135** | **TGAAGGAAG** | **0,96** |
| PLA2G5 | phospholipase A2, group V | 270 | **-171** | **-163** | **CTTCCTGCC** | **0** |
|  |  |  | **61** | **69** | **CCAAGGAAG** | **3,12** |
| PLA2G6 | phospholipase A2, group VI (cytosolic, calcium-independent) | 28730 | -629 | -621 | CTTCCCGGG | 11,1 |
|  |  |  | **-280** | **-272** | **GTCAGGAAG** | **3,25** |
|  |  |  | -11 | -3 | CTTCCAGAC | 12,04 |
|  |  |  | 0 | 8 | AGGGGGAAG | 8,93 |
|  |  |  | **156** | **64** | **GGGAGGAAG** | **0** |
|  |  |  | 190 | 198 | CTTCCCTGG | 11,92 |
| PLA2G2D | phospholipase A2, group IIA (platelets, synovial fluid) | 3894 | -590 | -582 | AGTGGGAAG | 10,58 |
|  |  |  | **-484** | **-476** | **CTTCCTGTG** | **2,3** |
|  |  |  | -148 | -140 | GAGGGGAAG | 10,96 |
|  |  |  | 286 | 294 | ATGTGGAAG | 12,18 |
| POU5F1 | POU class 5 homeobox 1 | 36944 | **-529** | **-521** | **CTTCCTTTT** | **3,12** |
|  |  |  | -8 | 0 | TGGGGGAAG | 8,93 |
|  |  |  | 90 | 98 | GCTTGGAAG | 12,61 |
| PPP1R14D | protein phosphatase 1, regulatory (inhibitor) subunit 14D | 14305 | -700 | -692 | CTTCCTAAG | 5,03 |
|  |  |  | **-403** | **-395** | **CCCAGGAAG** | **2,3** |
|  |  |  | **-291** | **-283** | **CTTCCTCCT** | **0,13** |
|  |  |  | -30 | -22 | CTTCCCTCT | 9,75 |
|  |  |  | 31 | 39 | CTTCCATGC | 11,78 |
|  |  |  | **55** | **63** | **CTTCCTGCA** | **0,13** |
|  |  |  | 284 | 292 | CTTCCAGGT | 11,1 |
| PPP2R2C | protein phosphatase 2, regulatory subunit B, gamma | 32700 | **-677** | **-669** | **CTTCCTTCC** | **0,82** |
|  |  |  | **-673** | **-665** | **CTTCCTAGT** | **3,94** |
|  |  |  | -622 | -614 | CTTCCCAGC | 12,61 |
|  |  |  | **-508** | **-500** | **CTTCCTCCC** | **0** |
|  |  |  | **91** | **99** | **CTTCCTGCG** | **0,13** |
| PRKCA | protein kinase C, alpha | 17595 | **-684** | **-676** | **AGAAGGAAG** | **0,96** |
|  |  |  | -647 | -639 | GAGCGGAAG | 8,76 |
|  |  |  | **-589** | **-581** | **CGCAGGAAG** | **0,13** |
|  |  |  | **-243** | **-235** | **AGTAGGAAG** | **1,78** |
| PSEN1 | presenilin 1 | 11969 | -135 | -127 | GTGGGGAAG | 12,04 |
|  |  |  | **-67** | **-59** | **CTTCCTCCT** | **0,13** |
|  |  |  | 81 | 89 | GTCTGGAAG | 12,04 |
|  |  |  | 125 | 133 | GCGGGGAAG | 10,96 |
|  |  |  | **163** | **171** | **GCCAGGAAG** | **2,16** |
| PSEN2 | presenilin 2 | 1943 | **-405** | **-397** | **CGCAGGAAG** | **0,13** |
|  |  |  | -16 | -8 | CTTCCCCGG | 11,1 |
|  |  |  | 104 | 112 | TCGTGGAAG | 11,1 |
|  |  |  | 159 | 167 | CTTCCCTGC | 11,78 |
| PTGER3 | prostaglandin E receptor 3 (subtype EP3) | 3343 | -151 | -143 | CTTCCCAAC | 13,69 |
|  |  |  | **14** | **22** | **GAGAGGAAG** | **2,16** |
| PTK2B | protein tyrosine kinase 2 beta | 39829 | -633 | -625 | CCCTGGAAG | 11,1 |
|  |  |  | **-87** | **-79** | **TTAAGGAAG** | **4,2** |
| PTPN6 | protein tyrosine phosphatase, non-receptor type 6 | 8664 | -167 | -159 | TGTGGGAAG | 10,58 |
|  |  |  | -116 | -108 | CTTCCCTTG | 11,92 |
|  |  |  | **-52** | **-44** | **CTTCCTGTC** | **2,16** |
|  |  |  | **116** | **124** | **CTTCCTCTC** | **2,16** |
|  |  |  | 122 | 130 | CTCCGGAAG | 9,98 |
| RARA | retinoic acid receptor, alpha | 17216 | -656 | -648 | CTTCCCTAA | 13 |
|  |  |  | **-436** | **-428** | **ATGAGGAAG** | **3,38** |
|  |  |  | **-290** | **-282** | **GGCAGGAAG** | **0** |
|  |  |  | -168 | -160 | CTTCCAGTG | 11,1 |
|  |  |  | -101 | -93 | TGGTGGAAG | 8,93 |
|  |  |  | 23 | 31 | CTTCCCCGG | 11,1 |
|  |  |  | 97 | 105 | CTTCCCAAT | 13,82 |
|  |  |  | **277** | **285** | **CTTCCTGCA** | **0,13** |
| RARG | retinoic acid receptor, gamma | 10178 | -673 | -665 | GACGGGAAG | 10,96 |
|  |  |  | **-669** | **-661** | **GGAAGGAAG** | **0,82** |
|  |  |  | -389 | -381 | CTTCCAACC | 10,44 |
|  |  |  | 81 | 89 | CTTCCCCGC | 10,96 |
| RELA | v-rel reticuloendotheliosis viral oncogene homolog A | 7862 | **164** | **172** | **CTTCCTGCG** | **0,13** |
| RGS12 | regulator of G-protein signalling 12 | 31208 | **-484** | **-476** | **CTTCCTTCA** | **0,96** |
|  |  |  | **-394** | **-386** | **CTTCCTCAT** | **3,38** |
| RHOG | ras homolog gene family, member G | 8387 | -660 | -652 | CGAGGGAAG | 9,75 |
|  |  |  | -494 | -486 | CTTCCCCCC | 8,8 |
|  |  |  | -453 | -445 | CTTCCCACT | 10,58 |
|  |  |  | -316 | -308 | CTTCCCGCG | 8,93 |
|  |  |  | -287 | -279 | GGGGGGAAG | 8,8 |
|  |  |  | **-79** | **-71** | **CTTCCTGGT** | **2,3** |
|  |  |  | **-24** | **-16** | **CTTCCTCCG** | **0,13** |
|  |  |  | **-1** | **7** | **CTTCCTTCT** | **0,96** |
|  |  |  | 270 | 278 | CTTCCCGGC | 10,96 |
| RPS6KA4 | ribosomal protein S6 kinase, 90kDa, polypeptide 4 | 6674 | -422 | -414 | CTTCCAGGA | 11,1 |
|  |  |  | **-32** | **-24** | **ACCAGGAAG** | **2,3** |
| RXRA | retinoid X receptor, alpha | 42036 | **-691** | **-683** | **CTCAGGAAG** | **3,38** |
|  |  |  | **-565** | **-557** | **CTTCCTTTC** | **2,99** |
|  |  |  | -311 | -303 | GTCCGGAAG | 9,85 |
|  |  |  | -295 | -287 | CTTCCCCGG | 11,1 |
| RXRB | retinoid X receptor, beta | 36804 | **-225** | **-217** | **AACAGGAAG** | **2,3** |
|  |  |  | -22 | -14 | CTTCCCGTC | 10,96 |
|  |  |  | **87** | **95** | **CTTCCTCGT** | **2,3** |
|  |  |  | **193** | **201** | **CTTCCTCCC** | **0** |
| SET | SET nuclear proto-oncogene | 41931 | **-512** | **-504** | **GTCAGGAAG** | **3,25** |
|  |  |  | **-274** | **-266** | **TCAAGGAAG** | **3,12** |
|  |  |  | -120 | -112 | CTTCCAGGC | 10,96 |
|  |  |  | -50 | -42 | CTTCCGGAC | 9,85 |
|  |  |  | -10 | -2 | CTTCCCTAA | 13 |
| SFRP5 | secreted frizzled-related protein 5 | 5477 | -384 | -376 | TCGGGGAAG | 11,1 |
|  |  |  | **-280** | **-272** | **AGGAGGAAG** | **0,13** |
| SHC1 | SHC (Src homology 2 domain containing) transforming protein 1 | 2898 | **-670** | **-662** | **CTTCCTGAT** | **3,38** |
|  |  |  | **-614** | **-606** | **CTTCCTCTT** | **2,3** |
|  |  |  | -574 | -566 | CTTCCCATC | 12,61 |
|  |  |  | -414 | -406 | GACAGGAAG | 2,16 |
|  |  |  | **-320** | **-312** | **CTTCCTGCC** | **0** |
|  |  |  | **-261** | **-253** | **CTTCCTTTC** | **2,99** |
|  |  |  | -243 | -235 | CTTCCATCC | 9,62 |
|  |  |  | -235 | -227 | CTTCCCTCC | 9,62 |
|  |  |  | 60 | 68 | GAGCGGAAG | 8,76 |
|  |  |  | **282** | **290** | **TGGAGGAAG** | **0,13** |
| SIX3 | sine oculis homeobox homolog 3 (Drosophila) | 23219 | **-559** | **-551** | **CTTCCTTGC** | **2,99** |
|  |  |  | -326 | -318 | AAAGGGAAG | 11,92 |
|  |  |  | -187 | -179 | CTTCCCTGC | 11,78 |
|  |  |  | **227** | **235** | **CTTCCTCCT** | **0,13** |
| SLC2A5 | solute carrier family 2 (facilitated glucose/fructose transporter), member 5 | 4038 | -570 | -562 | AGGTGGAAG | 8,93 |
|  |  |  | -232 | -224 | CTTCCAGTC | 10,96 |
|  |  |  | **-79** | **-71** | **GAAAGGAAG** | **2,99** |
|  |  |  | 82 | 90 | CTTCCAGAG | 12,18 |
|  |  |  | **118** | **126** | **TGAAGGAAG** | **0,96** |
|  |  |  | **190** | **198** | **CTTCCTGGA** | **2,3** |
| SMAD6 | SMAD family member 6 | 13377 | **-531** | **-523** | **TGCAGGAAG** | **0,13** |
|  |  |  | **-88** | **-80** | **GAGAGGAAG** | **2,16** |
| SMPD1 | sphingomyelin phosphodiesterase 1 | 6236 | **-549** | **-541** | **CTTCCTAGT** | **3,94** |
|  |  |  | -439 | -431 | CGAGGGAAG | 9,75 |
|  |  |  | **-423** | **-418** | **CTTCCTGTC** | **2,16** |
|  |  |  | -362 | -354 | CTTCCCGGG | 11,1 |
|  |  |  | -286 | -278 | CTTCCACCG | 8,93 |
|  |  |  | **-47** | **-39** | **CAGAGGAAG** | **2,3** |
|  |  |  | **-41** | **-33** | **AAGAGGAAG** | **2,3** |
|  |  |  | 109 | 117 | TGTAGGAAG | 1,78 |
| SMPD2 | sphingomyelin phosphodiesterase 2 | 35713 | **-667** | **-659** | **CTTCCTCGG** | **2,3** |
|  |  |  | 205 | 213 | CTTCCGTTC | 9,59 |
|  |  |  | **225** | **235** | **CTTCCTCCC** | **0** |
| SOD3 | superoxide dismutase 3, extracellular | 31316 | **-491** | **-483** | **GATAGGAAG** | **3,81** |
|  |  |  | 193 | 201 | CTTGGGAAG | 13,82 |
|  |  |  | **256** | **264** | **GGGAGGAAG** | **0** |
|  |  |  | **273** | **281** | **GGGAGGAAG** | **0** |
| SOX2 | SRY (sex determining region Y)-box 2 | 29975 | -690 | -682 | TATGGGAAG | 12,74 |
|  |  |  | -408 | -400 | GAGTGGAAG | 10,96 |
|  |  |  | -156 | -148 | CTTCCCCAG | 12,18 |
|  |  |  | **99** | **107** | **GCCAGGAAG** | **2,16** |
|  |  |  | 148 | 156 | CTTCCGTCT | 7,56 |
| SOX4 | SRY (sex determining region Y)-box 4 | 35024 | -697 | -689 | CTTCCCTGC | 11,78 |
|  |  |  | -688 | -680 | CTTCCACAA | 12,18 |
|  |  |  | -528 | -520 | CTTCCCCTT | 11,1 |
|  |  |  | -404 | -396 | CTTCCATTT | 11,92 |
|  |  |  | -92 | -84 | AATGGGAAG | 12,74 |
|  |  |  | **-64** | **-56** | **ATGAGGAAG** | **3,38** |
|  |  |  | 51 | 59 | GAGGGGAAG | 10,96 |
|  |  |  | **210** | **218** | **CTTCCTACC** | **1,65** |
| SOX10 | SRY (sex determining region Y)-box 10 | 28735 | -306 | -298 | CTTCCATCC | 9,62 |
|  |  |  | **-268** | **-260** | **CTTCCTTCC** | **0,82** |
|  |  |  | **-264** | **-256** | **CTTCCTCGG** | **2,3** |
|  |  |  | -96 | -88 | CTTCCCTCC | 9,62 |
|  |  |  | **-59** | **-51** | **CTTCCTTCC** | **0,82** |
|  |  |  | -55 | -47 | CTTCCCCAG | 12,18 |
|  |  |  | 150 | 158 | CTTCCTAAG | 5,03 |
| SOX14 | SRY (sex determining region Y)-box 14 | 29803 | -570 | -562 | AAATGGAAG | 11,92 |
|  |  |  | **-425** | **-417** | **AAGAGGAAG** | **2,3** |
|  |  |  | **-335** | **-327** | **CTTCCTTCA** | **0,96** |
|  |  |  | -326 | -318 | CTTCCGGGA | 8,9 |
|  |  |  | 218 | 226 | GCCTGGAAG | 10,96 |
| SRC | v-src sarcoma (Schmidt-Ruppin A-2) viral oncogene homolog | 26243 | -450 | -442 | CTTCCCAAG | 13,82 |
|  |  |  | -422 | -414 | GAACGGAAG | 9,59 |
|  |  |  | -76 | -68 | CTTCCGTTC | 9,59 |
|  |  |  | **-56** | **-48** | **CCGAGGAAG** | **2,3** |
| SRF | serum response factor | 35472 | -608 | -600 | AAAGGGAAG | 11,92 |
|  |  |  | -603 | -595 | GAAGGGAAG | 11,78 |
|  |  |  | -491 | -483 | CTTAGGAAG | 5,03 |
|  |  |  | -329 | -321 | CTTCCGCAA | 9,98 |
|  |  |  | -9 | -1 | GGGGGGAAG | 8,8 |
|  |  |  | 280 | 288 | GCCGGGAAG | 10,96 |
| SSTR3 | somatostatin receptor 3 | 28762 | -598 | -590 | CTTCCACTC | 10,96 |
|  |  |  | **-85** | **-77** | **CTTCCTCCC** | **0** |
|  |  |  | **174** | **182** | **CTTCCTCAC** | **3,25** |
|  |  |  | 183 | 191 | CCTGGGAAG | 12,74 |
|  |  |  | 265 | 273 | GTTGGGAAG | 13,69 |
| T | T brachyury transcription factor | 36104 | -662 | -654 | CTTCCCCAC | 12,04 |
|  |  |  | **-570** | **-562** | **CTTCCTGAC** | **3,25** |
|  |  |  | -500 | -492 | CTTCCCCGG | 11,1 |
|  |  |  | -414 | -406 | CTTCCCCAG | 12,18 |
|  |  |  | -124 | -116 | CCTTGGAAG | 12,74 |
|  |  |  | -99 | -91 | CTTCCCCCT | 8,93 |
|  |  |  | **-92** | **-84** | **CTTCCTGGG** | **2,3** |
|  |  |  | 128 | 136 | CTTCCGTGA | 9,72 |
| TAB1 | TGF-beta activated kinase 1/MAP3K7 binding protein 1 | 28333 | -330 | -322 | CTCGGGAAG | 12,18 |
|  |  |  | **-321** | **-313** | **CTGAGGAAG** | **3,38** |
|  |  |  | -299 | -291 | GGCCGGAAG | 6,6 |
| TCF3 | transcription factor 3 | 22828 | **-425** | **-417** | **CGCAGGAAG** | **0,13** |
|  |  |  | **-28** | **-20** | **CGGAGGAAG** | **0,13** |
| TCF7L1 | transcription factor 7-like 1 | 23441 | **-659** | **-651** | **CTTCCTCGG** | **2,3** |
|  |  |  | **-394** | **-386** | **GGCAGGAAG** | **0** |
|  |  |  | **-167** | **-159** | **GGGAGGAAG** | **0** |
|  |  |  | -8 | 0 | CTTCCCAGG | 12,74 |
|  |  |  | 147 | 155 | CCTTGGAAG | 12,74 |
|  |  |  | 291 | 299 | GCCCGGAAG | 8,76 |
| TDGF1 | teratocarcinoma-derived growth factor 1 | 29345 | **-477** | **-469** | **CTTCCTAGT** | **3,94** |
|  |  |  | -468 | -460 | CTTCCCCAC | 12,04 |
|  |  |  | -357 | -349 | AGGTGGAAG | 8,93 |
|  |  |  | **-343** | **-335** | **TTCAGGAAG** | **3,38** |
|  |  |  | **-329** | **-321** | **CTTCCTGAA** | **3,38** |
|  |  |  | -97 | -89 | CTTCCCGCG | 8,93 |
|  |  |  | **245** | **253** | **TGCAGGAAG** | **0,13** |
| TGFB1 | transforming growth factor, beta 1 | 21997 | **-620** | **-612** | **CTTCCTGGG** | **2,3** |
|  |  |  | **-408** | **-400** | **CGGAGGAAG** | **0,13** |
| TLE3 | transducin-like enhancer of split 3 | 14076 | -598 | -590 | GAGGGGAAG | 10,96 |
|  |  |  | **-594** | **-586** | **GGAAGGAAG** | **0,82** |
|  |  |  | **-590** | **-582** | **GGAAGGAAG** | **0,82** |
|  |  |  | -435 | -427 | CTTCCCGTG | 11,1 |
|  |  |  | -361 | -353 | CCTGGGAAG | 12,74 |
|  |  |  | **-196** | **-188** | **CTTCCTCTT** | **2,3** |
|  |  |  | 37 | 45 | CCAGGGAAG | 11,92 |
| TLN1 | talin 1 | 42835 | -641 | -633 | CTTCCCAGT | 12,74 |
|  |  |  | **-449** | **-441** | **AGCAGGAAG** | **0,13** |
|  |  |  | **-26** | **-18** | **AAAAGGAAG** | **3,12** |
|  |  |  | **49** | **57** | **CTTCCTGCC** | **0** |
|  |  |  | **115** | **123** | **CTTCCTGCC** | **0** |
|  |  |  | 185 | 193 | GGACGGAAG | 7,42 |
| TNFRSF10A | tumor necrosis factor receptor superfamily, member 10a | 41162 | **-104** | **-96** | **CAGAGGAAG** | **2,3** |
|  |  |  | **-100** | **-92** | **GGAAGGAAG** | **0,82** |
|  |  |  | 156 | 164 | CTTCCGCGG | 8,9 |
|  |  |  | 273 | 281 | CGCGGGAAG | 8,93 |
| TNFRSF10C | tumor necrosis factor receptor superfamily, member 10c | 39797 | -245 | -237 | CTTCCCCTT | 11,1 |
|  |  |  | **-125** | **-117** | **CTTCCTGGA** | **2,3** |
|  |  |  | -83 | -75 | GCTTGGAAG | 12,61 |
|  |  |  | **102** | **110** | **CTTCCTACC** | **1,65** |
|  |  |  | 286 | 294 | CTGGGGAAG | 12,18 |
| TP53 | tumor protein p53 | 112708 | -391 | -383 | CTTCCTAAA | 5,03 |
|  |  |  | **-323** | **-315** | **CTTCCTTCC** | **0,82** |
|  |  |  | -319 | -311 | CTTCCACCC | 8,8 |
|  |  |  | **-147** | **-139** | **CTTCCTCCG** | **0,13** |
|  |  |  | 92 | 100 | CTTCCCTGG | 11,92 |
|  |  |  | 236 | 244 | GGATGGAAG | 9,62 |
| TRADD | TNFRSF1A-associated via death domain | 15934 | -579 | -571 | CTTCCAAAG | 13,82 |
|  |  |  | -458 | -450 | CTTCCGGGC | 8,76 |
|  |  |  | **-198** | **-190** | **CTTCCTGCC** | **0** |
|  |  |  | -103 | -95 | GACGGGAAG | 10,96 |
|  |  |  | -11 | -3 | ACCCGGAAG | 8,9 |
| TULP2 | tubby-like protein 2 | 115324 | **17** | **25** | **TGGAGGAAG** | **0,13** |
| WNT3 | wingless-type MMTV integration site family, member 3 | 18466 | -553 | -545 | CCCGGGAAG | 11,1 |
|  |  |  | -495 | -487 | GGCTGGAAG | 8,8 |
|  |  |  | -171 | -163 | CTTCCCGGA | 11,1 |
|  |  |  | **95** | **103** | **CTTCCTCCT** | **0,13** |
|  |  |  | 245 | 253 | CCGGGGAAG | 11,1 |
| WNT6 | wingless-type MMTV integration site family, member 6 | 24177 | **-627** | **-619** | **CTTCCTCTC** | **2,16** |
|  |  |  | -562 | -554 | CCTGGGAAG | 12,74 |
|  |  |  | -513 | -505 | CTTCCAGCC | 8,8 |
|  |  |  | -452 | -444 | CTTCCCAGC | 12,61 |
|  |  |  | **-442** | **-434** | **CTTCCTTGC** | **2,99** |
| XCR1 | X-C motif chemokine receptor 1 | 116753 | **-649** | **-641** | **CTTCCTGAC** | **3,25** |
|  |  |  | -505 | -497 | CTTCCACAA | 12,18 |
|  |  |  | **-424** | **-416** | **CTTCCTGCT** | **0,13** |
|  |  |  | **-301** | **-293** | **CTTCCTCAG** | **3,38** |
|  |  |  | 68 | 76 | GCCTGGAAG | 10,96 |
|  |  |  | **188** | **196** | **CTTCCTACC** | **1,65** |
| ZC3HAV1 | zinc finger CCCH type, antiviral 1 | 38771 | -673 | -665 | TAAGGGAAG | 11,92 |
|  |  |  | **-223** | **-215** | **CTTCCTCAA** | **3,38** |
|  |  |  | -176 | -168 | GCTGGGAAG | 12,61 |
|  |  |  | **-135** | **-127** | **CGCAGGAAG** | **0,13** |

Supplementary Table S4. Soft agar assay colony formation assay. Cells were counted either with staining to confirm the number of colonies grown in each well. For each experimental group, cells were seeded quadruplicates and the colonies ≥ 20 um were counted with care trying to avoid artifacts. The resulting colony numbers are given as mean±SD

| **Cell Group** | **Transfection** | **# Cells Seeded** | **Colonies Formed**  **(mean±SD)** |
| --- | --- | --- | --- |
| **SK-N-BE (2)** | - | 100 | 25±11 |
| **CD133+** | - | 100 | 33±9 |
| **CD133-** | - | 100 | 24±11 |
| **CD133-** | pcDNA3.1 | 100 | 32±3 |
| **CD133-** | Elk1-VP16/pcDNA3.1 | 100 | 37±10 |
| **CD133-** | pCMV6-Flag | 100 | 24±10 |
| **CD133-** | Elk-1/pCMV6-Flag | 100 | 50±15 |
| **CD133+** | scrRNA | 100 | 26±9 |
| **CD133+** | siElk-1 | 100 | 18±5 |
